# Synthesis of 1,3-Enynes by Iron-Catalyzed Propargylic C–H Functionalization: An Alkyne Analogue for the Eschenmoser Methenylation

**DOI:** 10.1021/acs.orglett.4c00696

**Published:** 2024-04-11

**Authors:** Shalini Dey, Aaron D. Charlack, Austin C. Durham, Jin Zhu, Yidong Wang, Yi-Ming Wang

**Affiliations:** † Department of Chemistry, University of Pittsburgh, Pittsburgh, Pennsylvania 15260, United States; ‡ School of Chemistry & Chemical Engineering, Yangzhou University, Yangzhou, Jiangsu 225002, China

## Abstract

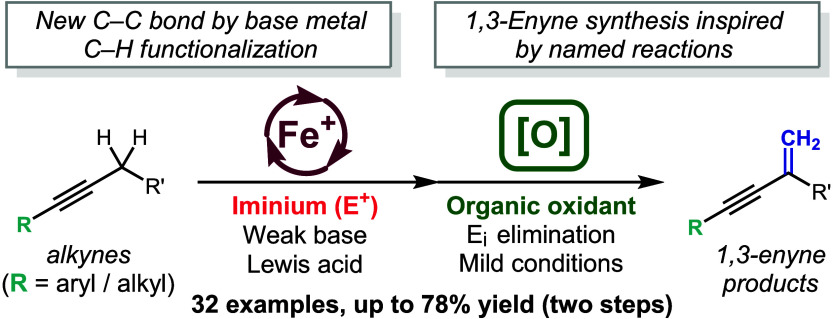

A two-step protocol for the conversion of alkyl-substituted alkynes to 1,3-enynes is reported. In this α-methenylation process, an iron-catalyzed propargylic C–H functionalization delivers tetramethylpiperidine-derived homopropargylic amines which undergo facile Cope elimination upon N-oxidation to afford the enyne products. A range of aryl alkyl and dialkyl acetylenes were found to be suitable substrates for this process, which constitutes an alkyne analogue for the Eschenmoser methenylation of carbonyl derivatives. In addition, a new bench-stable precatalyst for iron-catalyzed propargylic C–H functionalization is reported.

Conjugated enynes are prominent and fundamental substructures found in a range of natural products, pharmaceuticals, and other bioactive molecules (Figure 1).^1^ Additionally, 1,3-enynes serve as versatile building blocks for the synthesis of polysubstituted aromatic compounds, conjugated dienes, and chiral allenes.^2^

**Figure 1:**
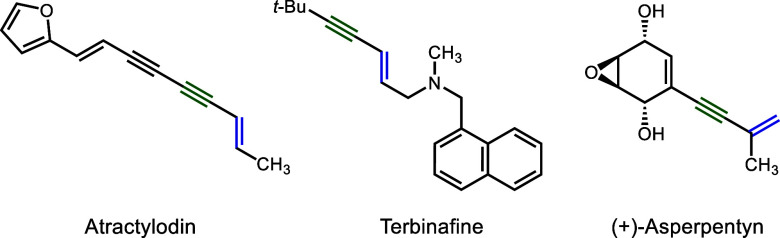
1,3-Enyne motif in natural products and bioactive molecules.

A number of general approaches convert difunctional starting materials to 1,3-enynes through standard functional group interconversions. These approaches include dehydration of propargylic alcohols,^3^ Wittig olefination of conjugated ynones, and Corey–Fuchs alkynylation of conjugated enals.^4^ Apart from these transformations, transition metal catalysis has also enabled the assembly of 1,3-enynes through C–C bond forming coupling reactions. The most common method to access 1,3-enynes is transition metal catalyzed cross-coupling reactions between alkynyl and alkenyl precursors, with Pd-catalyzed transformations being the most versatile and well-developed (Scheme 1A).^5^ The Sonogashira reaction between terminal alkynes and alkenyl (pseudo)halides is the most widely used among them, due to the convenience and availability of the starting materials.^6^ Palladium catalysis has also been successfully applied to the synthesis of 1,3-enynes by the three-component coupling of aryl iodides, internal alkynes, and alkynylsilanes,^7^ by Heck-type coupling of vinylarenes and alkynyl bromides,^8^ and by the oxidative coupling of terminal alkynes with alkenes or vinylmetals.^9^ As Earth-abundant alternatives to palladium catalysis, iron-^10^ or copper-^11^ based catalysts have also been successfully employed in cross-coupling reactions for the synthesis of 1,3-enynes. Additionally, Cu or Cu/Fe-mixed catalysts have also been used in the coupling of vinylmetal species with alkynyl halides.^12^ Transition metal catalyzed dimerization^13^ or trimerization^14^ of alkynes can also be highly effective approaches for 1,3-enyne synthesis (Scheme 1B). In some special cases, the coupling of two distinct alkynes through Pd- or Co-catalysis has also been achieved.^15^ The transition metal catalyzed coupling of terminal alkynes with carbene precursors has also proved to be a highly general approach for the synthesis of enynes (Scheme 1C).^16^

**Scheme 1:**
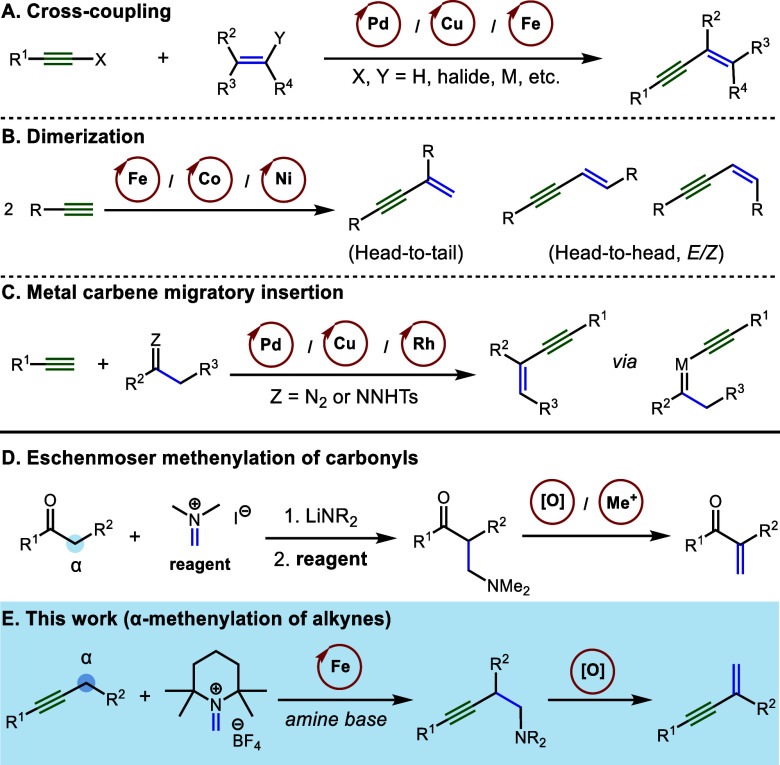
Previous Approaches to 1,3-Enynes through Transition Metal Catalysis

These approaches generally involve the coupling of two building blocks, which are often prefunctionalized, or they require the manipulation of di- or trifunctionalized starting materials. The direct installation of a methylene group at the α-position of an aryl alkyl or dialkyl acetylene would constitute a more direct approach for the synthesis of 4-substituted or 2,4-disubstituted 1,3-enynes. Such a process would resemble the celebrated Eschenmosher methenylation of carbonyl derivatives for the synthesis of conjugated enones.^17^ Due to the relatively high acidity of the C–H bonds α to a carbonyl group (p*K*_a_ ≈ 20 to 25), selective deprotonation to form an enolate is possible using various lithium amide reagents. Subsequent aminomethylation with an iminium reagent (Eschenmoser salt) would afford a Mannich base, which could then be converted to the corresponding α-methylene carbonyl compound by Cope or Hofmann elimination (Scheme 1D). We wondered whether a similar process could be developed for the more weakly acidic (p*K*_a_ ≈ 35 to 40) propargylic position of alkynes.

Previously our group developed a catalytic method for the C–H functionalization of unsaturated hydrocarbons by employing cationic iron complexes for π-activation to increase the acidity of the propargylic, allylic, or allenic C–H bonds and enable their deprotonation by weak amine or pyridine bases to generate nucleophilic organoiron species. These organometallic nucleophiles undergo subsequent functionalization with carbonyl and iminium electrophiles to generate α-C–H functionalization products.^18^ We hypothesized that allenyliron intermediates generated from alkyne substrates could react with an Eschenmosher salt to give homopropargylic amine products. These adducts could then undergo elimination of the pendent dialkylamino group to afford 1,3-enynes, allowing for the development of a protocol that is formally and mechanistically analogous to the Eschenmoser methenylation (Scheme 1E). In this Communication, we report the successful development of this process through the use of an *in situ* generated iminium electrophile to give homopropargylic 2,2,6,6-tetramethylpiperidine derivatives. Upon N*-*oxidation, spontaneous Cope elimination occurred to deliver the desired 1,3-enyne products.

We employed a hydride abstraction strategy for *in situ* formation of the requisite iminium intermediate.^19^ A mixture of tritylium tetrafluoroborate (Ph_3_C^+^BF_4_^–^) and 1,2,2,6,6-pentamethylpiperidine (**2**) was first stirred at rt for 1 h in toluene to generate iminium salt **2′**.^18a,18b,18e^ Using [Cp*Fe(CO)_2_(thf)]^+^[BF_4_]^−^ ([Fp*(thf)]^+^BF_4_^–^, **10a**) as the catalyst and applying previously reported conditions, desired aminomethylation product **3a** was observed in 10% yield by NMR analysis (Table 1, entry 1). While switching the solvent to trifluorotoluene was found to be beneficial (entry 2), the addition of BF_3_·Et_2_O as an additive resulted in a dramatic (and unexpected) improvement of the yield to 80% (entry 3). Other Lewis acids were, therefore, explored as additives. The use of a substoichiometric amount of zinc bistriflimide (Zn(NTf_2_)_2_) in place of BF_3_·Et_2_O was found to further enhance the yield (entries 5–6), while use of zinc triflate gave a poor outcome, and other metal bistriflimide salts were less effective (entries 8–12).^20^ Finally, the reaction conditions were further optimized by additional adjustments of Lewis acid and base stoichiometries (entry 7), delivering amine **3a** in 86% isolated yield. Although 3.0 and 4.0 equiv of TMPH gave identical yields for model substrate **1a**, 4.0 equiv was found to be more general for more challenging substrates and was therefore chosen as the standard condition for subsequent investigations of the substrate scope. Control experiments showed the necessity of both base and catalyst in this reaction (see Supporting Information for details).

**Table 1:**
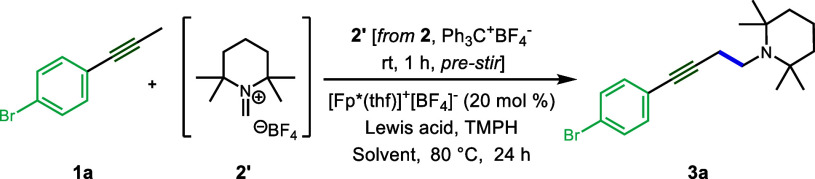
Optimization of the Fe-Catalyzed α-C–H Functionalization Step^a^

Entry	L.A.	TMPH/L.A. equiv	Solvent	Yield [%]^b^
1	-	3.0/0	PhCH_3_	10
2	-	3.0/0	PhCF_3_	35
3	BF_3_·OEt_2_	3.0/2.5	PhCF_3_	80
4	BF_3_·OEt_2_	2.0/2.5	PhCF_3_	4
5	Zn(NTf_2_)_2_	3.0/0.15	PhCF_3_	76
6	Zn(NTf_2_)_2_	3.0/0.30	PhCF_3_	85
**7**	**Zn(NTf**_**2**_**)**_**2**_	**3.0/0.58**	**PhCF**_**3**_	**92 (86)**^c^
8	Zn(OTf)_2_	3.0/0.58	PhCF_3_	5
9	Mg(NTf_2_)_2_	3.0/0.58	PhCF_3_	76
10	Ca(NTf_2_)_2_	3.0/0.58	PhCF_3_	24
11	LiNTf_2_	3.0/0.58	PhCF_3_	43
12	AgNTf_2_	3.0/0.58	PhCF_3_	56

a Reaction conditions. **1a** (0.3 mmol, 1.0 equiv), **2** (2.0 equiv), Ph_3_C^+^BF_4_^–^ (2.0 equiv), TMPH, [Fp*(thf)]^+^BF_4_^–^ (20 mol %), Lewis acid, and dry solvent [0.2 M] at 80 °C for 24 h.

b The yield was determined by ^1^H NMR spectroscopy of the crude material, using 1,1,2,2-tetrachloroethane as the internal standard.

c Isolated yield, TMPH (4.0 equiv) used.

To form the enyne, purified samples of **3a** were then subjected to oxidation with 3-chloroperoxybenzoic acid (*m*-CPBA) to generate the *N*-oxide. Pleasingly, the amine was found to undergo oxidation and subsequent Cope elimination under very mild conditions (0 °C, THF, 1 h) to deliver **4a** in a 78% isolated yield over two steps (Scheme 2). Little (<5%) to no overoxidation to the epoxide was observed under these optimized conditions. It was subsequently found that after filtration through SiO_2_ gel to remove excess base and iron residue, crude products **3** could be subjected directly to oxidation and Cope elimination to minimize material loss and improve the overall yield of the 1,3-enyne.

**Scheme 2:**
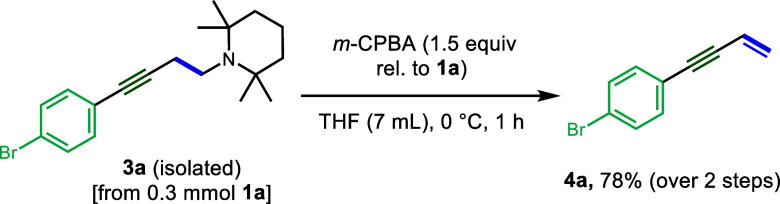
Cope Elimination

With an effective two-step procedure in hand, we evaluated the scope of alkynes that could be converted to 1,3-enynes by using this process (Table 2). The protocol was compatible with electron-rich (e.g., **1g**, **1i**, **1q**) as well as electron-poor (**1f**, **1l**, **1p)** aryl substituents. Substitution at the *ortho* position (**1h**, **1k**), including di-*ortho* substitution (**1j**), was well tolerated under these reaction conditions. Functional groups, including esters (**1l**, **1q**), sulfonamides (**1f**, **1y**), a phthalimide (**1v**), and an aryl-substituted alkene (**1o**), also gave moderate to good yields. Electron-rich heterocycles (**1g**, **1q**) and a 2,6-dichloropyridine ring (**1zb**) were also found to be compatible with this protocol. Unsymmetrical dialkyl alkyne substrates could also be employed. In cases where the alkyne α-positions are non-benzylic, functionalization took place cleanly (>20:1 r.r.) at the less hindered α-position (**1r**–**1zb**) to give the corresponding 1,3-enyne in moderate to good yields. Regioselectivity was unaffected by the presence of nitrogen or oxygen substituents on the alkyl chain (**1r**, **1u**−**1zb**). However, substrates that have a benzylic-propargylic position were observed to functionalize at that position with high selectivity (>20:1 r.r., **1zc**–**1zf**), in preference to a non-benzylic propargylic position.

**Table 2:**
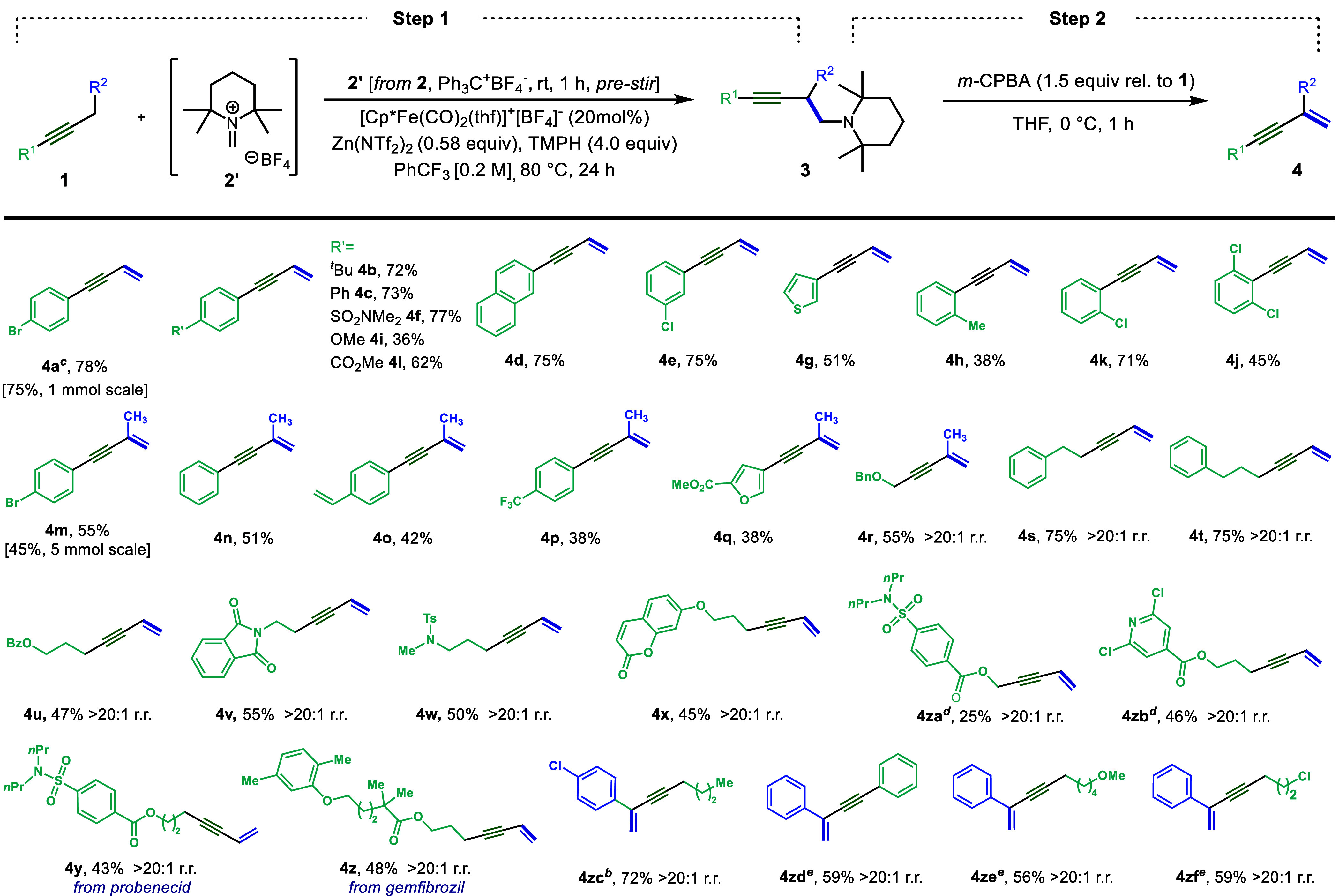
Substrate Scope^a^

a Step 1: **1** (0.3 mmol, 1.0 equiv), **2** (2.0 equiv), Ph_3_C^+^BF_4_^–^ (2.0 equiv), TMPH (4.0 equiv), [Fp*(thf)]^+^BF_4_^–^ (20 mol %), Zn(NTf_2_)_2_ (0.58 equiv), and PhCF_3_ [0.2 M] at 80 °C for 24 h. Step 2: Crude material from Step 1 in dry THF (7 mL) and *m*-CPBA (1.5 equiv rel. to **1**) at 0 °C for 1 h. Isolated yield over two steps.

b Step 1: Mg(NTf_2_)_2_ (0.5 equiv), TMPH (5.0 equiv), 70 °C. Step 2: Crude material from Step 1 in dry THF (7 mL) and *m*-CPBA (1.5 equiv rel. to **1zc**) at 0 °C for 5 min.

c Pure **3a** isolated from Step 1. Isolated yield over two steps.

d On 0.15 mmol scale.

e Step 1: TMPH (5.0 equiv), 70 °C, 24 h. Step 2: 0 °C, 10 min.

The above protocol was readily performed on a 1 mmol scale to deliver 0.16 g of the desired product **4a** (75% isolated yield over 2 steps). Likewise, the functionalization of substrate **1m** could be performed on a 5 mmol scale, also without a significant decrease in yield (0.49 g, 45% yield). To further demonstrate the utility of this protocol, we investigated several subsequent transformations of the products. The double bond of **4s** was further oxidized by using 3.0 equiv of *m*-CPBA to form epoxide **5** in 78% yield, and the triple bond of **4s** was selectively reduced by a CuH catalyst to form the corresponding conjugated diene **6** with 65% yield (*Z*/*E* = 3.5:1). Ozonolysis and cyclopropanation of **4a** gave the products **7** and **8** in 40% and 60% yield, respectively. Finally, Cu-catalyzed cascade cyclization of **4a** with *p*-toluidine gave rise to a disubstituted pyrrole derivative **9** in 43% yield (Scheme 3).

**Scheme 3:**
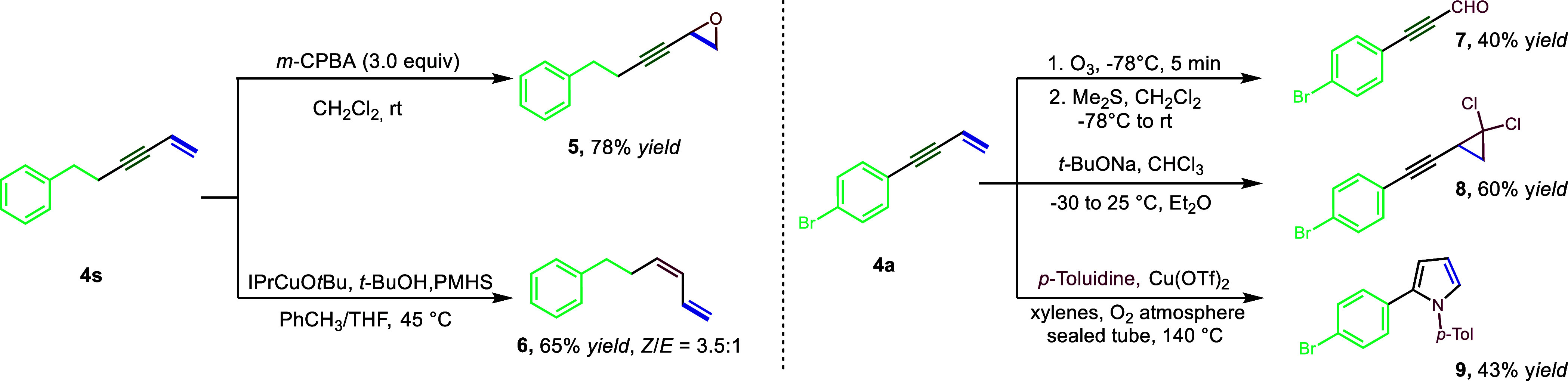
Synthetic Applications

During the course of mechanistic investigations (see the Supporting Information for the results of some preliminary studies on the role of the secondary Lewis acid), we explored some alternative strategies for accessing the cationic iron species. For instance, we considered the one-electron oxidation of [Fp*]_2_ dimer **10b** with AgBF_4_.^21^ Using this approach, the desired reactivity was observed, giving a 53% NMR yield of **3a** under standard reaction conditions (Scheme 4A). This encouraging result prompted us to explore other potentially bench-stable catalyst precursors for this transformation. While tetrahydrofuran complex **10a** demonstrates excellent catalytic activity, its water sensitivity and lability under dynamic vacuum complicate its synthesis and isolation. We turned to pyridine complexes of [Fp*]^+^ as alternatives, given their tunability and potentially improved robustness. While the unsubstituted pyridine complex **10c** was inactive, 2,6-difluoropyridine complex **10d** was found to exhibit reactivity similar to that of **10a** for three representative substrates (Scheme 4B). Moreover, catalyst samples that were stored on the benchtop for more than one month retained their full catalytic activity and did not show signs of degradation, either visually or by ^1^H NMR analysis. As such, complex **10d** may serve as a more user-friendly catalyst for accessing [Fp*]^+^ for the current protocol as well as other synthetic applications.

**Scheme 4:**
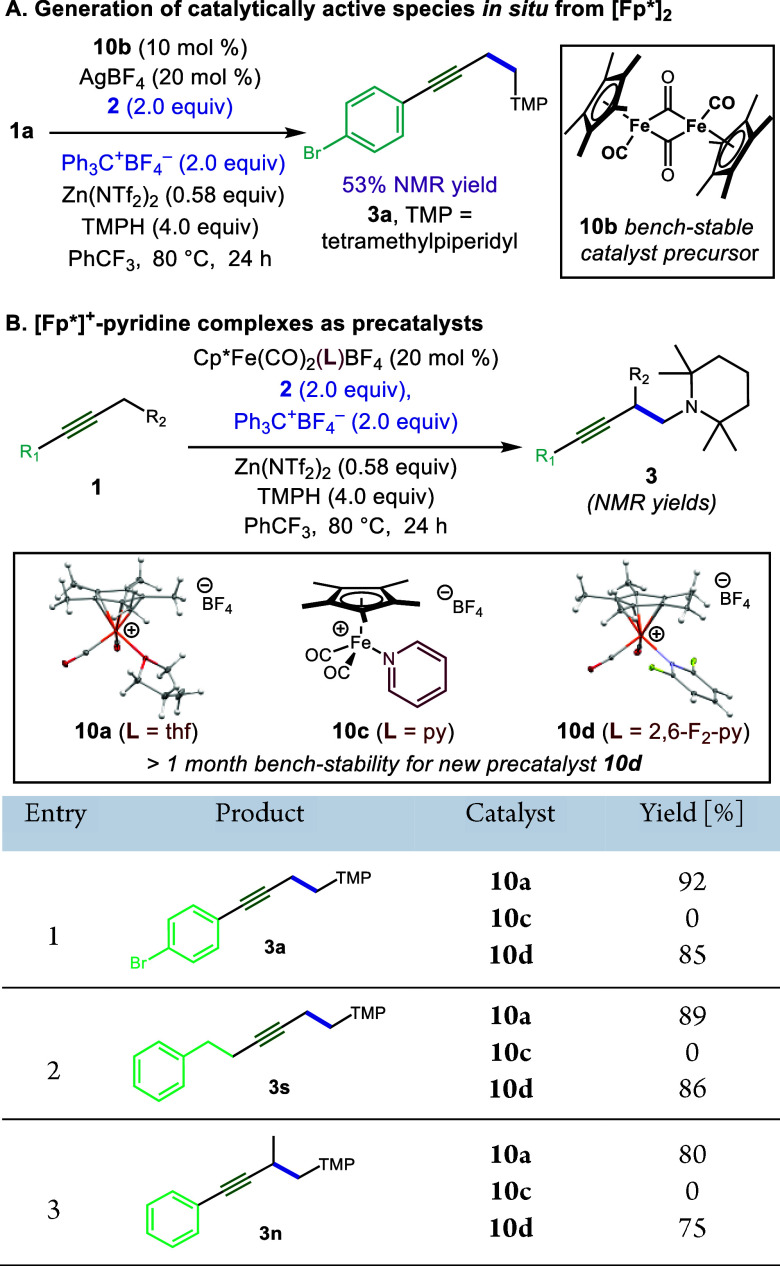
New [Fp*]^+^ Sources As Bench-Stable Precatalysts

In conclusion, we have developed a method for the methenylation of propargylic C**–**H bonds using inexpensive and readily prepared cyclopentadienyliron(II) dicarbonyl complexes as catalysts. Further investigations toward the formation of strategic C**–**C bonds using this approach are ongoing and will be reported in due course.

## Data Availability

The data underlying this study are available in the published article and its Supporting Information.

## Supporting Information Available

Experimental procedures and spectroscopic data for the substrates and products (PDF)
