# Supplementary material for: Synthesis of 1,3-Enynes by Iron-Catalyzed Propargylic C–H Functionalization: An Alkyne Analogue for the Eschenmoser Methenylation
Source: Org Lett. 2024 Apr 11;26(16):3355–60. doi: 10.1021/acs.orglett.4c00696 (PMC11059102; doi:10.1021/acs.orglett.4c00696)

Supporting Information

Synthesis of 1,3-Enynes by Iron-Catalyzed Propargylic C–H Functionalization: An Alkyne Analogue for the Eschenmoser Methenylation

*Shalini Dey,[a] Aaron D. Charlack,[a] Austin C. Durham,[a] Jin Zhu,[a] Yidong Wang,[a,b] and Yi-Ming Wang[a]**

[a]*Department of Chemistry, University of Pittsburgh, Pennsylvania 15260, United States.*

[b]*School of Chemistry & Chemical Engineering, Yangzhou University, Yangzhou, Jiangsu 225002, China*

E-mail: ym.wang@pitt.edu (Corresponding Author)

Contents

1. General Information S2

2. Optimization of reaction conditions S3

3. General procedures S6

4. Characterization Data for Products 3 .... S8

5. Characterization Data for Products 4 .... S9

6. Synthesis of substrates and characterization data for new compounds S26

7. Large scale synthesis and synthetic applications of product S31

8. Preliminary Mechanistic investigations

A. Kinetic isotopic effect experiments S36

B. Investigating the role of Lewis acid S37

9. New $[Fp^*]^+$ sources as bench-stable precatalysts S41

10. X-ray crystal structures of **10d** and **10a** S46

11. References S48

12. Copies of ^{1}H and ^{13}C NMR spectra for products and substrates S49

13. IR Data S100

General information:

General reagent information: Anhydrous toluene, 1,2-dichloroethane, tetrahydrofuran, and α,α,α-trifluorotoluene were purchased from Acros (AcroSeal packaging), Sigma Aldrich (Sure/Seal packaging), and Frontier Scientific (J&KSeal packaging), respectively, and were sparged with nitrogen before transferring into an argon-filled glovebox and used without further purification. Other dry solvents were obtained by distillation and storage over 3Å or 4Å molecular sieves. Triphenylcarbenium tetrafluoroborate and 1,2,2,6,6-pentamethylpiperidine were purchased from Sigma Aldrich, stored in an argon-filled glove box, and used as received. 2,2,6,6-Tetramethylpiperidine (TMPH) was freshly distilled and stored in an argon glovebox <1 month before use. All other reagents were purchased from Oakwood, Acros, Alfa Aesar, or Sigma Aldrich and used as received. Compounds were purified by flash column chromatography using SiliCycle *SiliaFlash® F60* silica gel, unless otherwise indicated.

General analytical information: New compounds were characterized by ^{1}H NMR, ^{13}C NMR and HRMS. Copies of the ^{1}H NMR and ^{13}C NMR spectra can be found at the end of the Supporting Information. ^{1}H and ^{13}C NMR spectra were recorded on Bruker 400 MHz or 500 MHz instruments. All ^{1}H NMR data are reported in δ units, parts per million (ppm), and were measured relative to the residual proton signal in the deuterated solvent at 7.26 ppm ($CDCl_3$). All ^{13}C NMR spectra are ^{1}H decoupled and reported in ppm relative to the solvent signal at 77.03 ppm ($CDCl_3$). Thin-layer chromatography (TLC) was performed on Silicycle 250 µm (analytical) or 1000 µm (preparative) silica gel plates. Compounds were visualized byirradiation with UV light, or by staining with iodine/silica gel, or potassium permanganate. Yields refer to isolated compounds, unless otherwise indicated. High resolution mass spectra were recorded on a Thermo Scientific Q-Exactivemass spectrometer. NMR yields were determined by using 1,1,2,2-tetrachloroethane as the internal standard for ^{1}H spectroscopy. High resolution mass spectra were obtained by quadrupole mass analyzer on a Bruker Daltonics, Inc. APEXIII 7.0 TESLA FTMS instrument (ESI). X-ray data were collected on a Bruker X8 Prospector Ultra single crystal diffractometer with Cu Kα radiation and an Apex II CCD detector.

Optimization of reaction conditions:

1. Optimization of solvent and temperature. [a]

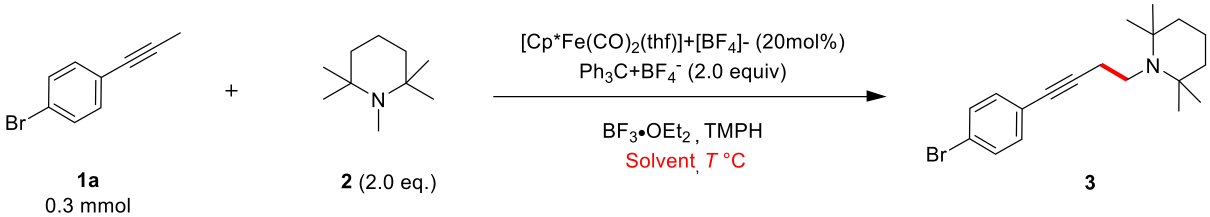

Entry	Solvent	*T* °C	NMR yield (%) [b]
1	DCE	80	64
2	$PhCH_3$	80	70
3	*p*-$C_6H_4Cl_2$	80	76
4	PhCl	80	75
5	$PhCF_3$	80	80
6	$PhCF_3$	60	78
7	$PhCF_3$	100	72

[a] All reactions were carried out with **1a** (0.3 mmol), **2** (2.0 equiv), $Ph_3C^+BF_4^-$ (2.0 equiv), TMPH (3.0 equiv), $Cp^*Fe(CO)_2(thf)BF_4$ (20 mol%), $BF_3.OEt_2$ (2.5 equiv) and dry solvent (0.2 M) at *T* °C for 24 h. [b] NMR yield was determined by using 1,1,2,2- tetrachloroethane as internal standard.

2. Optimization of concentration. [a]

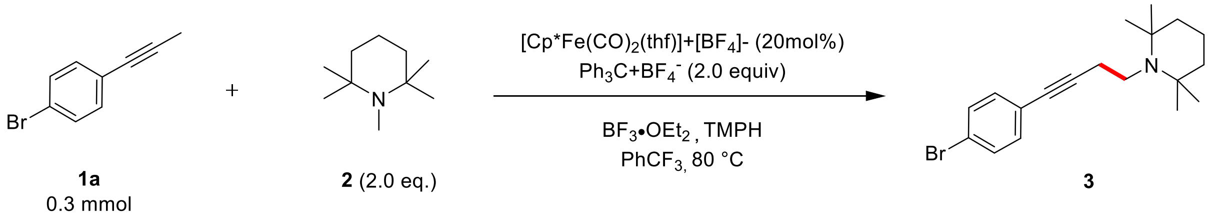

Entry	Concentration	NMR yield (%) [b]
1	0.5 M	47
2	0.33 M	77
3	0.2 M	80

[a] All reactions were carried out with **1a** (0.3 mmol), **2** (2.0 equiv), $Ph_3C^+BF_4^-$ (2.0 equiv), TMPH (3.0 equiv), Cp*Fe(CO)$_2$(thf)BF_4 (20 mol%), $BF_3.OEt_2$ (2.5 equiv) and dry $PhCF_3$ at 80 °C for 24 h. [b] NMR yield was determined by using 1,1,2,2- tetrachloroethane as internal standard.

3. Optimization of base and equivalence used. [a]

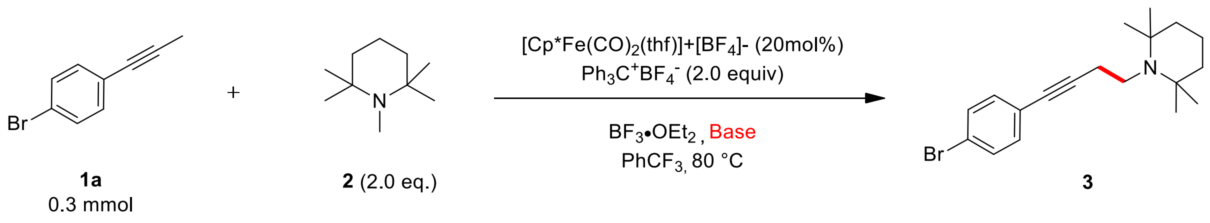

Entry	Base	Equivalence used	NMR yield (%) [b]
1	2,4,6-Collidine	3.0	39
2	2,6-Lutidine	3.0	0
3	TMPH	3.0	80
4	TMPH	2.5	37
5	TMPH	2.0	<4
6	TMPH	1.5	NP

[a] All reactions were carried out with **1a** (0.3 mmol), **2** (2.0 equiv), $Ph_3C^+BF_4^-$ (2.0 equiv), Base, Cp*Fe(CO)$_2$(thf)BF_4 (20 mol%), $BF_3.OEt_2$ (2.5 equiv) and dry $PhCF_3$ (0.2 M) at 80 °C for 24 h. [b] NMR yield was determined by using 1,1,2,2- tetrachloroethane as internal standard.

4. Optimization of Lewis acid used. [a]

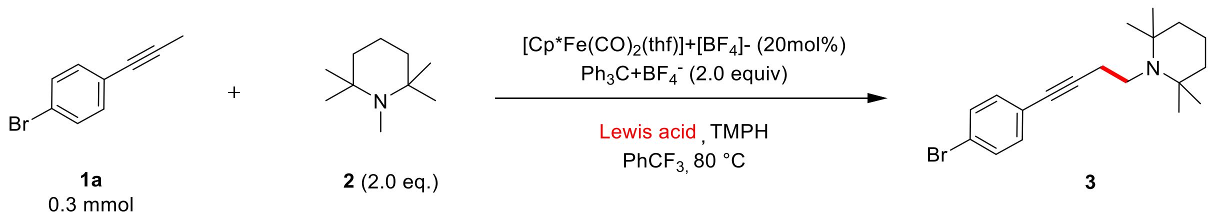

Entry	Lewis acid	Equivalence	NMR yield (%) [b]
1	-	0	35
2	$BF_3{\cdot}OEt_2$	1.5	76
3	$BF_3{\cdot}OEt_2$	2.5	80
4	$BF_3{\cdot}OEt_2$	3.5	6

5	$Zn(OTf)_2$	0.58	5
6	$Zn(NTf_2)_2$	0.15	76
7	$Zn(NTf_2)_2$	0.3	85
8	$Zn(NTf_2)_2$	0.58	92 (86)
9	$Mg(NTf_2)_2$	0.58	76
10	$Ca(NTf_2)_2$	0.58	24
11	$LiNTf_2$	0.58	43
12	$AgNTf_2$	0.58	56

[a] All reactions were carried out with **1a** (0.3 mmol), **2** (2.0 equiv), $Ph_3C^+BF_4^-$ (2.0 equiv), TMPH (3.0 equiv), $Cp^*Fe(CO)_2(thf)BF_4$ (20 mol%), Lewis acid and dry $PhCF_3$ (0.2 M) at 80 °C for 24 h. [b] NMR yield was determined by using 1,1,2,2- tetrachloroethane as internal standard.

Control experiments: [a]

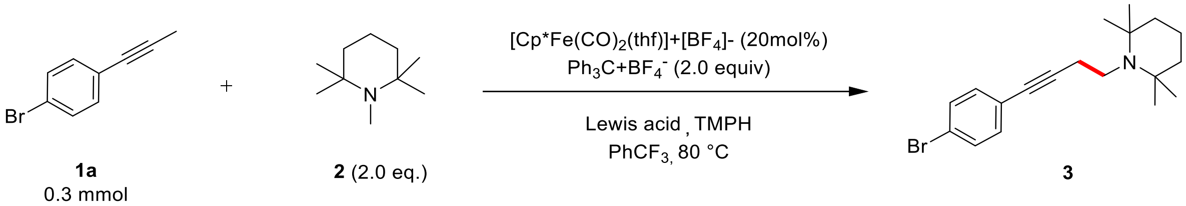

Entry	Lewis acid	TMPH	[Fe]	NMR yield (%) [b]
1	-	3.0	20 mol%	35
2	$BF_3·OEt_2$ (2.5 equiv)	-	20 mol%	NP
3	$Zn(NTf_2)_2$ (0.58 equiv)	4.0	-	NP

[a] All reactions were carried out with **1a** (0.3 mmol), **2** (2.0 equiv), $Ph_3C^+BF_4^-$ (2.0 equiv), TMPH, $Cp^*Fe(CO)_2(thf)BF_4$ (20 mol%), Lewis acid and dry $PhCF_3$ (0.2 M) at 80 °C for 24 h. [b] NMR yield was determined by using 1,1,2,2- tetrachloroethane as internal standard.

General Procedures

General Procedure A for the synthesis of enynes **4**:

General Procedure A Step 1:

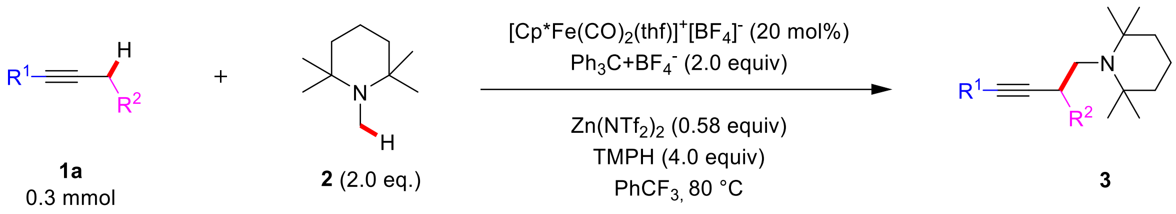

A reaction tube (13 mm × 100 mm, Fisherbrand, part # 14-959-35C) equipped with a magnetic stir bar was capped with a Teflon/silicone septum (Thermo/National part # C4015-66A) screw cap and flame dried under vacuum. The reaction tube was cooled under nitrogen and transferred into an argon-filled glovebox. In the glovebox, the suspension of 1,2,2,6,6-pentamethylpiperidine (**2**, 109 µL, 0.6 mmol, 2.0 equiv) and $Ph_3C^+BF_4^-$ (198 mg, 0.6 mmol, 2.0 equiv) in dry trifluorotoluene (1 mL) was stirred at room temperature for 1 h at r.t. to generate the iminium salt (**2'**). After an hour, $[Cp^*Fe(CO)_2(thf)]^+[BF_4]^-$ (20 mol %, 24.6 mg), $Zn(NTf_2)_2$ (108.9 mg, 0.174 mmol, 0.58 equiv), trifluorotoluene (0.5 mL), alkyne **1** (0.3 mmol, 1.0 equiv), and 2,2,6,6-tetramethylpiperidine (205 µL, 1.2 mmol, 4.0 equiv) were added to the reaction tube containing **2'** in rapid succession. The reaction tube was capped and removed from the glovebox. The reaction tube was then placed in an oil bath that was preheated to 80 °C, where it was stirred at this temperature for 24 h. After completion of the reaction, the reaction mixture was cooled to room temperature. The crude mixture was concentrated *in vacuo*, subjected to a short silica plug eluting with hexanes or a mixture of (ethyl acetate: hexanes) to remove any residual, unreacted alkyne and then a second solution (1:1 ethyl acetate: hexanes) to elute the desired tertiary amine **3**. The eluents were concentrated to dryness *in vacuo*, and the crude material was taken forward to Step 2 without further purification. Intermediate **3** was isolated and fully characterized in the case of products **3a** and **3n**. The NMR yield of **3** was determined by adding 1,1,2,2-tetrachloroethane (63.3 µL, 0.6 mmol, 2 equiv) as the internal standard and integrating the signals of the methylene protons at Carbon a and Carbon b in Figure 1 relative to the signal of the internal standard.

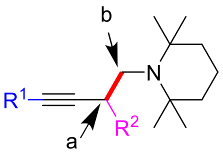

3

Figure 1

General Procedure A Step 2:

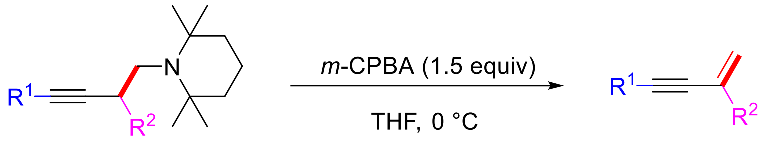

3 **4**

To a flame dried round bottom flask under nitrogen equipped with a stir bar was added a solution of **3** in dry tetrahydrofuran (2 mL), and the solution was cooled to 0 °C in an ice bath. A solution of *meta*-chloroperbenzoic acid (78 mg, 0.45 mmol, 1.5 equiv relative to the alkyne **1**) in dry tetrahydrofuran (5 mL) was added to the stirring solution at 0 °C dropwise for over 5 min. Subsequently, the reaction flask was kept at 0 °C for 1 h. After completion of the reaction, as determined by TLC analysis, the crude mixture was concentrated *in vacuo* and purified by column chromatography (SiO_2, hexanes: EtOAc unless otherwise specified) to provide the desired 1,3-enyne **4**.

General procedure B for the synthesis of alkyne substrates 1:

The decarboxylative cross coupling procedure is adapted from literature. [1] To a round bottom flask equipped with a stir bar were added the aryl halide (5.0 mmol, 1.0 equiv), but-2-ynoic acid (0.5 g, 6.0 mmol, 1.2 equiv), $PdCl_2(PPh_3)_2$ (35 mg, 0.050 mmol, 1 mol %), 1,4-bis(diphenylphosphino)butane (dppb, 42.6 mg, 0.10 mmol, 2 mol %). The flask was then evacuated and refilled with N_2. Anhydrous DMSO (10 mL, 0.5 M) was then added, followed by the addition of 1,8-diazabicyclo[5.4.0]undec-7-ene (DBU, 2.2 mL, 15 mmol, 3.0 equiv). The reaction mixture was stirred at 110 °C for 2 h. It was then cooled to room temperature and quenched by addition of saturated NH_4Cl solution (10 mL). The product was extracted with Et_2O (3 × 10 mL), and the combined organic layers were washed with brine (10 mL). The organic phase was further dried over $MgSO_4$ and concentrated. The crude mixture was purified by vacuum distillation or flash column chromatography to afford the title compound.

General Procedure C for the synthesis of alkyne substrates 1:

Amide coupling procedure was adapted from the literature. [2] To a stirred solution of the alkynyl alcohol (5.0 mmol, 1.0 equiv) and carboxylic acid (5.0 mmol, 1.0 equiv) in dichloromethane (15 mL) were added 1-ethyl-3-(3-dimethylaminopropyl)carbodiimide hydrochloride (1.2 g, 6.0 mmol, 1.2 equiv) and 4-(dimethylamino)pyridine (30 mg, 0.25 mmol, 5 mol %), and the reaction mixture was stirred at room temperature (25 °C) overnight. Subsequently, the reaction mixture was poured into a separatory funnel and washed with 1 M HCl (20 mL). The aqueous layer was extracted with CH_2Cl_2 (3 × 10 mL), and the combined organic layers were washed with brine (10 mL). The organic phase was dried over Na_2SO_4 and concentrated. After the

removal of the solvent, the residue was purified by flash column chromatography to afford the title compound.

Characterization Data for Products 3:

Compounds **3** were generally used directly in Step 2 of General Procedure A without careful purification and characterization. In the case of **3a** and **3n**, the compounds were isolated for the purpose of identifying key NMR signals that were used for determination of internal standard yield.

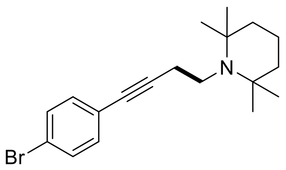

3a

1-(4-(4-Bromophenyl) but-3-yn-1-yl)-2,2,6,6-tetramethylpiperidine (3a):

Prepared following general procedure A Step 1, using 1,2,2,6,6-pentamethylpiperidine (**2**, 109 µL, 0.6 mmol, 2.0 equiv), $Ph_3C^+BF_4^-$ (198 mg, 0.6 mmol, 2.0 equiv), $[Cp^*Fe(CO)_2(thf)]^+[BF_4]^-$ (20 mol %, 24.6 mg), $Zn(NTf_2)_2$ (108.9 mg, 0.174 mmol, 0.58 equiv), trifluorotoluene (1.5 mL), 1-bromo-4-(prop-1-yn-1-yl)benzene (**1a**, 58.5mg, 0.3 mmol, 1.0 equiv), and 2,2,6,6-tetramethylpiperidine (205 µL, 1.2 mmol, 3.0 equiv) at 80 °C for 24 h. The crude residue was purified by flash column chromatography, eluting with 1:10 (ethyl acetate: hexanes) to provide the product **3a** as a colorless oil (89.8 mg, 86% yield).

^{1}H NMR (500 MHz, $CDCl_3$) δ (ppm) 7.41-7.39 (m, 2H), 7.26-7.25 (m, 2H), 2.73-2.70 (dd, *J* =9.7, 6.8 Hz, 2H), 2.44-2.40 (m, 2H), 1.52 (m, 2H), 1.41-1.39 (m, 4H), 1.06 (s, 12H).

^{13}C NMR (125 MHz, $CDCl_3$) δ (ppm) 133.2, 131.5, 123.2, 121.7, 90.5, 80.5, 54.8, 44.7, 41.2, 27.7, 25.8, 17.8.

HRMS (ESI) calcd. for $C_{19}H_{27}BrN$ $[M+H]^+$: 348.1321, found: 348.1323.

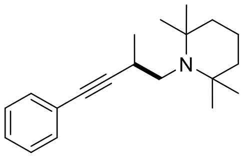

3n

2,2,6,6-Tetramethyl-1-(2-methyl-4-phenylbut-3-yn-1-yl) piperidine (3n):

Prepared following general procedure A Step 1, using 1,2,2,6,6-pentamethylpiperidine (**2**, 109 µL, 0.6 mmol, 2.0 equiv), $Ph_3C^+BF_4^-$ (198 mg, 0.6 mmol, 2.0 equiv), $[Cp^*Fe(CO)_2(thf)]^+[BF_4]^-$ (20 mol %, 24.6 mg), $Zn(NTf_2)_2$ (108.9 mg, 0.174 mmol, 0.58 equiv), trifluorotoluene (0.5 mL), but-1-yn-1-ylbenzene (**1n**, 43 µL, 0.3 mmol, 1.0 equiv) and 2,2,6,6-tetramethylpiperidine (205 µL, 1.2 mmol, 3.0 equiv) at 80 °C for 48 h. The crude residue was purified by flash column chromatography, eluting with 1:10 (ethyl acetate: hexanes) to provide the product **3n** as a colorless oil (52.7 mg, 62% yield).

^{1}H NMR (500 MHz, $CDCl_3$) δ (ppm) 7.41-7.39 (m, 2H), 7.29-7.24 (m, 3H), 2.81-2.71 (m, 2H), 2.56-2.47 (m, 1H), 1.45-1.43 (m, 4H), 1.26 (s, 2H), 1.23 (d, J=6.59 Hz, 3H), 1.11 (s, 6H), 1.06(s, 6H).

^{13}C NMR (125 MHz, $CDCl_3$) δ (ppm) 131.6, 128.2, 127.3, 124.5, 95.7, 81.6, 54.8, 51.1, 41.5, 30.8, 29.8, 19.5, 18.0.

HRMS (ESI) calcd. for $C_{20}H_{30}N$ $[M+H]^+$: 284.4668, found: 284.4669.

Characterization Data for Products 4:

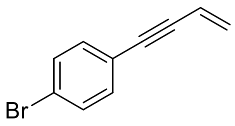

4a

1-Bromo-4-(but-3-en-1-yn-1-yl) benzene (4a):

Prepared following general procedure A using 1-bromo-4-(prop-1-yn-1-yl) benzene (**1a**, 58.5 mg, 0.3 mmol, 1.0 equiv). The crude residue from Step 1 was purified by flash column chromatography, eluting with 1:10 (ethyl acetate: hexanes) to provide the product **3a** as a colorless oil (89.8 mg, 86% yield). Pure **3a** (89.8 mg, 1 equiv) was used in Step 2. The crude residue from Step 2 was purified by flash column chromatography, eluting with pentane to provide product **4a** as a colorless oil (48.4 mg, 78% yield over 2 steps).

^{1}H NMR (500 MHz, $CDCl_3$) δ (ppm) 7.46-7.43 (m, 2H), 7.31-7.28 (m, 2H), 6.00 (dd, *J* =11.2, 17.6 Hz, 1H), 5.74 (dd, *J* =1.9, 17.5 Hz, 1H), 5.57 (dd, *J* =1.9, 11.2 Hz, 1H).

^{13}C NMR (125 MHz, $CDCl_3$) δ (ppm) 133.1, 131.7, 127.5, 122.6, 122.2, 117.0, 89.3, 89.0.

HRMS (ESI) calcd. for $C_{10}H_8Br$ $[M+H]^+$: 206.9803, found: 206.9804.

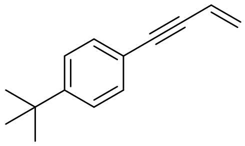

4b

1-(But-3-en-1-yn-1-yl)-4-(*tert*-butyl) benzene (4b):

Prepared following general procedure A using 1-(*tert*-butyl)-4-(prop-1-yn-1-yl)benzene (**1b**, 51.6 mg, 0.3 mmol, 1.0 equiv). The crude residue from step 2 was purified by flash column chromatography, eluting with pentane to provide the product **4b** as a colorless oil (39.8 mg, 72% yield over 2 steps).

^{1}H NMR (500 MHz, $CDCl_3$) δ (ppm) 7.38 (d, *J* =8.4 Hz, 2H), 7.34 (d, *J* =8.4 Hz, 2H), 6.02 (dd, *J* =11.2, 17.5 Hz, 1H), 5.72 (dd, *J* =2.0, 17.5 Hz, 1H), 5.52 (dd, *J* =2.0, 11.2 Hz, 1H), 1.32 (s, 9H).

^{13}C NMR (125 MHz, $CDCl_3$) δ (ppm) 151.5, 131.3, 126.4, 125.3, 120.1, 117.3, 90.1, 87.4, 34.78, 31.17.

HRMS (ESI) calcd. for $C_{14}H_{17}$ $[M+H]^+$: 185.1324, found: 185.1326.

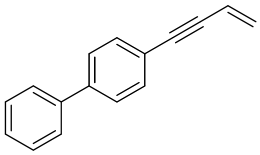

4c

4-(But-3-en-1-yn-1-yl)-1,1'-biphenyl (4c):

Prepared following general procedure A using 4-(prop-1-yn-1-yl)-1,1'-biphenyl (**1c**, 57.6 mg, 0.3 mmol, 1.0 equiv). The crude residue from step 2 was purified by flash column chromatography, eluting with pentane to provide the product **4c** as a white solid (44.7 mg, 73% yield over 2 steps). Spectroscopic data is consistent with previous reports. [3]

^{1}H NMR (500 MHz, $CDCl_3$) δ (ppm) 7.60 – 7.58 (m, 2H), 7.56 (d, *J* = 8.5 Hz, 2H), 7.51 (d, *J* = 8.5 Hz, 2H), 7.44 (t, *J* = 7.5, 2H), 7.36 (t, *J* = 7.5, 1H), 6.04 (dd, *J* = 17.5, 11.0 Hz, 1H), 5.75 (dd, *J* = 17.5, 2.0 Hz, 1H), 5.56 (dd, *J* = 11.0, 2.0 Hz, 1H).

^{13}C NMR (125 MHz, $CDCl_3$) δ (ppm) 141.9, 140.3, 132.0, 128.8, 127.6, 127.0, 127.0, 126.9, 122.0, 117.2, 90.0, 88.8.

HRMS (ESI) calcd. for $C_{16}H_{13}$ $[M+H]^+$: 205.1011, found: 205.1011.

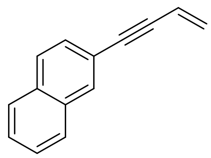

4d

2-(But-3-en-1-yn-1-yl)naphthalene (4d):

Prepared following general procedure A using 2-(prop-1-yn-1-yl)naphthalene (**1d**, 50 mg, 0.3 mmol, 1.0 equiv). The crude residue from step 2 was purified by flash column chromatography, eluting with hexanes to provide the product **4d** as a yellow solid (40.1 mg, 75% yield over 2 steps). Spectroscopic data is consistent with previous reports. [4]

^{1}H NMR (500 MHz, $CDCl_3$) δ (ppm) 7.94 (s, 1H), 7.71-7.75 (m, 3H), 7.41- 7.48 (m, 3H), 6.09 (dd, *J* = 17.2, 11.2 Hz, 1H), 5.71 (dd, *J* = 17.2, 2.1 Hz, 1H), 5.51 (dd, *J* = 11.2, 2.1 Hz, 1H).

^{13}C NMR (125 MHz, $CDCl_3$) δ (ppm) 133.1, 132.9, 131.6, 128.4, 128.1, 127.9,127.9, 127.2, 126.8, 126.7, 120.5, 117.3, 90.5, 88.6.

HRMS (ESI) calcd. for $C_{14}H_{11}$ $[M+H]^+$: 179.0855, found: 179.0853.

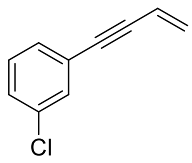

4e

1-(But-3-en-1-yn-1-yl)-3-chlorobenzene (4e):

Prepared following general procedure A using 1-chloro-3-(prop-1-yn-1-yl)benzene (**1e**, 45.2 mg, 0.3 mmol, 1.0 equiv). The crude residue from step 2 was purified by flash column chromatography, eluting with hexanes to provide the product **4e** as a colorless oil (36.5 mg, 75% yield over 2 steps).

^{1}H NMR (500 MHz, $CDCl_3$) δ (ppm) 7.46 (t, J = 1.66, 1H), 7.35 (dq *J* = 7.42, 1.33 Hz, 1H), 7.32 (dq, *J* = 8.56, 1.99 Hz, 1H), 7.28 (dd, *J* = 9.0, 1.23 Hz, 1H), 6.04 (dd, *J* = 17.5, 11.2, 1H), 5.79 (dd, *J* = 17.5, 2.0, 1H), 5.60 (dd, *J* = 11.2, 2.0, 1H).

^{13}C NMR (125 MHz, $CDCl_3$) δ (ppm) 134.3, 131.5, 129.8, 129.6, 128.6, 127.8, 125.0, 116.9, 89.3, 88.6.

HRMS (ESI) calcd. for $C_{10}H_8Cl$ $[M+H]^+$: 163.0309, found: 163.0307.

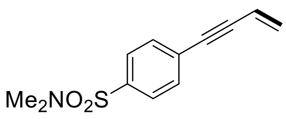

4f

4-(But-3-en-1-yn-1-yl)-*N*,*N*-dimethylbenzenesulfonamide (4f):

Prepared following general procedure A using *N*,*N*-dimethyl-4-(prop-1-yn-1-yl)benzenesulfonamide (**1f**, 67 mg, 0.3 mmol, 1.0 equiv). The crude residue from step 2 was purified by flash column chromatography, eluting with 1:3 (ethyl acetate: hexanes) to provide the product **4f** as a white foam (54.3 mg, 77% yield over 2 steps).

^{1}H NMR (500 MHz, $CDCl_3$) δ (ppm) 7.72 (d, *J* =8.5 Hz, 2H), 7.58 (d, *J* =8.5 Hz, 2H), 6.03 (dd, *J* =11.2, 17.5 Hz, 1H), 5.81 (dd, *J* =1.9, 17.5 Hz, 1H), 5.64 (dd, *J* =1.9, 11.2 Hz, 1H), 2.71 (s, 6H)

^{13}C NMR (125 MHz, $CDCl_3$) δ (ppm) 134.8, 131.9, 128.5, 127.9, 127.6, 116.6, 91.5, 88.2, 37.9.

HRMS (ESI) calcd. for $C_{12}H_{14}O_2NS$ $[M+H]^+$: 236.0739, found: 236.0739.

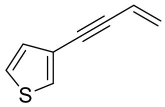

4g

3-(But-3-en-1-yn-1-yl) thiophene (4g):

Prepared following general procedure A using 3-(prop-1-yn-1-yl)thiophene (**1g**, 36.7 mg , 0.3 mmol, 1.0 equiv). The crude residue from step 2 was purified by flash column chromatography, eluting with pentane to provide the product **4g** as a colorless oil (20.5 mg, 51% yield over 2 steps).

^{1}H NMR (500 MHz, $CDCl_3$) δ (ppm) 7.44 (dd, *J* = 3.0, 1.0 Hz, 1H), 7.27 (dd, *J* = 5.0, 3.0 Hz, 1H), 7.12 (dd, *J* = 5.0, 1.0 Hz, 1H), 5.99 (dd, *J* = 17.5, 11.0 Hz, 1H), 5.72 (dd, *J* = 17.5, 2.0 Hz, 1H), 5.53 (dd, *J* = 11.0, 2.0 Hz, 1H).

^{13}C NMR (125 MHz, $CDCl_3$) δ (ppm) 129.8, 128.6, 126.7, 125.3, 122.2, 117.1, 87.6, 85.1.

HRMS (ESI) calcd. for C_8H_7S [M+H]$^+$: 135.0263, found: 135.0263.

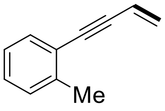

4h

1-(But-3-en-1-yn-1-yl)-2-methylbenzene (4h):

Prepared following general procedure A using 1-methyl-2-(prop-1-yn-1-yl)benzene (**1h**, 39.1 mg , 0.3 mmol, 1.0 equiv) (45% NMR yield for Step 1). The crude residue from step 2 was purified by flash column chromatography, eluting with pentane to provide the product **4h** as a colorless oil (16.2 mg, 38% yield over 2 steps). Spectroscopic data is consistent with previous reports. [5]

^{1}H NMR (500 MHz, $CDCl_3$) δ (ppm) 7.40 (d, *J* = 7.3 Hz, 1H), 7.24-7.16 (m, 2H), 7.14-7.09 (m, 1H), 6.00 (dd, J = 17.5, 11.1 Hz, 1H), 5.71 (dd, J = 17.5, 2.0 Hz, 1H), 5.53 (dd, J = 11.1, 2.0 Hz, 1H), 2.44 (s, 3H)

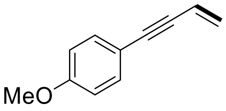

4i

1-(But-3-en-1-yn-1-yl)-4-methoxybenzene (4i):

Prepared following general procedure A using 1-methoxy-4-(prop-1-yn-1-yl)benzene (**1i**, 43.9 mg , 0.3 mmol, 1.0 equiv). The crude residue from step 2 was purified by flash column chromatography, eluting with 1:3 (ethyl acetate: hexanes) to provide the product **4i** as a colorless oil (17.1 mg, 36% yield over 2 steps).

^{1}H NMR (500 MHz, $CDCl_3$) δ (ppm) 7.38 (d, *J* = 8.8 Hz, 2H), 6.84 (d, *J* = 8.8 Hz, 2H), 6.00 (dd, *J* = 8.8, 5.6 Hz, 1H), 5.69 (dd, *J* = 8.8, 2.0 Hz, 1H), 5.49 (dd, *J* = 5.6, 2.0 Hz, 1H), 3.81 (s, 3H).

^{13}C NMR (125 MHz, $CDCl_3$) δ (ppm) 159.6, 133.0, 126.1, 117.4, 115.2, 114.0, 90.0, 86.9, 55.3.

HRMS (ESI) calcd. for $C_{11}H_{11}O$ [M+H]$^+$: 159.0804, found: 159.0805.

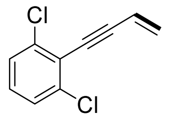

4j

2-(But-3-en-1-yn-1-yl)-1,3-dichlorobenzene (4j):

Prepared following general procedure A using 1,3-dichloro-2-(prop-1-yn-1-yl)benzene (**1j**, 55.5 mg, 0.3 mmol, 1.0 equiv) (55% NMR yield for Step 1). The crude residue from step 2 was purified by flash column chromatography, eluting with pentane to provide the product **4j** as a white paste (26.6 mg, 45% yield over 2 steps).

^{1}H NMR (500 MHz, $CDCl_3$) δ (ppm) 7.28-7.21 (m, 1H), 7.13-7.03 (m, 1H), 7.08 (dd, *J* = 17.5, 11.1 Hz, 1H), 6.07 (dd, *J* = 17.5, 11.1 Hz, 1H), 5.82 (dd, *J* = 17.5, 2.1 Hz, 1H), 5.62 (dd, *J* = 11.1, 2.1 Hz, 1H).

^{13}C NMR (125 MHz, $CDCl_3$) δ (ppm) 137.7, 129.5, 128.8, 127.9, 124.3, 123.7, 117.3, 99.0, 98.1, 84.3.

HRMS (ESI) calcd. for $C_{10}H_7Cl_2$ $[M+H]^+$: 198.0658, found: 198.0659.

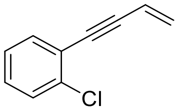

4k

1-(But-3-en-1-yn-1-yl)-2-chlorobenzene (4k):

Prepared following general procedure A using 1-chloro-2-(prop-1-yn-1-yl)benzene (**1k**, 45.2 mg, 0.3 mmol, 1.0 equiv). The crude residue from step 2 was purified by flash column chromatography, eluting with pentane to provide the product **4k** as a colorless oil (34.6 mg, 71% yield over 2 steps).

^{1}H NMR (500 MHz, $CDCl_3$) δ (ppm) 7.49 (dd, *J* = 7.6, 1.6 Hz, 1H), 7.40 (dd, *J* = 8.0, 1.6 Hz, 1H), 7.26 – 7.19 (m, 2H), 6.10 (dd, *J* = 17.6, 11.2 Hz, 1H), 5.84 (d, *J* = 17.6 Hz, 1H), 5.62 (d, *J* = 11.2 Hz, 1H).

^{13}C NMR (125 MHz, $CDCl_3$) δ (ppm) 135.8, 133.3, 129.4, 129.3, 127.9, 126.5, 123.1, 117.0, 93.2, 86.7.

HRMS (ESI) calcd for $C_{10}H_8Cl$ $[M+H]^+$: 163.0309, found: 163.0307.

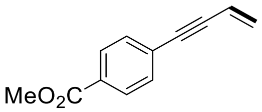

4l

Methyl 4-(but-3-en-1-yn-1-yl)benzoate (4l):

Prepared following general procedure A using methyl 4-(prop-1-yn-1-yl)benzoate (**1l**, 52.3 mg, 0.3 mmol, 1.0 equiv). The crude residue from step 2 was purified by flash column chromatography, eluting with 1:3 (ethyl acetate: hexanes) to provide the product **4l** as a white solid (34.6 mg, 62% yield over 2 steps). Spectroscopic data is consistent with previous reports.[5]

^{1}H NMR (500 MHz, $CDCl_3$) δ 8.0 – 8.0 (m, 2H), 7.5 – 7.5 (m, 2H), 6.0 (dd, J = 17.5, 11.2 Hz, 1H), 5.8 (dd, J = 17.5, 1.9 Hz, 1H), 5.6 (dd, J = 11.2, 2.0 Hz, 1H), 3.9 (s, 3H).

^{13}C NMR (125 MHz, $CDCl_3$) δ (ppm) 166.5, 131.4, 129.5, 129.4, 128.0, 127.8, 116.8, 90.9, 89.1, 52.2.

HRMS (ESI) calcd. for $C_{12}H_{11}O_2$ $[M+H]^+$: 187.0753, found: 187.0749.

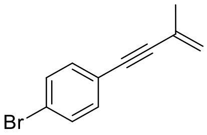

1-Bromo-4-(3-methylbut-3-en-1-yn-1-yl)benzene (4m):

Prepared following general procedure A using 1-bromo-4-(but-1-yn-1-yl)benzene (**1m**, 62.7 mg , 0.3 mmol, 1.0 equiv). The crude residue from step 2 was purified by flash column chromatography, eluting with pentane to provide the product **4m** as a colorless oil (66.3 mg, 55% yield over 2 steps).

^{1}H NMR (500 MHz, $CDCl_3$) δ 7.44 (d, J = 8.5 Hz, 2H), 7.3 (d, J = 8.5 Hz, 2H), 5.39-5.41 (m, 1H), 5.31-5.33 (m, 1H), 1.98 (s, 3H).

^{13}C NMR (125 MHz, $CDCl_3$) δ (ppm) 133.0, 131.5, 126.6, 122.4, 122.3, 122.2, 91.68, 87.3, 23.3.

HRMS (ESI) calcd. for $C_{11}H_{10}Br$ $[M+H]^+$: 220.9960, found: 220.9955.

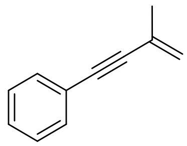

4n

(3-Methylbut-3-en-1-yn-1-yl) benzene (4n):

Prepared following general procedure A using but-1-yn-1-ylbenzene (**1n**, 43 µL, 0.3 mmol, 1.0 equiv). The crude residue from step 2 was purified by flash column chromatography, eluting with pentane to provide the product **4n** as a colorless oil (21.7 mg, 51% yield over 2 steps). Spectroscopic data is consistent with previous reports.[5]

^{1}H NMR (500 MHz, $CDCl_3$) δ (ppm) 7.46-7.43 (m, 2H), 7.33-7.30 (m, 3H), 5.4 (q, 1H), 5.30 (q, 1H), 2.01 (t, *J* = 1.3 Hz, 3H).

^{13}C NMR (125 MHz, $CDCl_3$) δ (ppm) 131.7, 128.4, 128.2, 127, 123.5, 122, 90.7, 88.5, 23.6.

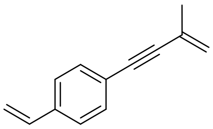

4o

1-(3-Methylbut-3-en-1-yn-1-yl)-4-vinylbenzene (4o):

Prepared following general procedure A using 1-(but-1-yn-1-yl)-4-vinylbenzene (**1o**, 46.8 mg, 0.3 mmol, 1.0 equiv) (55% NMR yield for step 1). The crude residue from Step 2 was purified by flash column chromatography, eluting with hexanes to provide the product **4o** as a resin (21.0 mg, 42% isolated yield over 2 steps).

^{1}H NMR (400 MHz, $CDCl_3$): δ (ppm) 7.40 (d, *J* = 8.3 Hz, 2H), 7.35 (d, *J* = 8.3 Hz, 2H), 6.69 (dd, *J* = 17.6 Hz, 10.9 Hz, 1H), 5.76 (d, *J* = 17.6 Hz, 1H), 5.4 (s, 1H), 5.28 (app. d, *J* = 11.1 Hz, 2H), 1.99 (s, 3H).

^{13}C NMR (100 MHz, $CDCl_3$): δ (ppm) 137.5, 136.4, 131.9, 127.0 126.3, 122.7, 122.1, 114.8, 91.4, 88.6, 23.6.

HRMS: (ESI) calcd. for $C_{13}H_{13}$ $[M+H]^{+}$: 169.1012, found: 169.1014.

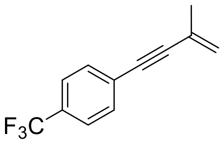

4p

1-(3-Methylbut-3-en-1-yn-1-yl)-4-(trifluoromethyl)benzene (4p):

Prepared following general procedure A using 1-(but-1-yn-1-yl)-4-(trifluoromethyl) benzene (**1p**, 59.4 mg, 0.3 mmol, 1.0 equiv) (55% NMR yield for step 1). The crude residue from Step 2 was purified by flash column chromatography, eluting with hexanes to provide the product **4p** as a white solid (23.8 mg, 38% isolated yield over 2 steps). Spectroscopic data is consistent with previous reports. [6]

^{1}H NMR (500 MHz, $CDCl_3$): δ (ppm) 7.57 (d, J = 8.4 Hz, 2H), 7.53 (d, J = 8.4 Hz, 2H), 5.45 (app. d, J = 0.6 Hz, 1H), 5.38-5.34 (m, 1H), 2.0 (s, 3H).

^{13}C NMR (125 MHz, $CDCl_3$): δ (ppm) 131.9, 130.0 (q, J_{CF} = 32.7 Hz), 127.3 (q, J_{CF} = 1.3 Hz), 126.6, 125.4 (q, J_{CF} = 3.8 Hz), 124.1 (q, J_{CF} = 272.1 Hz), 123.3, 93.1, 87.1, 23.4.

^{19}F NMR (471 MHz, $CDCl_3$): –62.8.

HRMS: (ESI) calcd. for $C_{12}H_{10}F_3$ $[M+H]^+$: 211.0729, found: 211.0730.

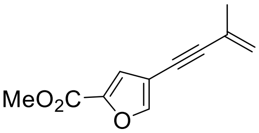

4q

Methyl 4-(3-methylbut-3-en-1-yn-1-yl)furan-2-carboxylate (4q):

Prepared following general procedure A using methyl 4-(but-1-yn-1-yl)furan-2-carboxylate (**1q**, 53.45 mg, 0.3 mmol, 1.0 equiv) (56% NMR yield for step 1). The crude residue from Step 2 was purified by flash column chromatography, eluting with 1:19 (ethyl acetate: hexanes) to provide the product **4q** as a yellow solid (21.4 mg, 38% isolated yield over 2 steps), **m.p.** 64.1–66.2 °C.

^{1}H NMR (500 MHz, $CDCl_3$): δ (ppm) 7.15 (d, J = 3.6 Hz, 1H), 6.61 (d, J = 3.6 Hz, 1H), 5.48 (app. s, 1H), 5.40-5.37 (m, 1H), 3.90 (s, 3H), 2.07-1.92 (m, 3H).

^{13}C NMR (125 MHz, $CDCl_3$): δ (ppm) 158.6, 144.5, 140.5, 125.7, 124.4, 119.0, 116.5, 96.4, 77.7, 52.2, 22.9.

HRMS: (ESI) calcd. for $C_{11}H_{11}O_3$ $[M+H]^+$: 191.0703, found: 191.0710.

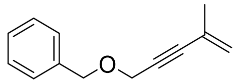

4r

(((4-Methylpent-4-en-2-yn-1-yl)oxy)methyl)benzene (4r):

Prepared following general procedure A ((pent-2-yn-1-yloxy)methyl)benzene (**1r**, 52.3 mg, 0.3 mmol, 1.0 equiv) (58% NMR yield for step 1). The crude residue from Step 2 was purified by flash column chromatography, eluting with 1:39 (ethyl acetate: hexanes) to provide the product **4r** as a yellow oil (30.8 mg, 55.1% isolated yield over 2 steps, > 20:1 r.r.).

^{1}H NMR (500 MHz, $CDCl_3$): δ (ppm) 7.40-7.28 (m, 5H), 5.34 (app. s, 1H), 5.27-5.24 (m, 1H), 4.62 (s, 2H), 4.30 (s, 2H), 1.92 (s, 3H).

^{13}C NMR (125 MHz, $CDCl_3$): δ (ppm) 137.7, 128.6, 128.2, 128.0, 126.5, 122.4, 87.8, 84.2, 71.7, 57.9, 23.5.

HRMS: (ESI) calcd. for $C_{13}H_{15}O$ $[M+H]^+$: 187.1117, found: 187.1125.

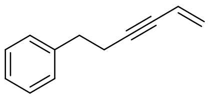

4s

Hex-5-en-3-yn-1-ylbenzene (4s):

Prepared following general procedure A using pent-3-yn-1-ylbenzene (**1s**, 43.3 mg, 0.3 mmol, 1.0 equiv). The crude residue from Step 2 was purified by flash column chromatography, eluting with pentane to provide the product **4s** as colorless oil (35.1 mg, 75% isolated yield over 2 steps, > 20:1 r.r). Spectroscopic data is consistent with previous reports. [5]

^{1}H NMR (500 MHz, $CDCl_3$) δ (ppm) 7.30 (dd, J = 10.0, 5.0 Hz, 2H), 7.2-7.24 (m, 3H), 5.77 (ddt, J = 17.5, 11.1, 2.1 Hz, 1H), 5.55 (dd, J = 17.5, 2.1 Hz 1H), 5.39 (dd, J = 11.1, 2.1 Hz, 1H), 2.86 (t, J = 7.6 Hz, 2H), 2.6 (td, J = 7.6, 1.9 Hz, 2H).

13C NMR (125 MHz, $CDCl_3$) δ (ppm) 141.1, 128.9, 128.9, 126.8, 126.3, 118, 90.7, 90.5, 35.6, 22.

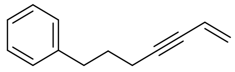

4t

Hept-6-en-4-yn-1-ylbenzene (4t):

Prepared following general procedure A using hex-4-yn-1-ylbenzene (**1t**, 47.4 mg, 0.3 mmol, 1.0 equiv). The crude residue from Step 2 was purified by flash column chromatography, eluting with pentane to provide the product **4t** as colorless oil (38.3 mg, 75% isolated yield over 2 steps, > 20:1 r.r). Spectroscopic data is consistent with previous reports. [7]

1H NMR (500 MHz, $CDCl_3$) δ (ppm) 7.18-7.31 (m, 5H), 5.78-5.84 (m, 1H), 5.55-5.61 (m, 1H), 5.38-5.42 (m, 1H), 2.74 (t, *J* =7.6 Hz, 2H), 2.33 (t, *J* =6.5 Hz, 2H), 1.83-19.1 (m, 2H).

13C NMR (125 MHz, $CDCl_3$) δ (ppm) 141.6, 128.5, 128.3, 125.9, 125.6, 117.6, 90.6, 79.8, 34.8, 30.2, 18.7.

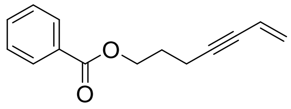

4u

Hept-6-en-4-yn-1-yl benzoate (4u):

Prepared following general procedure A using hex-4-yn-1-yl benzoate (**1u**, 60.6 mg, 0.3 mmol, 1.0 equiv). The crude residue from Step 2 was purified by flash column chromatography, eluting with 1:39 (ethyl acetate: hexanes) to provide the product **4r** as a white foam (30.2 mg, 47% isolated yield over 2 steps, > 20:1 r.r).

1H NMR (500 MHz, $CDCl_3$) δ (ppm) 8.02-8.08 (m, 2H), 7.53-7.57 (m, 1H), 7.44(t, *J* =7.7Hz, 2H), 5.76 (ddt, *J* = 17.5, 11.1, 2.1 Hz, 1H), 5.56 (dd, *J* = 17.5, 2.1 Hz 1H), 5.39 (dd, *J* = 11.1, 2.1 Hz, 1H), 4.43 (t, 6.3 Hz, 2H), 2.51 (td, *J* =7.0, 1.9 Hz, 2H), 2.01 (p, *J* =6.7 Hz, 2H).

13C NMR (125 MHz, $CDCl_3$) δ (ppm) 166.5, 132.9, 130.3, 129.6, 128.3, 125.9, 117.4, 89.4, 80, 63.7, 22.9, 16.3.

HRMS (ESI) calcd. for $C_{14}H_{15}O_2$ $[M+H]^+$: 215.1066, found: 215.1076.

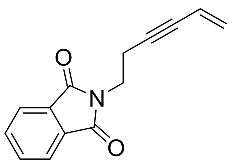

4v

2-(Hex-5-en-3-yn-1-yl)isoindoline-1,3-dione (4v):

Prepared following general procedure A using 2-(pent-3-yn-1-yl)isoindoline-1,3-dione (**1v**, 63.9 mg, 0.3 mmol, 1.0 equiv). The crude residue from Step 2 was purified by flash column chromatography, eluting with 1:39 (ethyl acetate: hexanes) to provide the product **4v** as a white solid (35.2 mg, 55% isolated yield over 2 steps, > 20:1 r.r). Spectroscopic data is consistent with previous reports. Spectroscopic data is consistent with previous reports. [8]

^{1}H NMR (500 MHz, $CDCl_3$) δ (ppm) 7.84-7.88 (m, 2H), 7.70-7.74 (m, 2H), 5.65-5.75 (m, 1H), 5.51 (dd, *J* = 17.5, 2.3 Hz, 1H), 5.37 (dd, *J* = 10.9, 2.3 Hz), 3.89 (t, *J* = 7.2 Hz, 2H), 2.72 (td, *J* =7.1, 1.7 Hz, 2H).

^{13}C NMR (125 MHz, $CDCl_3$) δ (ppm) 168.0, 134.0, 132.0, 126.5, 123.3, 117.0, 86.47, 81.09, 36.7, 19.3.

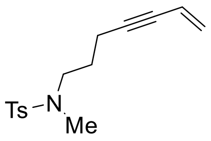

4w

***N*-(Hept-6-en-4-yn-1-yl)-*N*,4-dimethylbenzenesulfonamide (4w):**

Prepared following general procedure A using *N*-(hex-4-yn-1-yl)-*N*,4-dimethylbenzenesulfonamide (**1w**, 79.5 mg, 0.3 mmol, 1.0 equiv) (64% NMR yield for step 1). The crude residue from Step 2 was purified by flash column chromatography, eluting with 1:39 (ethyl acetate: hexanes) to provide the product **4w** as a white foam (41.6 mg, 50% isolated yield over 2 steps, > 20:1 r.r).

^{1}H NMR (500 MHz, $CDCl_3$) δ (ppm) 7.65-7.68 (m, 2H), 7.30-7.32 (m, 2H), 5.75 (ddt, *J* = 17.4, 11.1, 1.9 Hz, 1H), 5.55 (dd, *J* = 17.5, 1.8 Hz, 1H), 5.39 (dd, *J* = 11.1, 1.9 Hz, 1H), 3.08 (t, *J* = 7.1 Hz, 2H), 2.73 (s, 3H), 2.42 (s, 3H), 2.35-2.39 (m, 2H), 1.77 (p, *J* = 7.1 Hz, 2H).

^{13}**C NMR** (125 MHz, $CDCl_3$) δ (ppm) 143.3, 134.5, 129.6, 127.4, 125.9, 117.3, 89.5, 80.0, 49.3, 35.0, 27.0, 21.51, 16.65.

HRMS (ESI) calcd. for $C_{15}H_{20}O_2NS$ $[M+H]^+$: 278.1209, found: 278.1212.

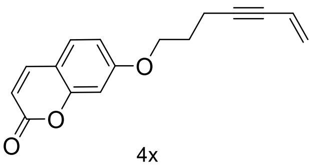

4x

7-(Hept-6-en-4-yn-1-yloxy)-2*H*-chromen-2-one (4x):

Prepared following general procedure A using 7-(hex-4-yn-1-yloxy)-2*H*-chromen-2-one (**1x**, 72.6 mg, 0.3 mmol, 1.0 equiv) (66% NMR yield for step 1). The crude residue from Step 2 was purified by flash column chromatography, eluting with 1:39 (ethyl acetate: hexanes) to provide the product **4x** as a colorless oil (34.3 mg, 45% isolated yield over 2 steps, > 20:1 r.r).

^{1}**H NMR** (500 MHz, $CDCl_3$) δ (ppm) 7.63 (d, *J* = 9.5 Hz, 1H), 7.36 (d, *J* = 8.4 Hz, 1H), 6.81-6.86 (m, 2H), 6.25 (d, *J* = 9.5 Hz, 1H), 5.77 (ddt, *J* = 17.5, 11.1, 2.0 Hz, 1H), 5.56 (dd, *J* = 17.5, 2.0 Hz, 1H), 5.4 (dd, *J* = 11.1, 2.0 Hz, 1H), 4.09-4.15 (m, 2H), 2.52-2.56 (m, 2H), 2.01-2.07 (m, 2H).

^{13}**C NMR** (125 MHz, $CDCl_3$) δ (ppm) 162.1, 161.2, 155.9, 143.4, 128.7, 126.1, 117.3, 113.1, 112.9, 112.5, 101.4, 89.2, 80.2, 66.9, 28.0, 16.0.

HRMS (ESI) calcd for $C_{16}H_{15}O_3$ $[M+H]^+$: 255.1015, found: 255.1017.

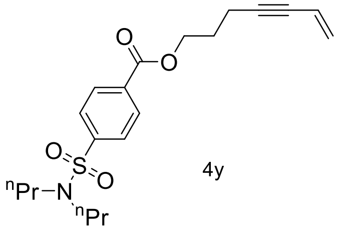

4y

Hept-6-en-4-yn-1-yl 4-(*N*,*N*-dipropylsulfamoyl)benzoate (4y):

Prepared following general procedure A using hex-4-yn-1-yl 4-(*N*,*N*-dipropylsulfamoyl)benzoate (**1y**, 109.5 mg, 0.3 mmol, 1.0 equiv). The crude residue from Step 2 was purified by flash column chromatography, eluting with 1:39 (ethyl acetate: hexanes) to provide the product **4y** as a colorless oil (48.6 mg, 43% isolated yield over 2 steps, > 20:1 r.r).

^{1}**H NMR** (500 MHz, $CDCl_3$) δ (ppm) 8.14-8.16 (m, 2H), 7.85-7.88 (m, 2H), 5.75 (ddt, *J* =

17.5, 11.0, 2.1 Hz, 1H), 5.55 (dd, J = 17.5, 2.1 Hz, 1H), 5.39 (dd, J = 11.1, 2.1 Hz, 1H), 4.46 (t, J = 6.3 Hz, 2H), 3.07-3.11 (m, 4H), 2.51 (td, J = 6.9, 1.9 Hz, 2H), 2.02 (p, J = 6.6 Hz, 2H), 1.50-1.58 (m, 4H), 0.86 (t, 7.4 Hz, 6H).

^{13}C NMR (125 MHz, $CDCl_3$) δ (ppm) 165.2, 144.3, 133.5, 130.2, 127.0, 126.1, 117.2, 89.1, 80.2, 64.38, 49.9, 27.7, 21.9, 16.3, 11.1.

HRMS (ESI) calcd. for $C_{20}H_{28}O_4NS$ $[M+H]^+$: 378.1733, found: 378.1735.

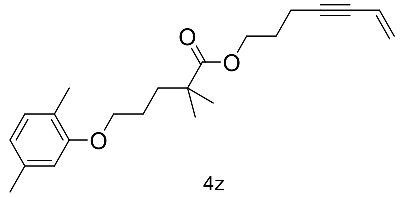

4z

Hept-6-en-4-yn-1-yl 5-(2,5-dimethylphenoxy)-2,2-dimethylpentanoate (4z):

Prepared following general procedure A using hex-4-yn-1-yl 5-(2,5-dimethylphenoxy)-2,2-dimethylpentanoate (**1z**, 99.1 mg, 0.3 mmol, 1.0 equiv). The crude residue from Step 2 was purified by flash column chromatography, eluting with 1:39 (ethyl acetate: hexanes) to provide the product **4z** as a yellow oil (49.2 mg, 48% isolated yield over 2 steps, > 20:1 r.r).

^{1}H NMR (500 MHz, $CDCl_3$) δ (ppm) 7.01 (d, J = 7.5 Hz, 1H), 6.66 (d, J = 7.5 Hz, 1H), 6.61 (s, 1H), 5.73-5.8 (m, 1H), 5.53-5.59 (m, 1H), 5.37-5.41 (m, 1H), 4.17 (t, J = 6.3 Hz, 2H), 3.91-3.94 (m, 2H), 2.39-2.44 (m, 2H), 2.31 (s, 3H), 2.19 (s, 3H), 1.87 (p, J = 6.7 Hz, 2H), 1.72-1.76 (m, 4H), 1.23 (s, 6H).

^{13}C NMR (125 MHz, $CDCl_3$) δ (ppm) 177.7, 156.9, 136.4, 130.3, 125.9, 123.6, 120.71, 117.4, 111.9, 89.3, 80.0, 67.9, 63.1, 42.1, 37.1, 27.7, 25.2, 25.1, 21.4, 16.1, 15.7.

HRMS (ESI) calcd. for $C_{22}H_{31}O_3$ $[M+H]^+$: 343.2267, found: 343.2260.

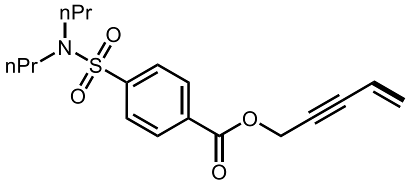

4za

Pent-4-en-2-yn-1-yl 4-(*N*,*N*-dipropylsulfamoyl)benzoate (4za):

Prepared following general procedure A using but-2-yn-1-yl 4-(*N*,*N*-dipropylsulfamoyl)benzoate (**1za**, 50.6 mg, 0.15 mmol, 1.0 equiv) (51% NMR yield for step 1). The crude residue from Step 2 was purified by flash column chromatography, eluting with

1:19 (ethyl acetate: hexanes) to provide the product **4za** as a white solid (13.1 mg, 25% isolated yield over 2 steps, > 20:1 r.r), **m.p.**: 56.9–60.1 °C.

^{1}H NMR (500 MHz, $CDCl_3$): δ (ppm) 8.19 (d, *J* = 8.4 Hz, 2H), 7.88 (d, *J* = 8.4 Hz, 2H), 5.83 (ddt, *J* = 17.5 Hz, 11.0 Hz, 1.8 Hz, 1H), 5.72 (dd, *J* = 17.6 Hz, 2.0 Hz, 1H), 5.56 (dd, *J* = 11.0 Hz, 2.1 Hz, 1H) 5.07 (d, *J* = 1.6 Hz, 2H), 3.16-3.04 (m, 4H), 1.61-1.48 (m, 4H), 0.87 (t, *J* = 7.4 Hz, 6H).

^{13}C NMR (125 MHz, $CDCl_3$): δ (ppm) 164.8, 144.8, 133.0, 130.6, 128.6, 127.2, 116.4, 85.8, 83.2, 53.9, 50.0, 22.0, 11.3.

HRMS: (ESI) calcd. for $C_{18}H_{24}O_4NS$ $[M+H]^+$: 350.1421, found: 350.1423.

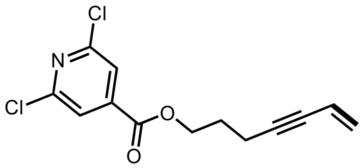

4zb

Hept-6-en-4-yn-1-yl 2,6-dichloroisonicotinate (4zb):

Prepared following general procedure A using hex-4-yn-1-yl 2,6-dichloroisonicotinate (**1zb**, 40.8 mg, 0.15 mmol, 1.0 equiv) (75% NMR yield for step 1). The crude residue from Step 2 was purified by flash column chromatography, eluting with 1:19 (ethyl acetate:hexanes) to provide the product **4zb** as a resin (19.6 mg, 46 % isolated yield over 2 steps, > 20:1 r.r.).

^{1}H NMR (500 MHz, $CDCl_3$): δ (ppm) 7.80 (s, 2H), 5.74 (ddt, *J* = 17.5 Hz, 11.0 Hz, 2.1 Hz, 1H), 5.55 (dd, *J* = 17.5 Hz, 2.0 Hz, 1H) 5.40 (dd, *J* = 11.1 Hz, 2.1 Hz, 1H) 4.48 (t, *J* = 6.3 Hz, 2H), 2.5 (td, *J* = 6.8 Hz, 1.9 Hz, 2H) 2.02 (p, *J* = 6.6 Hz, 2H).

^{13}C NMR (125 MHz, $CDCl_3$): δ (ppm) 162.8, 151.6, 142.7, 126.4, 122.7, 117.3, 88.9, 80.5, 65.6, 27.6, 16.4.

HRMS: (ESI) calcd. for $C_{13}H_{12}O_2NCl_2$ $[M+H]^+$: 284.0240, found: 284.0238.

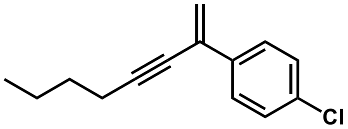

4zc

1-Chloro-4-(oct-1-en-3-yn-2-yl)benzene (4zc)

Prepared following a variation of general procedure A using 1-chloro-4-(hept-2-yn-1-yl) benzene (**1zc**, 62.0 mg, 0.3 mmol, 1 equiv) with the following modifications. In Step 1, $Mg(NTf_2)_2$ (87.7 mg, 0.15 mmol, 0.50 equiv) and TMPH (5.0 equiv) was used. After the sealed reaction vial was removed from the glovebox, the reaction mixture was stirred at 70 °C for 24 h (92% NMR yield for step 1) In Step 2, a solution of *m*-chloroperbenzoic acid (78 mg, 0.45 mmol, 1.5 equiv) in THF (5 mL) was added to a flask containing the crude tertiary amine in THF (2 mL) at 0 °C dropwise over 2 min. Subsequently, the flask was kept at 0 °C for 5 min. The solution was then quenched immediately with saturated aqueous sodium bicarbonate while still at 0 °C, then warmed to r.t. and extracted with ethyl acetate (3 × 10 mL). The organic phase was dried over Na_2SO_4 and concentrated. After the removal of the solvent, the crude residue was purified by flash column chromatography eluting with hexanes to afford the title compound as a pale-yellow oil (47.4 mg, 72% yield over 2 steps, > 20:1 r.r).

^{1}H NMR (500 MHz, $CDCl_3$): δ (ppm) 7.61-7.53 (m, 2H), 7.33-7.27 (m, 2H), 5.81 (app. s, 1H), 5.58 (app. s, 1H), 2.41 (t, *J* = 7.1 Hz, 2H), 1.63-1.56 (m, 2H), 1.52-1.43 (m, 2H), 0.95 (t, *J* = 7.3 Hz, 3H).

^{13}C NMR (125 MHz, $CDCl_3$): δ (ppm) 136.5, 134.1, 130.1, 128.5, 127.5, 119.8, 92.6, 79.5, 30.9, 22.2, 19.2, 13.8.

HRMS: (ESI) calcd. for $C_{14}H_{16}Cl$ $[M+H]^+$: 219.0935, found: 219.0936.

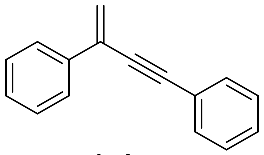

4zd

But-3-en-1-yne-1,3-diyldibenzene (4zd)

Prepared following a variation of general procedure A using prop-1-yne-1,3-diyldibenzene (**1zd**, 57.7 mg, 0.3 mmol, 1.0 equiv) with the following modifications. In Step 1, TMPH (5.0 equiv) was used. After the sealed reaction vial was removed from the glovebox, the reaction mixture was stirred at 70 °C for 24 h (88% NMR yield for step 1). In Step 2, a solution of *m*-chloroperbenzoic acid (78 mg, 0.45 mmol, 1.5 equiv) in THF (5 mL) was added to a flask containing the crude tertiary amine in THF (2 mL) at 0 °C dropwise over 2 min. Subsequently, the flask was kept at 0 °C for 10 min. The solution was then quenched immediately with saturated aqueous sodium bicarbonate while still at 0 °C, then warmed to r.t. and extracted with ethyl acetate (3 × 10 mL). The organic phase was dried over Na_2SO_4 and concentrated. After the removal of the solvent, the crude residue was purified by flash column chromatography eluting with hexanes to provide the product **4zd** as a yellow oil (36.0 mg, 59% isolated yield over 2 steps, > 20:1 r.r).

^{1}H NMR (500 MHz, $CDCl_3$): δ (ppm) 7.74 (d, *J* = 7.8 Hz, 2H), 7.58-7.52 (m, 6H), 7.43-7.32 (m, 2H), 6.00 (s, 1H), 5.77 (s, 1H).

^{13}C NMR (125 MHz, $CDCl_3$): δ (ppm) 137.4, 131.8, 130.8, 128.6, 128.5, 126.3, 123.4, 120.8, 90.9, 88.7.

HRMS: (ESI) calcd. for $C_{16}H_{13}$ $[M+H]^+$: 205.1012, found: 205.1026.

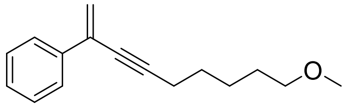

4ze

(9-Methoxynon-1-en-3-yn-2-yl)benzene (4ze)

Prepared following a variation of general procedure A using (8-methoxyoct-2-yn-1-yl)benzene (**1ze**, 64.8 mg, 0.3 mmol, 1.0 equiv) with the following modifications. In Step 1, TMPH (5.0 equiv) was used. After the sealed reaction vial was removed from the glovebox, the reaction mixture was stirred at 70 °C for 24 h (84% NMR yield for step 1). In Step 2, a solution of *m*-chloroperbenzoic acid (78 mg, 0.45 mmol, 1.5 equiv) in THF (5 mL) was added to a flask containing the crude tertiary amine in THF (2 mL) at 0 °C dropwise over 2 min. Subsequently, the flask was kept at 0 °C for 10 min. The solution was then quenched immediately with saturated aqueous sodium bicarbonate while still at 0 °C, then warmed to r.t. and extracted with ethyl acetate (3 × 10 mL). The organic phase was dried over Na_2SO_4 and concentrated. After the removal of the solvent, the crude residue was purified by flash column chromatography eluting with 1:19 (ethyl acetate: hexanes) to provide the product **4ze** as a pale yellow oil (38.0 mg, 56% isolated yield over 2 steps, > 20:1 r.r).

^{1}H NMR (500 MHz, $CDCl_3$): δ (ppm) 7.67-7.63 (m, 2H), 7.37-7.32 (m, 2H), 7.31-7.27 (m, 1H), 5.84 (d, *J* = 1.0 Hz, 1H), 5.58 (app. s, 1H), 3.39 (t, *J* = 6.5 Hz, 2H), 3.34 (s, 3H), 2.43 (t, *J* = 7.1 Hz, 2H), 1.68-1.59 (m, 4H), 1.55-1.48 (m, 2H).

^{13}C NMR (125 MHz, $CDCl_3$): δ (ppm) 137.9, 131.1, 128.4, 128.2, 126.2, 119.6, 91.9, 80.0, 72.8, 58.7, 29.3, 28.7, 25.7, 19.4.

HRMS: (ESI) calcd. for $C_{16}H_{21}O$ $[M+H]^+$: 229.1587, found: 229.1586.

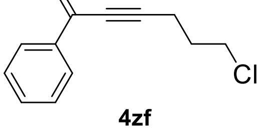

4zf

(7-Chlorohept-1-en-3-yn-2-yl)benzene (4zf)

Prepared following a variation of general procedure A using (6-chlorohex-2-yn-1-yl)benzene (**1zf**, 57.8 mg, 0.3 mmol, 1.0 equiv) with the following modifications. In Step 1, TMPH (5.0 equiv) was used. After the sealed reaction vial was removed from the glovebox, the reaction mixture was stirred at 70 °C for 24 h (82% NMR yield for step 1). In Step 2, a solution of *m*-chloroperbenzoic acid (78 mg, 0.45 mmol, 1.5 equiv) in THF (5 mL) was added to a flask containing the crude tertiary amine in THF (2 mL) at 0 °C dropwise over 2 min. Subsequently, the flask was kept at 0 °C for 10 min. The solution was then quenched immediately with saturated aqueous sodium bicarbonate while still at 0 °C, then warmed to r.t. and extracted with ethyl acetate (3 × 10 mL). The organic phase was dried over Na_2SO_4 and concentrated. After the removal of the solvent, the crude residue was purified by flash column chromatography eluting with hexanes to provide the product **4zf** as a pale yellow oil (36.0 mg, 59% isolated yield over 2 steps, > 20:1 r.r).

^{1}H NMR (500 MHz, $CDCl_3$): δ (ppm) 7.65-7.61 (m, 2H), 7.38-7.33 (m, 2H), 7.33-7.28 (m, 1H), 5.86 (d, *J* = 0.8 Hz, 1H), 5.60 (app. s, 1H), 3.71 (t, *J* = 6.4 Hz, 2H), 2.62 (t, *J* = 6.8 Hz, 2H), 2.07 (p, *J* = 6.6 Hz, 2H).

^{13}C NMR (125 MHz, $CDCl_3$): δ (ppm) 137.7, 130.8, 128.5, 128.4, 126.2, 120.1, 89.8, 80.9, 43.9, 31.6, 17.0.

HRMS: (ESI) calcd. for $C_{13}H_{14}Cl$ $[M+H]^+$: 205.0779, found: 205.0793.

Synthesis of substrates and characterization data for new compounds:

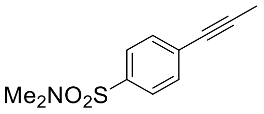

1f

***N*,*N*-Dimethyl-4-(prop-1-yn-1-yl)benzenesulfonamide (1f):**

Prepared following general procedure B using 4-bromo-*N*,*N*-dimethylbenzenesulfonamide (1.32 g, 5 mmol, 1.0 equiv) and 1,8-Diazabicyclo(5.4. 0)undec-7-ene (2.28 g, 15 mmol, 3.0 equiv). The crude mixture was purified by flash column chromatography by eluting with 1:30 (ethyl acetate: hexanes) to afford the title compound as a white solid (75% yield, 0.837 g), **m.p.** 90.5–92.8 °C.

^{1}H NMR (500 MHz, $CDCl_3$): δ (ppm) 7.66-7.69 (m, 2H), 7.50-7.53 (m, 2H), 2.69(s, 6H), 2.08 (s, 3H)
^{13}C NMR (125 MHz, $CDCl_3$): δ (ppm) 134.1, 131.9, 128.9, 127.6, 90.2, 78.5, 37.9, 4.4.
HRMS: (ESI) calcd. for C_{11}H14O_2NS $[M+H]^+$: 224.0739, found: 224.0739.

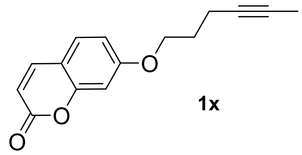

1x

7-(Hex-4-yn-1-yloxy)-2*H*-chromen-2-one (1x)

6-Chlorohex-2-yne (643 mg, 5.5 mmol, 1.1 equiv), 7-hydroxy-2*H*-chromen-2-one (811 mg, 5.0 mmol, 1.0 equiv), cesium carbonate (2.44 g, 7.5 mmol, 1.5 equiv), and potassium iodide (83 mg, 0.5 mmol, 0.05 equiv) were added to MeCN (10 mL) in a 20 mL pressure tube equipped with a stir bar. After sealing with a Teflon stopper, the reaction mixture was stirred at 85 °C for 18 h. After cooling and unsealing the pressure tube, the reaction mixture was filtered through a plug of Celite and concentrated. The crude residue was purified by flash column chromatography, eluting with 10:1 (hexanes:ethyl acetate) to give a slightly impure solid, which was the further purified by recrystallization from hexanes/dichloromethane to give the pure product as a white solid (500 mg, 41% yield), **m.p.**: 98.7–100.4 °C.

^{1}H NMR (500 MHz, $CDCl_3$): 7.63 (d, J = 9.5 Hz, 1H), 7.39-7.34 (m, 1H), 6.87-6.81 (m, 2H), 6.25 (d, J = 9.5 Hz, 1H), 4.12 (t, J = 6.2 Hz, 2H), 2.39-2.30 (m, 2H), 1.98 (p, J = 6.5 Hz, 2H), 1.78 (t, J = 2.5 Hz, 3H).

^{13}C NMR (125 MHz, $CDCl_3$): 162.4, 161.4, 156.1, 143.6, 128.8, 113.2, 113.2, 112.6, 101.6, 77.8, 76.7, 67.2, 28.5, 15.5, 3.6.

HRMS: (ESI) calcd. for $C_{15}H_{15}O_3$ $[M+H]^+$: 243.1016, found: 243.1016.

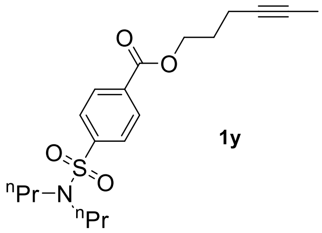

1y

Hex-4-yn-1-yl 4-(*N*,*N*-dipropylsulfamoyl)benzoate (1y)

Prepared from general procedure C using hex-4-yn-1-ol (491 mg, 5.0 mmol, 1.0 equiv) and 2,6-dichloroisonicotinic acid (1.43 g, 5.0 mmol, 1.0 equiv). The crude material purified by flash

column chromatography, eluting with 20:1 (hexanes:ethyl acetate) to give the product as a colorless oil (959 mg, 52% yield).

^{1}H NMR (500 MHz, $CDCl_3$): 8.15 (d, J = 8.4 Hz, 2H), 7.87 (d, J = 8.4 Hz, 2H), 4.45 (t, J = 6.3 Hz, 2H), 3.14-3.06 (m, 4H), 2.33 (tq, J = 6.9 Hz, 2.4 Hz, 2H) 1.96 (p, J = 6.6 Hz, 2H), 1.76 (t, J = 6.6 Hz, 3H), 1.59-1.50 (m, 4H,), 0.87 (t, J = 7.4 Hz, 6H).

^{13}C NMR (125 MHz, $CDCl_3$): 165.4, 144.4, 133.8, 130.6, 127.1, 77.7, 76.7, 64.6, 50.1, 28.2, 22.1, 15.8, 11.3, 3.6.

HRMS: (ESI) calcd. for $C_{19}H_{28}O_4NS$ $[M+H]^+$: 366.1734, found: 366.1733.

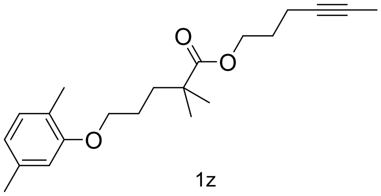

1z

Hex-4-yn-1-yl 5-(2,5-dimethylphenoxy)-2,2-dimethylpentanoate (1z):

To a suspension of 5-(2,5-dimethylphenoxy)-2,2-dimethylpentanoic acid (1.25g, 5 mmol, 1 equiv), K_2CO_3 (1.38g, 10 mmol, 2 equiv), KI (83 mg, 0.5 mmol, 0.1 equiv) in MeCN was added 6-bromohex-2-yne (0.88 g, 5.5 mmol, 1.1 equiv) at r.t. The reaction mixture was allowed to stir at 85 °C for 4 hr. After completion, the reaction mixture was quenched with water, extracted by ethyl acetate (3 × 10 mL), and the combined organic layers were washed with brine (10 mL). The organic phase was dried over Na_2SO_4 and concentrated. After the removal of the solvent, the residue was purified by flash column chromatography eluting with 50:1 (hexanes:ethyl acetate) to afford the title compound **1z** as a colorless oil. (55% yield, 0.908g).

^{1}H NMR (500 MHz, $CDCl_3$): δ (ppm) 7.00 (d, J = 7.5 Hz, 1H), 6.65 (d, J = 7.5 Hz, 1H), 6.61 (s, 1H),4.15 (t, J = 6.3 Hz, 2H), 3.94-3.89 (m, 2H), 2.30 (s, 3H), 2.22 (tq, J = 7.1, 2.5 Hz, 2H), 2.17 (s, 3H), 1.80 (p, J = 6.7 Hz, 2H), 1.76 (t, J = 2.5 Hz, 3H), 1.75-1.69 (m, 4H), 1.22 (s, 6H).

^{13}C NMR (125 MHz, $CDCl_3$): δ (ppm) 177.7, 156.9, 136.4, 130.3, 120.7, 111.9, 77.6, 76.2, 67.9, 63.2, 42.1, 37.1, 29.7, 28.1, 25.2, 25.1, 21.4, 15.7, 15.5, 3.4.

HRMS: (ESI) calcd. for $C_{21}H_{31}O_3$ $[M+H]^+$: 331.2267, found: 331.2268.

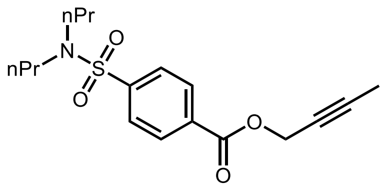

1za

But-2-yn-1-yl 4-(*N*,*N*-dipropylsulfamoyl)benzoate (1za)

Prepared from general procedure C using but-2-yn-1-ol (3.7 mL, 5 mmol, 1.0 equiv) and 4-(*N*,*N*-dipropylsulfamoyl)benzoic acid (1.43 g, 5 mmol, 1.0 equiv). The crude material purified by flash column chromatography, eluting with 3:1 (hexanes:ethyl acetate) containing 1% triethylamine to give the product as a white solid (926 mg, 55% yield), **m.p.** 67.6–69.1 °C.

^{1}H NMR (500 MHz, $CDCl_3$): 8.19 (d, *J* = 8.6 Hz, 2H), 7.88, (d, *J* = 8.6 Hz, 2H), 4.92 (q, *J* = 2.4 Hz, 2H), 3.12-3.07 (m, 4H), 1.89 (t, *J* = 2.4 Hz, 3H), 1.60-1.49 (m, 4H), 0.87 (t, *J* = 7.4 Hz, 6H).

^{13}C NMR (125 MHz, $CDCl_3$): 164.9, 144.4, 133.2, 130.6, 127.2, 84.0, 72.9, 54.0, 50.0, 22.1, 11.3, 3.8.

HRMS: (ESI) calcd. for $C_{17}H_{24}O_4NS$ $[M+H]^+$: 338.1421, found: 338.1419.

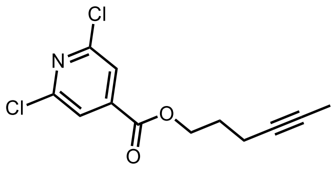

1zb

Pent-3-yn-1-yl 2,6-dichloroisonicotinate (1zb)

Prepared from general procedure C using hex-4-yn-1-ol (491 mg, 5.0 mmol, 1.0 equiv) and 2,6-dichloroisonicotinic acid (960 mg, 5.0 mmol, 1.0 equiv). The crude material purified by flash column chromatography, eluting with 100:1 (hexanes:ethyl acetate) to give the product as a white solid (1.29 g, 95% yield), **m.p.**: 34.4–36.4 °C.

^{1}H NMR (500 MHz, $CDCl_3$): 7.81 (s, 2H), 4.47 (t, *J* = 6.3 Hz, 2H), 2.37-2.26 (m, 2H), 1.95 (p, *J* = 6.6 Hz, 2H), 1.77 (t, *J* = 2.5 Hz, 3H).

^{13}C NMR (125 MHz, $CDCl_3$): 162.9, 151.7, 142.8, 122.8, 77.4 ,77.0, 65.7, 27.9, 15.8, 3.6.

HRMS: (ESI) calcd. for $C_{12}H_{12}O_2NCl_2$ $[M+H]^+$: 272.0240, found: 272.0240.

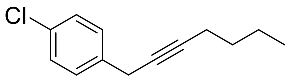

1zc

1-Chloro-4-(hept-2-yn-1-yl) benzene (1zc):

To a septum-sealed 100 mL round-bottom flask containing a stirred solution of iPrMgCl•LiCl complex (1.3 M in THF, 30 mL, 40 mmol, 4.0 equiv) under N_2 at 0 °C was added 1-hexyne (4.7 mL, 41 mmol, 4.1 equiv) dropwise. The solution was warmed to r.t. and stirred for 30 min. Then, CuI (190 mg, 1 mmol, 10 mol %) was added, and the reaction mixture was stirred for another 30 min. Thereafter, 4-chlorobenzyl bromide (2.10 g, 10 mmol, 1.0 equiv) was added and the flask was placed in an oil bath preheated to 60 °C and stirred for another 12 h. The reaction mixture was then cooled to r.t., quenched with saturated aqueous ammonium chloride (10 mL), and extracted with ethyl acetate (3 × 10 mL). The organic phase was dried over Na_2SO_4 and concentrated. After the removal of the solvent, the residue was purified by flash column chromatography eluting with hexanes to afford the title compound as a pale-yellow oil (946 mg, 47% yield).

^{1}H NMR (500 MHz, $CDCl_3$): δ (ppm) 7.27 (app. s, 4H), 3.54 (t, *J* = 2.4 Hz, 2H), 2.22 (tt, *J* = 7.1 Hz, 2.4 Hz, 2H), 1.55-1.47 (m, 2H), 1.47-1.38 (m, 2H), 0.92 (t, *J* = 7.3 Hz, 3H).

^{13}C NMR (125 MHz, $CDCl_3$): δ (ppm) 136.3, 132.3, 129.3, 128.6, 83.3, 77.1, 31.2, 24.8, 22.1, 18.6, 13.0.

HRMS: (ESI) calcd. for $C_{13}H_{14}Cl$ $[M+H]^+$: 205.0779, found: 205.0776.

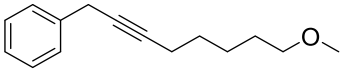

1ze

(8-Methoxyoct-2-yn-1-yl)benzene (1ze)

To a septum-sealed 100 mL round-bottom flask containing a stirred solution of iPrMgCl•LiCl complex (1.3 M in THF, 21.5 mL, 28 mmol, 2.8 equiv) under N_2 at 0 °C was added 7-methoxyhept-1-yne (3.53 g, 28 mmol, 2.8 equiv) dropwise. The solution was warmed to r.t. and stirred for 30 min. Then, CuI (190 mg, 1 mmol, 10 mol %) was added, and the reaction mixture was stirred for another 30 min. Thereafter, benzyl bromide (1.2 mL, 10 mmol, 1.0 equiv) was added and the flask was placed in an oil bath preheated to 60 °C and stirred for another 12 h. The reaction mixture was then cooled to r.t., quenched with saturated aqueous ammonium

chloride, and extracted with ethyl acetate (3 × 10 mL). The organic phase was dried over Na_2SO_4 and concentrated. After the removal of the solvent, the residue was purified by flash column chromatography eluting with 19:1 (hexanes: ethyl acetate) to afford product **1ze** as a yellow oil (1.43 g, 66% yield).

^{1}H NMR (500 MHz, $CDCl_3$): δ (ppm) 7.38-7.29 (m, 4H), 7.22 (t, J = 7.1 Hz, 1H), 3.58 (app. t, 2H), 3.38 (t, J = 6.6 Hz, 2H), 3.33 (s, 3H), 2.24 (tt, J = 7.1, 2.4 Hz, 2H), 1.64-1.52 (m, 4H), 1.51-1.43 (m, 2H)

^{13}C NMR (125 MHz, $CDCl_3$): δ (ppm) 137.7, 128.5, 128.0, 126.5, 82.6, 77.8, 72.9, 58.7, 29.3, 29.0, 25.6, 25.3, 19.0

HRMS: (ESI) calcd. for $C_{15}H_{20}ONa$ $[M+Na]^+$: 239.1406, found: 239.1445.

Large scale synthesis and synthetic applications of products:

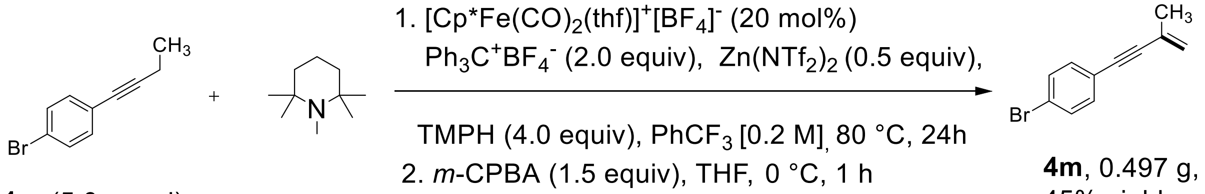

1m (5.0 mmol) **2** **4m**, 0.497 g, 45% yield

Large scale synthesis of 1-bromo-4-(3-methylbut-3-en-1-yn-1-yl)benzene (4m):

A round bottomed flask equipped with a magnetic stir bar was flame dried under vacuum. The flask was cooled under nitrogen and transferred into an argon-filled glovebox. In the glovebox, the suspension of 1,2,2,6,6-pentamethylpiperidine (**2**, 1.55 g, 10 mmol, 2.0 equiv) and $Ph_3C^+BF_4^-$ (3.3 g, 10 mmol, 2.0 equiv) in dry trifluorotoluene (16 mL) was stirred at room temperature for 1 hour to generate the iminium salt. Then $[Cp^*Fe(CO)_2(thf)]^+[BF_4]^-$ (0.41 g, 1 mmol, 20 mol%), $Zn(NTf_2)_2$ (1.81 g, 2.9 mmol, 0.58 equiv), trifluorotoluene (8 mL), alkyne 1-bromo-4-(but-1-yn-1-yl)benzene **1m** (1.04 g, 5 mmol, 1.0 equiv), and 2,2,6,6-tetramethylpiperidine (3.41 mL, 20 mmol, 4.0 equiv) were added in rapid succession. The reaction tube was capped and removed from the glovebox. The reaction tube was placed in an

oil bath, preheated to 80 °C, whereit was stirred for 24 h. After completion of the reaction, the reaction mixture was cooled to room temperature. The crude mixture was concentrated in vacuo, subjected to a short silica plug eluting with hexanes to remove any residual alkyne and then 1:1 (ethyl acetate: hexanes) to provide the homopropargylic amine product **3m** and moved forward to Step 2 without further purification.

To a flame dried round bottom flask under nitrogen was added a solution of crude **3m** in dry tetrahydrofuran (30 mL) and cooled to 0 °C . A solution of *meta*-chloroperbenzoic acid (1.29 g, 7.5 mmol, 1.5 equiv) in dry tetrahydrofuran (30 mL) was added to the flask at 0 °C dropwise for over 15 minutes. Subsequently, the reaction flask was kept at 0 °C for 1 h. After completion of the reaction, as determined by TLC analysis, the crude was concentrated in vacuo and purified by flash column chromatography to provide the desired compound **4m** as a colorless oil (45% yield over 2 steps, 0.497 g).

^{1}H NMR (500 MHz, $CDCl_3$) δ 7.44 (d, *J* = 8.5 Hz, 2H), 7.3 (d, *J* = 8.5 Hz, 2H), 5.39-5.41 (m, 1H), 5.31-5.33 (m, 1H), 1.98 (s, 3H).

^{13}C NMR (125 MHz, $CDCl_3$) δ (ppm) 133.0, 131.5, 126.6, 122.4, 122.3, 122.2, 91.68, 87.3, 23.3.

HRMS (ESI) calcd. for $C_{11}H_{10}Br$ $[M+H]^+$: 220.9960, found: 220.9955.

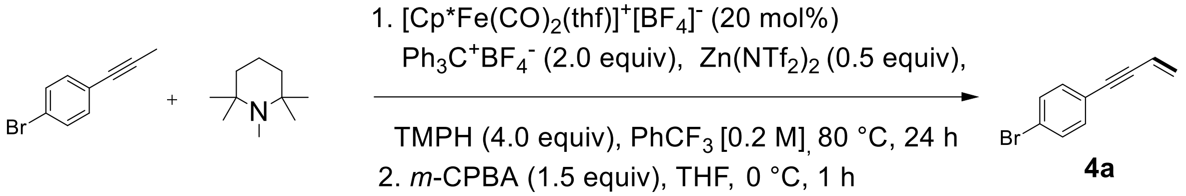

1a (1.0 mmol) **2** **4a**

4a, 0.155 g, 75% yield

Large scale synthesis of 1-bromo-4-(but-3-en-1-yn-1-yl)benzene (4a):

A round bottomed flask equipped with a magnetic stir bar was flame dried under vacuum. The flask was cooled under nitrogen and transferred into an argon-filled glovebox. In the glovebox, the suspension of 1,2,2,6,6-pentamethylpiperidine (**2**, 0.31 g, 2 mmol, 2.0 equiv) and $Ph_3C^+BF_4^-$ (0.66 g, 2 mmol, 2.0 equiv) in dry trifluorotoluene (3 mL) was stirred at room temperature for 1 hour to generate the iminium salt. Then $[Cp^*Fe(CO)_2(thf)]^+[BF_4]^-$ (82 mg, 0.2 mmol, 20 mol%), $Zn(NTf_2)_2$ (0.362 g, 0.58 mmol, 0.58 equiv), trifluorotoluene (1.5 mL), alkyne 1-bromo-4-(prop-1-yn-1-yl)benzene **1a** (0.195 g, 5 mmol, 1.0 equiv), and 2,2,6,6-tetramethylpiperidine (0.68 mL, 4 mmol, 4.0 equiv) were added in rapid succession. The reaction tube was capped and removed from the glovebox. The reaction tube was placed in an oil bath, preheated to 80 °C, where it was stirred for 24 h. After completion of the reaction, the reaction mixture was cooled to room temperature. The crude mixture was concentrated in

vacuo, subjected to a short silica plug eluting with hexanes to remove any residual alkyne and then 1:1 (ethyl acetate: hexanes) to provide the homopropargylic amine product **3a** and moved forward to Step 2 without further purification.

To a flame dried round bottom flask under nitrogen was added a solution of crude **3a** in dry tetrahydrofuran (6 mL) and cooled to 0 °C . A solution of *meta*-chloroperbenzoic acid (0.172 g, 1.5 mmol,1.5 equiv) in dry tetrahydrofuran (6 mL) was added to the flask at 0 °C dropwise for over 10 minutes. Subsequently, the reaction flask was kept at 0 °C for 1 hr. After completion of the reaction, as determined by TLC analysis, the crude was concentrated in vacuo and purified by flash column chromatography to provide the desired compound **4a** as a colorless oil (75% yield over 2 steps, 0.155 g).

^{1}H NMR (500 MHz, $CDCl_3$) δ (ppm) 7.46-7.43 (m, 2H), 7.31-7.28 (m, 2H), 6.00 (dd, *J* =11.2, 17.6 Hz, 1H), 5.74 (dd, *J* =1.9, 17.5 Hz, 1H), 5.57 (dd, *J* =1.9, 11.2 Hz, 1H).

^{13}C NMR (125 MHz, $CDCl_3$) δ (ppm) 133.1, 131.7, 127.5, 122.6, 122.2, 117.0, 89.3, 89.0.

HRMS (ESI) calcd. for $C_{10}H_8Br$ $[M+H]^+$: 206.9803, found: 206.9804.

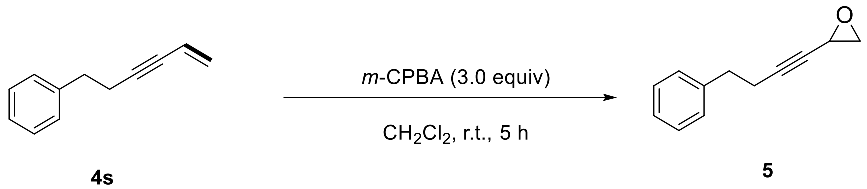

Synthesis of compound 2-(4-phenylbut-1-yn-1-yl)oxirane (5):

The title compound was synthesized according to a literature procedure[14] with some modifications as follows. To a flame dried round bottom flask under nitrogen was added a hex-5-en-3-yn-1-ylbenzene **4s** (0.046 g, 0.3 mmol, 1 equiv) in dry CH_2Cl_2 (7 mL) and cooled to 0 °C. *meta*-Chloroperbenzoic acid (3.0 equiv) was added to the flask at 0 °C. The reaction flask was kept at r.t. for 5 hours. After completion of the reaction, monitored by TLC, the crude was concentrated in vacuo and purified by flash column chromatography to provide the title compound **5** as a colorless oil (78% yield, 0.04 g).

^{1}H NMR (500 MHz, $CDCl_3$): δ (ppm) 7.19-7.32 (m, 5H), 3.32-3.35 (m, 1H), 2.81-2.89 (m, 4H), 2.48-2.52 (m, 2H)

^{13}C NMR (125 MHz, $CDCl_3$): δ (ppm) 140.3, 128.4, 128.4, 126.4, 83.7, 77.5, 48.7, 40.0, 34.7, 20.9

HRMS: (ESI) calcd. for $C_{12}H_{13}O$ $[M+H]^+$: 173.0960, found: 173.0963.

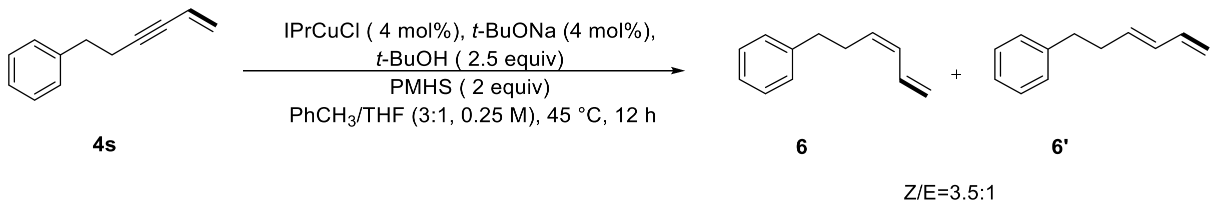

Synthesis of (Z)-hexa-3,5-dien-1-ylbenzene (6):

The title compound was synthesized according to a literature procedure. [15] In an argon-filled glovebox, to a flame dried vial was added IPrCuCl (IPr = 1,3-bis(2,6-diisopropylphenyl)imidazol-2-ylidene, 3.9 mg, 0.008 mmol, 4 mol %,), NaO*t*-Bu (1.5 mg, 0.008 mmol, 4 mol %), and THF (0.2 mL). The catalyst solution was allowed to stir vigorously for 20 min while a reaction vial equipped with a stir bar was charged with hex-5-en-3-yn-1-ylbenzene **4s** (31.2 mg, 0.2 mmol, 1 equiv), polymethylhydrosiloxane (PMHS, 24 mg, 0.4 mmol, 2 equiv), toluene (0.6 mL), and *tert*-butyl alcohol (47 μL, 0.5 mmol, 2.5 equiv). The catalyst solution was transferred to the reaction flask and the reaction mixture was heated to 45 °C for 12 h. The mixture was then diluted with CH_2Cl_2 (20 mL) and filtered through a silica plug with EtOAc (ca. 30 mL). The crude was purified by flash column chromatography with *n*-hexanes to afford the title compound as a colorless oil (16.9 mg, 65% yield, *Z*/*E*=3.5:1). Spectroscopic data is consistent with previous reports.[16]

^{1}H NMR of the major isomer (500 MHz, $CDCl_3$): δ (ppm) 7.31-7.28 (m, 2H), 7.21-7.18 (m, 3H), 6.65-6.57 (m, 1H), 6.02 (t, J = 10.9 Hz, 1H), 5.52-5.47 (m, 1H), 5.19 (d, J =16.8 Hz, 1H), 5.09 (d, J = 10.1 Hz, 1H), 2.74-2.69 (m, 2H), 2.54-2.50 (m, 2H).

^{1}H NMR of the minor isomer (500 MHz, $CDCl_3$): δ (ppm) δ 7.31-7.28 (m, 2H), 7.21-7.18 (m, 3H), 6.35-6.28 (m, 1H), 6.12-6.07 (m, 1H), 5.78-5.72 (m, 1H), 5.10 (d, J = 17.4 Hz, 1H), 4.97 (d, J = 10.1 Hz, 1H), 2.74-2.69 (m, 2H), 2.41 (dd, J = 15.1, 7.3 Hz, 2H).

^{13}C NMR of the major isomer (125 MHz, $CDCl_3$) δ (ppm) 141.7, 132.1, 131.6, 129.7, 128.4, 128.3, 125.9, 117.2, 35.8, 29.6.

^{13}C NMR of the minor isomer (125 MHz, $CDCl_3$) δ (ppm) 141.8, 137.2, 134.3, 131.5, 128.4, 128.3, 125.9, 115.2, 35.7, 34.4.

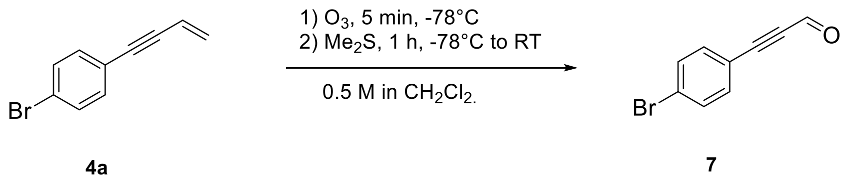

Synthesis of 3-(4-bromophenyl)propiolaldehyde (7):

The title compound was synthesized according to a literature procedure. [12] To a one-dram vial was equipped with a stir bar was added enyne 4a (20.7 mg, 0.1 mmol, 1 equiv) in

dichloromethane (200 μL). The vessel was cooled to –78 °C and a stream of ozone was bubbled through the solution for 5 min, followed by the addition of dimethyl sulfide (74 μL, 1.0 mmol, 10 equiv). The solution was stirred for 1 h at –78 °C and was then allowed to warm to r.t. with stirring continued for another 1 h. Subsequently, the solution was concentrated and purified via column chromatography, eluting with 9:1 (hexanes: ethyl acetate) to give the desired product as a white powder (8.4 mg, 40% yield). Spectroscopic data is consistent with previous reports. [13]

^{1}H NMR (500 MHz, $CDCl_3$): δ (ppm) 9.41 (s, 1H), 7.56 (d, *J* = 8.5 Hz, 2H), 7.46 (d, *J* = 8.5 Hz, 2H).

^{13}C NMR (125 MHz, $CDCl_3$): δ (ppm) 176.7, 134.7, 132.3, 126.4, 118.5, 93.7, 89.2.

HRMS: (ESI) calcd. for C_9H_6OBr $[M+H]^+$: 208.9597, found: 208.9595.

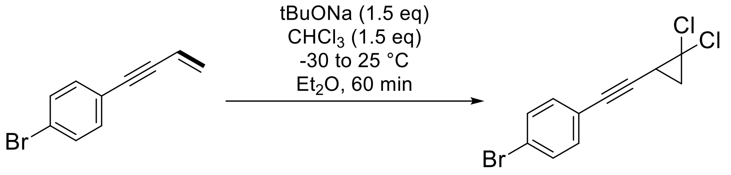

4a 8

Synthesis of 1-bromo-4-((2,2-dichlorocyclopropyl)ethynyl)benzene (8):

The title compound was synthesized according to a modified literature procedure. [9] In an argon-filled glovebox, a flame-dried screwtop vessel with stir bar was charged with sodium *tert*-butoxide (34.8 mg, 0.0362 mmol, 1.50 equiv.), and the vessel was capped, removed from the glovebox, and a Schlenk line inlet of nitrogen atmosphere was added. A solution of enyne **4a** (50.0 mg, 0.24 mmol, 1.0 equiv.) in dry diethyl ether (400 µL) was added, and the mixture was cooled to -40 °C by submerging the vessel in a bath of acetonitrile with dry ice. After cooling for 5 minutes, to the solution was added dropwise over 1 minute a solution of dry chloroform (28.9 µL, 0.0362 mmol, 1.50 equiv.) in dry diethyl ether (400 µL). The solution was stirred in the acetonitrile bath for 15 minutes, transferred to ice-water bath (0 °C) for 15 minutes, then warmed to room temperature (25 °C) for 30 minutes. The mixture was diluted with hexanes and flushed through a short (2 cm) plug of silica and concentrated to dryness *in vacuo*. The crude clear oil was purified by column chromatography (SiO_2, hexanes) to give the title compound as a colorless liquid (42.0 mg, 60% yield).

^{1}H NMR (400 MHz, $CDCl_3$) δ(ppm) 7.44 (d, *J* = 8.5 Hz, 1H), 7.29 (d, *J* = 8.5 Hz, 1H), 2.43 (dd, *J* = 10.3, 7.5 Hz, 1H), 1.95 (dd, *J* = 10.3, 6.9 Hz, 1H), 1.70 (t, *J* = 7.2 Hz, 1H).

^{13}C NMR (101 MHz, $CDCl_3$) δ(ppm) 133.3, 131.6, 122.7, 121.6, 86.3, 81.1, 59.5, 29.6, 22.2.

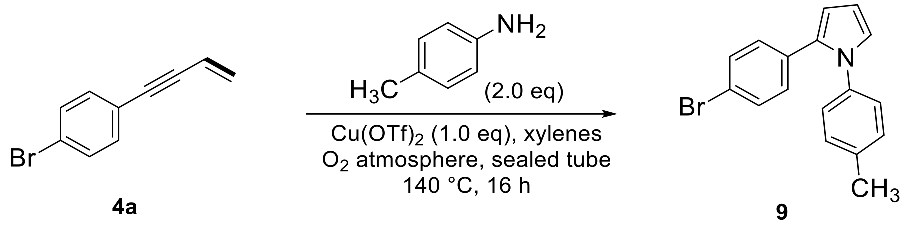

Synthesis of 2-(4-bromophenyl)-1-(*p*-tolyl)-1*H*-pyrrole (9):

The title compound was synthesized according to a literature procedure.[10] A flame-dried screwtop vessel with stir bar was charged with copper(II) triflate (46.0 mg, 0.24 mmol, 1.0 equiv.) then enyne **4a** (50.0 mg, 0.24 mmol, 1.0 equiv.) and xylenes (mixture of isomers, 600 µL). Neat *p*-toluidine (51.7 mg, 0.28 mmol, 2.0 equiv.) was added, and the solution changes from faint yellow to deep blue immediately. The flask is capped and tightly sealed with Parafilm. Using a Schlenk line and an oxygen balloon, the flask is evacuated to vacuum and refilled with oxygen five times to establish an oxygen atmosphere. The sealed flask is placed in a preheated oil bath behind a blast shield and heated at 140 °C for 16 h. After heating, the flask is removed from the bath and cooled to room temperature. The crude, black reaction mixture is diluted with dichloromethane (5 mL), flushed through a short plug of silica (2 x 2 inches), and the eluent concentrated to dryness *in vacuo*. ^{1}H-NMR analysis of the crude reaction mixture indicates ~58% conversion. The crude yellow oil is subjected to column chromatography (SiO_2, 99:1 hexane:Et_2O) to give the title compound **9** as an off-white solid (32.3 mg, 43% yield). Spectroscopic data is consistent with previous reports.[11]

^{1}H NMR (300 MHz, $CDCl_3$): δ(ppm) 7.32 (d, J = 8.7 Hz, 2H), 7.14 (d, J = 8.1 Hz, 2H), 7.04 (d, J = 8.4 Hz, 2H), 7.01 – 6.96 (m, 2H), 6.91 (dd, J = 2.8, 1.8 Hz, 1H), 6.42 (dd, J = 3.6, 1.8 Hz, 1H), 6.34 (dd, J = 3.5, 2.8 Hz, 1H), 2.35 (s, 3H).

Preliminary mechanistic investigations:

A. Kinetic isotopic effect experiments

1. Independent KIE results

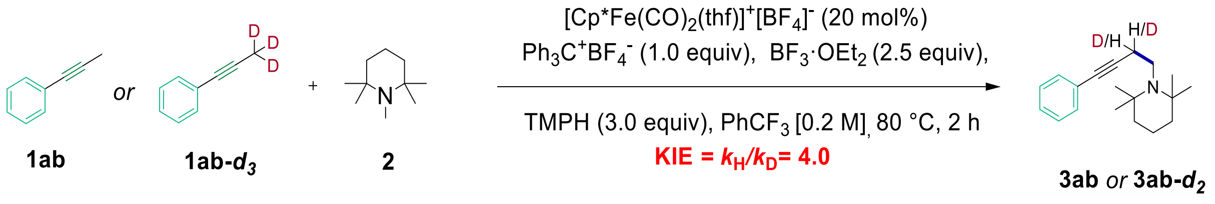

A reaction tube with a magnetic stir bar was capped with a Teflon/silicone septum screw cap and flame dried under vacuum. The reaction tube was cooled under nitrogen and transferred into an argon-filled glovebox. In the glovebox, the suspension of 1,2,2,6,6-pentamethylpiperidine (**2**, 109 μL, 0.6 mmol, 2.0 equiv) and $Ph_3C^+BF_4^−$ (198 mg, 0.6 mmol, 2.0 equiv) in dry trifluorotoluene (1 mL) was stirred at room temperature for 1 h to generate the iminium salt. Then $[Cp^*Fe(CO)_2(thf)]^+[BF_4]^−$ (20 mol %, 24.6 mg), trifluorotoluene (0.5 mL), $BF_3·OEt_2$ (93 μL, 0.75 mmol, 2.5 equiv), alkyne **1ab** or **1ab-d3** (0.3 mmol, 1.0 equiv), and 2,2,6,6-tetramethylpiperidine (205 μL, 1.2 mmol, 4.0 equiv) were added in rapid succession. The reaction tube was capped and removed from the glovebox. The reaction tube was placed in an oil bath, preheated to 80 °C, whereit was stirred for 2 h. Yields were determined by ^1H NMR using 1,1,2,2-tetrachloroethane as the internal standard.

2. Competition KIE results

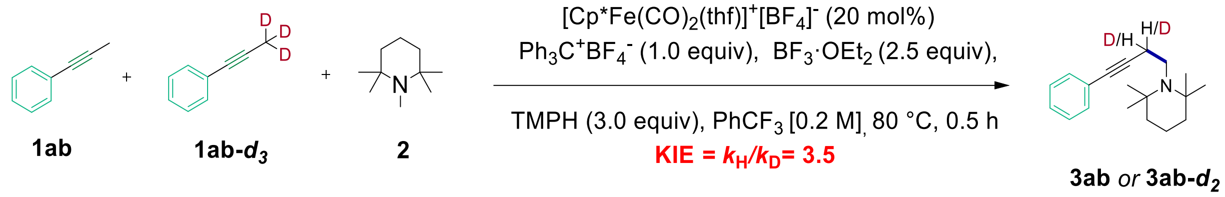

A reaction tube equipped with a magnetic stir bar was capped with a Teflon/silicone septum screw cap and flame dried under vacuum. The reaction tube was cooled under nitrogen and transferred into an argon-filled glovebox. In the glovebox, the suspension of 1,2,2,6,6-pentamethylpiperidine (**2**, 109 μL, 0.6 mmol, 2.0 equiv) and $Ph_3C^+BF_4^−$ (198 mg, 0.6 mmol, 2.0 equiv) in dry trifluorotoluene (1 mL) was stirred at room temperature for 1 h to generate the iminium salt. Then $[Cp^*Fe(CO)_2(thf)]^+[BF_4]^−$ (20 mol %, 24.6 mg), trifluorotoluene (0.5 mL), $BF_3·OEt_2$ (93 μL, 0.75 mmol, 2.5 equiv), alkyne **1ab** and **1ab-d3** (0.3 mmol, 1.0 equiv), and 2,2,6,6-tetramethylpiperidine (205 μL, 1.2 mmol, 4.0 equiv) were added in rapid succession. The reaction tube was capped and removed from the glovebox. The reaction tube was placed in an oil bath, preheated to 80 °C, where it was stirred for 0.5 h. Yields were determined by ^1H NMR using 1,1,2,2-tetrachloroethane as the internal standard.

B. Investigating the role of Lewis acid

1. Effect of added THF

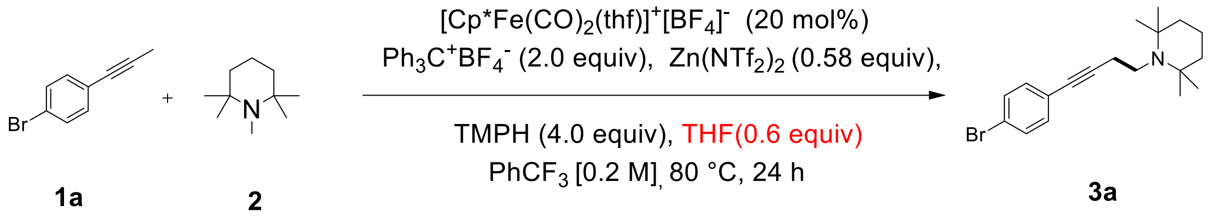

A reaction tube equipped with a magnetic stir bar was capped with a Teflon/silicone septum screw cap and flame dried under vacuum. The reaction tube was cooled under nitrogen and transferred into an argon-filled glovebox. In the glovebox, the suspension of 1,2,2,6,6-pentamethylpiperidine (**2**, 109 µL, 0.6 mmol, 2.0 equiv) and $Ph_3C^+BF_4^-$ (198 mg, 0.6 mmol, 2.0 equiv) in dry trifluorotoluene (1 mL) was stirred at room temperature for 1 h to generate the iminium salt. Then [Cp*Fe(CO)$_2$(thf)]+[BF4] (24.6 mg, 20 mol%, 0.06 mmol), trifluorotoluene (0.5 mL), $Zn(NTf_2)_2$ (108.9 mg, 0.174 mmol, 0.58 equiv), alkyne **1a** (58 mg, 0.3 mmol, 1.0 equiv), 2,2,6,6-tetramethylpiperidine (205 µL, 1.2 mmol, 4.0 equiv) and THF (14.6 µL, 0.18 mmol, 0.6 equiv) were added in rapid succession. The reaction tube was capped and removed from the glovebox. The reaction tube was placed in an oil bath, preheated to 80 °C, where it was stirred for 24 h. After completion of the reaction, the reaction mixture was cooled to room temperature. The crude mixture was concentrated in vacuo, subjected to a short silica plug eluting with 1:1 (ethyl acetate: hexanes) to provide the crude homopropargylic amine product **3a.** Yields were determined by ^{1}H NMR using 1,1,2,2-tetrachloroethane as the internal standard (80% NMR yield).

Preparation of allenyliron species (**10-1a**)

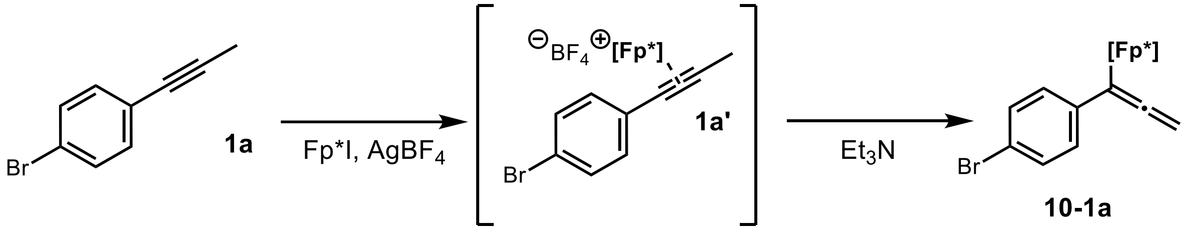

In a nitrogen-filled glovebox, a flame-dried round bottom flask containing a stir bar was charged with Fe(CO)$_2$(C$_{10}$H$_{15}$)I (Fp*I, 200.0 mg, 0.534 mmol, 1.0 equiv.), and silver tetrafluoroborate (127.8 mg, , 0.66 mmol, 1.23 equiv.), then was added neat alkyne **1a** (135.6 mg, 0.695 mmol, 1.3 equiv.) then chloroform (1 mL). The mixture was vigorously stirred in the

dark until the purple color of the supernatant turned deep orange (2 h) at room temperature (25 °C). After 2 h, neat triethylamine (0.15 mL, 1.07 mmol, 2 equiv) was added dropwise, and the reaction mixture color changes immediately from deep red to faintly orange. The reaction mixture was stirred for an additional 2 h and then diluted with dry diethyl ether (10 mL). The mixture was sonicated for 5 minutes then flushed through a short plug of neutral alumina (Brockmann I, 150 mesh, 3 cm) to remove a dark black-red band at the baseline. Additional diethyl ether (20 mL) was flushed through the plug until the filtrate was colorless. The orange filtrate was concentrated to dryness *in vacuo* to get bright orange crystals of **10-1a** upon subjection to high vacuum.

^{1}H NMR (300 MHz, $CDCl_3$) δ 7.31 (d, *J* = 8.5 Hz, 1H), 7.20 (d, *J* = 8.5 Hz, 1H), 4.14 (s, 2H), 1.72 (s, 15H).

Fp*I was prepared following the literature procedures.[17]

Note: Due to use of excess alkyne (1.3 equiv) in the first step, product **10-1a** had around 38% of starting material alkyne **1a** remaining as shown in the following NMR.

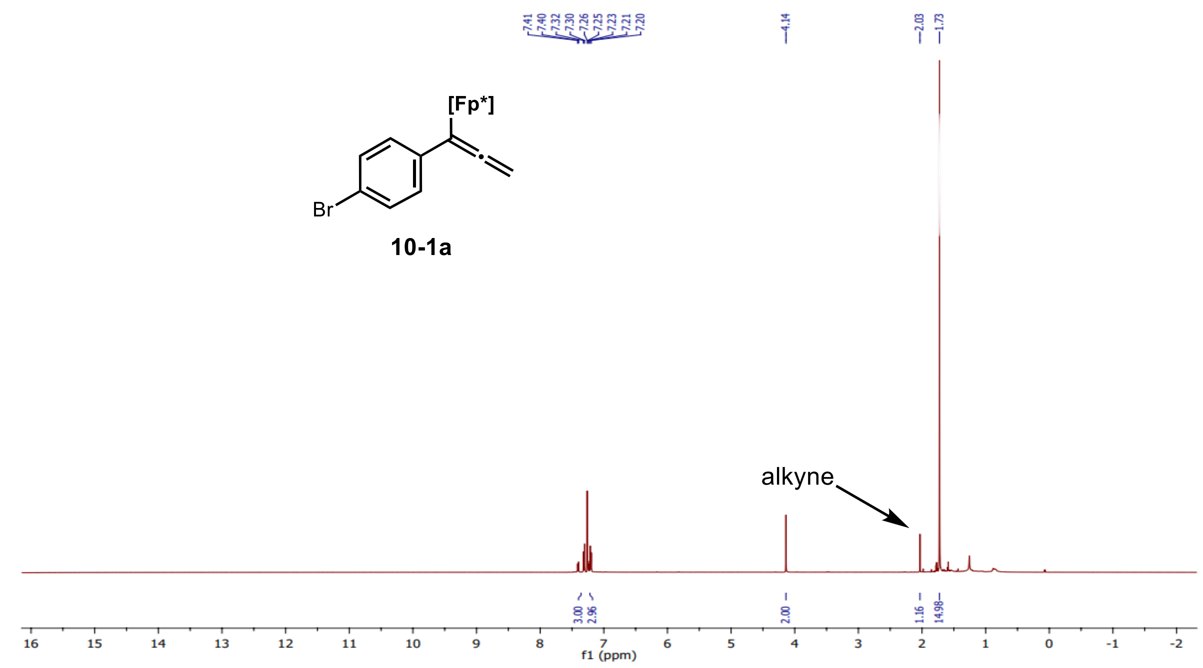

2. Use of an allenyliron species (10-1a) as catalyst with and without Lewis acid:

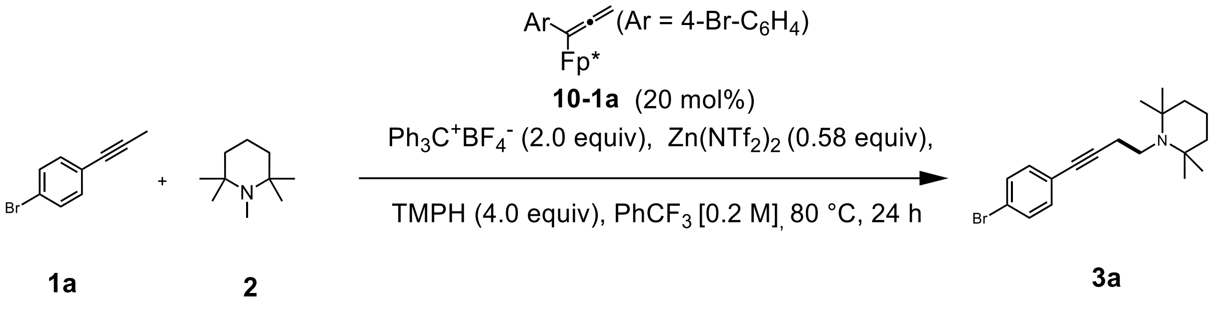

A reaction tube equipped with a magnetic stir bar was capped with a Teflon/silicone septum screw cap and flame dried under vacuum. The reaction tube was cooled under nitrogen and transferred into an argon-filled glovebox. In the glovebox, the suspension of 1,2,2,6,6-pentamethylpiperidine (**2**, 109 µL, 0.6 mmol, 2.0 equiv) and $Ph_3C^+BF_4^-$ (198 mg, 0.6 mmol, 2.0 equiv) in dry trifluorotoluene (1 mL) was stirred at room temperature for 1 h to generate the iminium salt. Then **10-1a** (20 mol %), trifluorotoluene (0.5 mL), $Zn(NTf_2)_2$ (108.9 mg, 0.174 mmol, 0.58 equiv), alkyne **1a** (0.3 mmol, 1.0 equiv), and 2,2,6,6-tetramethylpiperidine (205 µL, 1.2 mmol, 4.0 equiv) were added in rapid succession.* The reaction tube was capped and removed from the glovebox. The reaction tube was placed in an oil bath, preheated to 80 °C, where it was stirred for 24 h. After completion of the reaction, the reaction mixture was cooled to room temperature. The crude mixture was concentrated in vacuo, subjected to a short silica plug eluting with 1:1 (ethyl acetate: hexanes) to provide the crude homopropargylic amine product **3a.** Yields were determined by ^1H NMR using 1,1,2,2-tetrachloroethane as the internal standard (90% NMR yield and 36% without Lewis acid).

*The amount of **10-1a** was calculated according to the ratio of **10-1a** and alkyne **1a** in the sample of the allenyliron prepared above.

New [Fp*] + sources as bench-stable precatalysts:

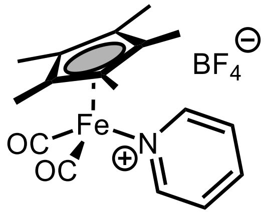

10c

Preparation of catalyst 10c. To a flame-dried vessel containing parent catalyst **10a** ($[Fp^*(thf)]^+BF_4^-$, 365 μmol, 150 mg) was added neat, distilled pyridine (5 mL, excess). The red solution gradually changed from bright red to orange over several hours. The reaction mixture was stirred overnight (12 h) and took on a faintly green tint. Separately, a round bottom flask was filled with diethyl ether (30 mL), and the flask was sonicated. The reaction mixture was transferred by pipette into the ethereal solution dropwise while sonicating, causing the immediate formation of an orange precipitate. Two additional portions of chloroform (2 × 1 mL) were used to completely transfer the reaction mixture to the sonicating flask. The precipitation mixture was filtered through a short Celite plug using a sintered glass filter funnel with vacuum adapter, trapping the orange and green solids while removing the ethereal solvent mixture. The collected solids were washed with dry diethyl ether (2 × 5 mL). The collection bulb beneath the funnel was replaced with a clean vessel. The solids were collected by flushing the Celite plug with dry dichloromethane (20 mL, until the filtrate ran colorless), and the orange/green filtrate was collected and concentrated to dryness *in vacuo*. The brownish solid was then redissolved in dichloromethane (0.5 mL) then while the solution was sonicated, dropwise over five minutes was added dry diethyl ether (20 mL) causing the precipitation of an orange-brown solid. The solid falls to the bottom of the flask after letting the mixture sit for 5 min. The greenish filtrate was decanted and discarded. Another portion of ether (20 mL) was added to the flask, and the flask was sonicated for 5 min before the ethereal supernatant was decanted and discarded. The flask was further dried *in vacuo* to give the product as an orange-brown solid (65.0 mg, 42.6%).

^{1}H NMR (400 MHz, $CDCl_3$) δ 8.45 (bs, 2H), 7.91 (bs, 1H), 7.59 (bs, 2H), 1.76 (s, 15H).

^{13}C NMR (101 MHz, $CDCl_3$) δ 211.6, 157.5, 139.8, 128.2, 99.2, 9.7.

^{19}F NMR (376 MHz, $CDCl_3$) δ -152.8.

IR (ATR, neat): ν(CO) = 2030, 1992 cm^{-1}

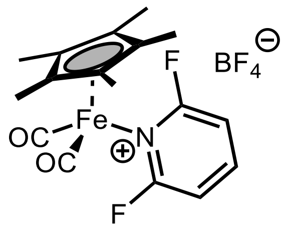

10d

Preparation of catalyst 10d. To a flame-dried vessel containing Fp*I (1.34 mmol, 500 mg) and $AgBF_4$ (1.47 mmol, 273 mg, 1.05 equiv) was added neat 2,6-difluoropyridine (135 µL, 1.47 mmol, 1.1 equiv) causing the color to change from black to dark orange within minutes. After 1 min, dry chloroform (5 mL) was added dropwise, and the reaction mixture was sonicated for 2 min to uniformly suspend the contents of the flask. The vessel was covered with aluminum foil and vigorously stirred for 12 h during which the color of the reaction mixture changed to a deep, dark orange. Separately, a round bottom flask was filled with hexanes (30 mL), and the flask was sonicated. The reaction mixture was transferred by pipette into the hexanes solution dropwise causing the immediate formation of an orange precipitate. Two additional portions of chloroform (2 × 1 mL) were used to completely transfer the reaction mixture to the sonicated flask. The precipitation mixture was filtered through a short Celite plug using a sintered glass filter funnel with vacuum adapter, trapping the orange solid while removing the solvent mixture. The collected solids were washed with dry diethyl ether (2 × 10 mL), and the ethereal filtrate was discarded. The collection flask was exchanged with a flask, then the Celite plug was flushed with dry dichloromethane (25 mL, until the filtrate ran colorless). The orange filtrate was concentrated to dryness *in vacuo* and the deep orange solid was transferred into a weighed vial for storage (570 mg, 95%). An X-ray quality crystal was grown by vapor-diffusion method: 20 mg of the solid was dissolved in dichloromethane (200 µL) in a 1 mL vial. This vial was placed in a second chamber of hexanes. The apparatus was sealed to allow for solvent exchange, producing bright orange needles after 8 h.

^{1}H NMR (500 MHz, CD_2Cl_2) δ 8.31 (p, *J* = 7.8 Hz, 1H), 7.30 (d, *J* = 8.2 Hz, 2H), 1.73 (s, 15H).

^{13}C NMR (151 MHz, CD_2Cl_2) δ 211.1, 163.0 (d, *J* = 261.5 Hz), 149.4 (t, *J* = 10.8 Hz), 109.2 (dd, *J* = 25.5, 8.6 Hz), 98.8 (s), 9.4 (s).

^{19}F NMR (376 MHz, $CDCl_3$) δ –48.2, –153.1.

IR (ATR, neat): ν(CO) = 2040, 1999 cm^{-1}

A. Generation of catalytically active species in situ from $[Fp^*]_2$

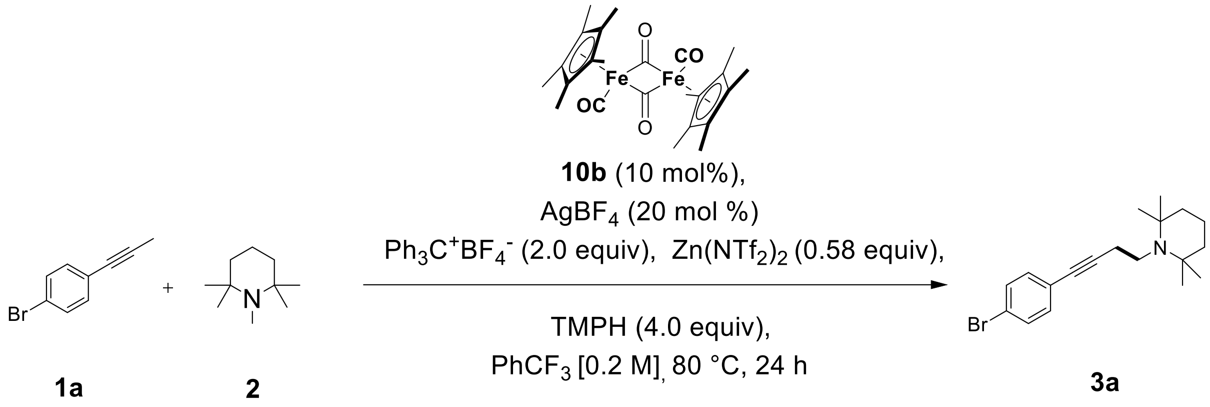

Two reaction tubes equipped with a magnetic stir bar was capped with a Teflon/silicone septum screw cap and flame dried under vacuum. The reaction tubes were cooled under nitrogen and transferred into an argon-filled glovebox. In the glovebox, to one reaction tube (TB1) the suspension of 1,2,2,6,6-pentamethylpiperidine (**2**, 109 µL, 0.6 mmol, 2.0 equiv) and $Ph_3C^+BF_4^-$ (198 mg, 0.6 mmol, 2.0 equiv) in dry trifluorotoluene (1 mL) was stirred at room temperature for 1 h to generate the iminium salt. To the other reaction tube (TB2) was added **10b** (14.4 mg, 10 mol%, 0.03 mmol), $AgBF_4$ (20 mol%, 11.6 mg, 0.06 mmol) and $PhCH_3$ (0.5 mL) was stirred for 1 h to generate the alkyne-iron complex **1a'** *in situ*. After 1 h, to TB2 was added the contents of TB1, along with $Zn(NTf_2)_2$ (108.9 mg, 0.174 mmol, 0.58 equiv), alkyne **1a** (0.3 mmol, 1.0 equiv), and 2,2,6,6-tetramethylpiperidine (205 µL, 1.2 mmol, 4.0 equiv) in rapid succession. The reaction tube was capped and removed from the glovebox. The reaction tube was placed in an oil bath, preheated to 80 °C, where it was stirred for 24 h. After completion of the reaction, the reaction mixture was cooled to room temperature. The crude mixture was concentrated in vacuo, subjected to a short silica plug eluting with 1:1 (ethyl acetate: hexanes) to provide the crude homopropargylic amine product **3a.** Yields were determined by ^1H NMR using 1,1,2,2-tetrachloroethane as the internal standard (53% NMR yield with Lewis acid and 25% without Lewis acid).

B. [Fp*]+-pyridine complexes as precatalysts

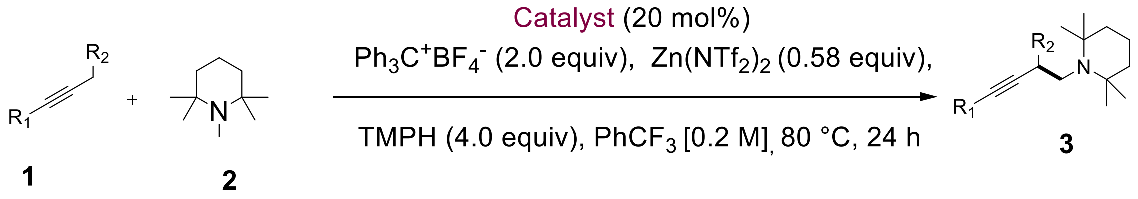

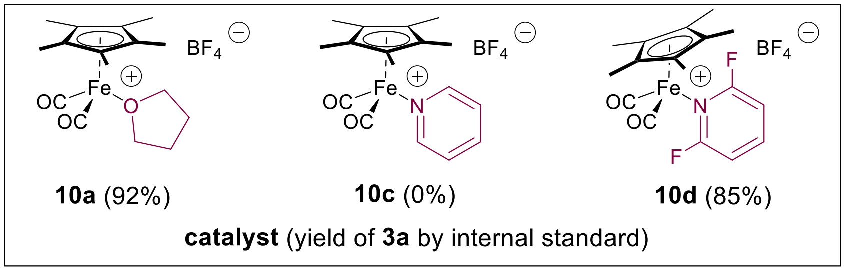

A reaction tube equipped with a magnetic stir bar was capped with a Teflon/silicone septum screw cap and flame dried under vacuum. The reaction tube was cooled under nitrogen and transferred into an argon-filled glovebox. In the glovebox, the suspension of 1,2,2,6,6-pentamethylpiperidine (**2**, 109 µL, 0.6 mmol, 2.0 equiv) and $Ph_3C^+BF_4^-$ (198 mg, 0.6 mmol, 2.0 equiv) in dry trifluorotoluene (1 mL) was stirred at room temperature for 1 h to generate the iminium salt. Then **10a** or **10c** or **10d** (20 mol %), trifluorotoluene (0.5 mL), $Zn(NTf_2)_2$ (108.9 mg, 0.174 mmol, 0.58 equiv), alkyne **1** (0.3 mmol, 1.0 equiv), and 2,2,6,6-tetramethylpiperidine (205 µL, 1.2 mmol, 4.0 equiv) were added in rapid succession. The reaction tube was capped and removed from the glovebox. The reaction tube was placed in an oil bath, preheated to 80 °C, where it was stirred for 24 h. After completion of the reaction, the reaction mixture was cooled to room temperature. The crude mixture was concentrated in vacuo, subjected to a short silica plug eluting with 1:1 (ethyl acetate: hexanes) to provide the crude homopropargylic amine product **3.** Yields were determined by ^{1}H NMR using 1,1,2,2-tetrachloroethane as the internal standard.

Entry	Product	Catalyst	Yield [%]
1	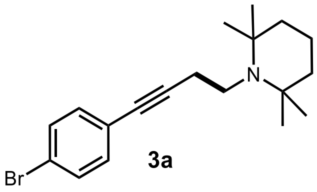 **3a**	**10a**	92
		**10c**	0
		**10d**	85
2	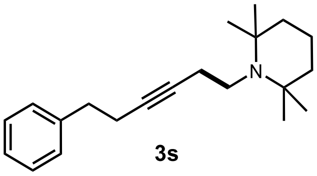 **3s**	**10a**	89
		**10c**	0
		**10d**	86
3	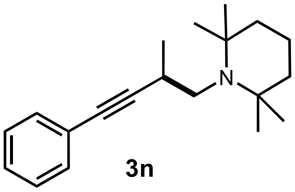 **3n**	**10a**	80
		**10c**	0
		**10d**	75

X-ray crystal structural data:

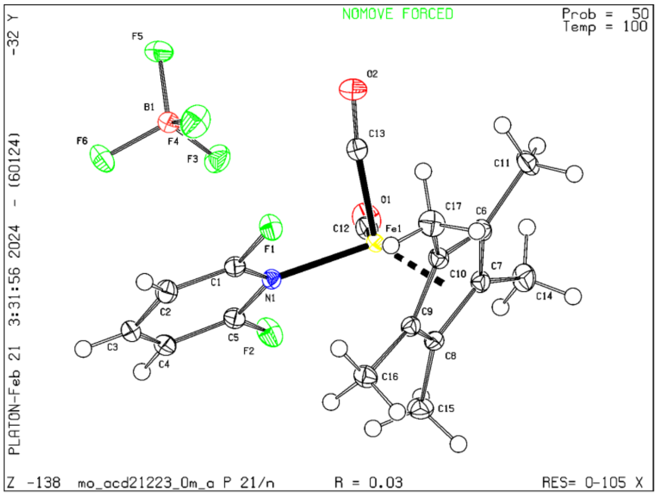

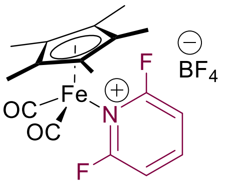

10d

X-ray crystal structure of **10d**. The ellipsoid contour probability is set at 50%.

Datablock: mo_acd21223_0m_a

Bond precision:	C-C = 0.0016 A		Wavelength=0.71073
Cell:	a=10.2685(3)	b=15.4548(5)	c=11.8916(4)
	alpha=90	beta=104.108(1)	gamma=90
Temperature:	100 K		

	Calculated	Reported
Volume	1830.25(10)	1830.25(10)
Space group	P 21/n	P 21/n
Hall group	-P 2yn	-P 2yn
Moiety formula	C17 H18 F2 Fe N O2, B F4	?
Sum formula	C17 H18 B F6 Fe N O2	C17 H18 B F6 Fe N O2
Mr	448.98	448.98
Dx,g cm-3	1.629	1.629
Z	4	4
Mu (mm-1)	0.895	0.895
F000	912.0	912.0
F000’	914.00	
h,k,lmax	15,23,18	15,23,18
Nref	6978	6966
Tmin,Tmax	0.918,0.965	0.860,0.970
Tmin’	0.851	

Correction method= # Reported T Limits: Tmin=0.860 Tmax=0.970
AbsCorr = MULTI-SCAN

Data completeness= 0.998 Theta(max)= 33.142

R(reflections)= 0.0307(6054) wR2(reflections)= 0.0903(6966)

S = 1.029 Npar= 258

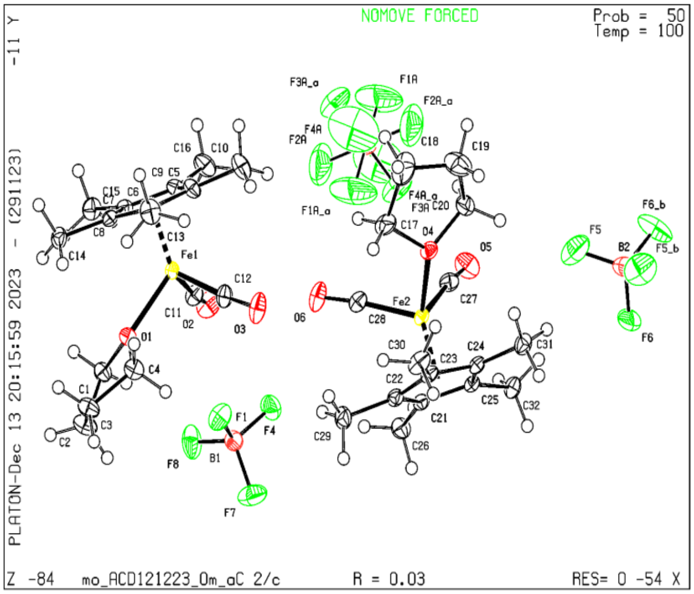

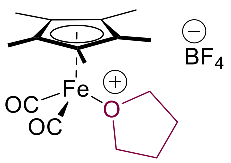

10a

X-ray crystal structure of **10a**. The ellipsoid contour probability is set at 50%.

Datablock: mo_ACD121223_0m_a

Bond precision: C-C = 0.0026 A   Wavelength=0.71073

Cell:	a=33.0304(12)	b=8.5977(3)	c=26.7895(9)
	alpha=90	beta=110.297(1)	gamma=90
Temperature:	100 K		

	Calculated	Reported
Volume	7135.4(4)	7135.4(4)
Space group	C 2/c	C 2/c
Hall group	-C 2yc	-C 2yc
Moiety formula	C16 H23 Fe O3, B F4	?
Sum formula	C16 H23 B F4 Fe O3	C32 H46 B2 F8 Fe2 O6
Mr	406.00	812.01
Dx,g cm-3	1.512	1.512
Z	16	8
Mu (mm-1)	0.897	0.897
F000	3360.0	3360.0
F000'	3367.57	
h,k,lmax	44,11,35	44,11,35
Nref	8945	8907
Tmin,Tmax	0.917,0.996	0.620,0.750
Tmin'	0.898	

Correction method= # Reported T Limits: Tmin=0.620 Tmax=0.750
AbsCorr = MULTI-SCAN

Data completeness= 0.996   Theta(max)= 28.372

R(reflections)= 0.0338(7650)   wR2(reflections)= 0.0862(8907)

S = 1.030   Npar= 481

References:

(1) Moon, J.; Jeong, M.; Nam, H.; Ju, J.; Moon, J. H.; Jung, H. M.; Lee, S. One-Pot Synthesis of Diarylalkynes Using Palladium-Catalyzed Sonogashira Reaction and Decarboxylative Coupling of sp Carbon and sp2 Carbon. *Org. Lett.* **2008**, *10*, 945-948.
(2) Ghosh, A. K.; Shahabi, D. Synthesis of amide derivatives for electron deficient amines and functionalized carboxylic acids using EDC and DMAP and a catalytic amount of HOBt as the coupling reagents. *Tetrahedron Lett.* **2021**, *63*.
(3) Yang, J.-W.; Tan, G.-Q.; Liang, K.-C.; Xu, K.-D.; Su, M.; Liu, F. Copper-Catalyzed, N-Directed Distal C (sp^3)–H Functionalization toward Azepanes. *Org. Lett.* **2022**, *24* , 7796-7800.
(4) Zhang, Y.; Yu, B.; Gao, B.; Zhang, T.; Huang, H. Triple-bond insertion triggers highly regioselective 1, 4-aminomethylamination of 1, 3-enynes with aminals enabled by Pd-catalyzed C–N bond activation. *Org. Lett.* **2019**, *21* , 535-539.
(5) Yang, S.-Q.; Wang, Y.-F.; Zhao, W.-C.; Lin, G.-Q.; He, Z.-T. Stereodivergent Synthesis of Tertiary Fluoride-Tethered Allenes viaCopper and Palladium Dual Catalysis. *J. Am. Chem. Soc.* **2021**, *143*, 19, 7285–7291.
(6) Song, Y.; Song, S.; Duan, X.; Wu, X.; Jiang, F.; Zhang, Y.; Fan, J.; Huang, X.; Fu, C.; Ma, S. Copper-catalyzed radical approach to allenyl iodides. *ChemComm* **2019**, *55* , 11774-11777.
(7) Bayeh-Romero, L.; Buchwald, S. L. Copper Hydride Catalyzed Enantioselective Synthesis of Axially Chiral 1,3-Disubstituted Allenes. *J. Am. Chem. Soc.* **2019**, *141*, 13788-13794.
(8) Wang, F.; Wang, D.; Zhou, Y.; Liang, L.; Lu, R.; Chen, P.; Lin, Z.; Liu, G. Divergent synthesis of CF_3-substituted allenyl nitriles by ligand-controlled radical 1, 2-and 1, 4-addition to 1, 3-enynes. *Angew. Chem. Int. Ed.* **2018**, *57* , 7140-7145.
(9) Vogel, E. W. Klug, A. Bruer. *Org. Synth* **1974**, *54*.
(10) Bharathiraja, G.; Sengoden, M.; Kannan, M.; Punniyamurthy, T. Expedient synthesis of tetrasubstituted pyrroles via a copper-catalyzed cascade inter-/intramolecular cyclization of 1,3-enynes carry a nitro group with amines. *Org. Biomol. Chem* **2015**, *13*, 2786-2792.
(11) Zheng, J.; Huang, L.; Huang, C.; Wu, W.; Jiang, H. Synthesis of polysubstituted pyrroles via Pd-catalyzed oxidative alkene C–H bond arylation and amination. *J. Org. Chem.* **2015**, *80*, 1235-1242.
(12) Albert, B. J.; Sivaramakrishnan, A.; Naka, T.; Koide, K. Total Synthesis of FR901464, an Antitumor Agent that Regulates the Transcription of Oncogenes and Tumor Suppressor Genes. *J. Am. Chem. Soc.* **2006**, *128* , 2792-2793.
(13) Xu, Y.; Wang, Q.; Wu, Y.; Zeng, Z.; Rudolph, M.; Hashmi, A. S. K. Gold-Catalyzed Synthesis of 2,5-Disubstituted Oxazoles from Carboxamides and Propynals. *Advanced Synthesis & Catalysis* **2019**, *361* , 2309-2314.
(14) Wang, L.; Maddess, M. L.; Lautens, M. Convenient access to functionalized vinylcyclopentenols from alkynyloxiranes. *The Journal of organic chemistry* **2007**, *72* , 1822-1825.
(15) Whittaker, A. M.; Lalic, G. Monophasic catalytic system for the selective semireduction of alkynes. *Organic letters* **2013**, *15* , 1112-1115.
(16) (a) Kliman, L. T.; Mlynarski, S. N.; Ferris, G. E.; Morken, J. P. Catalytic enantioselective 1, 2-diboration of 1, 3-dienes: versatile reagents for stereoselective allylation. *Angew. Chem. Int. Ed*. **2012**, *124* , 536-539. (b) Liu, B.; Liu, T.-Y.; Luo, S.-W.; Gong, L.-Z. Asymmetric Hetero-Diels−Alder Reaction of Diazenes Catalyzed byChiral Silver Phosphate: Water Participates in the Catalysis andStereocontrol. *Org. Lett.* **2014**, *16* , 6164–6167.
(17) Akita, M.; Terada, M.; Tanaka, M.; Morooka, Y. Some additional aspects of versatile starting compounds for cationic organoiron complexes: molecular structure of the aqua complex [(η5-C_5Me_4Et) $Fe(CO)_2(OH_2)$] BF_4 and solution behavior of the THF complex [(η^5-C_5R_5)$Fe(CO)_2$(THF)] BF_4. *J. Organomet. Chem.* **1996**, *510* , 255-261.

Copies of ^{1}H and ^{13}C NMR spectra for products and substrates:

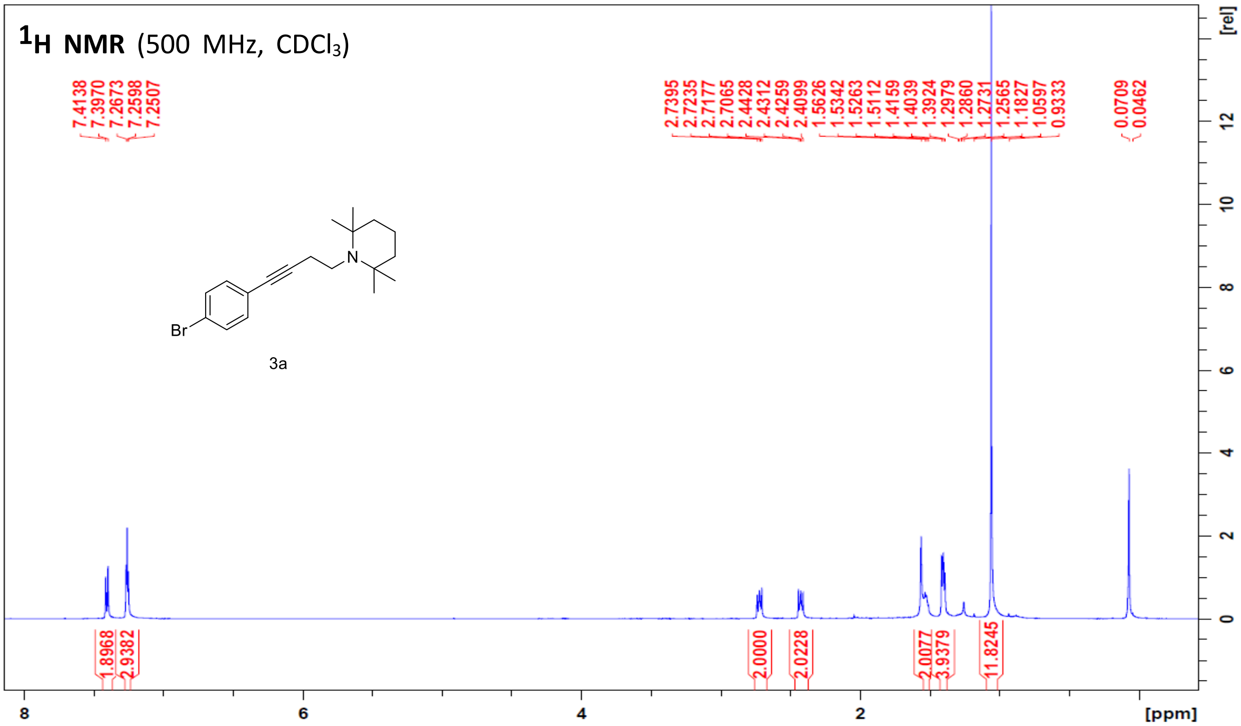

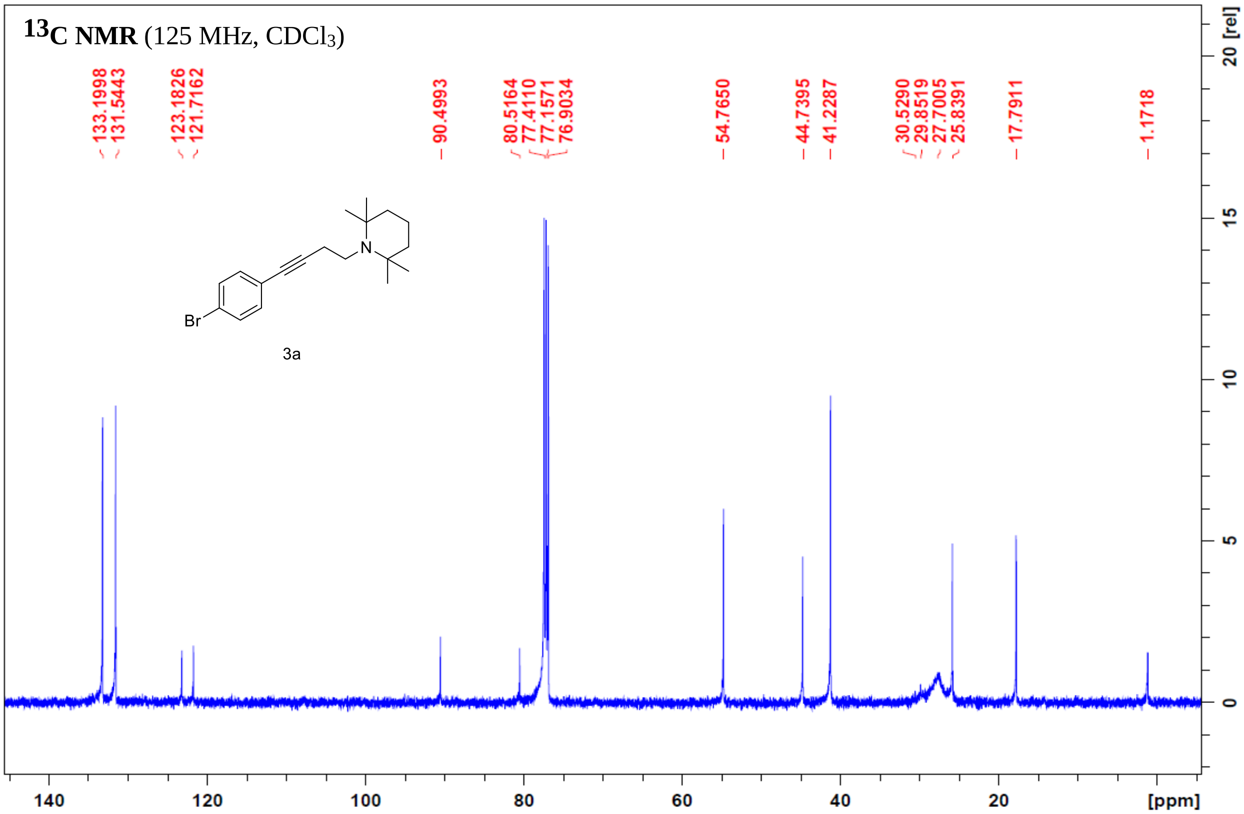

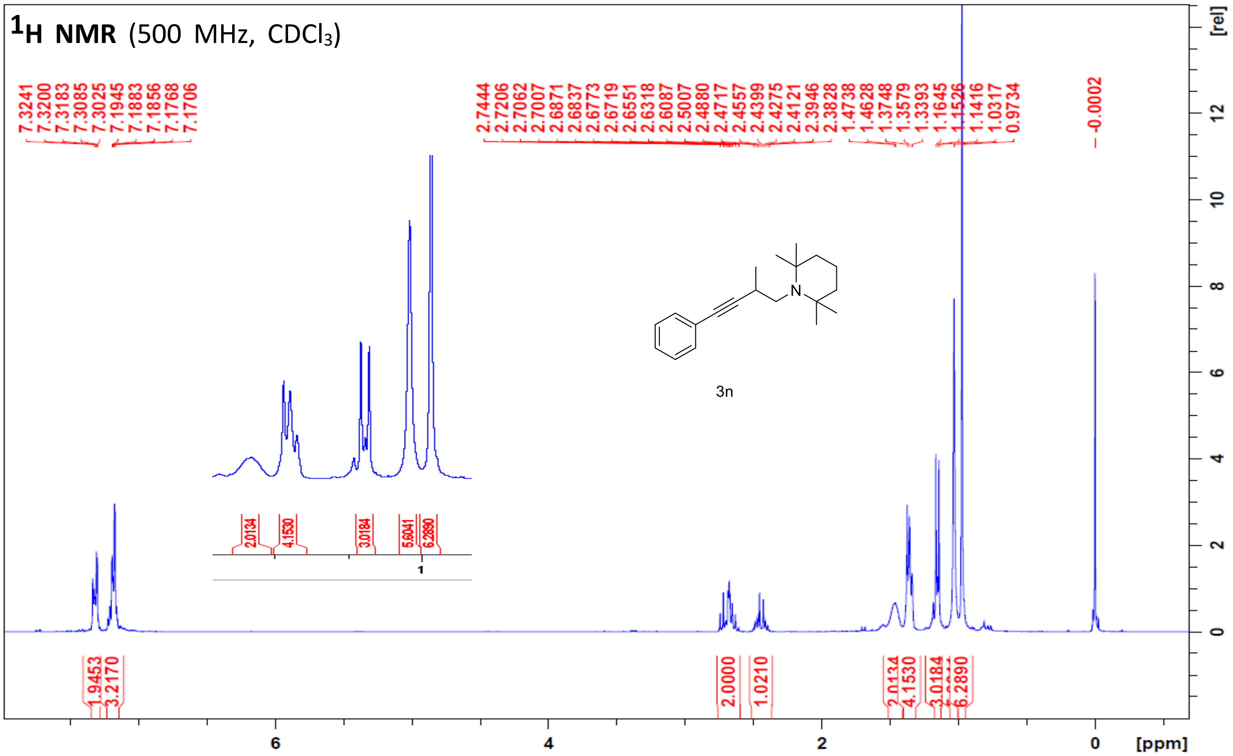
1H NMR (500 MHz, CDCl3)
3n
[rel]
[ppm]

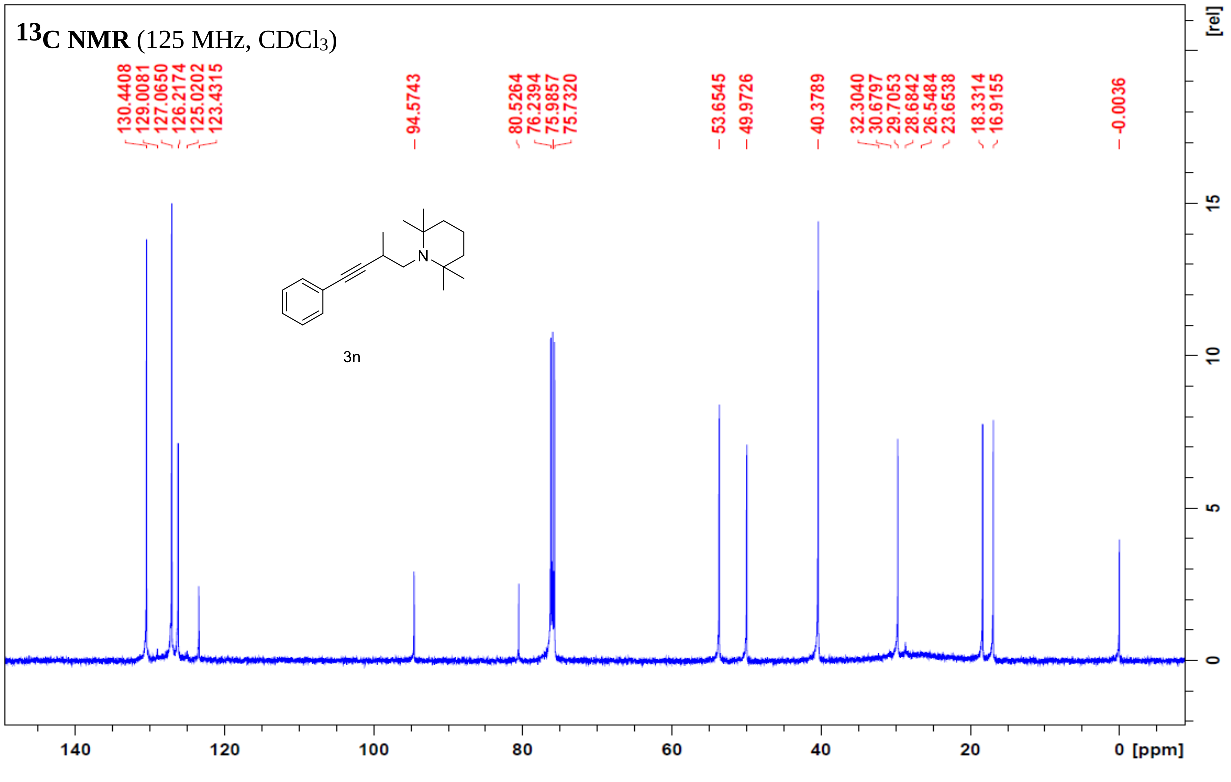
13C NMR (125 MHz, CDCl3)
3n
[rel]
[ppm]

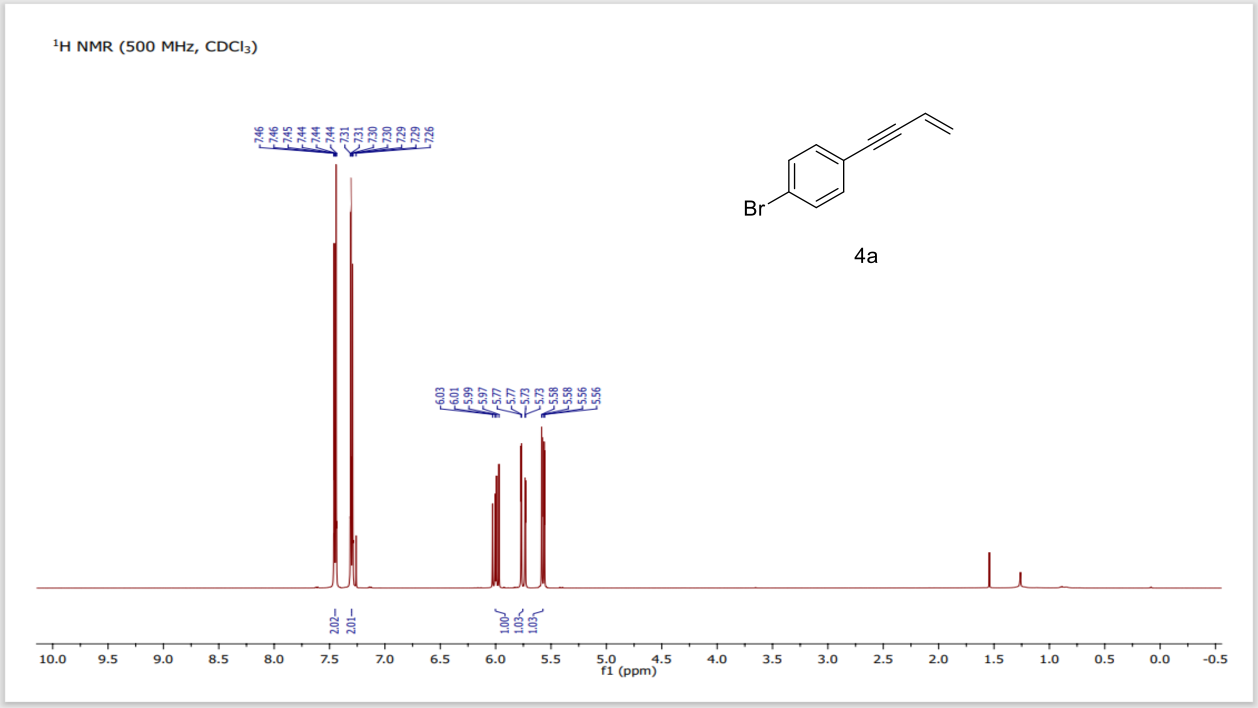

¹H NMR (500 MHz, CDCl₃)
7.46 7.46 7.45 7.44 7.44 7.44 7.31 7.31 7.30 7.30 7.29 7.29 7.26
6.03 6.01 5.99 5.97 5.77 5.77 5.73 5.73 5.58 5.58 5.56 5.56
2.02 2.01
1.00 1.03 1.03
Br
4a
10.0 9.5 9.0 8.5 8.0 7.5 7.0 6.5 6.0 5.5 5.0 4.5 4.0 3.5 3.0 2.5 2.0 1.5 1.0 0.5 0.0 -0.5
f1 (ppm)

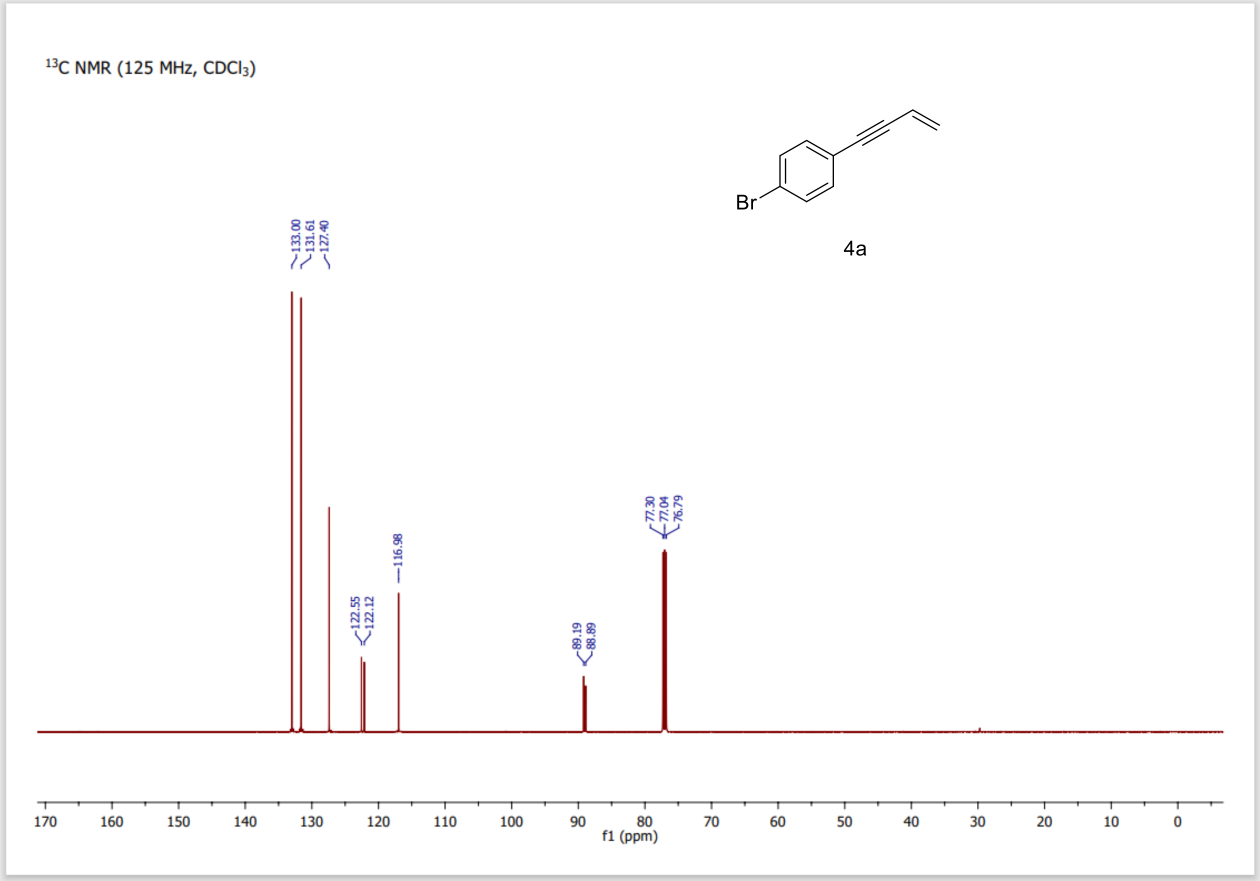

¹³C NMR (125 MHz, CDCl₃)
133.00 131.61 127.40
122.55 122.12
116.98
89.19 88.89
77.30 77.04 76.79
Br
4a
170 160 150 140 130 120 110 100 90 80 70 60 50 40 30 20 10 0
f1 (ppm)

^{1}H NMR (500 MHz, $CDCl_3$)

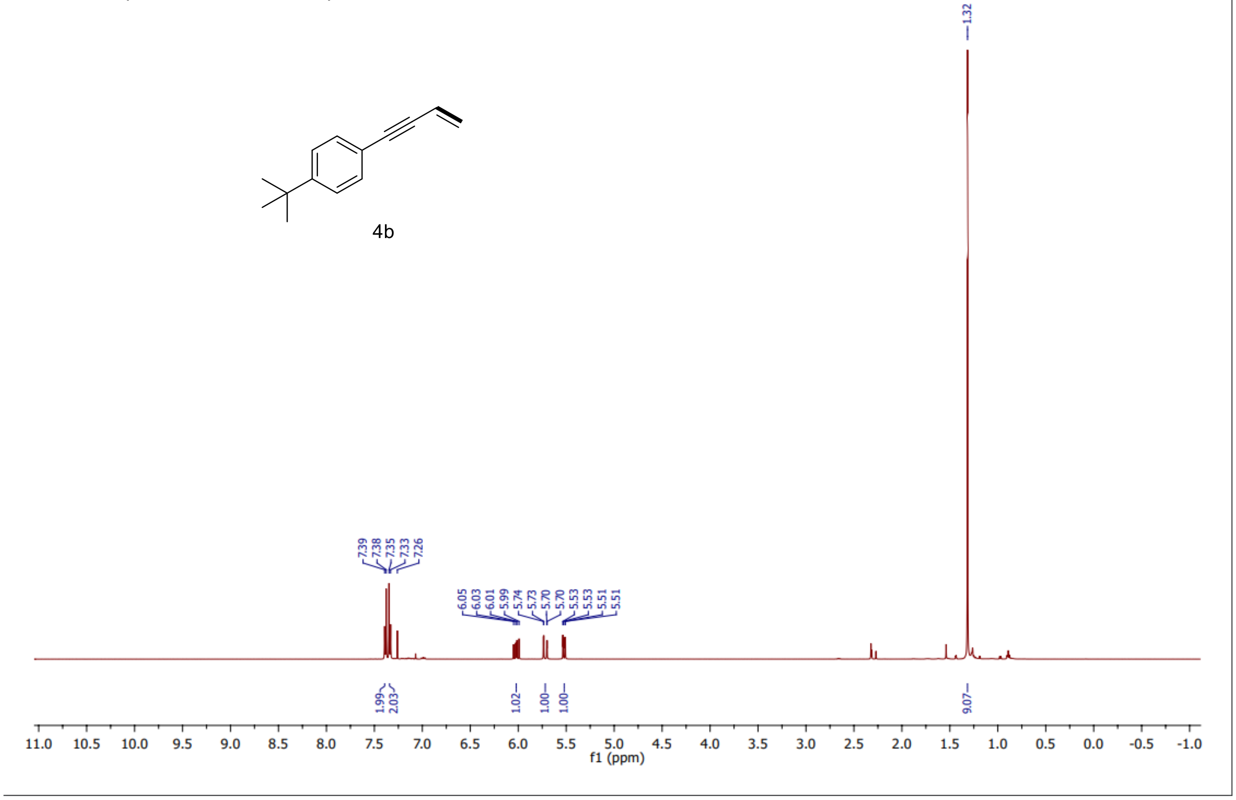

^{13}C NMR (125 MHz, $CDCl_3$)

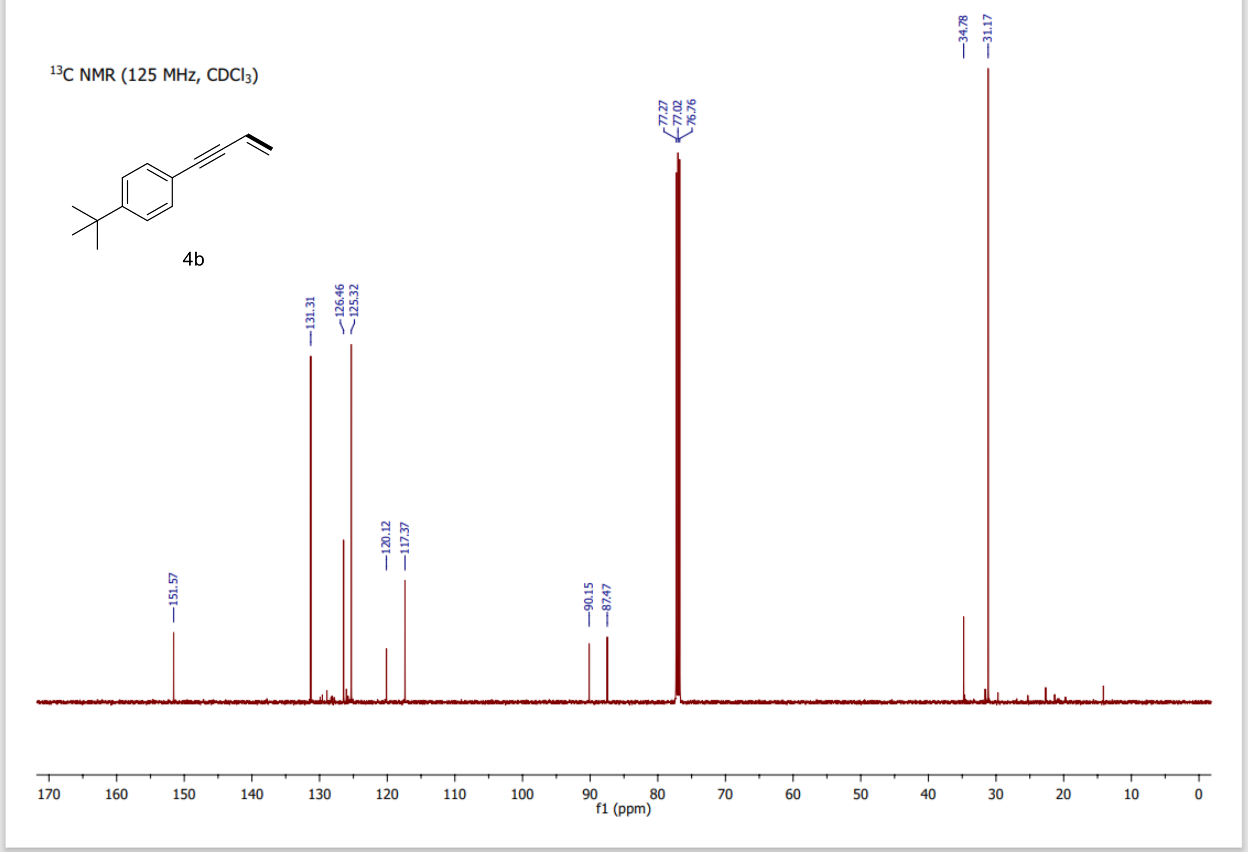

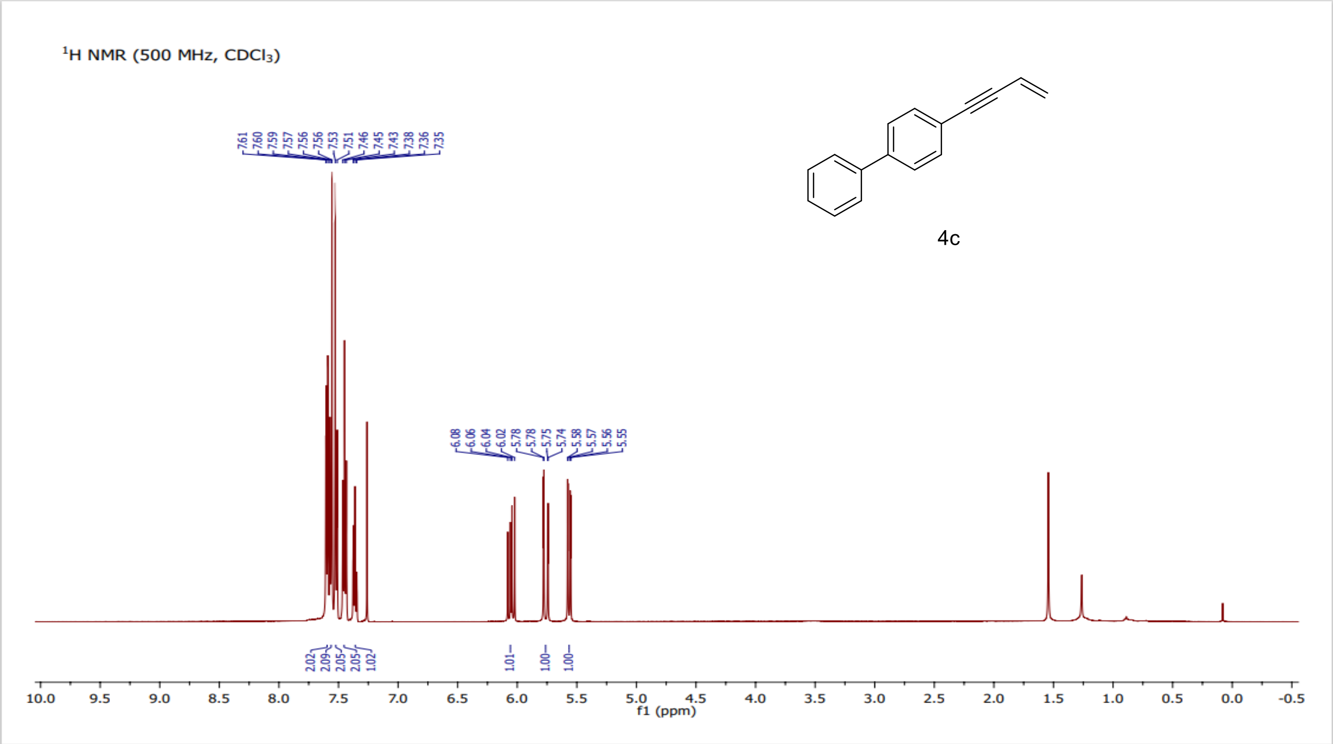

1H NMR (500 MHz, CDCl3)
7.61 7.60 7.59 7.57 7.56 7.56 7.53 7.51 7.46 7.45 7.43 7.38 7.36 7.35
6.08 6.06 6.04 6.02 5.78 5.78 5.75 5.74 5.58 5.57 5.56 5.55
2.02 2.09 2.05 2.05 1.02
1.01 1.00 1.00
4c
f1 (ppm)

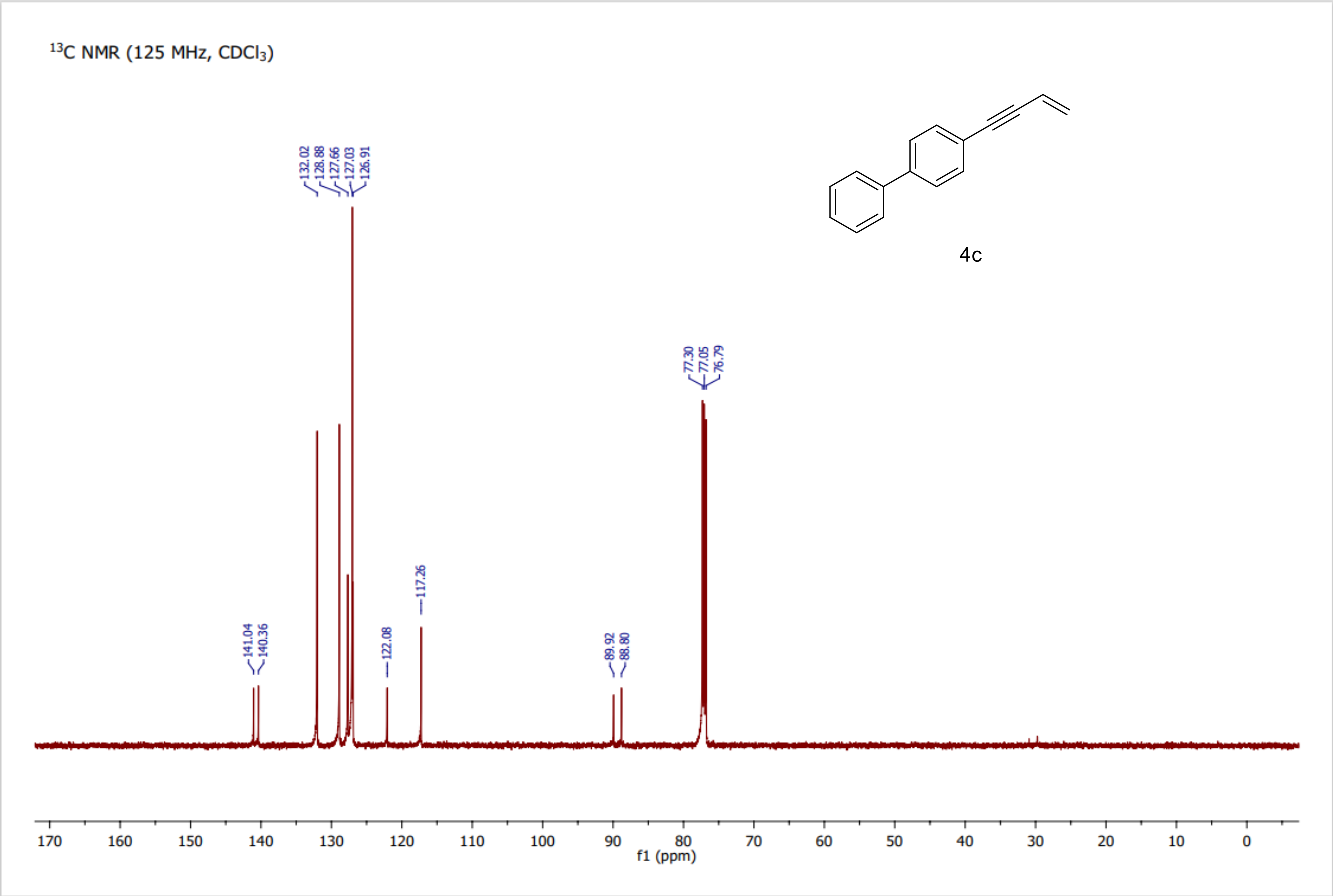

13C NMR (125 MHz, CDCl3)
132.02 128.88 127.66 127.03 126.91
77.30 77.05 76.79
141.04 140.36
122.08
117.26
89.92 88.80
4c
f1 (ppm)

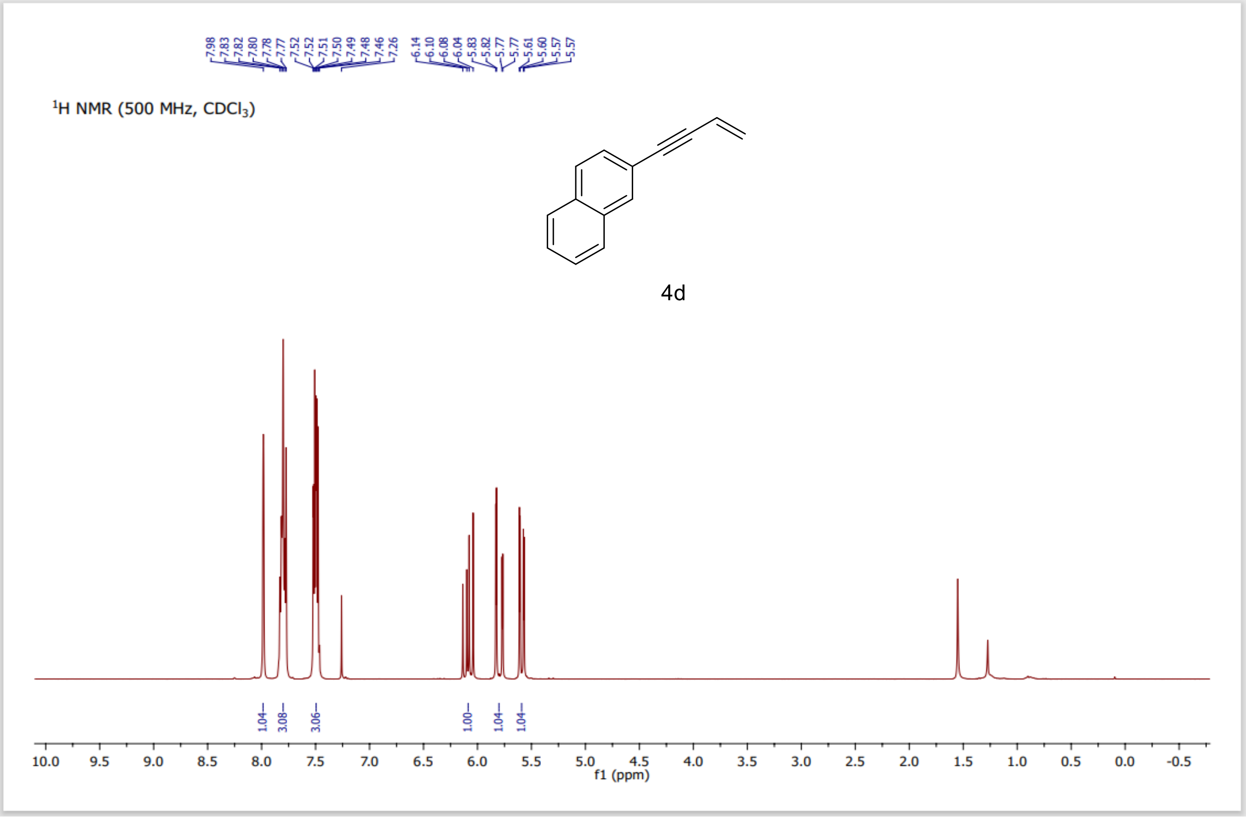

¹H NMR (500 MHz, CDCl₃)
4d
f1 (ppm)

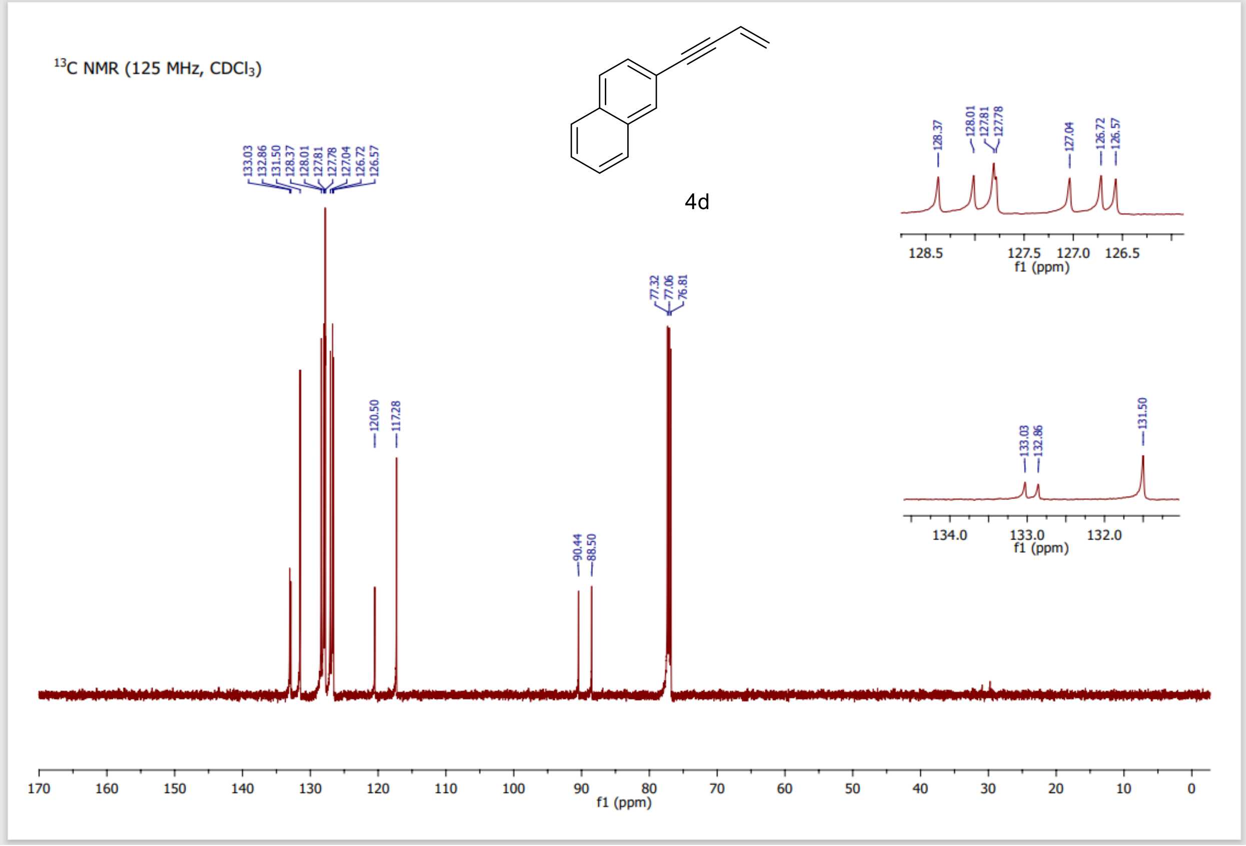

¹³C NMR (125 MHz, CDCl₃)
4d
f1 (ppm)

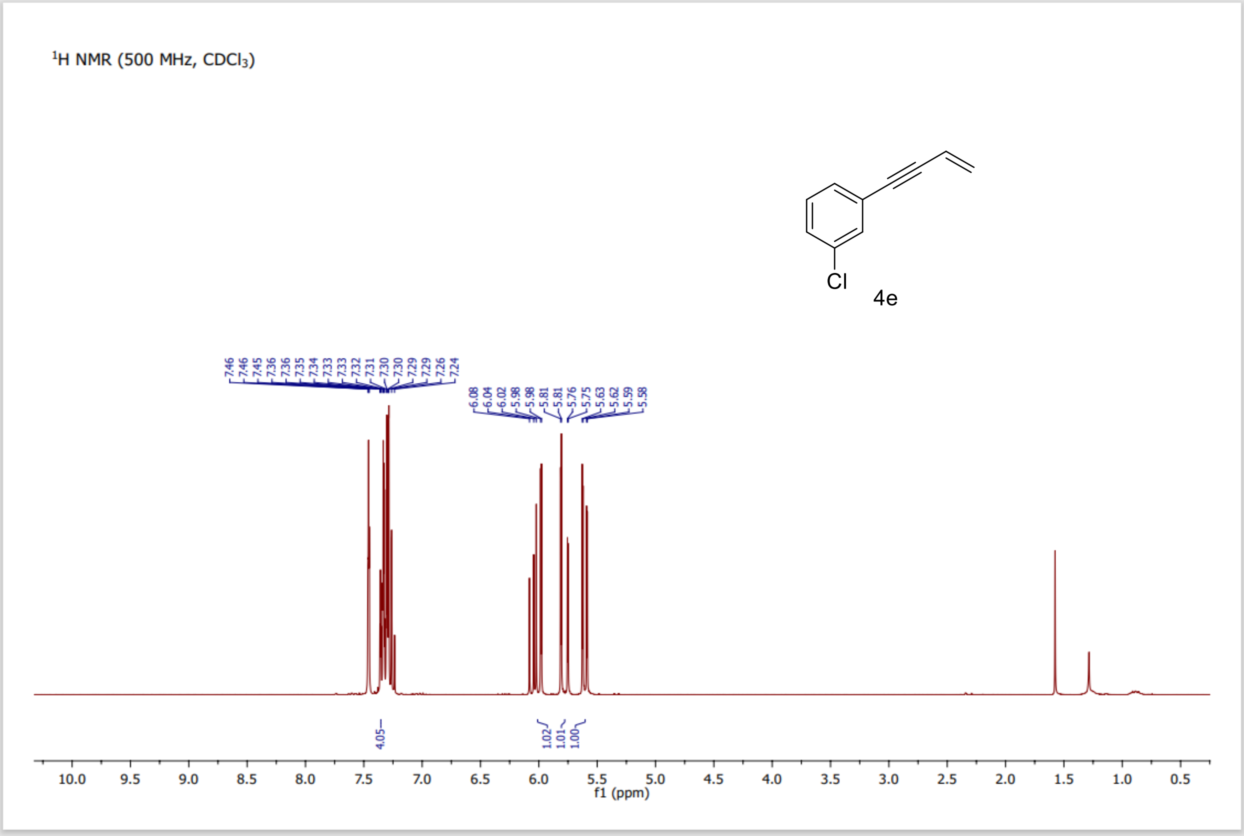
¹H NMR (500 MHz, CDCl₃)
7.46 7.46 7.45 7.36 7.36 7.35 7.34 7.33 7.33 7.32 7.31 7.30 7.30 7.29 7.29 7.26 7.24
6.08 6.04 6.02 5.98 5.98 5.81 5.81 5.76 5.75 5.63 5.62 5.59 5.58
4.05
1.02 1.01 1.00
Cl
4e
10.0 9.5 9.0 8.5 8.0 7.5 7.0 6.5 6.0 5.5 5.0 4.5 4.0 3.5 3.0 2.5 2.0 1.5 1.0 0.5
f1 (ppm)

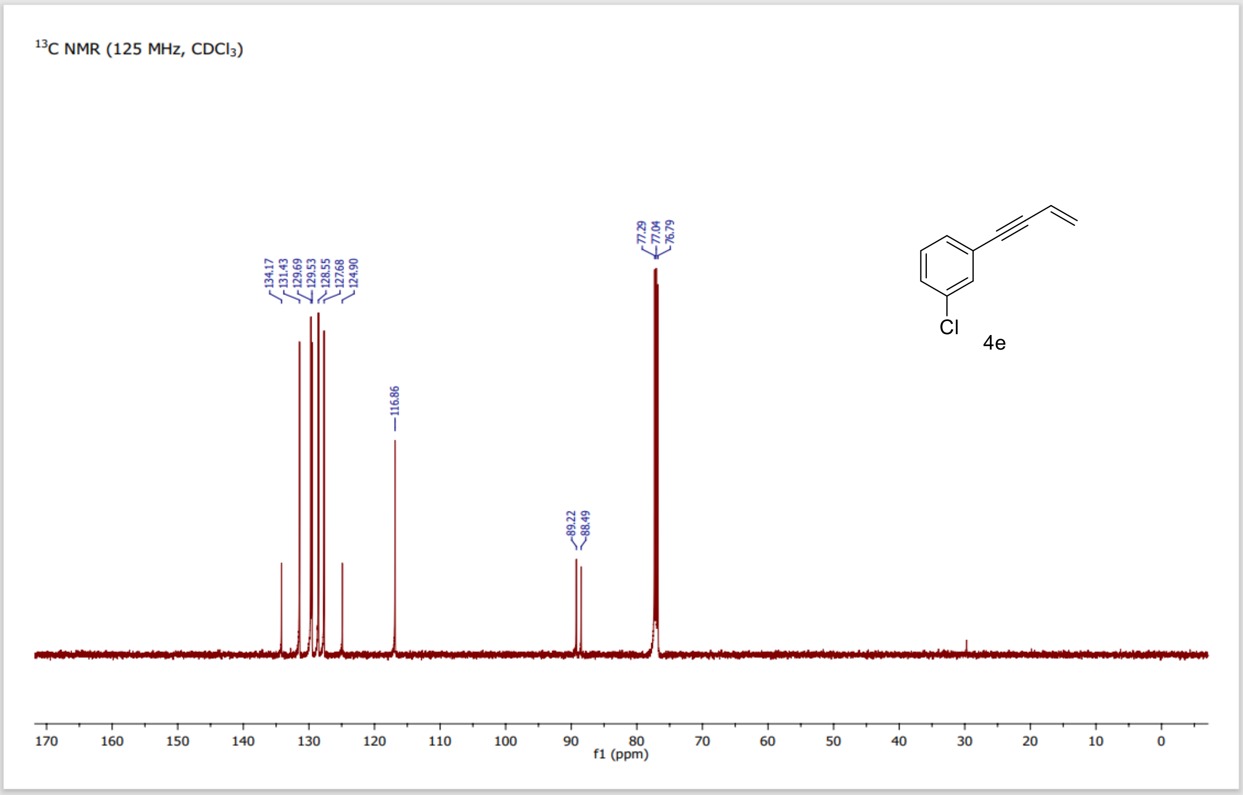
¹³C NMR (125 MHz, CDCl₃)
134.17 131.43 129.69 129.53 128.55 127.68 124.90
116.86
89.22 88.49
77.29 77.04 76.79
Cl
4e
170 160 150 140 130 120 110 100 90 80 70 60 50 40 30 20 10 0
f1 (ppm)

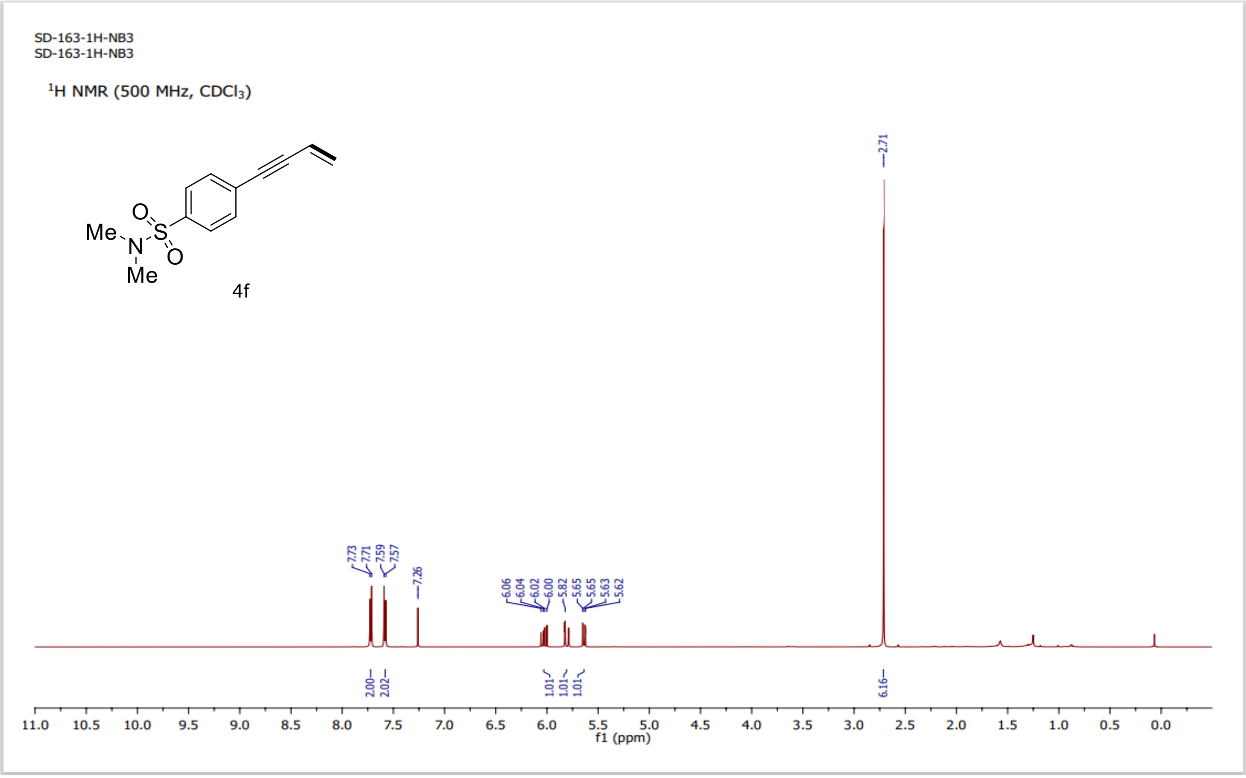
SD-163-1H-NB3
SD-163-1H-NB3
¹H NMR (500 MHz, CDCl3)
Me
N
S
O
O
Me
4f
7.73
7.71
7.59
7.57
7.26
6.06
6.04
6.02
6.00
5.82
5.65
5.65
5.63
5.62
2.71
2.00
2.02
1.01
1.01
1.01
6.16
f1 (ppm)

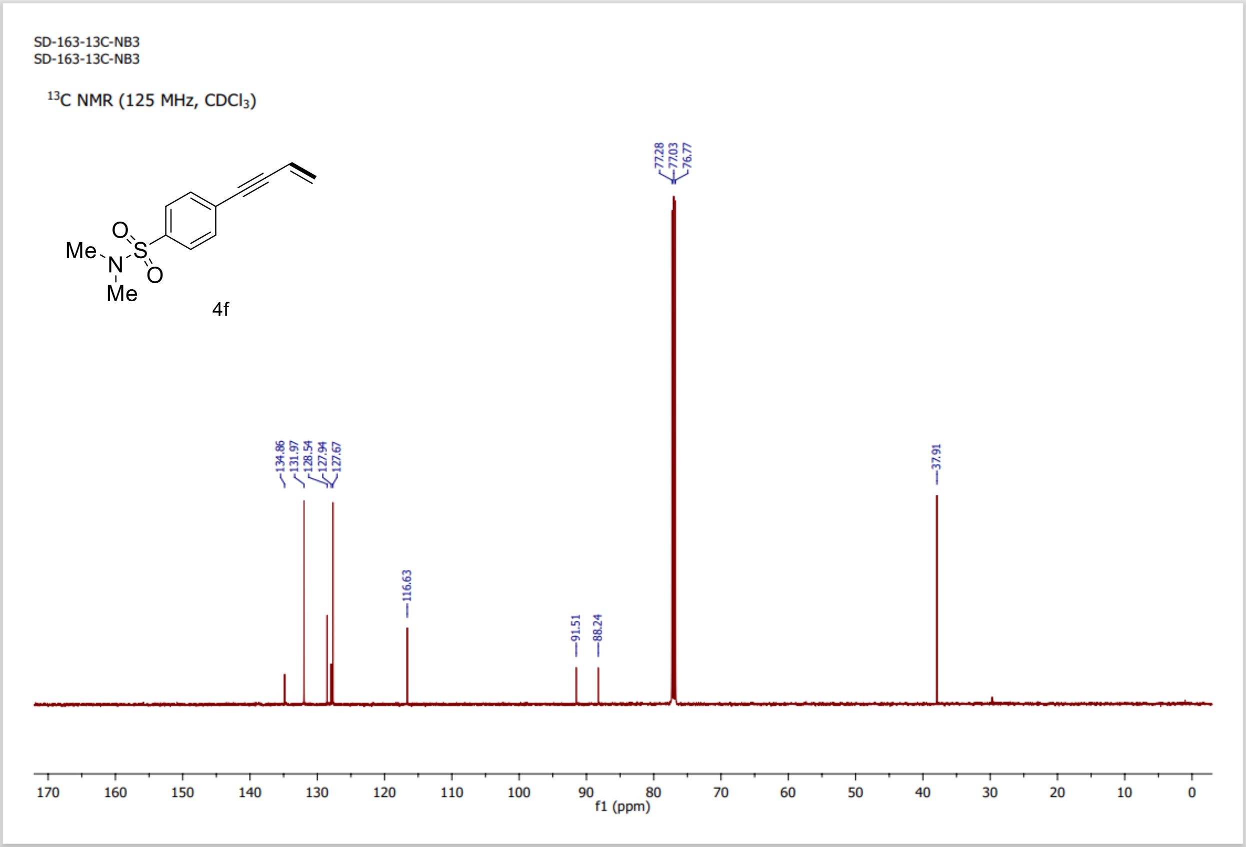
SD-163-13C-NB3
SD-163-13C-NB3
¹³C NMR (125 MHz, CDCl3)
Me
N
S
O
O
Me
4f
134.86
131.97
128.54
127.94
127.67
116.63
91.51
88.24
77.28
77.03
76.77
37.91
f1 (ppm)

^1H NMR (500 MHz, $CDCl_3$)

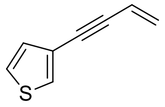

4g

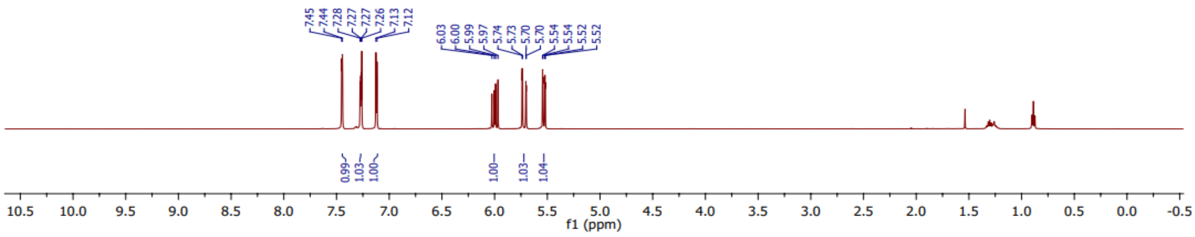

^{13}C NMR (125 MHz, $CDCl_3$)

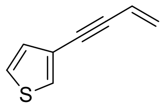

4g

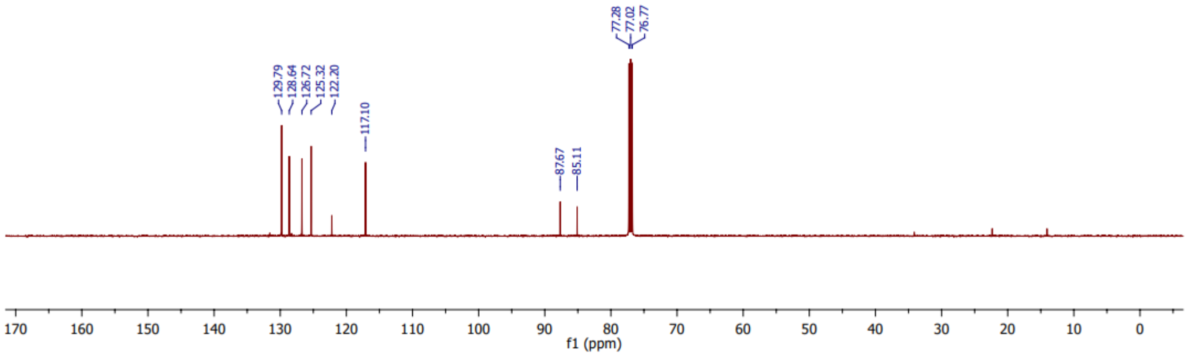

^{1}H NMR (500 MHz, $CDCl_3$)

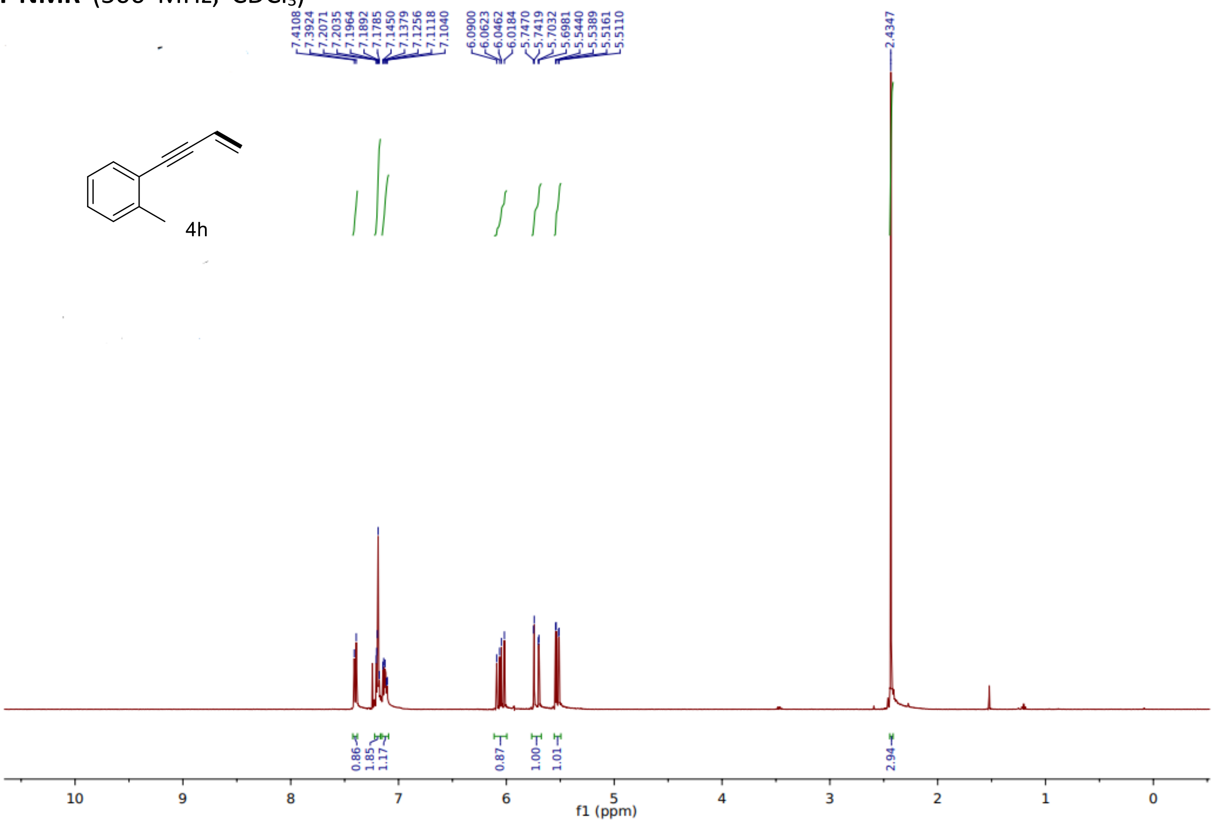

^{1}H NMR (500 MHz, $CDCl_3$)

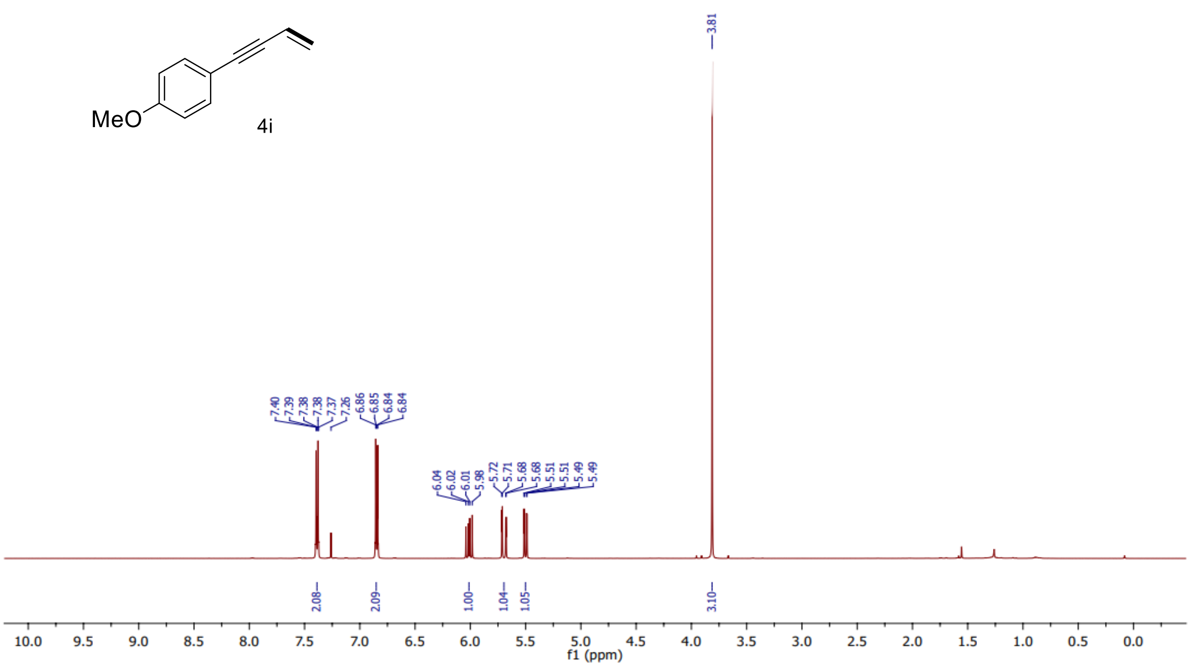

^{13}C NMR (125 MHz, $CDCl_3$)

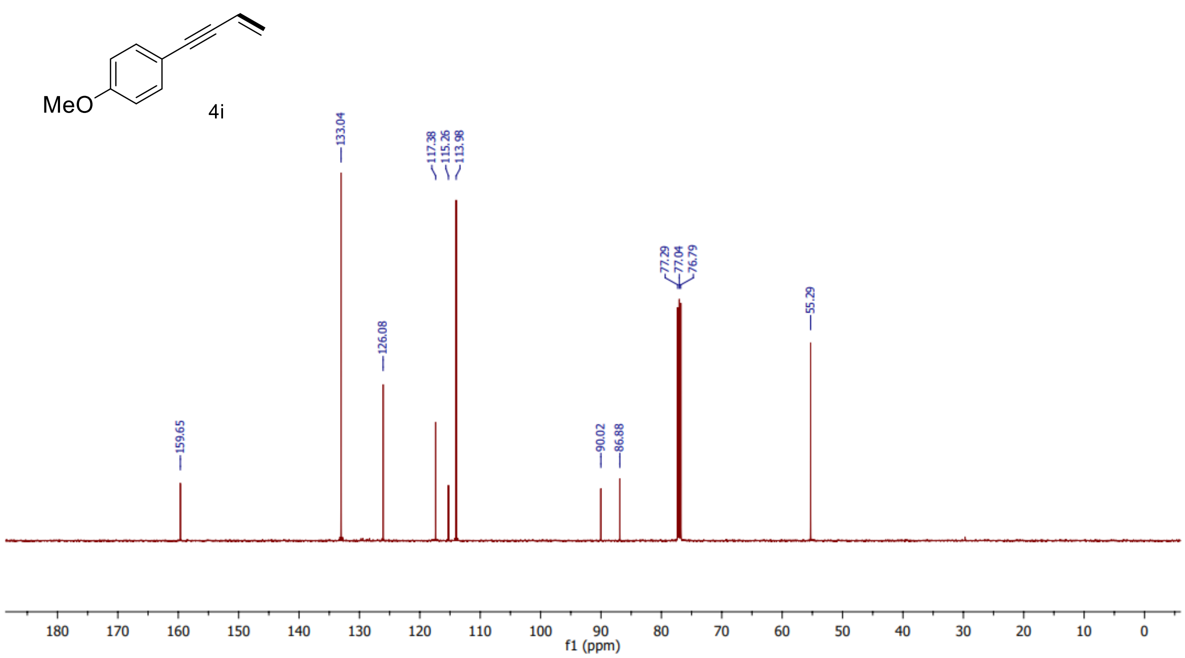

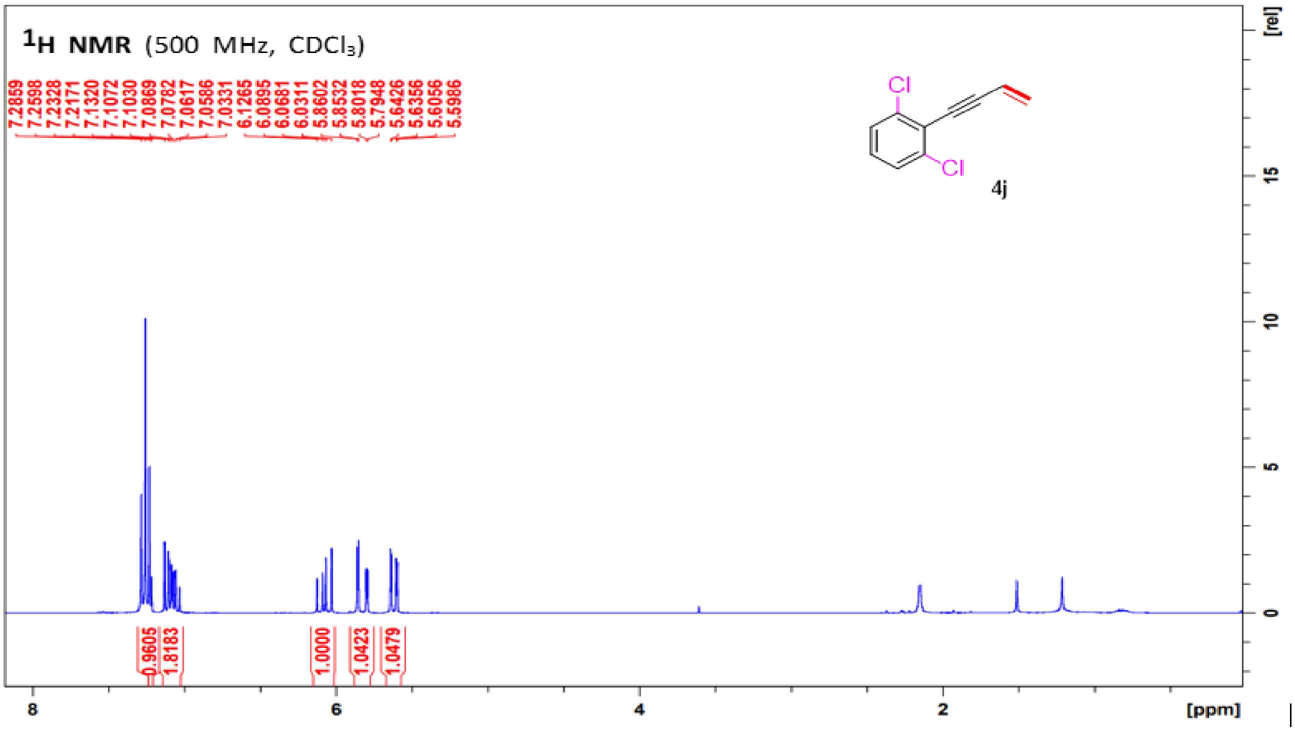
1H NMR (500 MHz, CDCl3)
7.2859
7.2598
7.2328
7.2171
7.1320
7.1072
7.1030
7.0869
7.0782
7.0617
7.0586
7.0331
6.1265
6.0895
6.0681
6.0311
5.8602
5.8532
5.8018
5.7948
5.6426
5.6356
5.6056
5.5986
Cl
Cl
4j
0.9605
1.8183
1.0000
1.0423
1.0479
[ppm]
[rel]

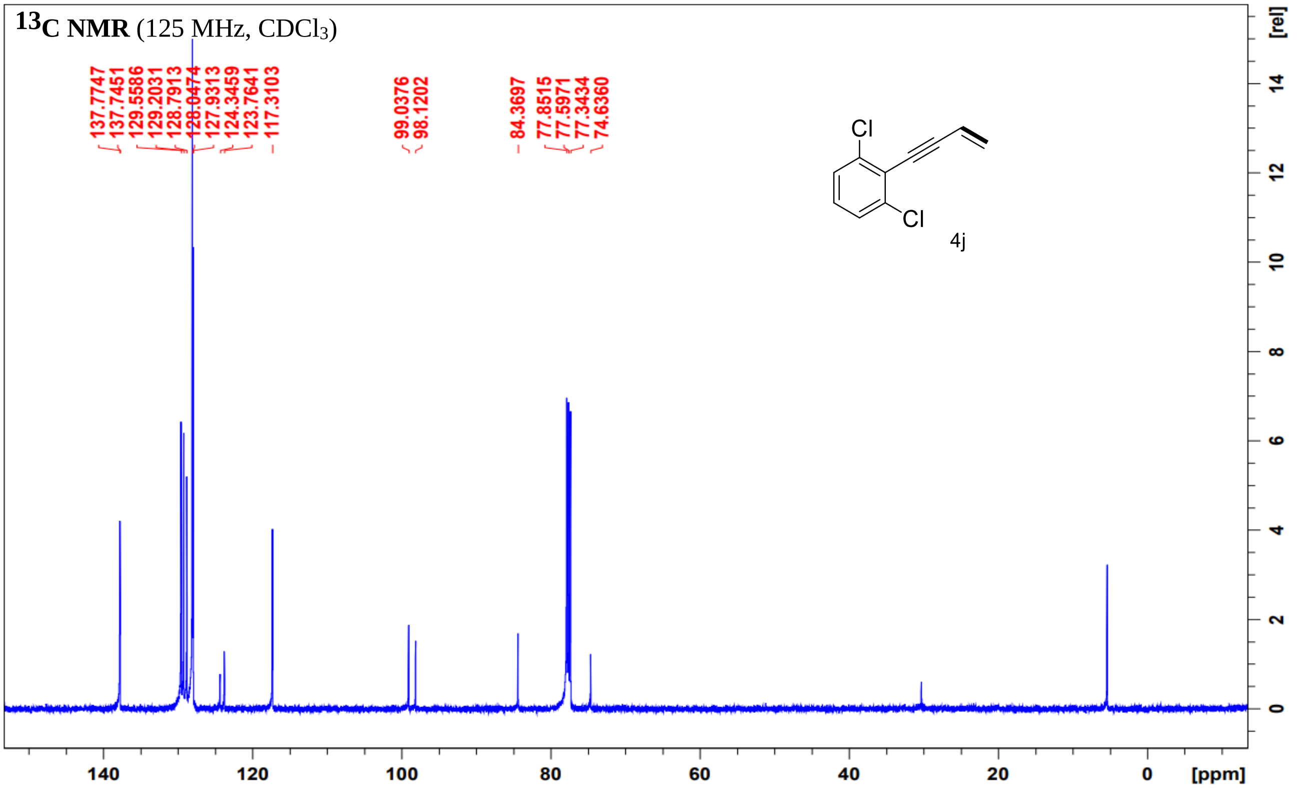
13C NMR (125 MHz, CDCl3)
137.7747
137.7451
129.5586
129.2031
128.7913
128.0474
127.9313
124.3459
123.7641
117.3103
99.0376
98.1202
84.3697
77.8515
77.5971
77.3434
74.6360
Cl
Cl
4j
[ppm]
[rel]

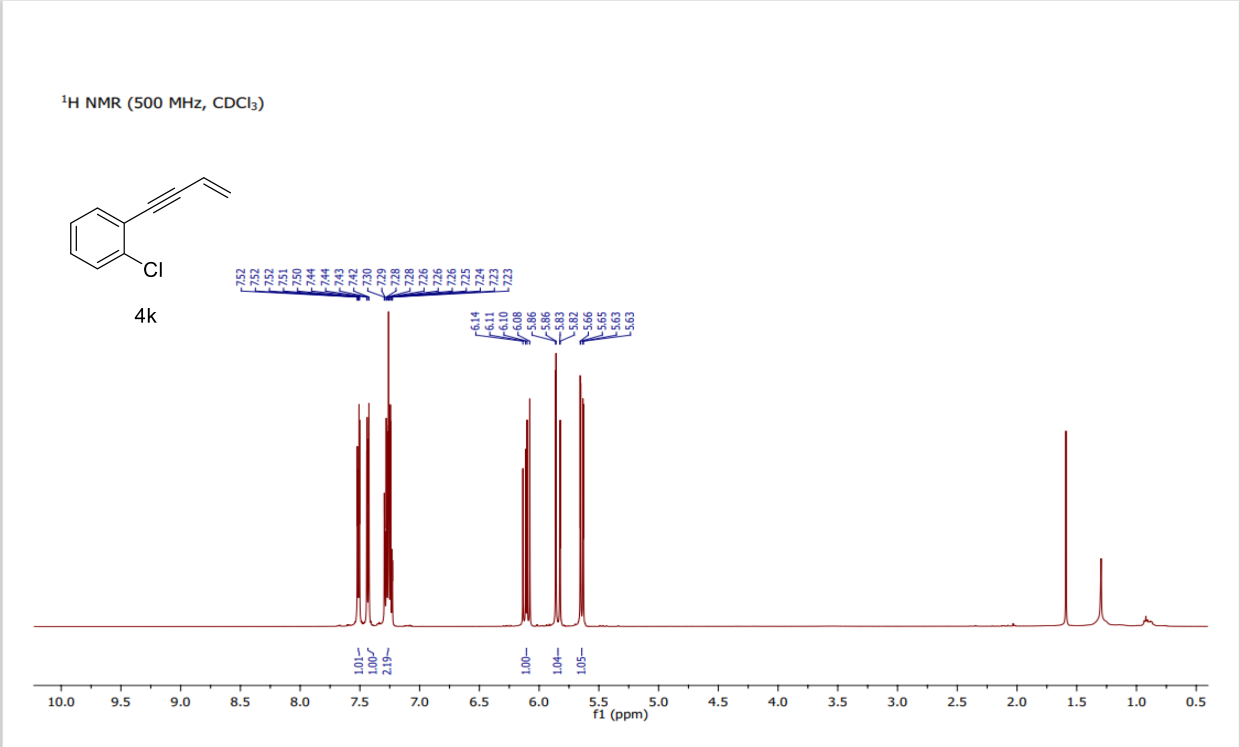

¹H NMR (500 MHz, CDCl3)
Cl
4k
7.52 7.52 7.52 7.51 7.50 7.44 7.44 7.43 7.42 7.30 7.29 7.28 7.28 7.26 7.26 7.26 7.25 7.24 7.23 7.23
6.14 6.11 6.10 6.08 5.86 5.86 5.83 5.82 5.66 5.65 5.63 5.63
1.01 1.00 2.19 1.00 1.04 1.05
10.0 9.5 9.0 8.5 8.0 7.5 7.0 6.5 6.0 5.5 5.0 4.5 4.0 3.5 3.0 2.5 2.0 1.5 1.0 0.5
f1 (ppm)

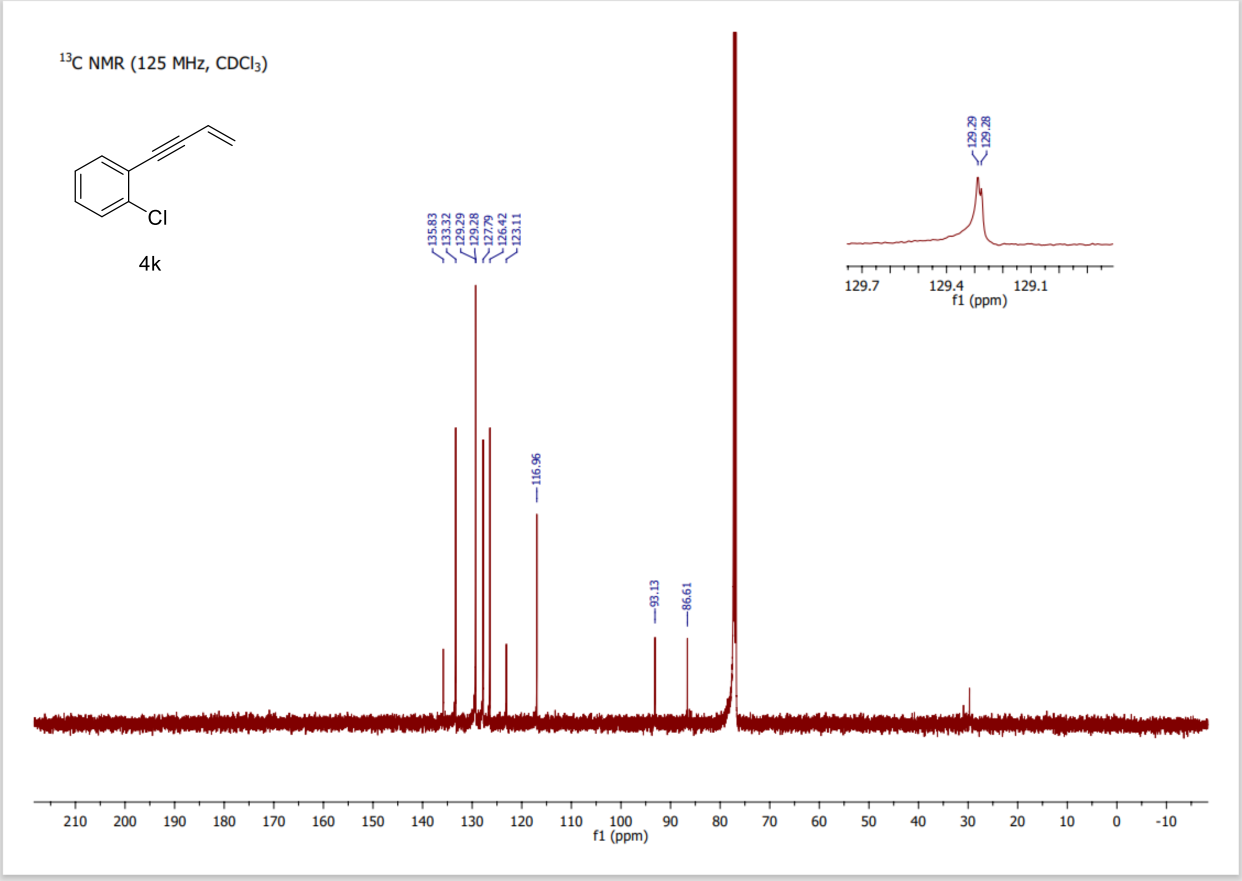

¹³C NMR (125 MHz, CDCl3)
Cl
4k
135.83 133.32 129.29 129.28 127.79 126.42 123.11
116.96
93.13
86.61
129.29 129.28
129.7 129.4 129.1
f1 (ppm)
210 200 190 180 170 160 150 140 130 120 110 100 90 80 70 60 50 40 30 20 10 0 -10
f1 (ppm)

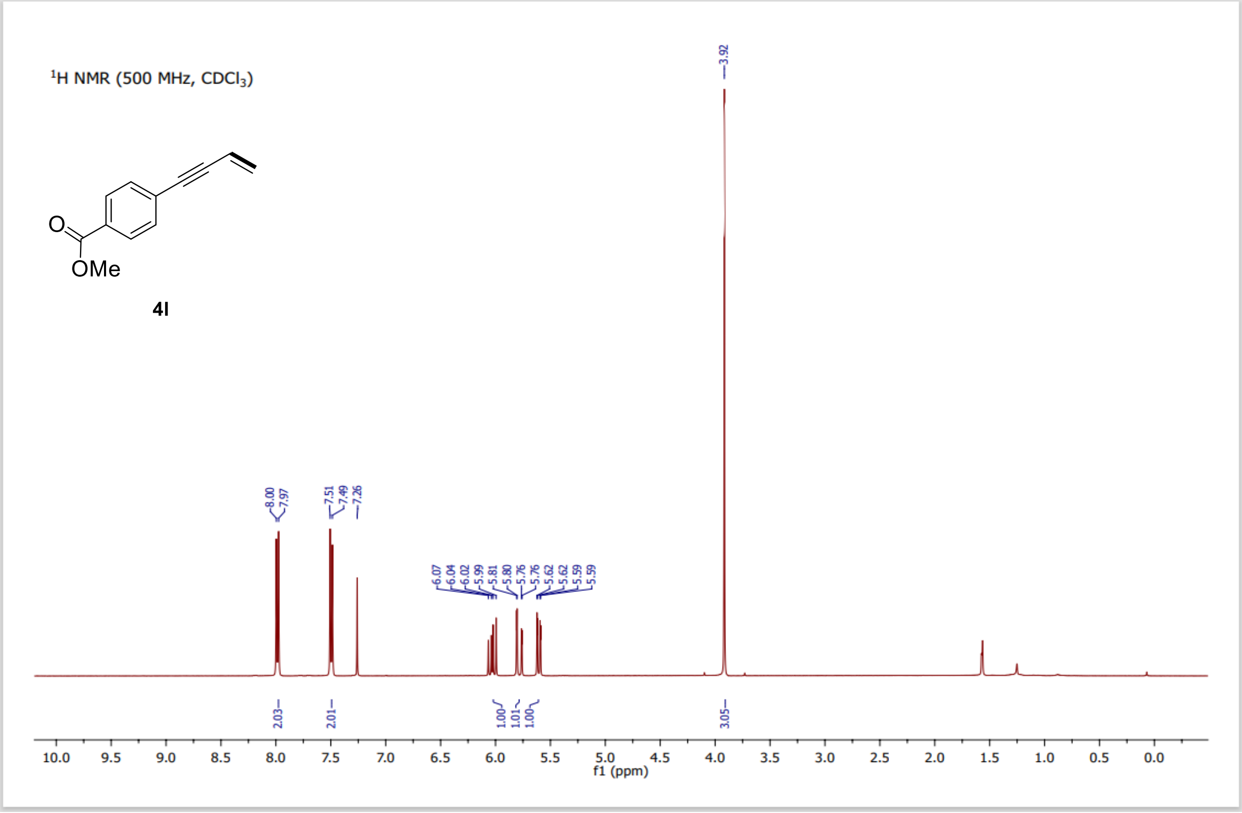

1H NMR (500 MHz, CDCl3)
OMe
4l
8.00
7.97
7.51
7.49
7.26
6.07
6.04
6.02
5.99
5.81
5.80
5.76
5.76
5.62
5.62
5.59
5.59
3.92
2.03
2.01
1.00
1.01
1.00
3.05
10.0 9.5 9.0 8.5 8.0 7.5 7.0 6.5 6.0 5.5 5.0 4.5 4.0 3.5 3.0 2.5 2.0 1.5 1.0 0.5 0.0
f1 (ppm)

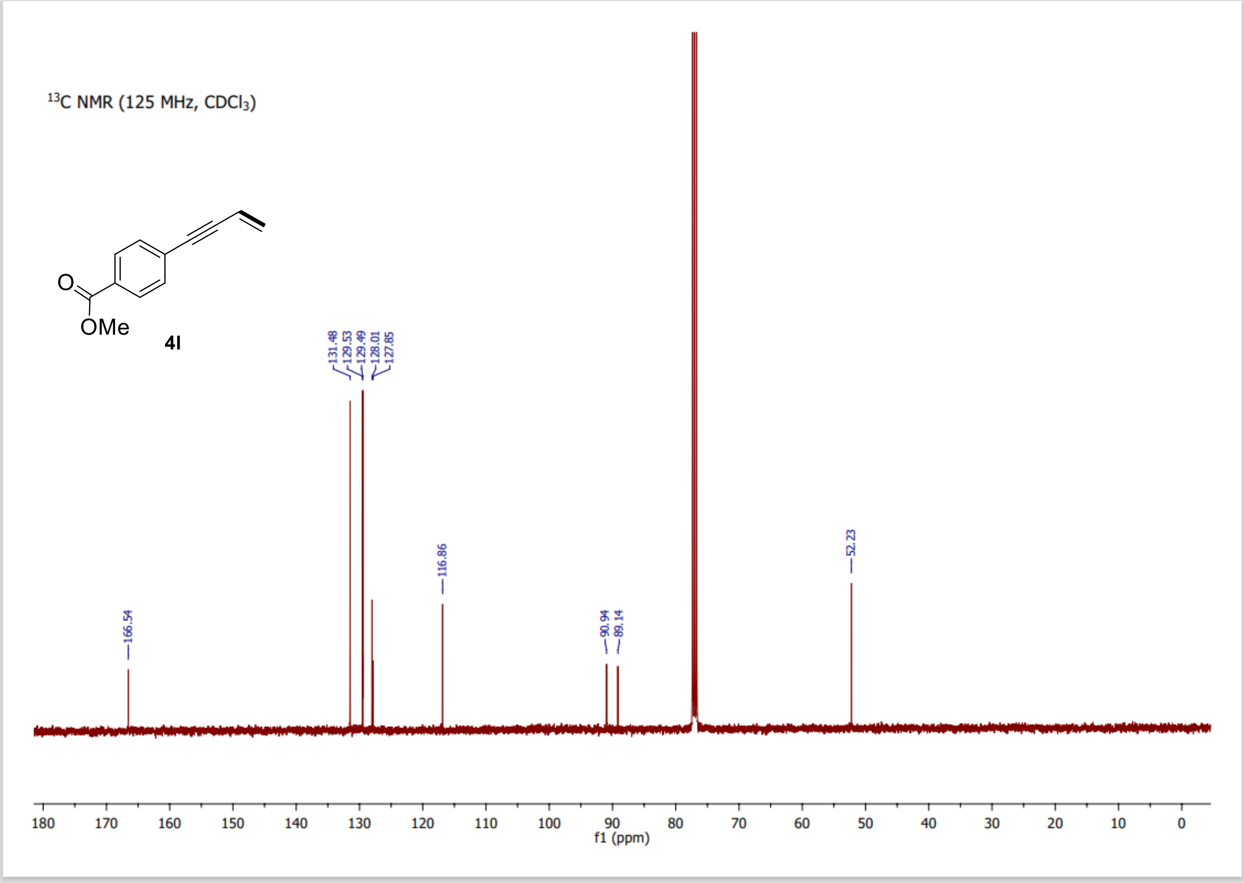

13C NMR (125 MHz, CDCl3)
OMe
4l
131.48
129.53
129.49
128.01
127.85
116.86
166.54
90.94
89.14
52.23
180 170 160 150 140 130 120 110 100 90 80 70 60 50 40 30 20 10 0
f1 (ppm)

1H NMR (500 MHz, $CDCl_3$)

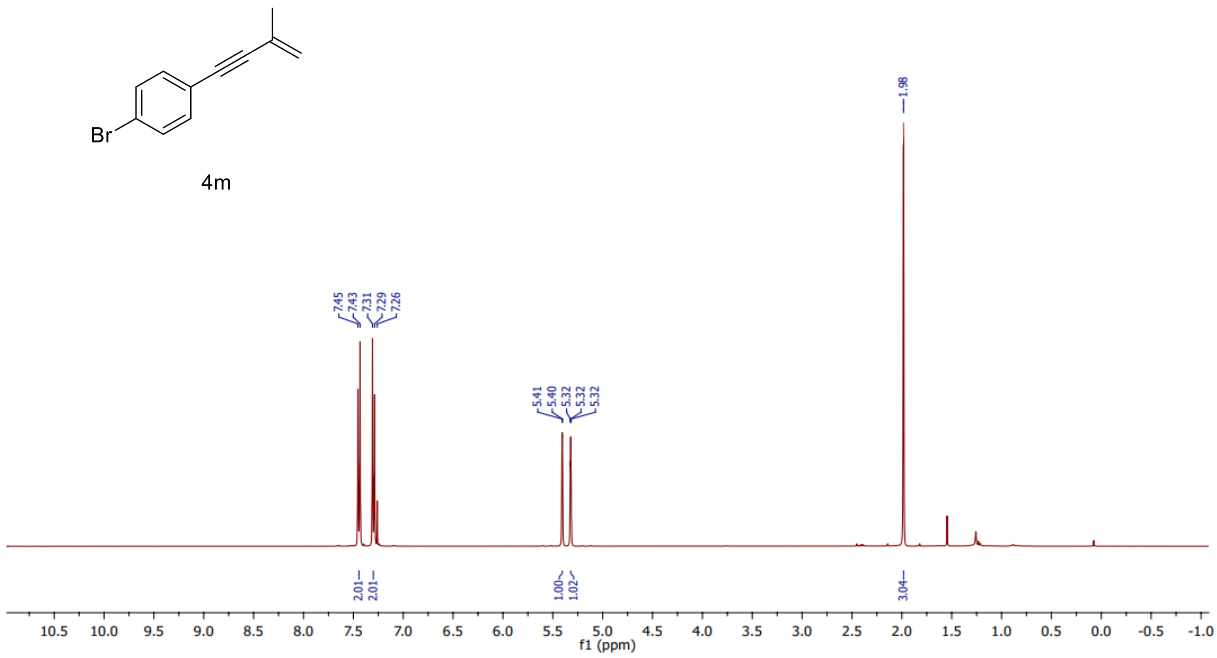

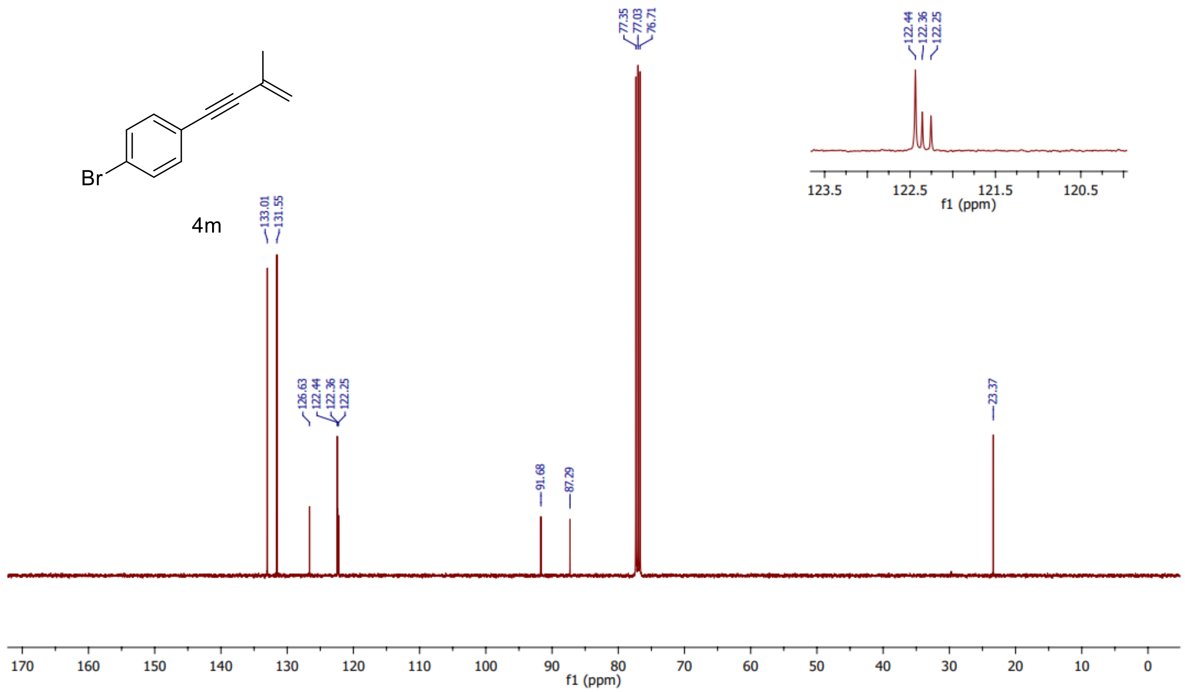

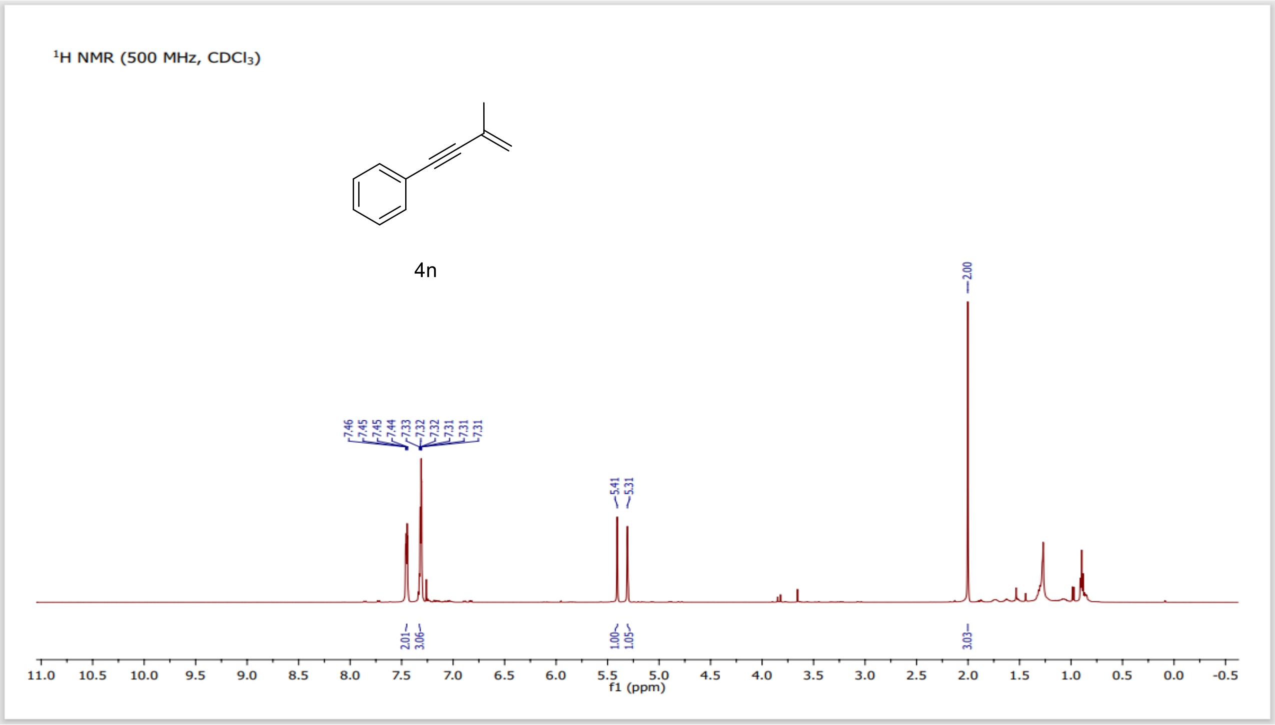
^{1}H NMR (500 MHz, CDCl$_3$)
4n
7.46 7.45 7.45 7.44 7.33 7.32 7.32 7.31 7.31 7.31
5.41 5.31
2.00
2.01 3.06 1.00 1.05 3.03
f1 (ppm)

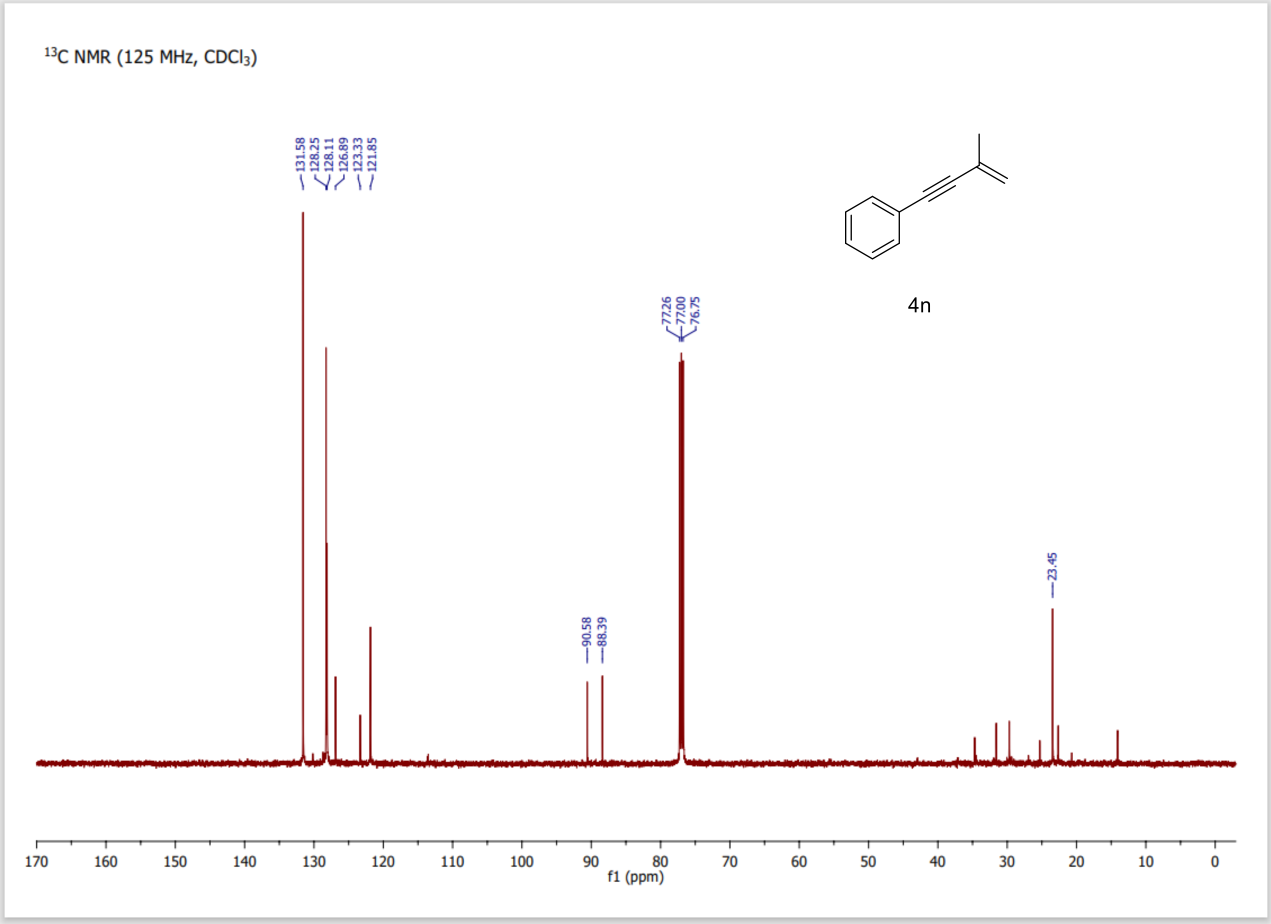
^{13}C NMR (125 MHz, CDCl$_3$)
131.58 128.25 128.11 126.89 123.33 121.85
4n
77.26 77.00 76.75
90.58 88.39
23.45
f1 (ppm)

1H NMR (500 MHz, $CDCl_3$)

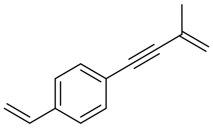

4o

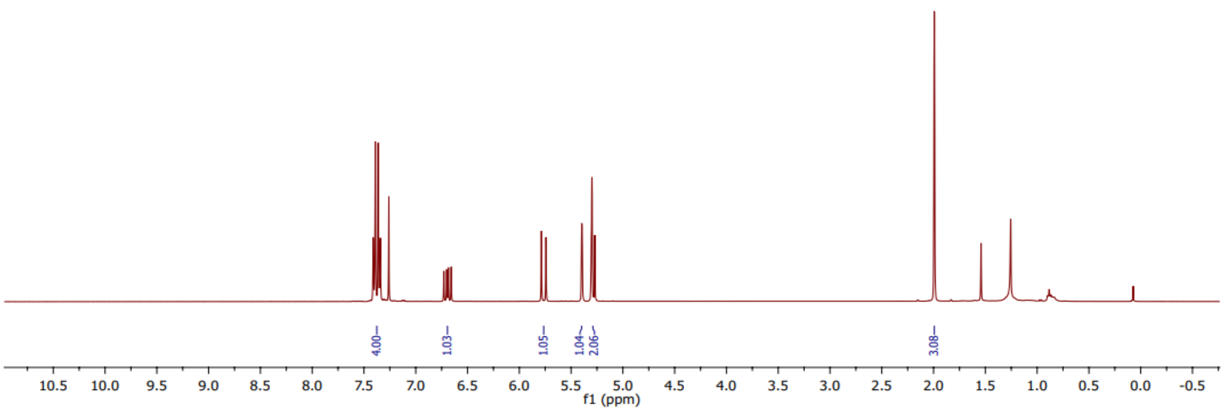

13C NMR (125 MHz, $CDCl_3$)

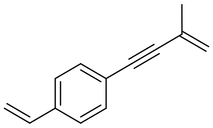

4o

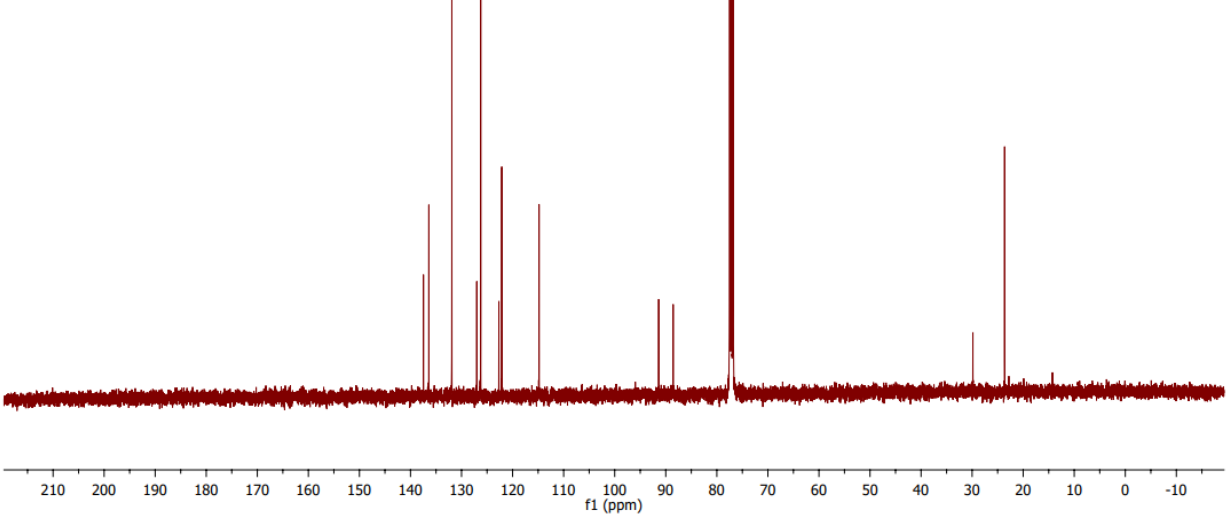

^{1}H NMR (500 MHz, $CDCl_3$)

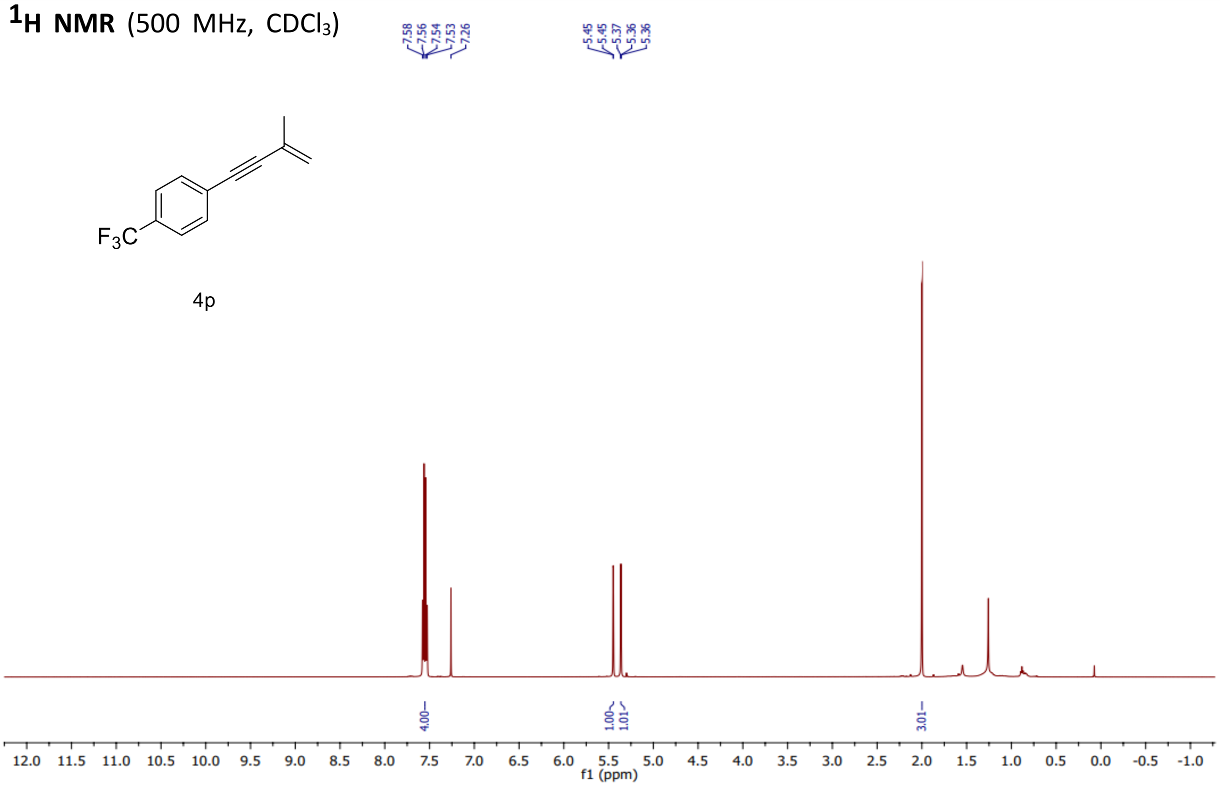

^{13}C NMR (125 MHz, $CDCl_3$)

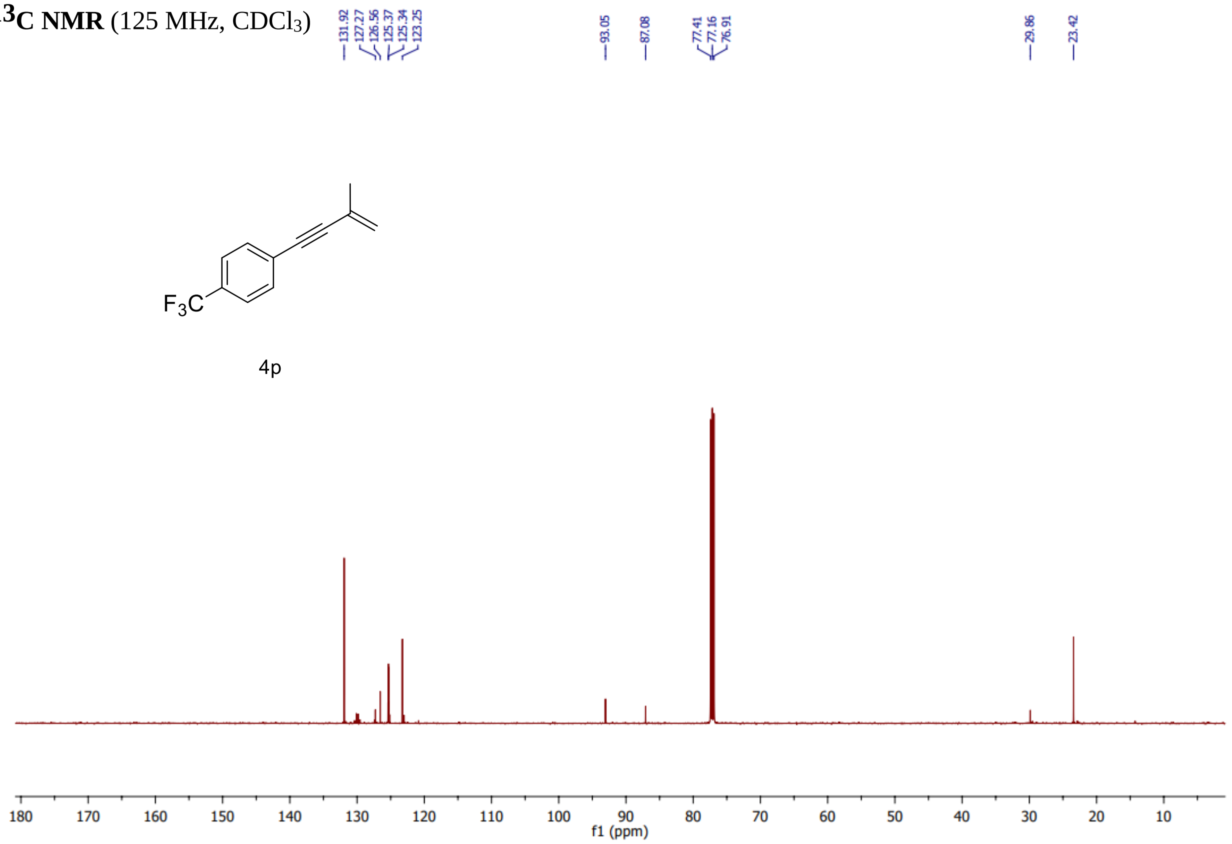

^{19}F NMR (470 MHz, $CDCl_3$)

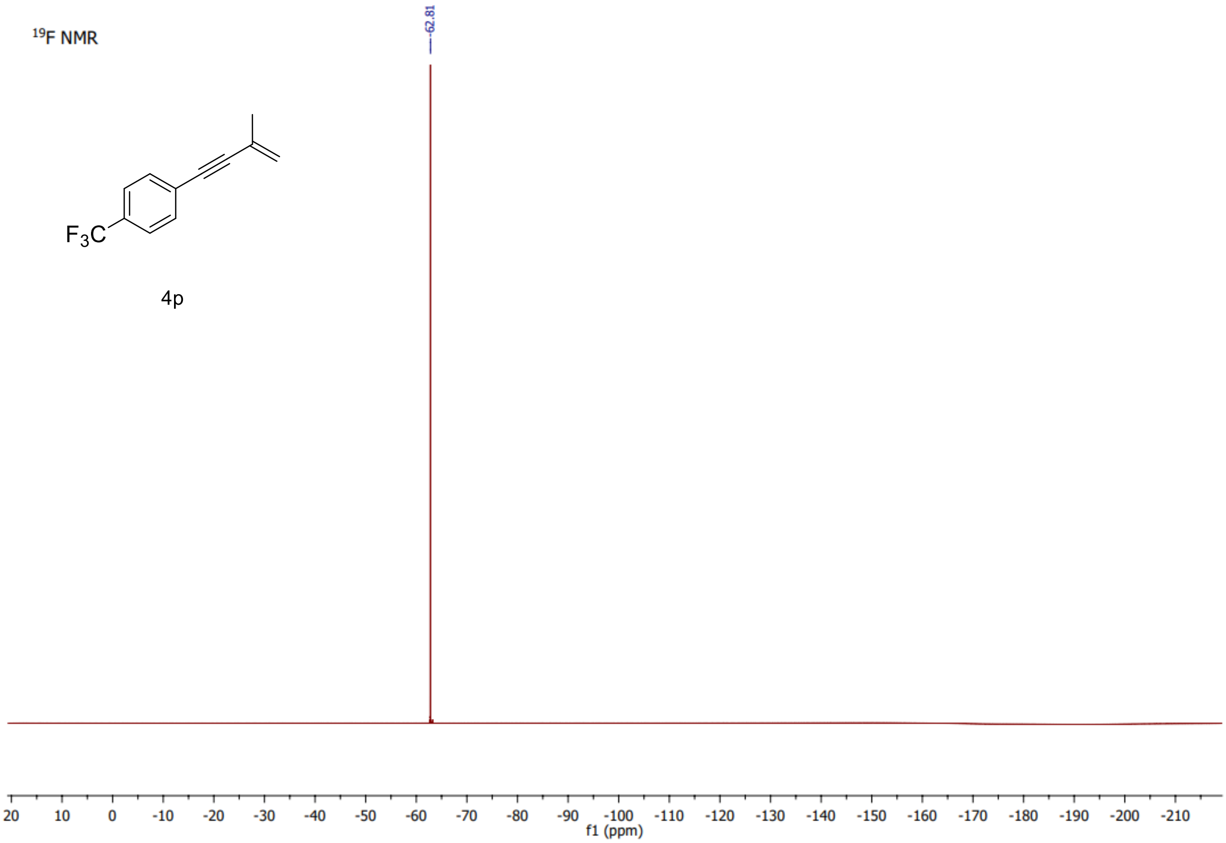

^{1}H NMR (500 MHz, $CDCl_3$)

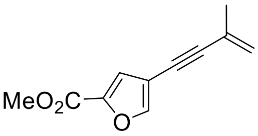

4q

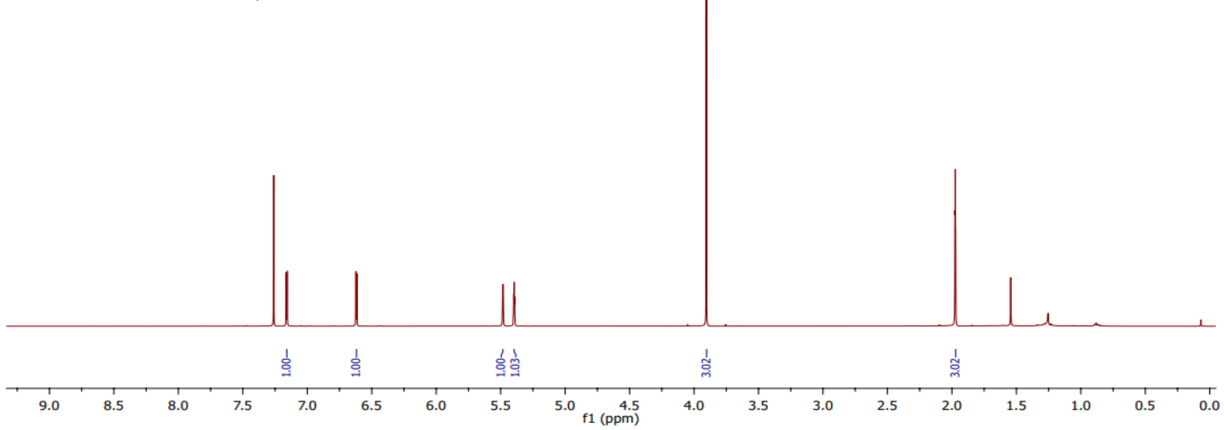

^{13}C NMR (125 MHz, $CDCl_3$)

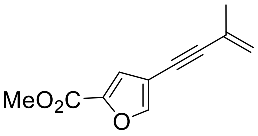

4q

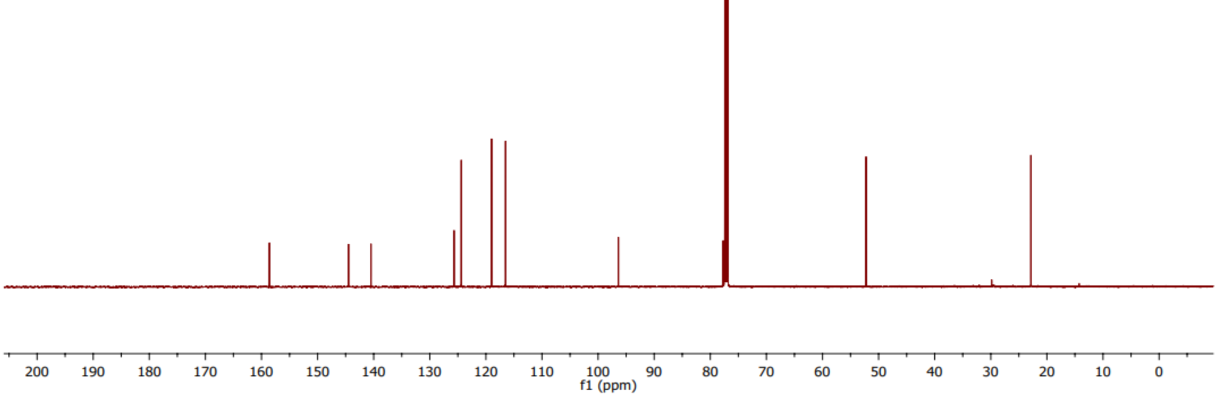

^{1}H NMR (500 MHz, $CDCl_3$)

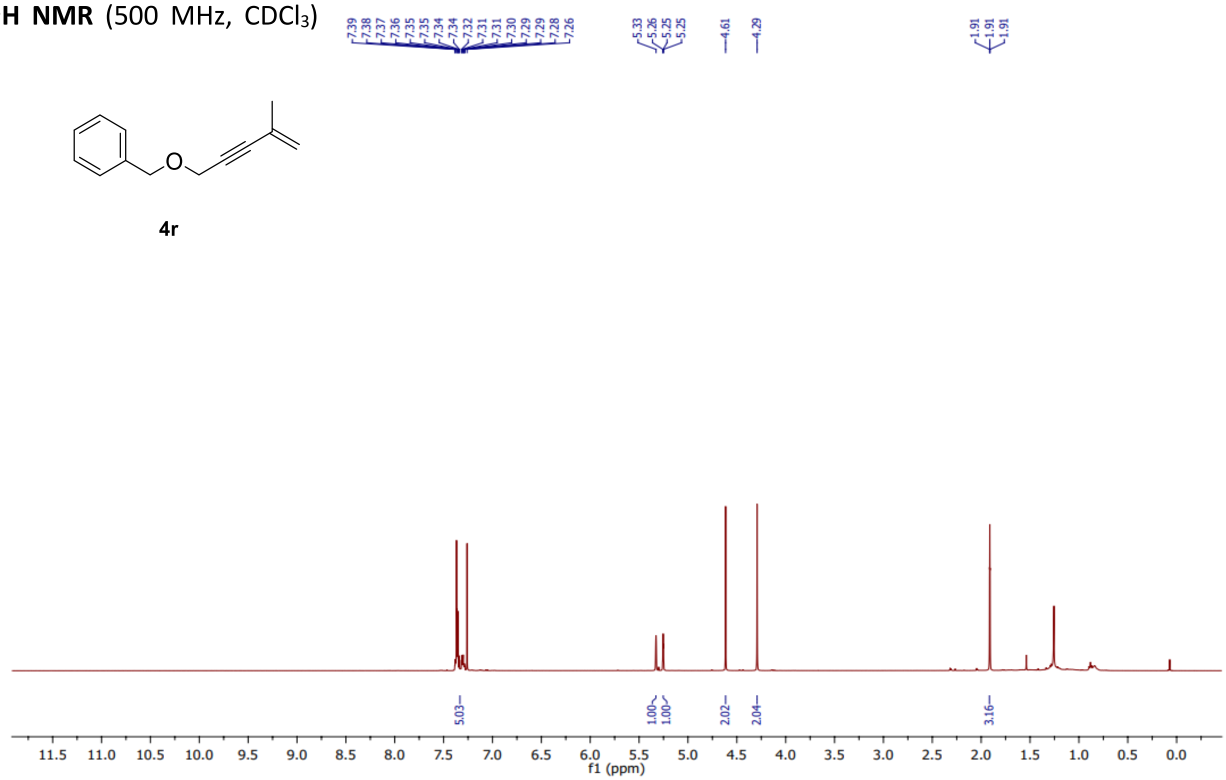

4r

^{13}C NMR (125 MHz, $CDCl_3$)

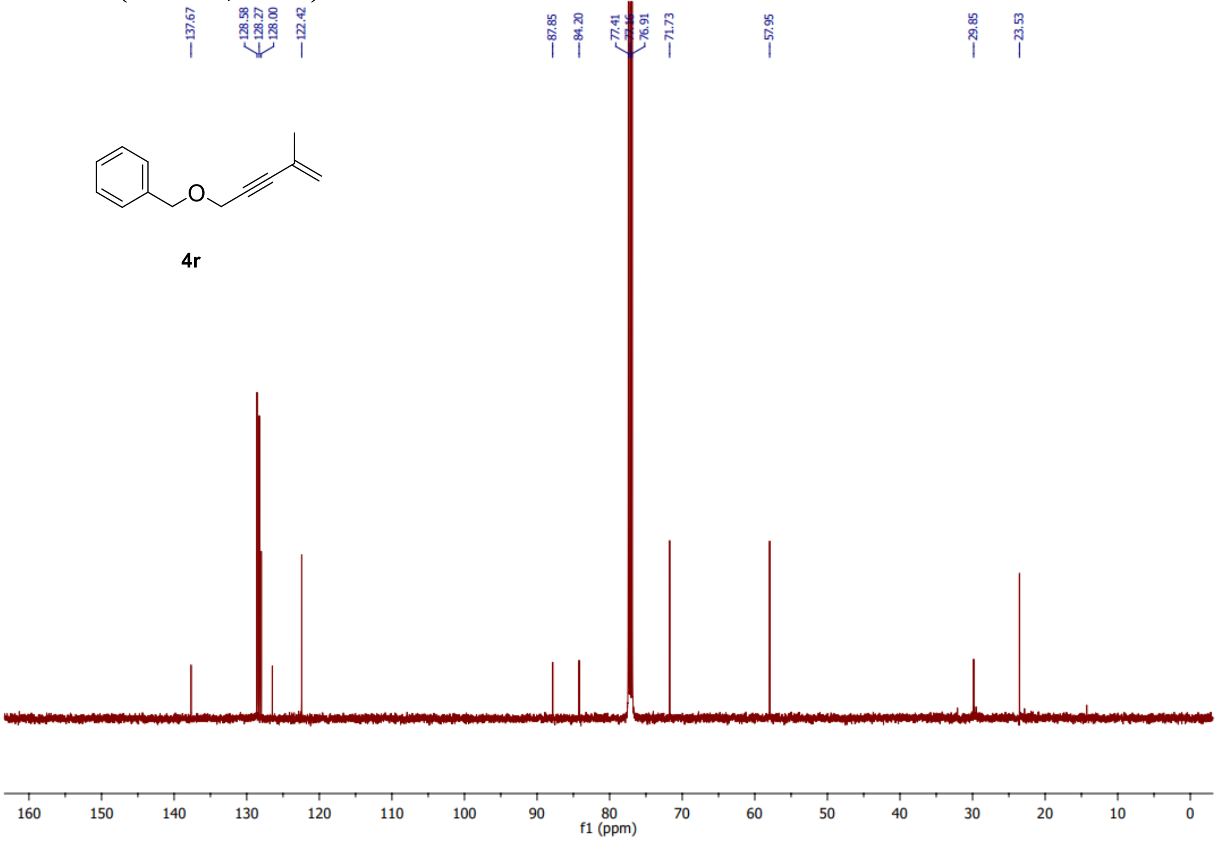

4r

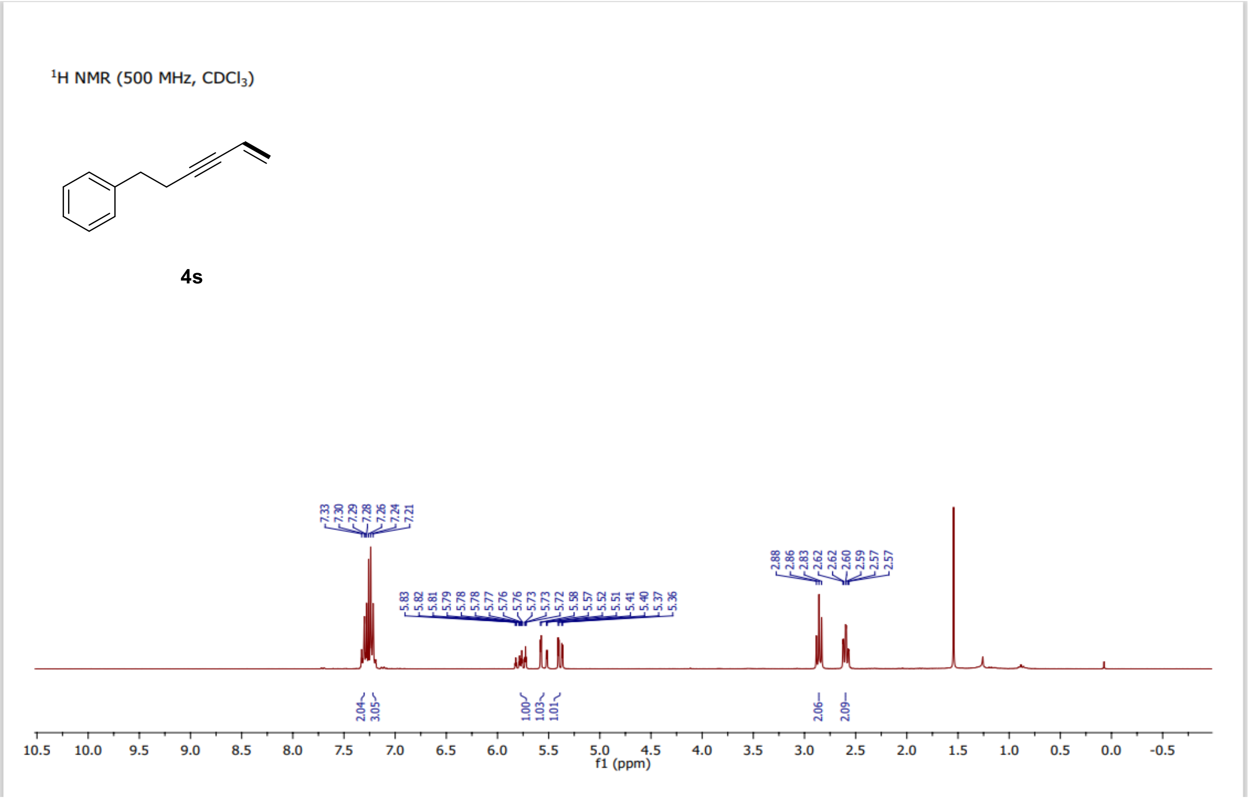

^{1}H NMR (500 MHz, CDCl$_3$)
4s
f1 (ppm)

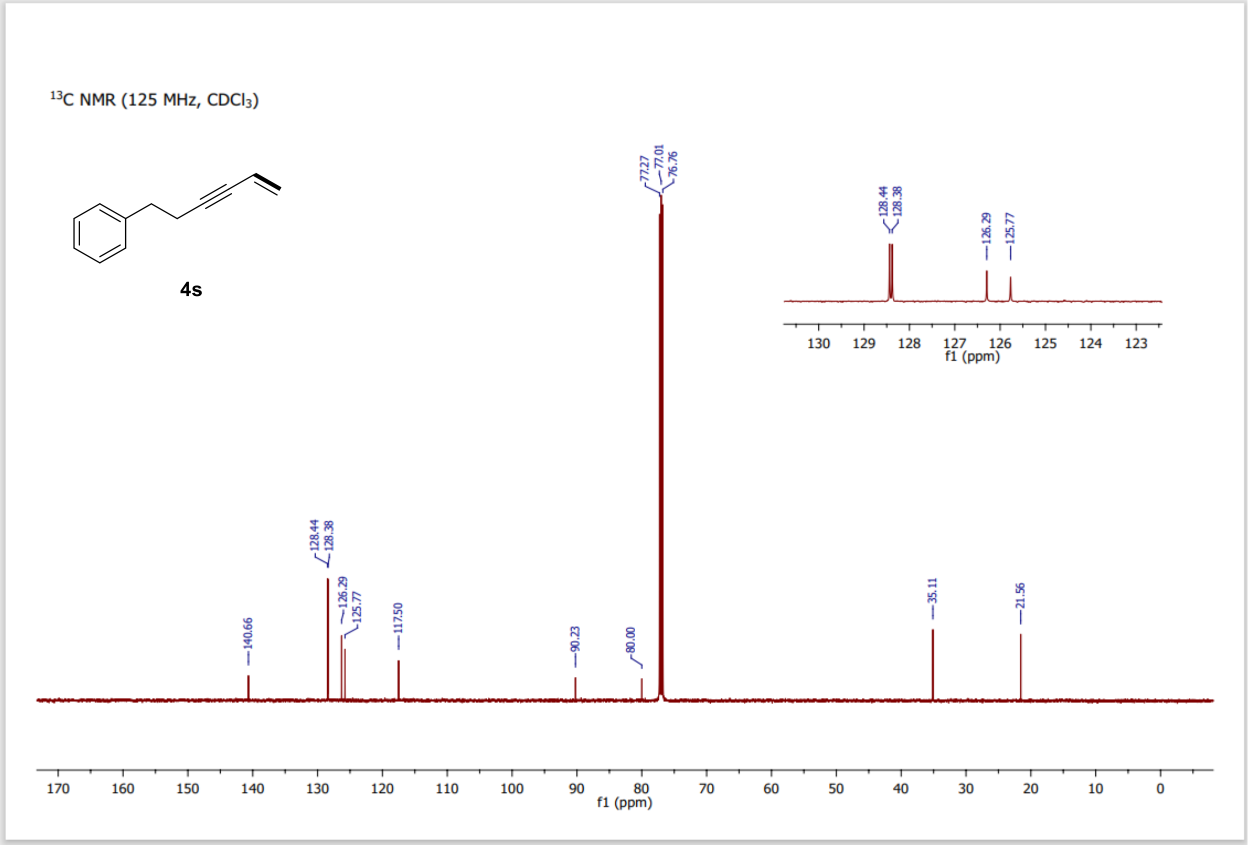

^{13}C NMR (125 MHz, CDCl$_3$)
4s
140.66
128.44
128.38
126.29
125.77
117.50
90.23
80.00
77.27
77.01
76.76
35.11
21.56
f1 (ppm)

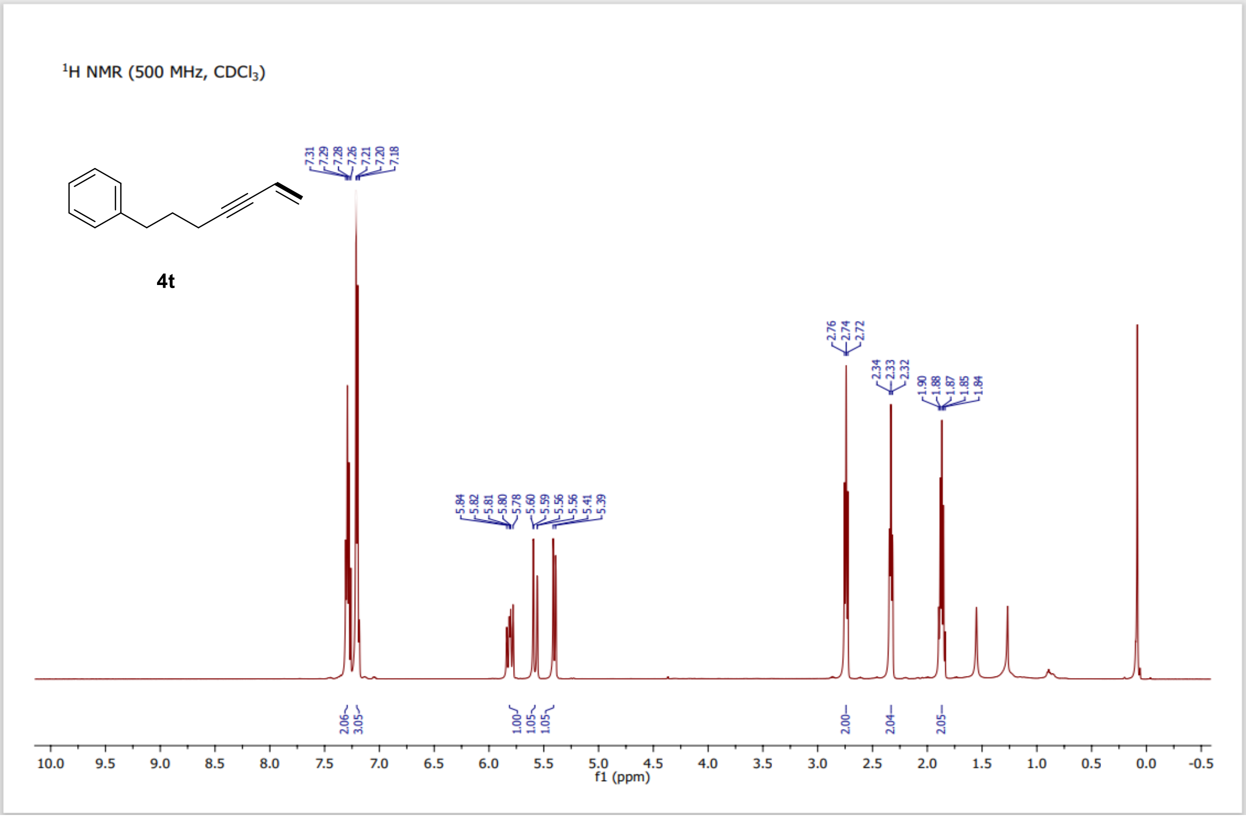
^{1}H NMR (500 MHz, CDCl$_3$)
4t
7.31 7.29 7.28 7.26 7.21 7.20 7.18
5.84 5.82 5.81 5.80 5.78 5.60 5.59 5.56 5.56 5.41 5.39
2.76 2.74 2.72
2.34 2.33 2.32
1.90 1.88 1.87 1.85 1.84
2.06 3.05 1.00 1.05 1.05 2.00 2.04 2.05
f1 (ppm)

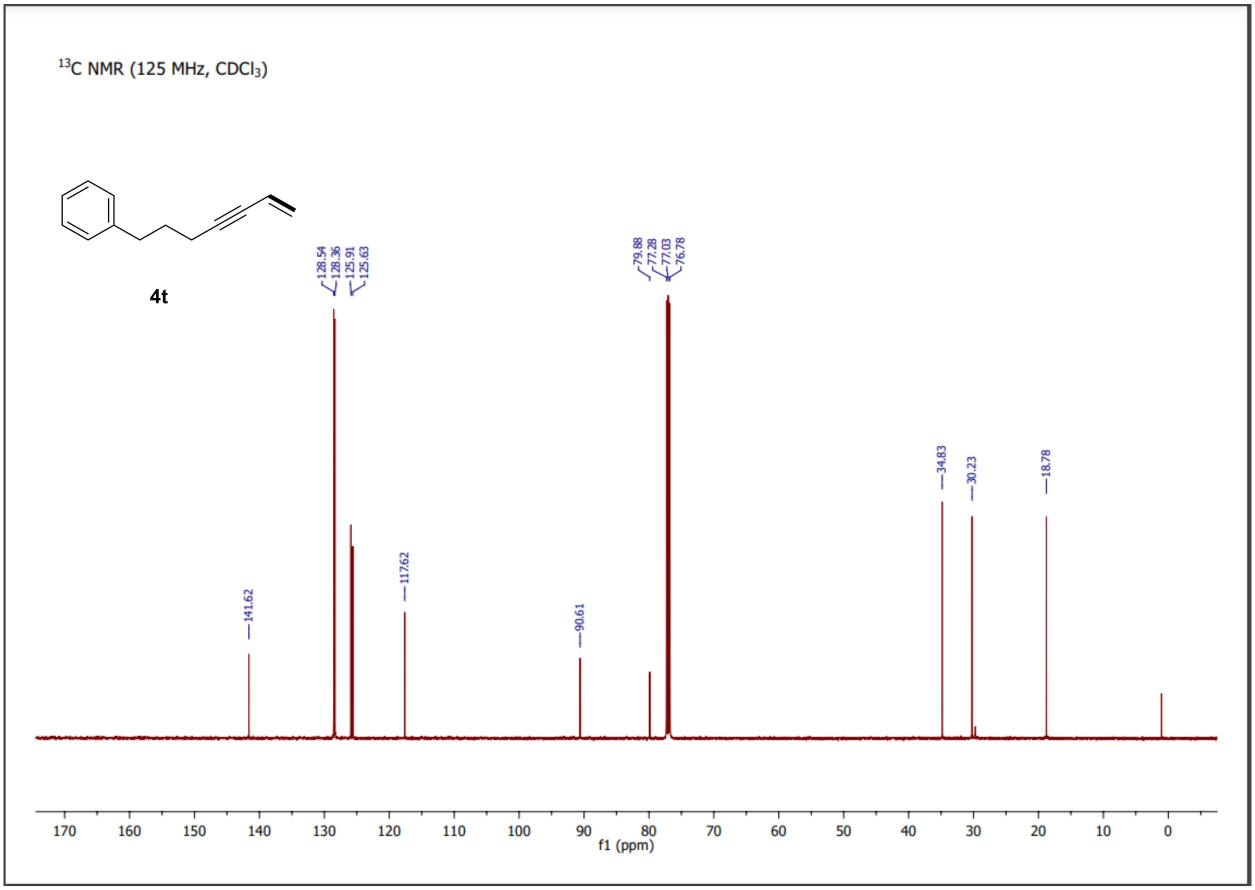
^{13}C NMR (125 MHz, CDCl$_3$)
4t
141.62
128.54 128.36 125.91 125.63
117.62
90.61
79.88 77.28 77.03 76.78
34.83
30.23
18.78
f1 (ppm)

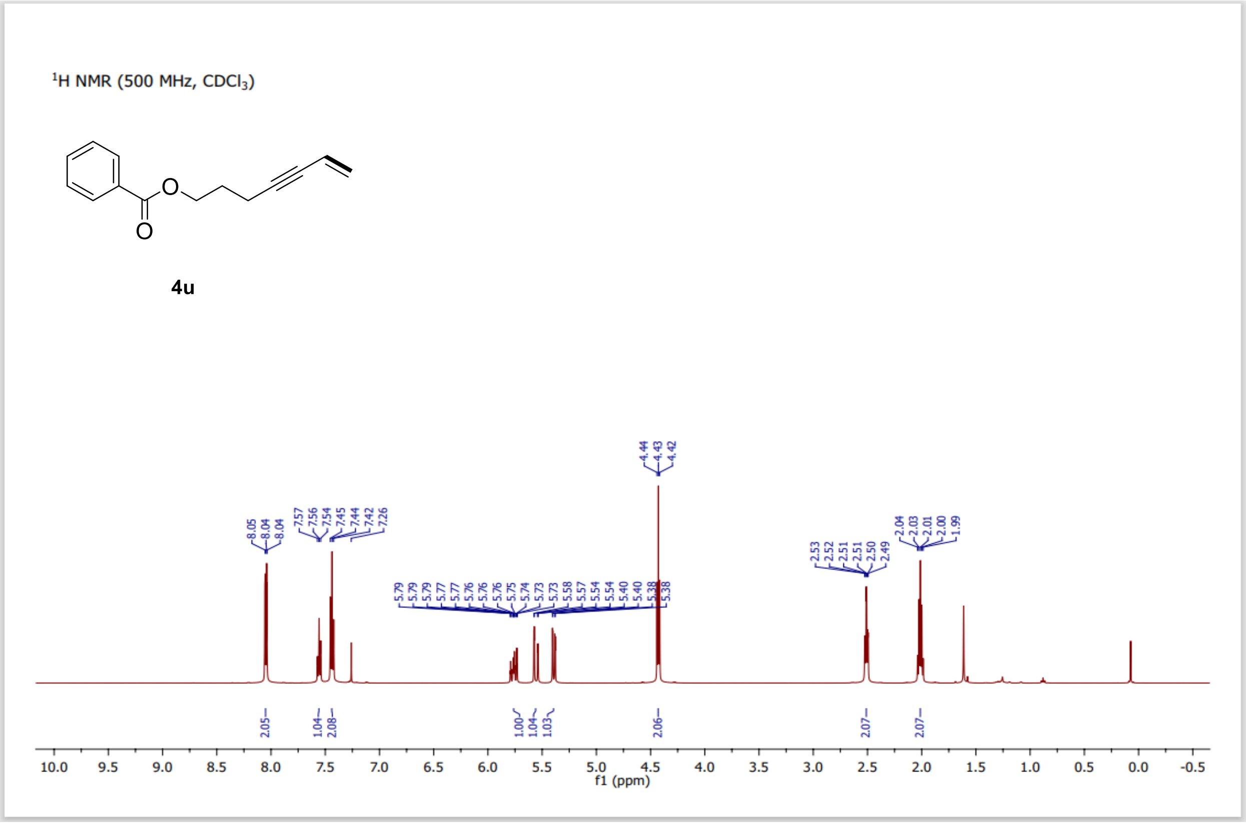

^{1}H NMR (500 MHz, CDCl$_3$)
O
O
4u
8.05 8.04 8.04
7.57 7.56 7.54 7.45 7.44 7.42 7.26
5.79 5.79 5.79 5.77 5.77 5.76 5.76 5.76 5.75 5.74 5.73 5.73 5.58 5.57 5.54 5.54 5.40 5.40 5.38 5.38
4.44 4.43 4.42
2.53 2.52 2.51 2.51 2.50 2.49
2.04 2.03 2.01 2.00 1.99
2.05 1.04 2.08 1.00 1.04 1.03 2.06 2.07 2.07
10.0 9.5 9.0 8.5 8.0 7.5 7.0 6.5 6.0 5.5 5.0 4.5 4.0 3.5 3.0 2.5 2.0 1.5 1.0 0.5 0.0 -0.5
f1 (ppm)

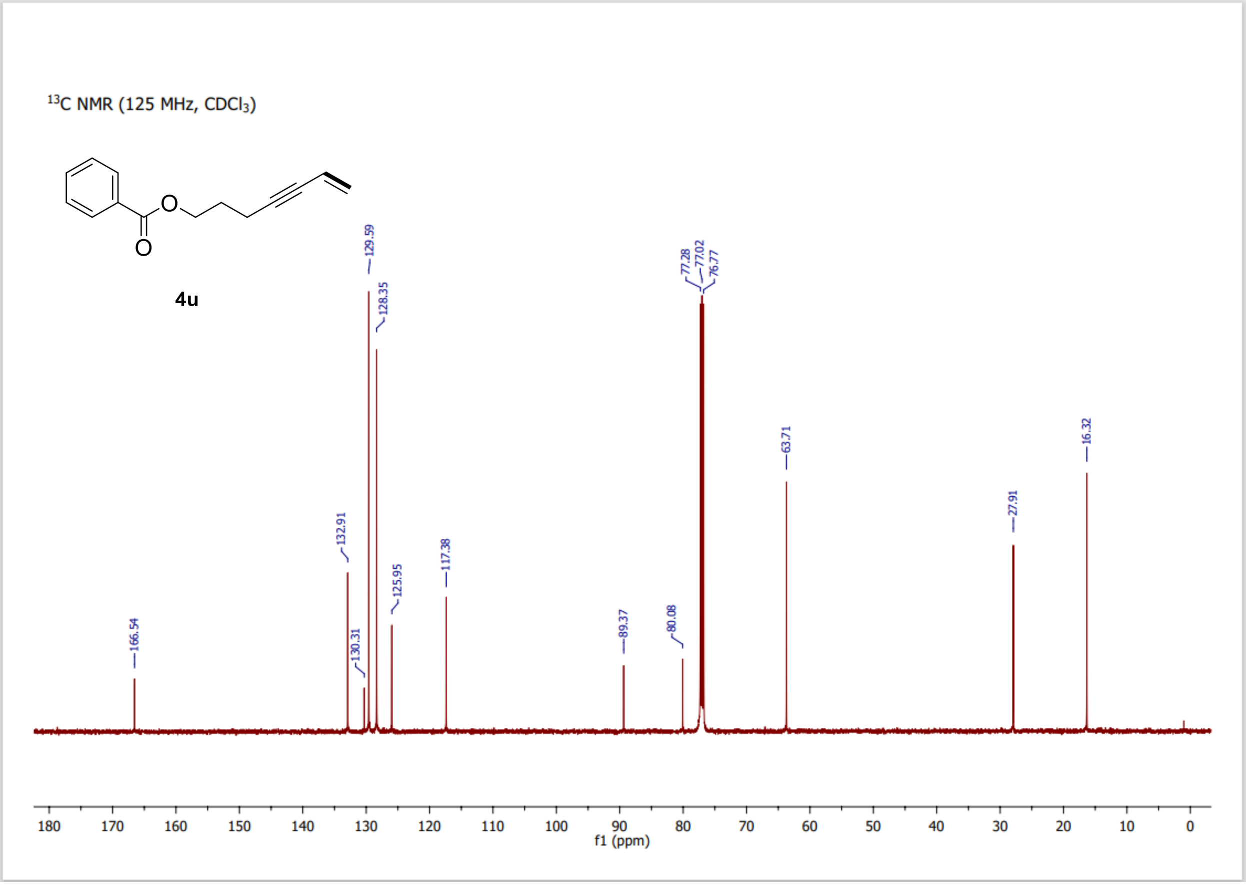

^{13}C NMR (125 MHz, CDCl$_3$)
O
O
4u
166.54
132.91
130.31
129.59
128.35
125.95
117.38
89.37
80.08
77.28 77.02 76.77
63.71
27.91
16.32
180 170 160 150 140 130 120 110 100 90 80 70 60 50 40 30 20 10 0
f1 (ppm)

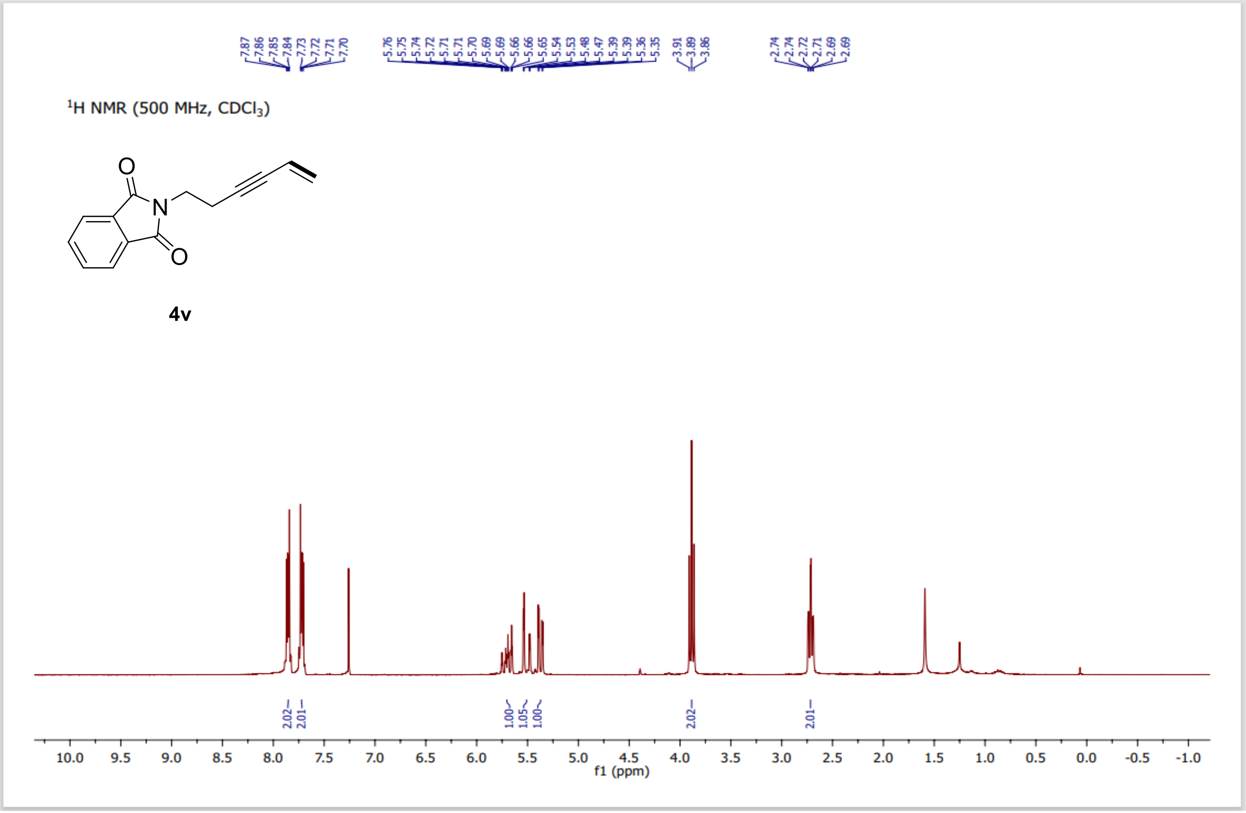

1H NMR (500 MHz, CDCl3)
7.87 7.86 7.85 7.84 7.73 7.72 7.71 7.70
5.76 5.75 5.74 5.72 5.71 5.71 5.70 5.69 5.69 5.66 5.66 5.65 5.54 5.53 5.48 5.47 5.39 5.39 5.36 5.35
3.91 3.89 3.86
2.74 2.74 2.72 2.71 2.69 2.69
O
N
O
4v
2.02 2.01 1.00 1.05 1.00 2.02 2.01
10.0 9.5 9.0 8.5 8.0 7.5 7.0 6.5 6.0 5.5 5.0 4.5 4.0 3.5 3.0 2.5 2.0 1.5 1.0 0.5 0.0 -0.5 -1.0
f1 (ppm)

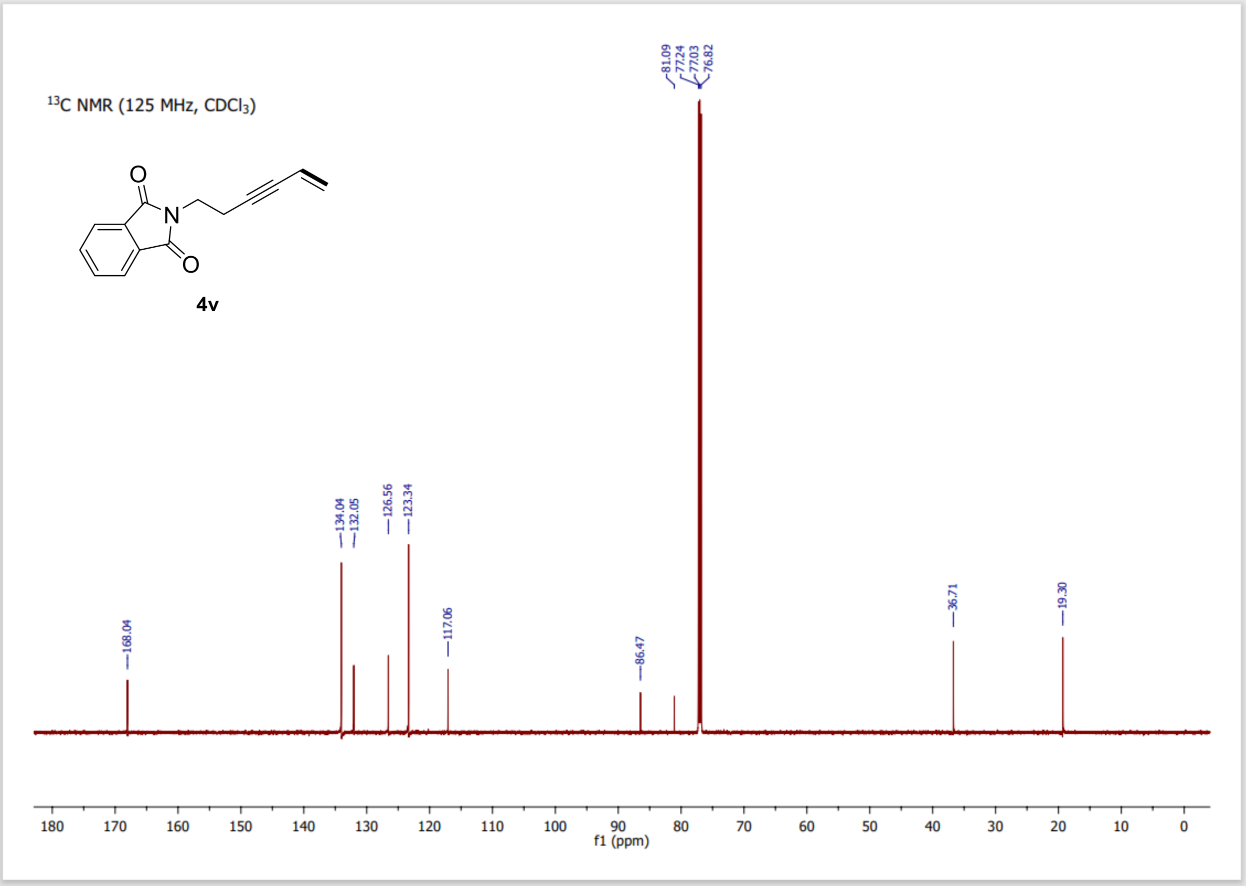

13C NMR (125 MHz, CDCl3)
81.09 77.24 77.03 76.82
O
N
O
4v
134.04 132.05 126.56 123.34
168.04
117.06
86.47
36.71
19.30
180 170 160 150 140 130 120 110 100 90 80 70 60 50 40 30 20 10 0
f1 (ppm)

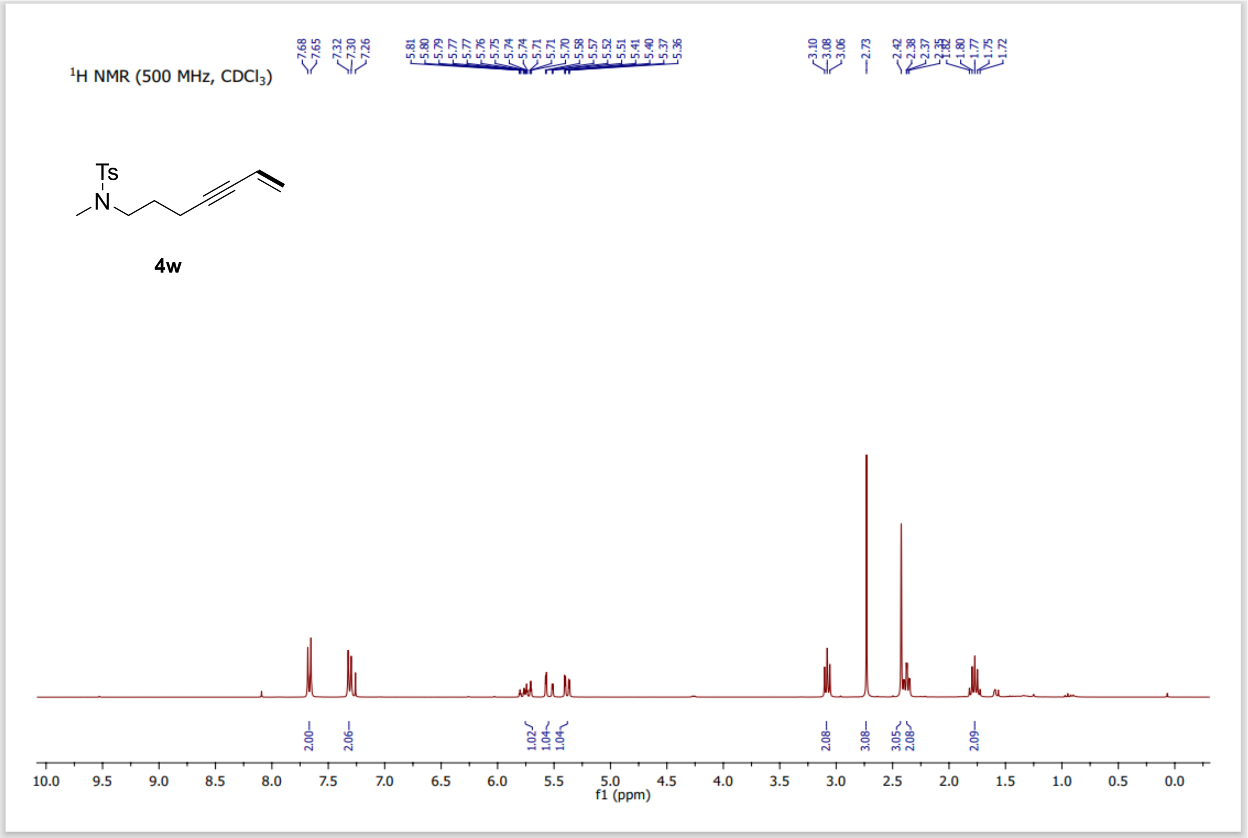

^{1}H NMR (500 MHz, CDCl$_3$)
Ts
N
4w
f1 (ppm)

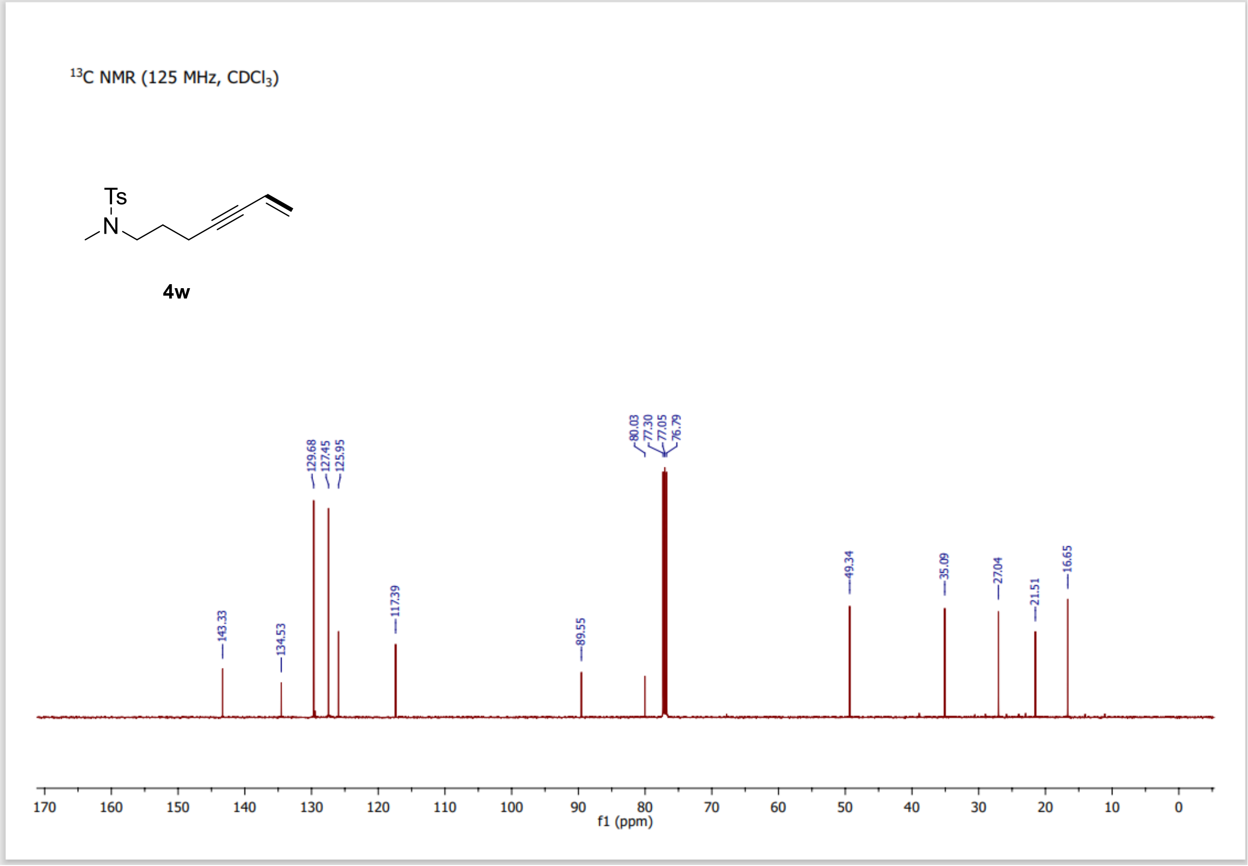

^{13}C NMR (125 MHz, CDCl$_3$)
Ts
N
4w
f1 (ppm)

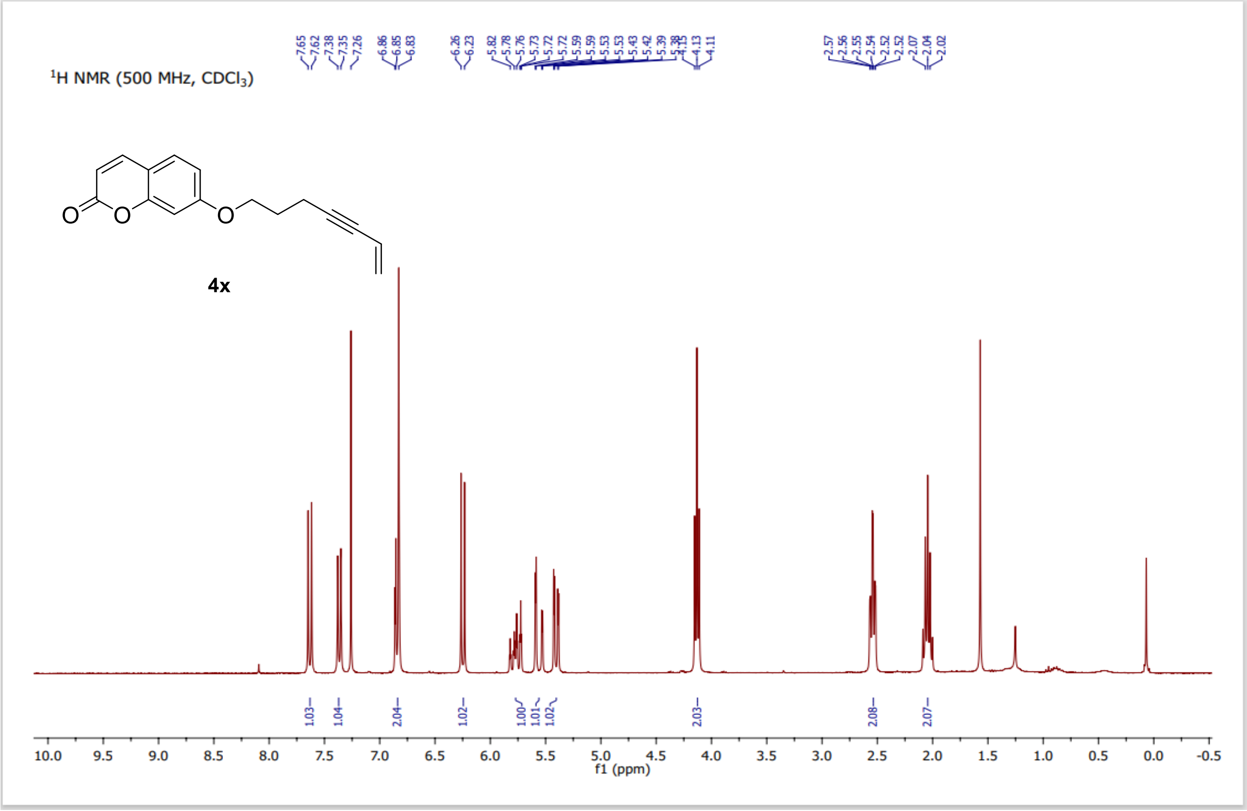

1H NMR (500 MHz, CDCl3)
4x
f1 (ppm)

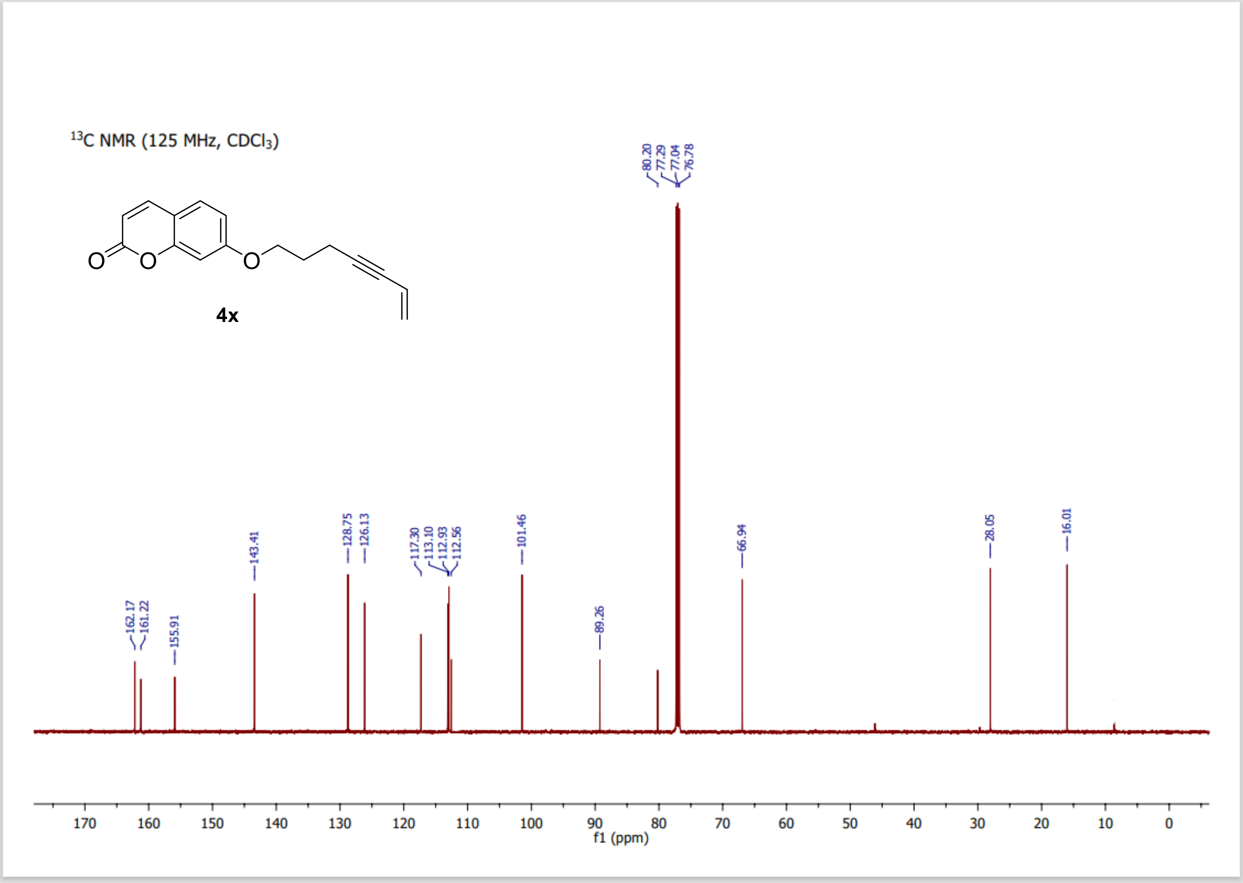

13C NMR (125 MHz, CDCl3)
4x
f1 (ppm)

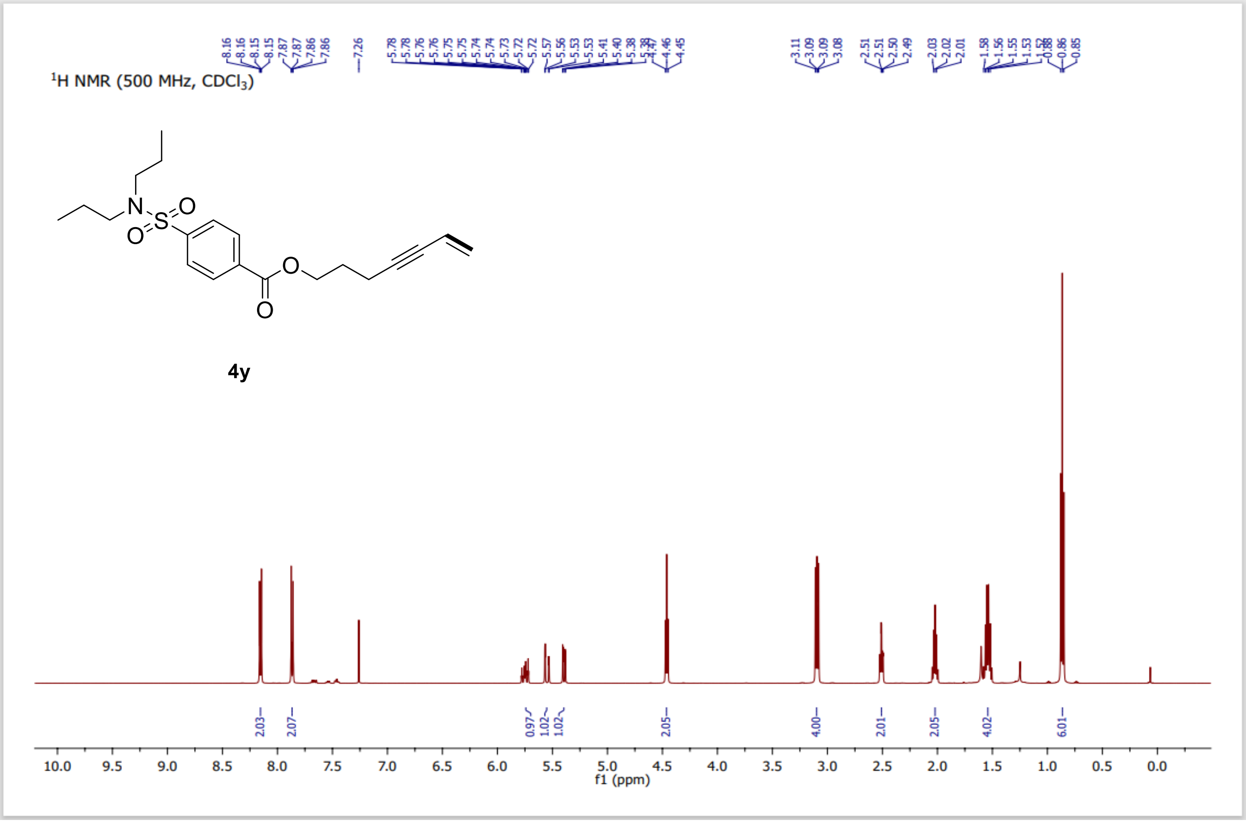

1H NMR (500 MHz, CDCl3)
4y
f1 (ppm)

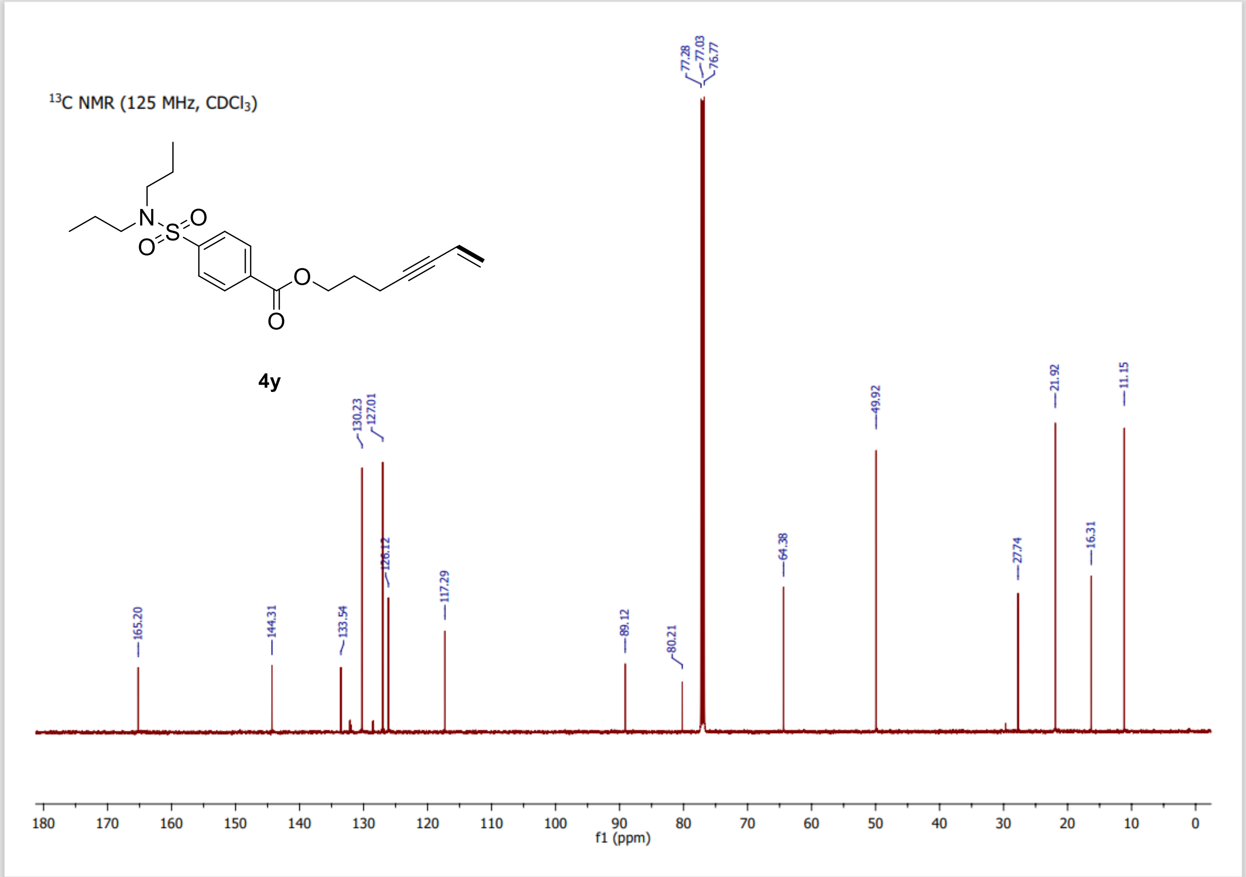

13C NMR (125 MHz, CDCl3)
4y
f1 (ppm)

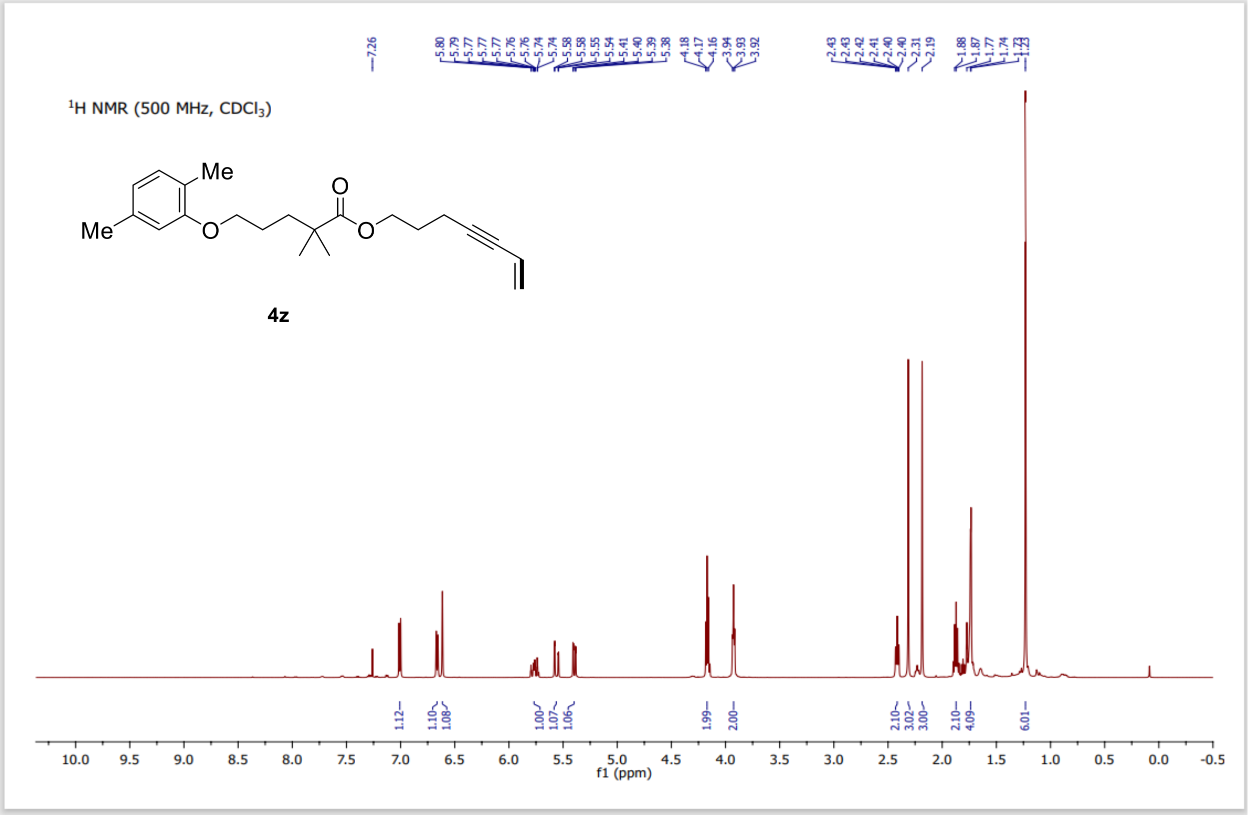
1H NMR (500 MHz, CDCl3)
Me
Me
O
O
O
4z
7.26
5.80 5.79 5.77 5.77 5.77 5.76 5.76 5.74 5.74 5.58 5.58 5.55 5.54 5.41 5.40 5.39 5.38
4.18 4.17 4.16 3.94 3.93 3.92
2.43 2.43 2.42 2.41 2.40 2.40 2.31 2.19
1.88 1.87 1.77 1.74 1.73 1.23
1.12 1.10 1.08 1.00 1.07 1.06 1.99 2.00 2.10 3.02 3.00 2.10 4.09 6.01
10.0 9.5 9.0 8.5 8.0 7.5 7.0 6.5 6.0 5.5 5.0 4.5 4.0 3.5 3.0 2.5 2.0 1.5 1.0 0.5 0.0 -0.5
f1 (ppm)

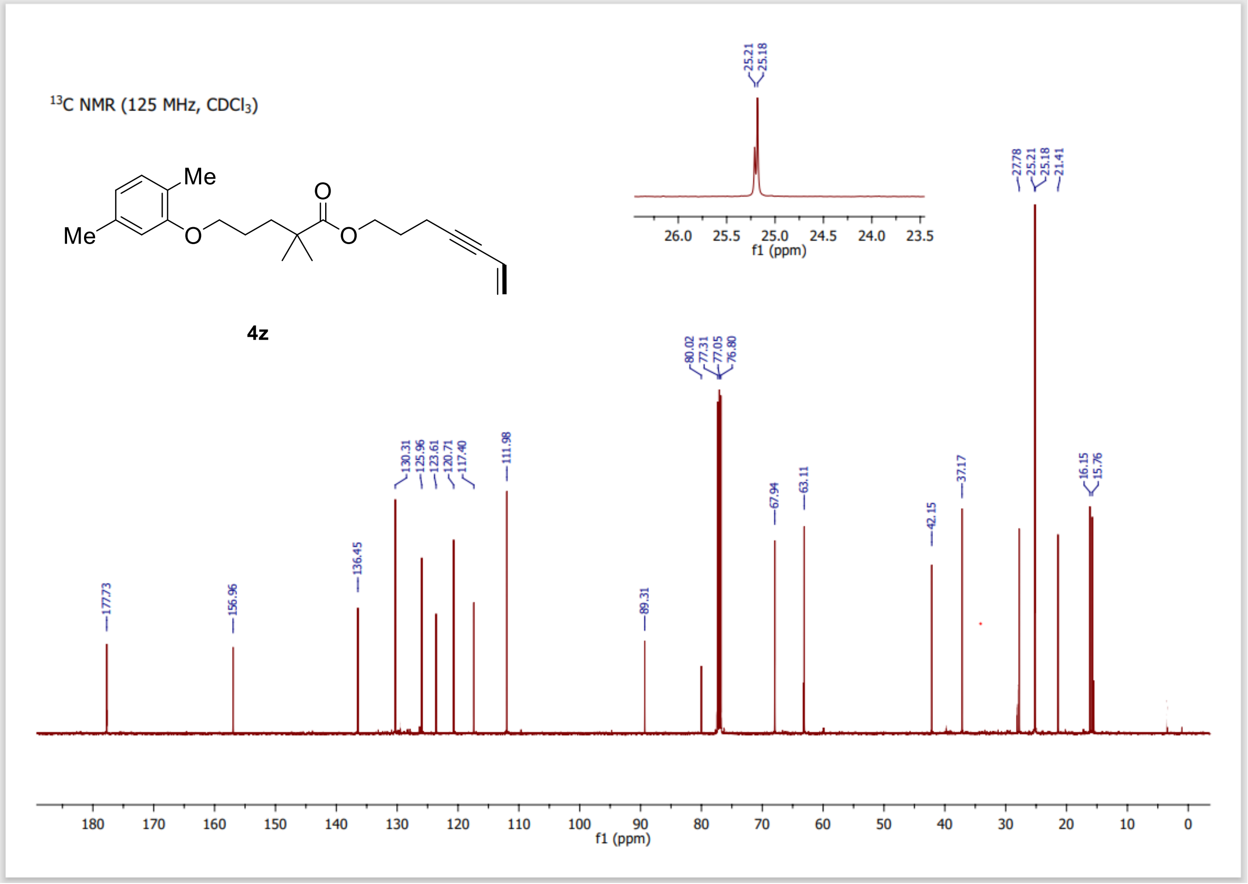
13C NMR (125 MHz, CDCl3)
Me
Me
O
O
O
4z
25.21 25.18
26.0 25.5 25.0 24.5 24.0 23.5
f1 (ppm)
177.73
156.96
136.45
130.31 125.96 123.61 120.71 117.40
111.98
89.31
80.02 77.31 77.05 76.80
67.94
63.11
42.15
37.17
27.78 25.21 25.18 21.41
16.15 15.76
180 170 160 150 140 130 120 110 100 90 80 70 60 50 40 30 20 10 0
f1 (ppm)

^{1}H NMR (500 MHz, $CDCl_3$)

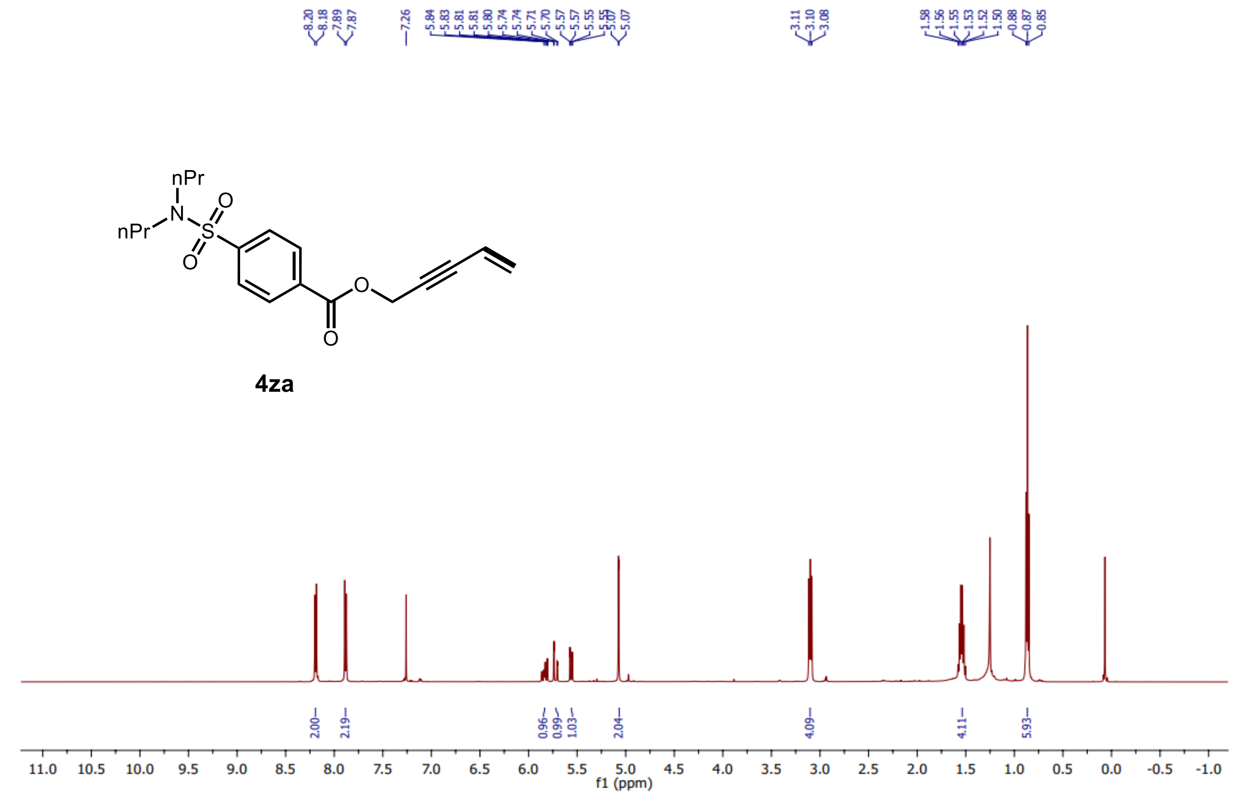

4za

^{13}C NMR (125 MHz, $CDCl_3$)

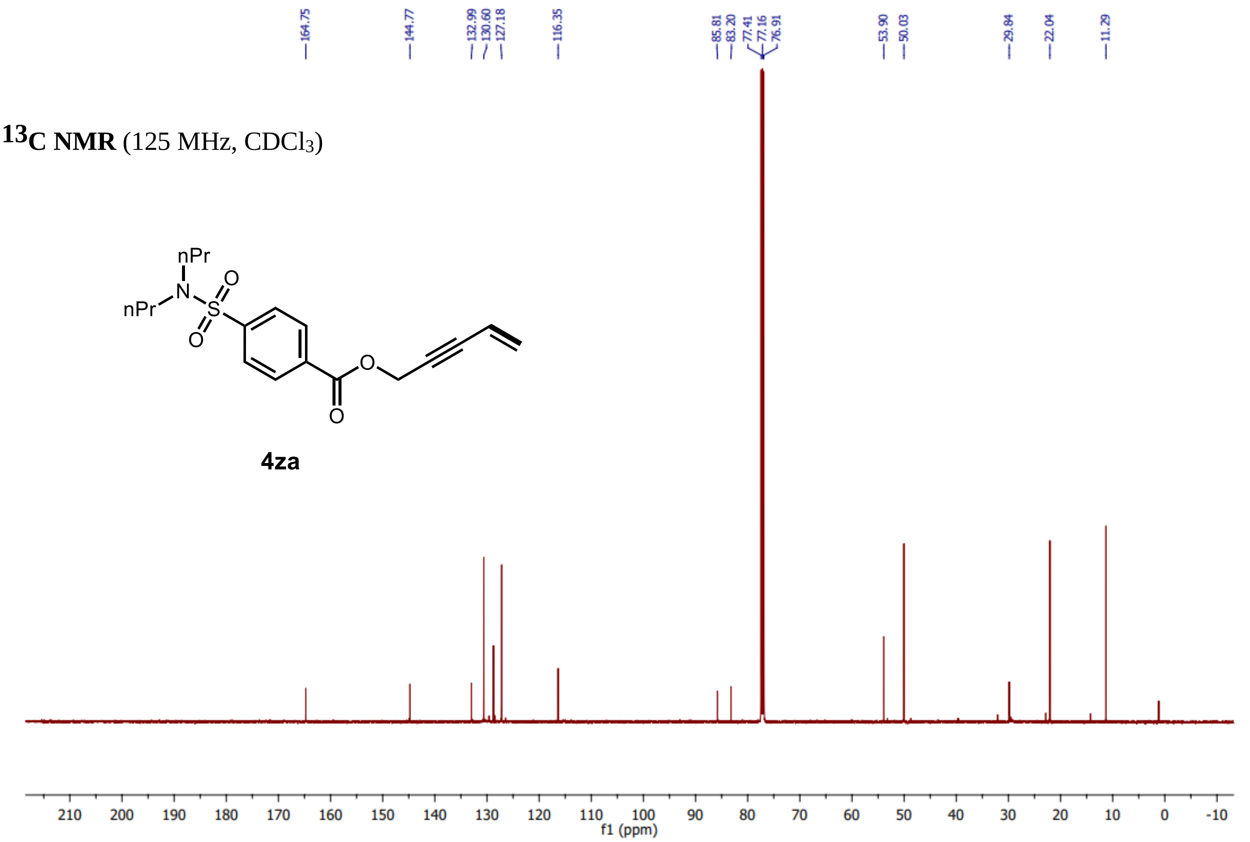

4za

^{1}H NMR (500 MHz, $CDCl_3$)

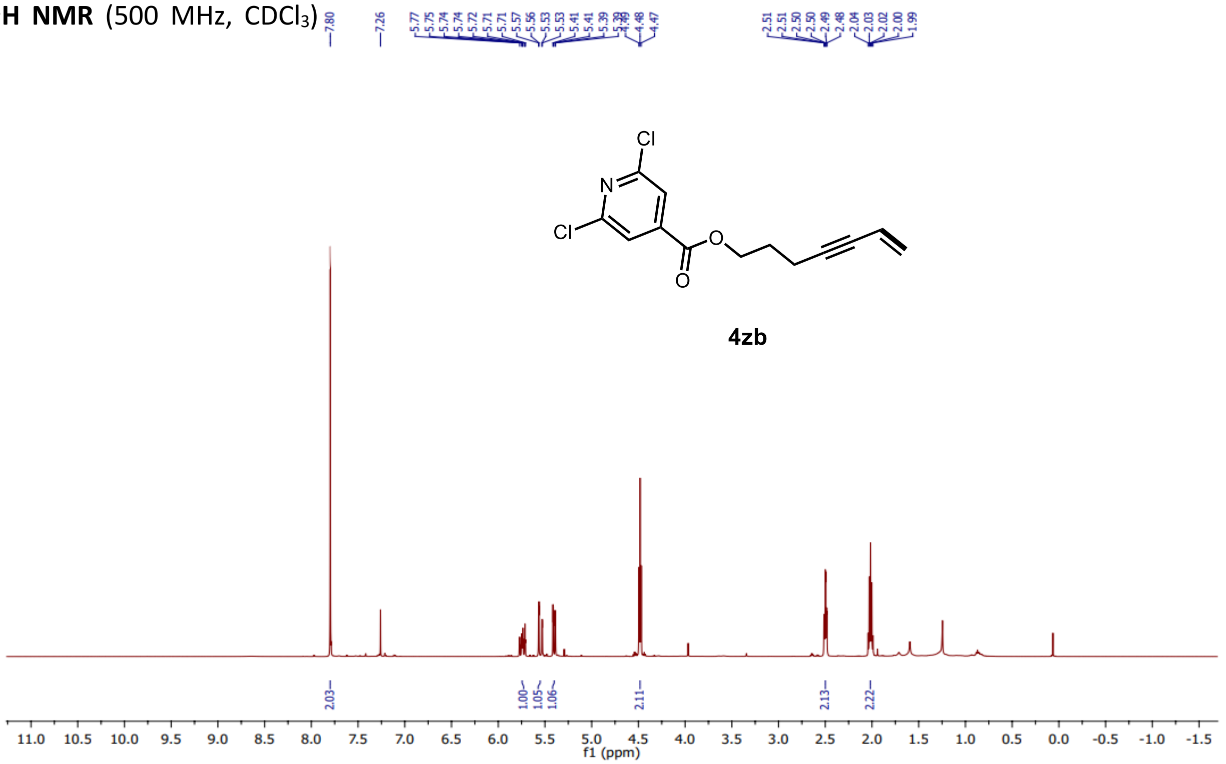

^{13}C NMR (125 MHz, $CDCl_3$)

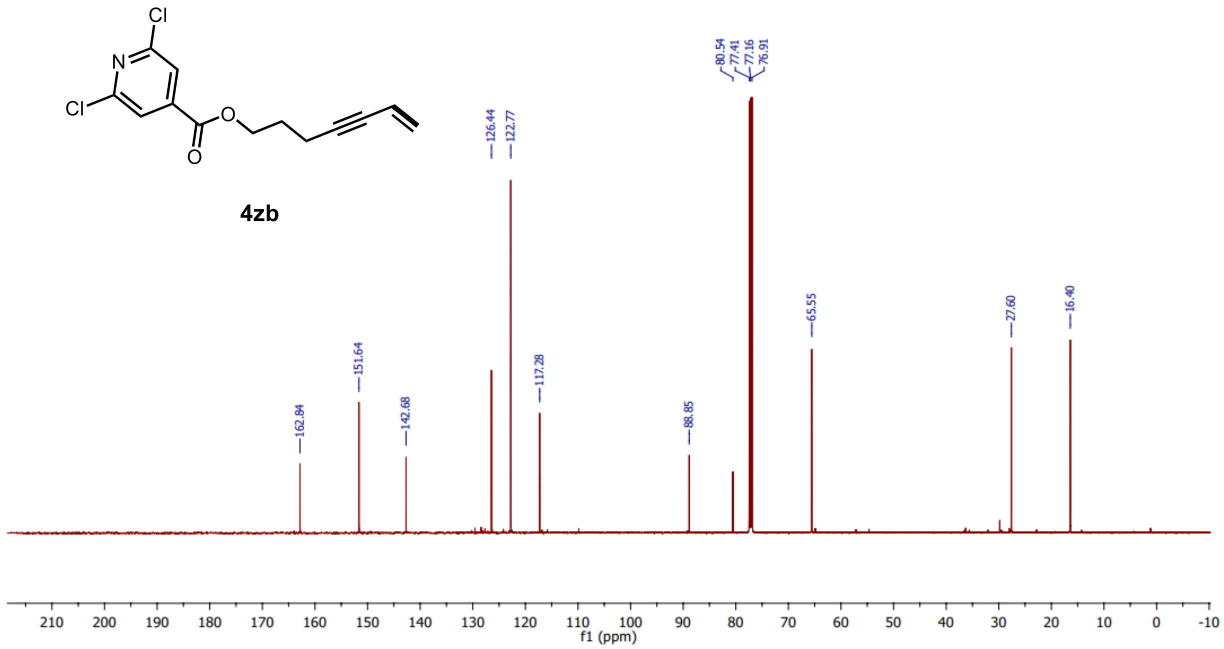

^{1}H NMR (500 MHz, $CDCl_3$)

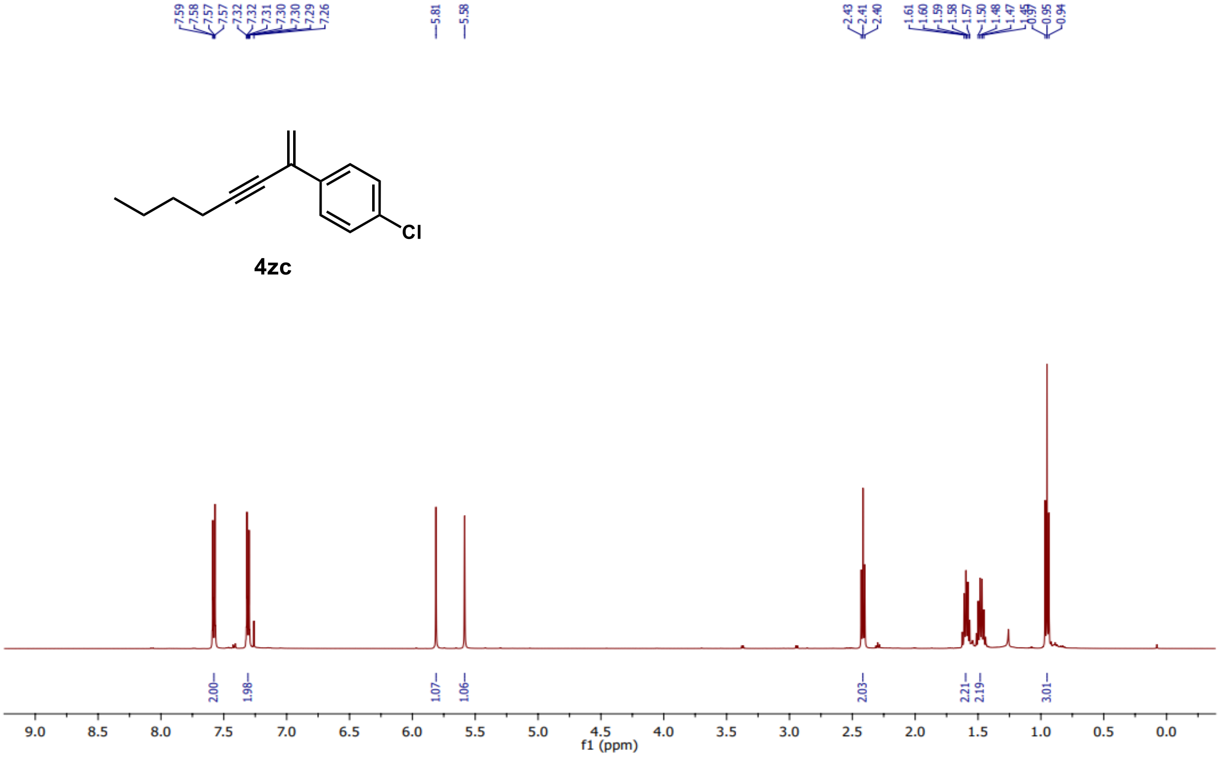

^{13}C NMR (125 MHz, $CDCl_3$)

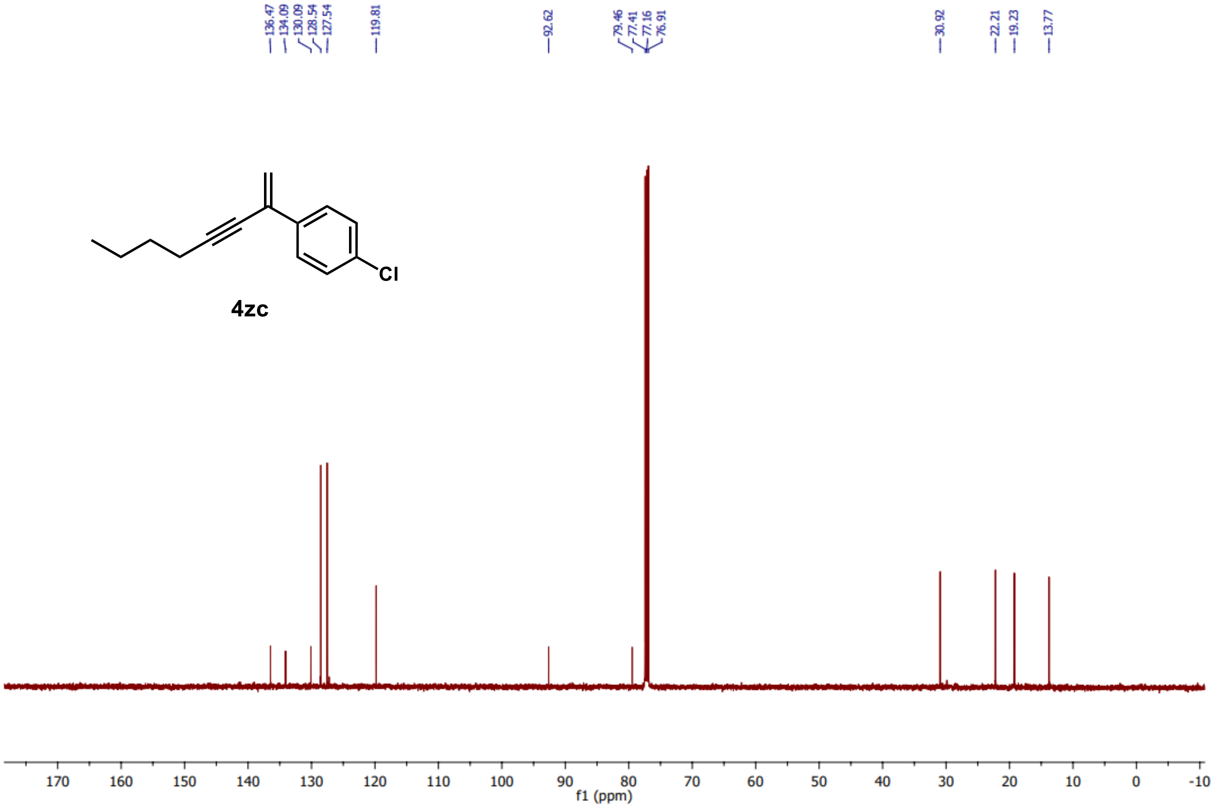

^{1}H NMR (500 MHz, $CDCl_3$)

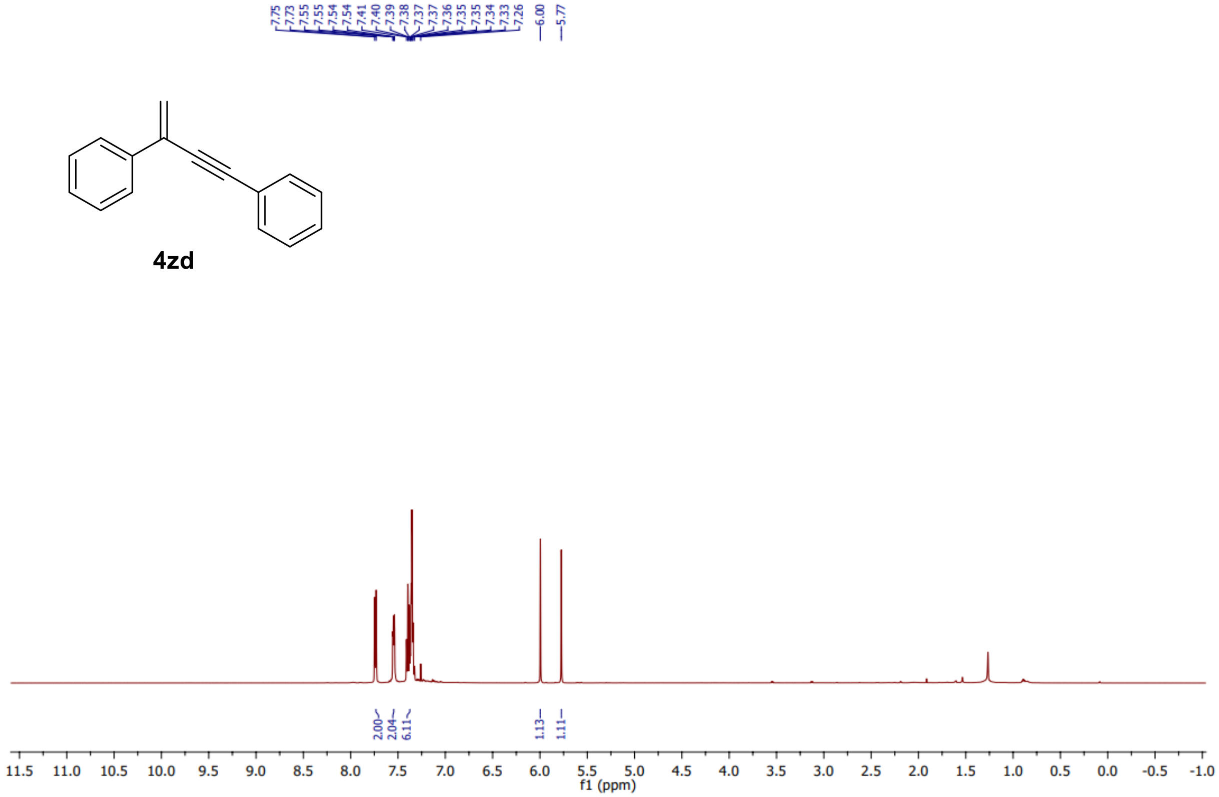

^{13}C NMR (125 MHz, $CDCl_3$)

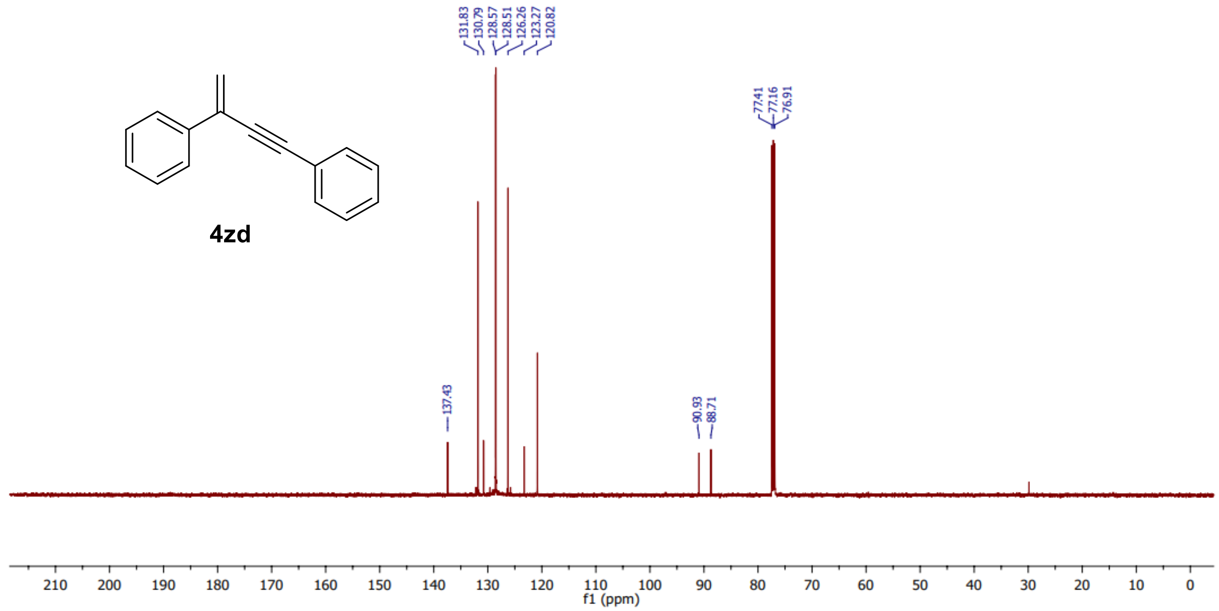

^{1}H NMR (500 MHz, $CDCl_3$)

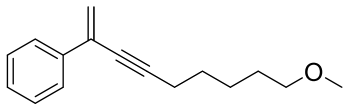

4ze

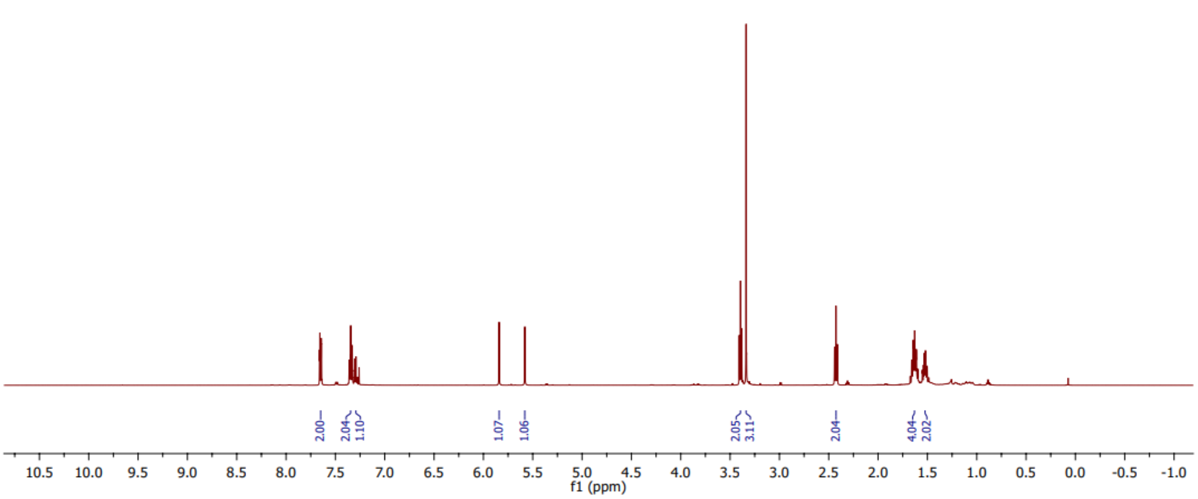

^{13}C NMR (125 MHz, $CDCl_3$)

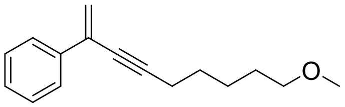

4ze

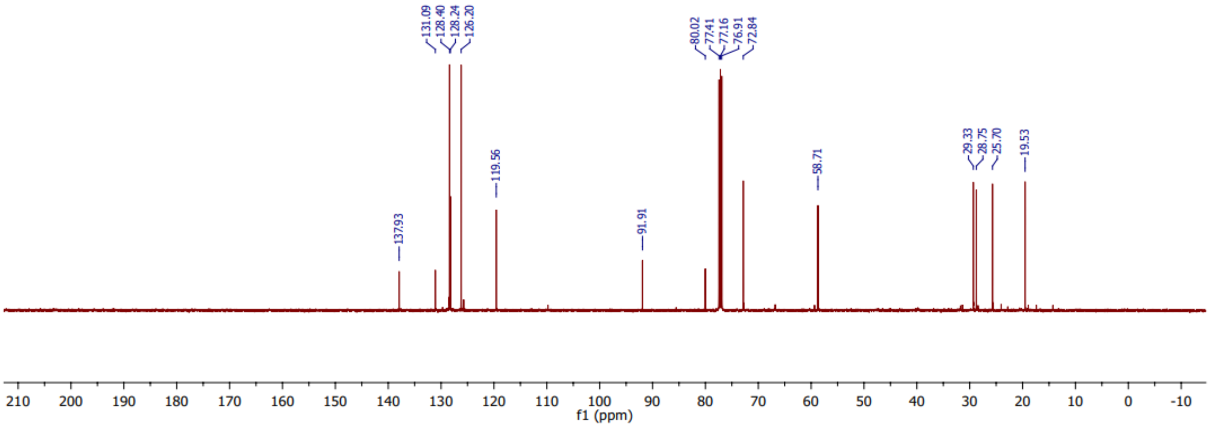

^{1}H NMR (500 MHz, $CDCl_3$)

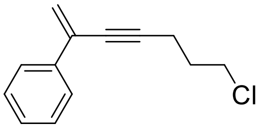

4zf

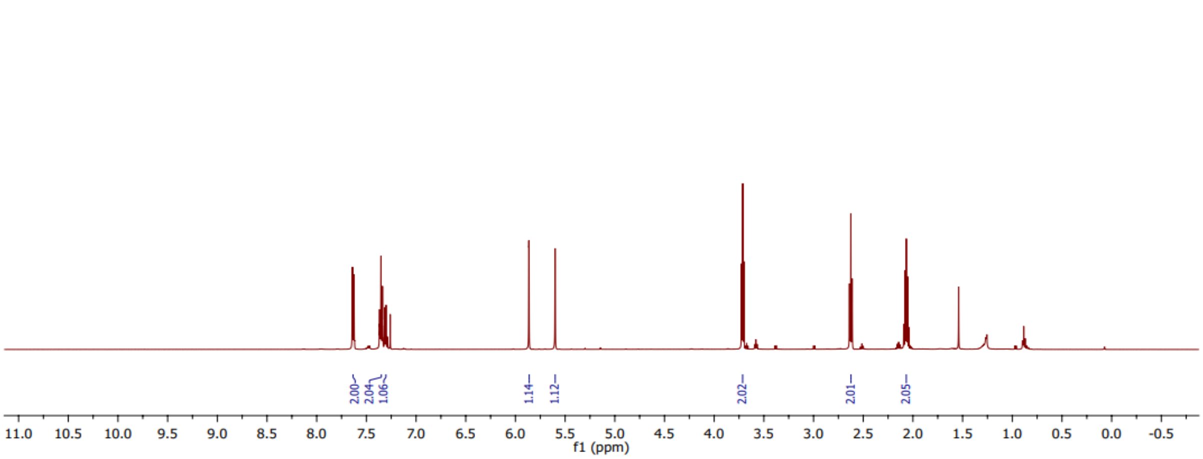

^{13}C NMR (125 MHz, $CDCl_3$)

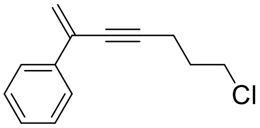

4zf

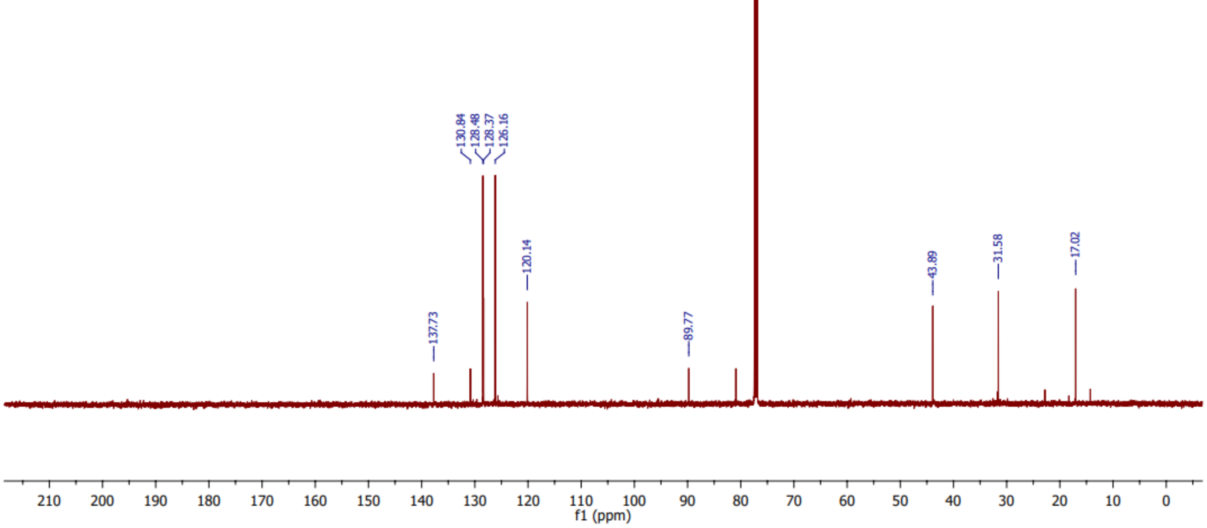

^{1}H NMR (500 MHz, $CDCl_3$)

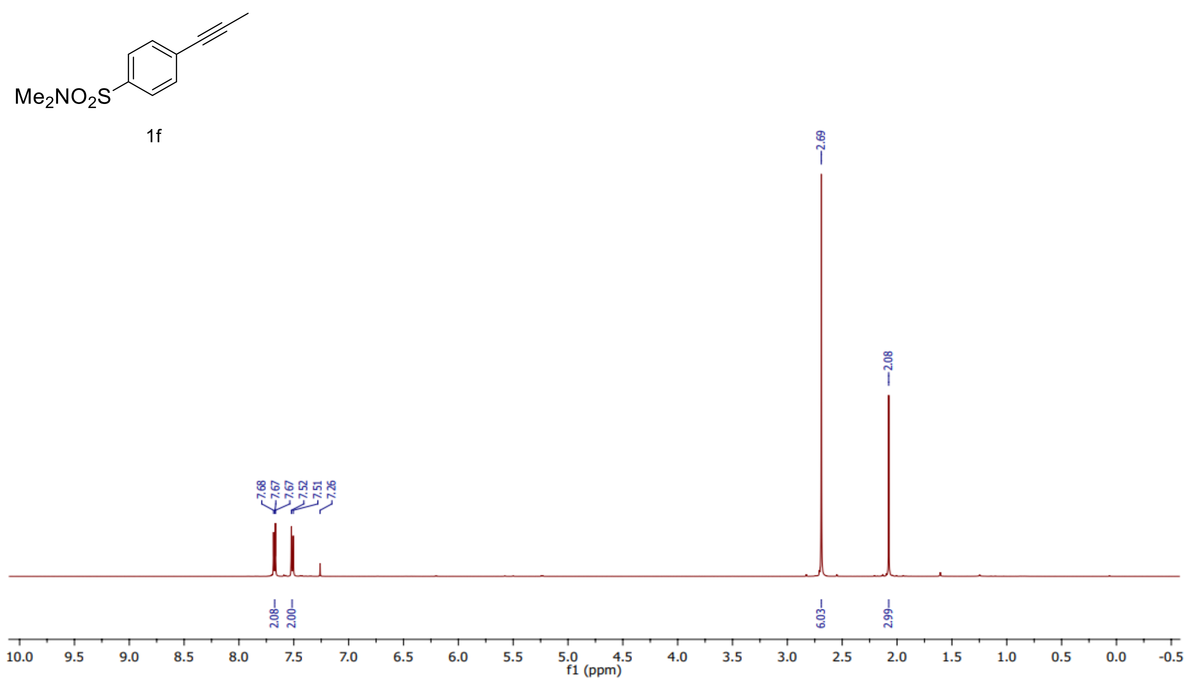

^{13}C NMR (125 MHz, $CDCl_3$)

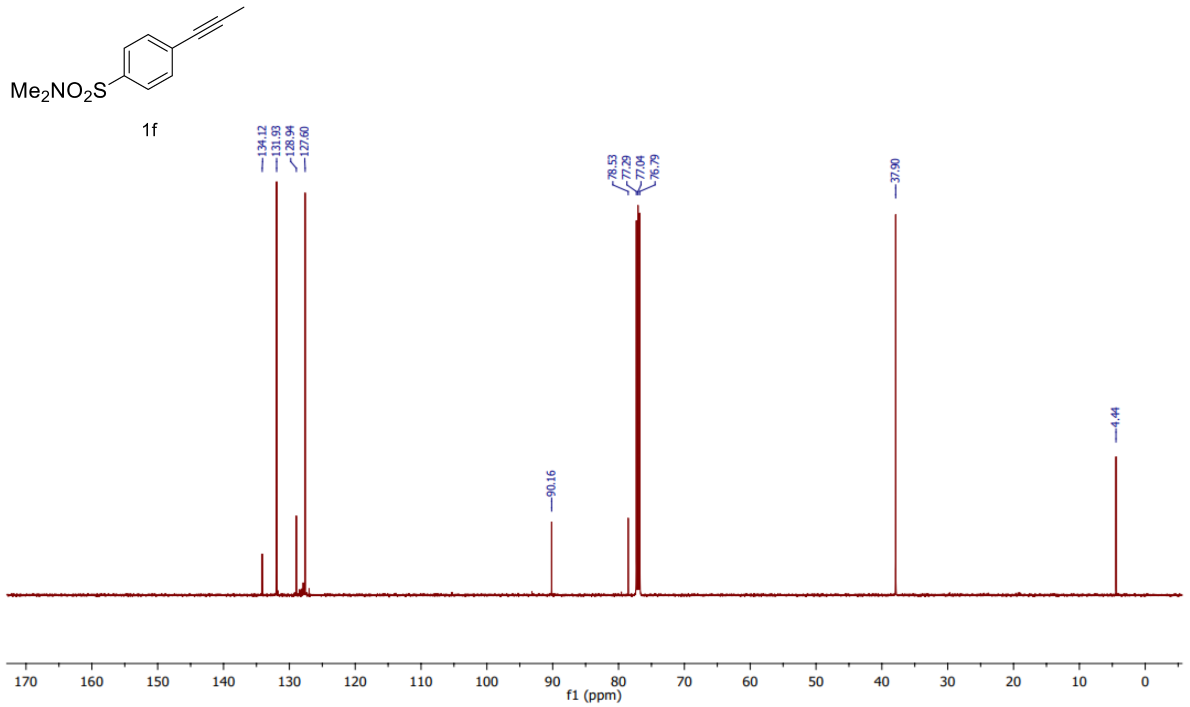

^{1}H NMR (500 MHz, $CDCl_3$)

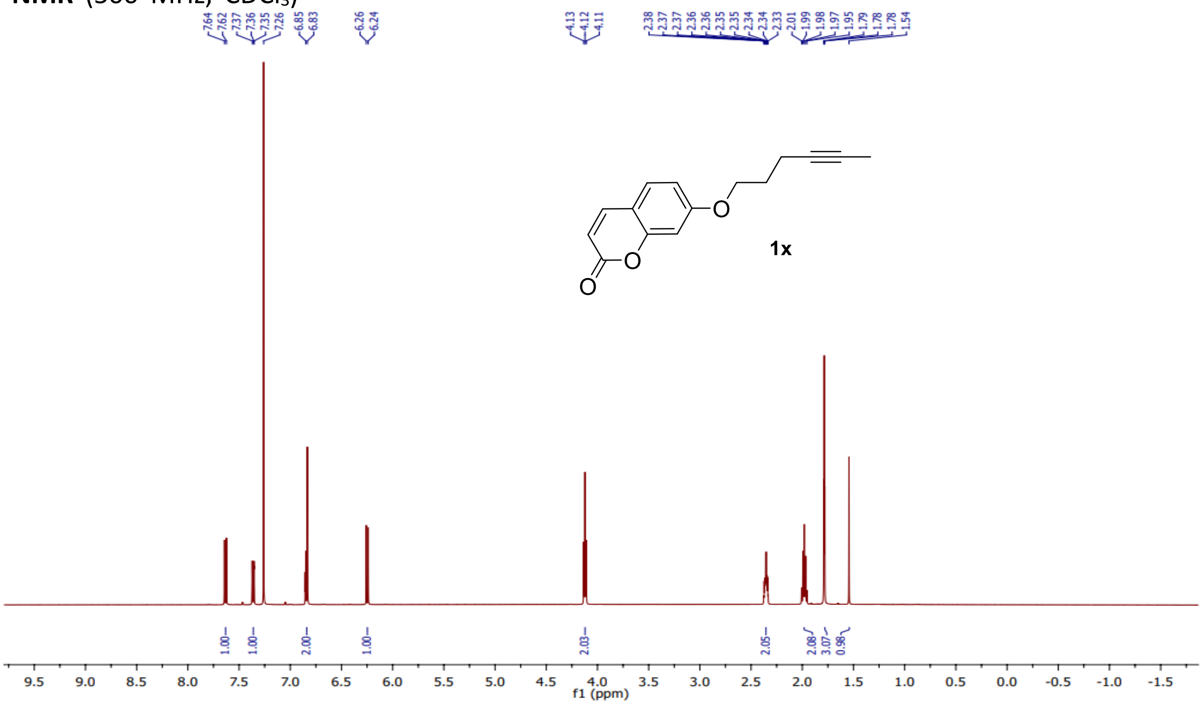

^{13}C NMR (125 MHz, $CDCl_3$)

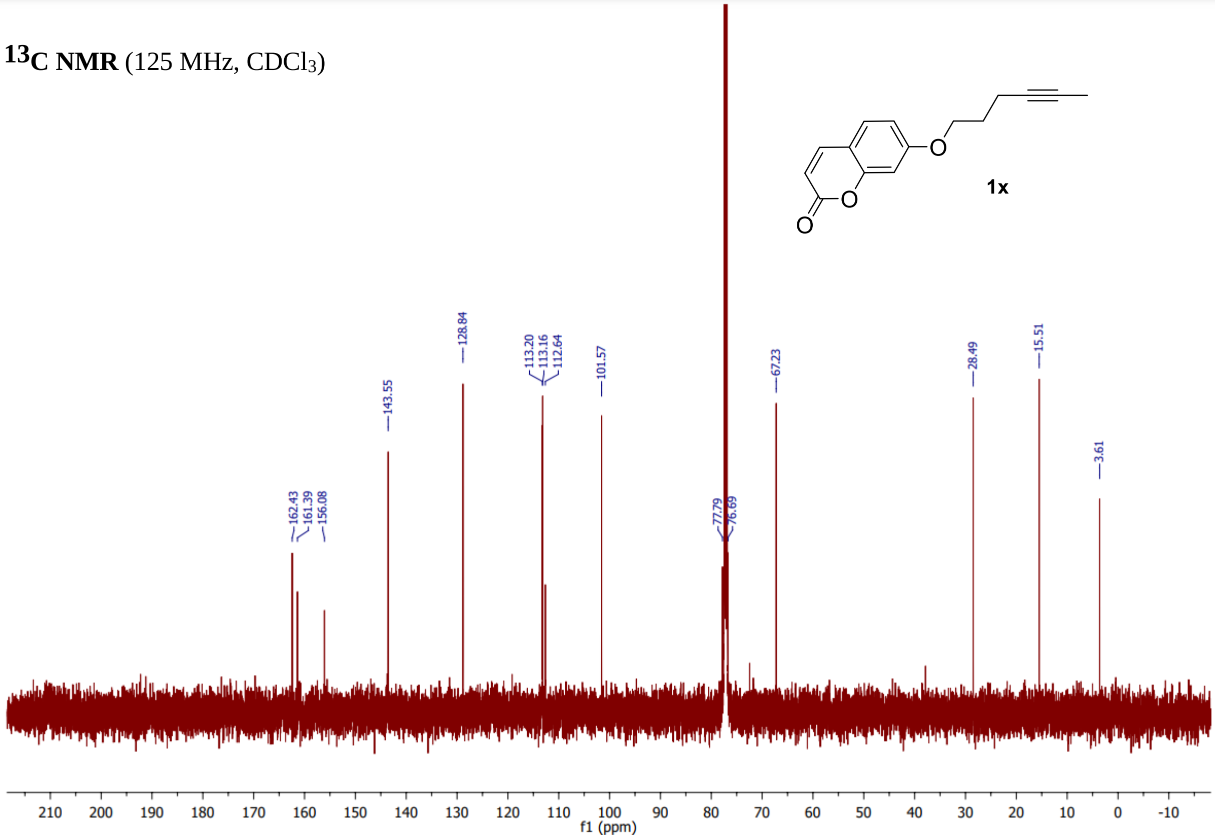

^{1}H NMR (500 MHz, $CDCl_3$)

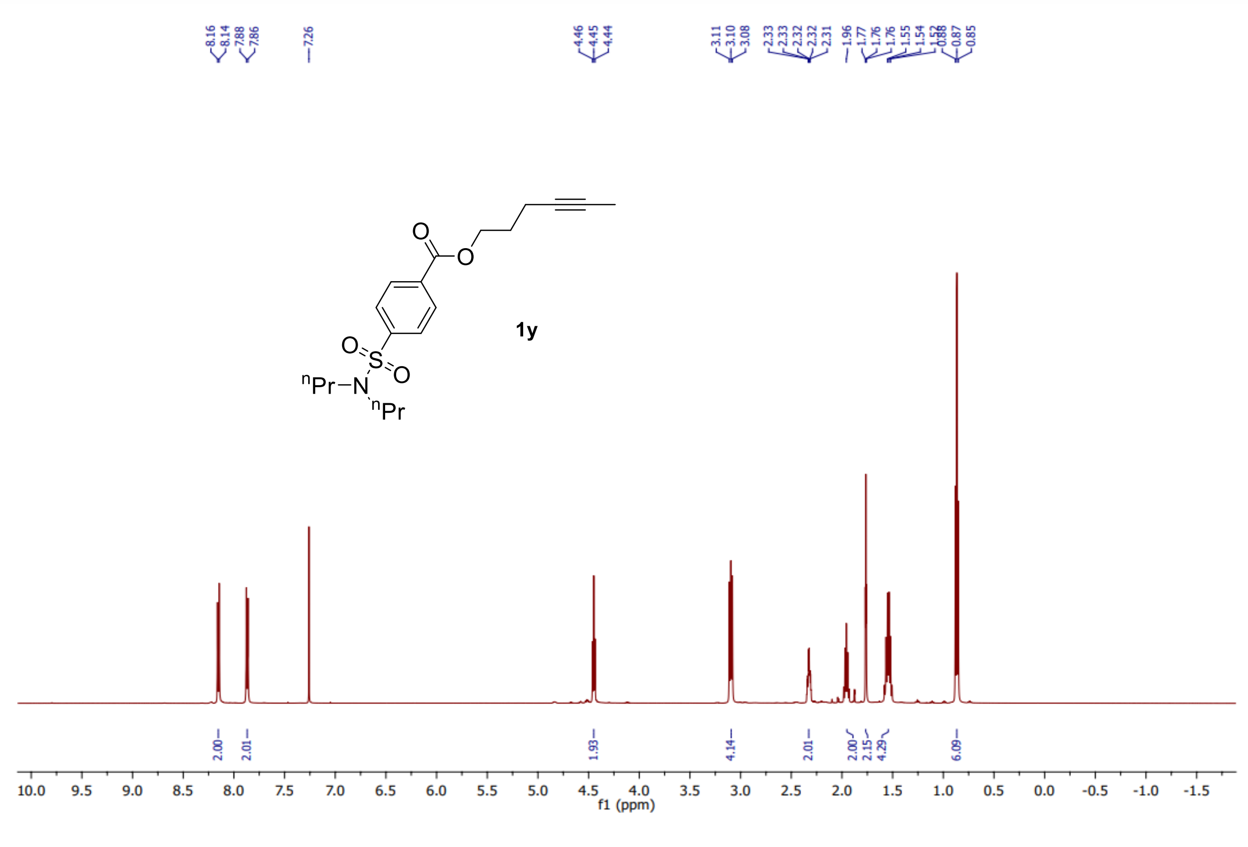

^{13}C NMR (125 MHz, $CDCl_3$)

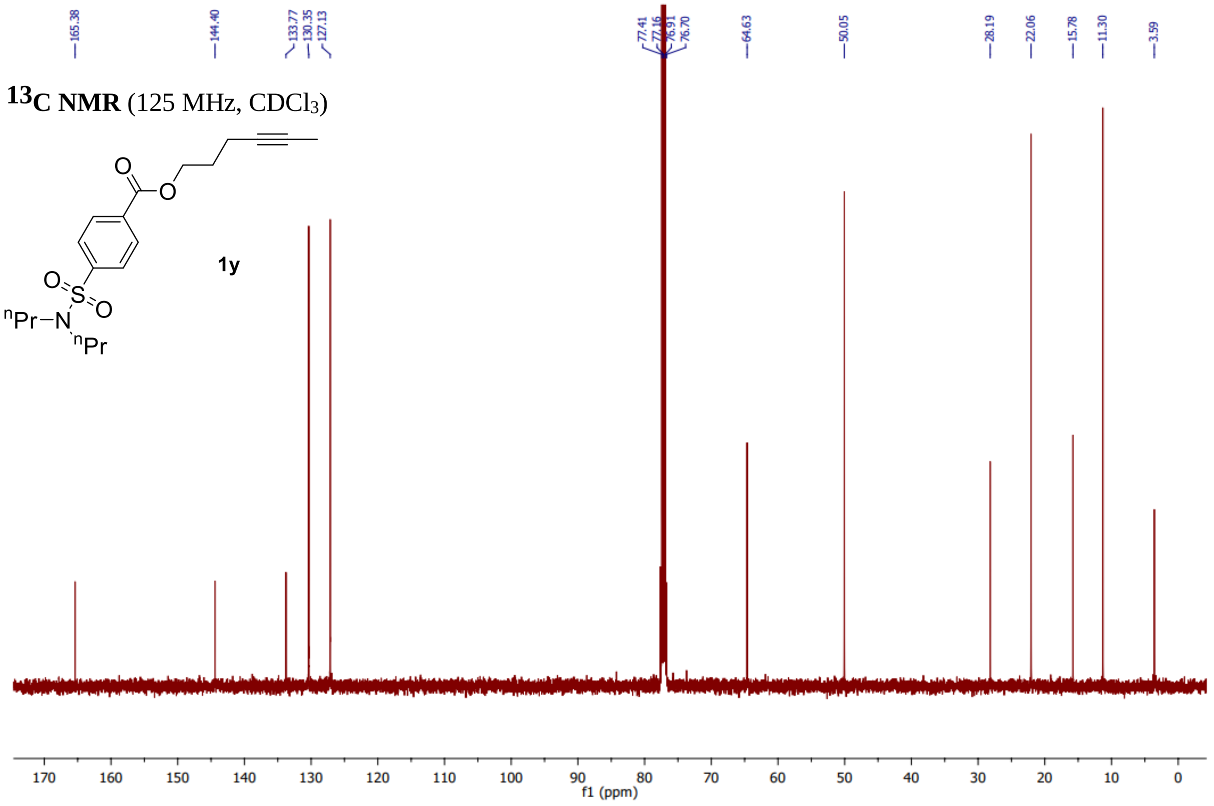

^{1}H NMR (500 MHz, $CDCl_3$)

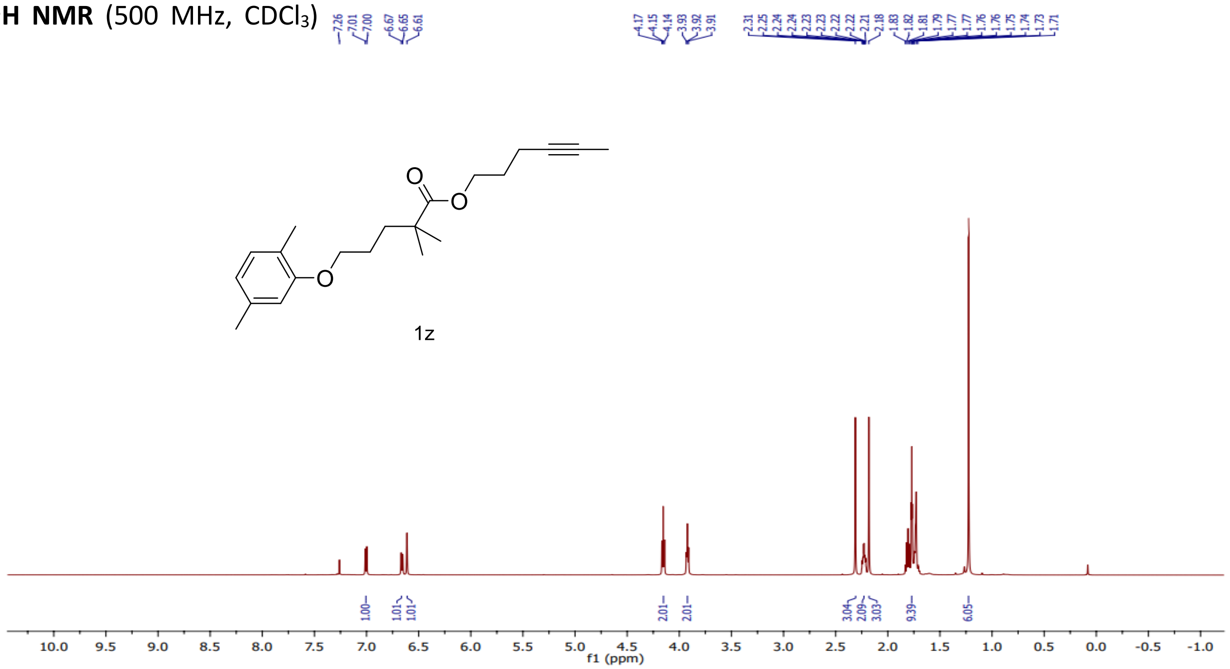

^{13}C NMR (125 MHz, $CDCl_3$)

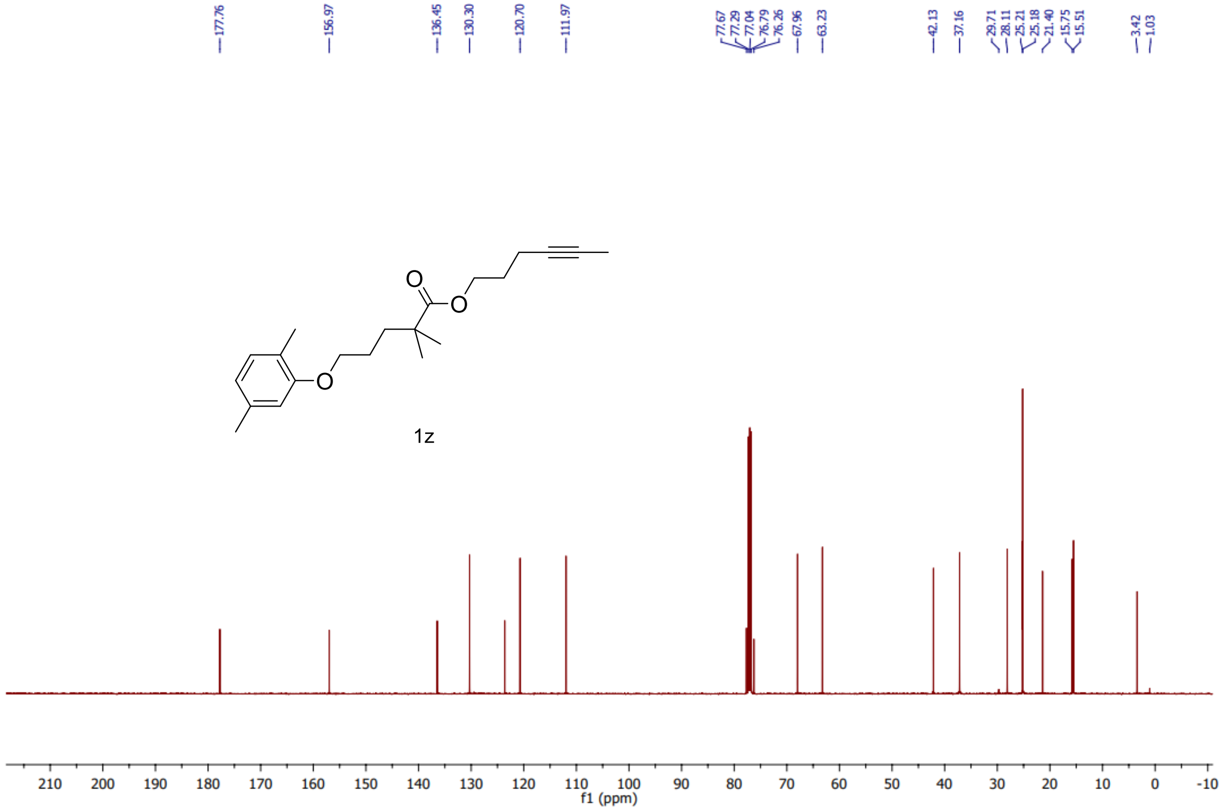

^{1}H NMR (500 MHz, $CDCl_3$)

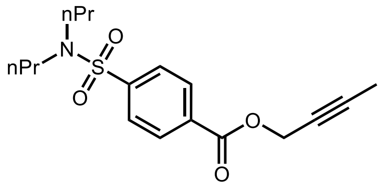

1za

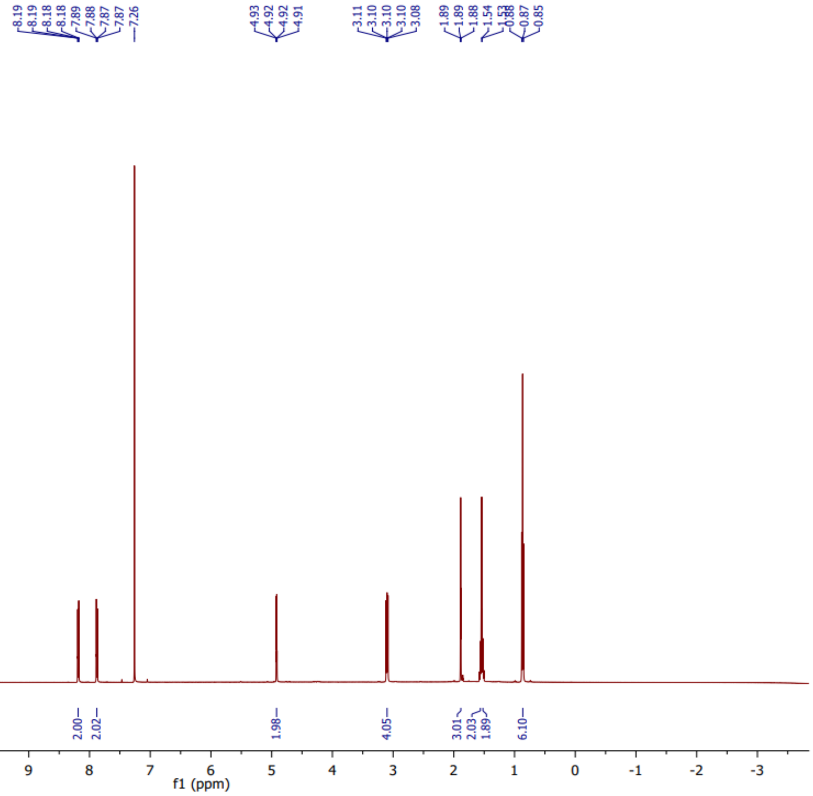

^{13}C NMR (125 MHz, $CDCl_3$)

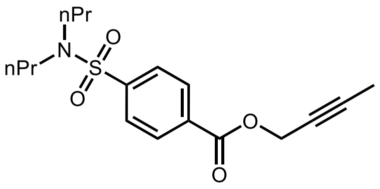

1za

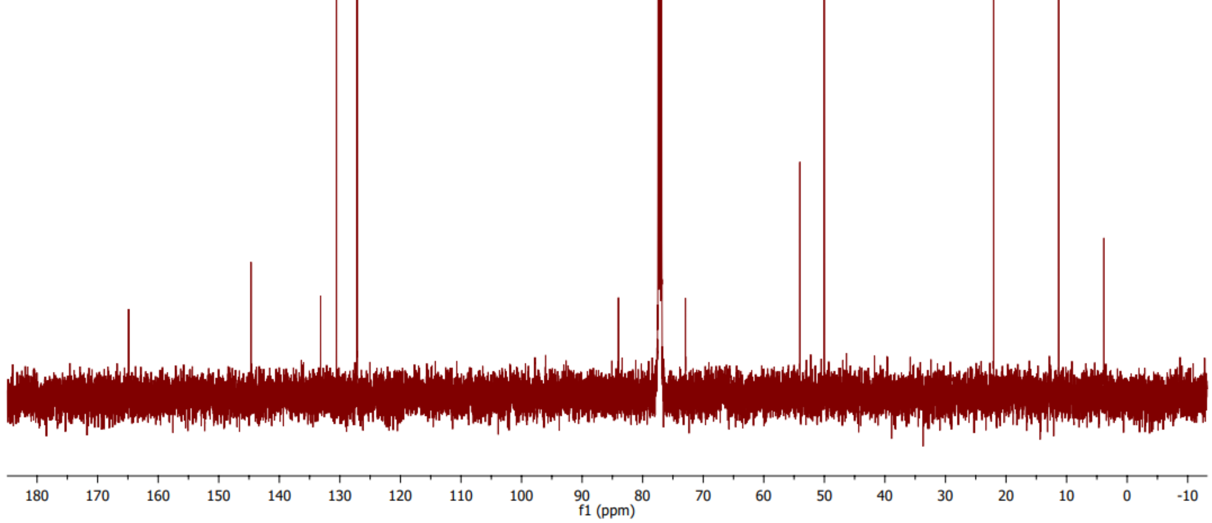

^{1}H NMR (500 MHz, $CDCl_3$)

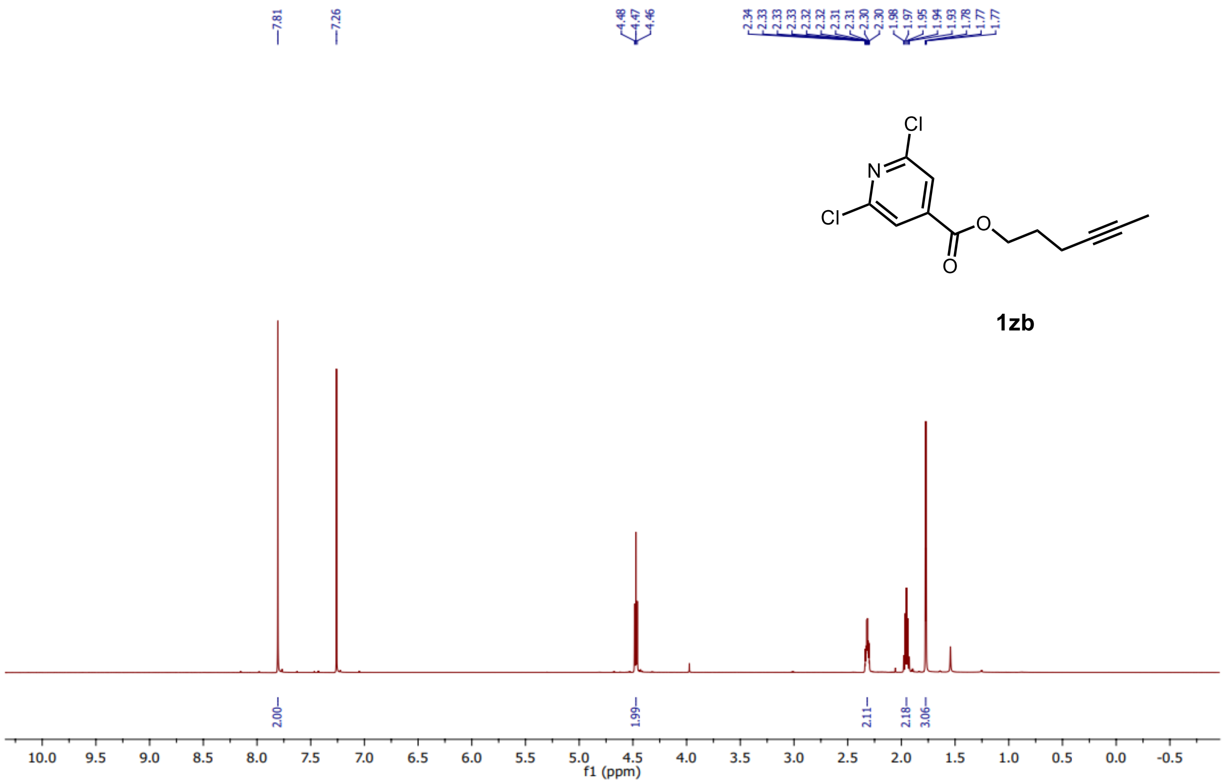

^{13}C NMR (125 MHz, $CDCl_3$)

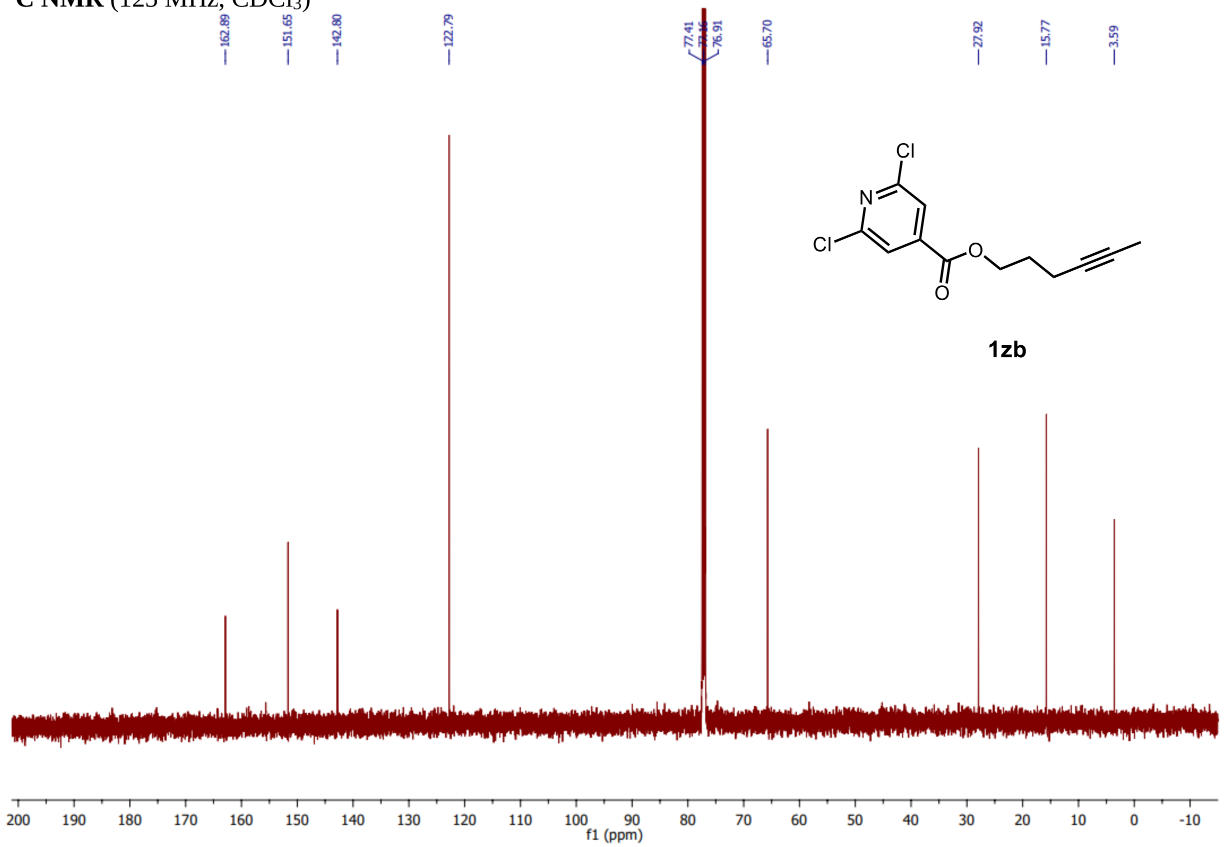

^{1}H NMR (500 MHz, CDCl$_3$)

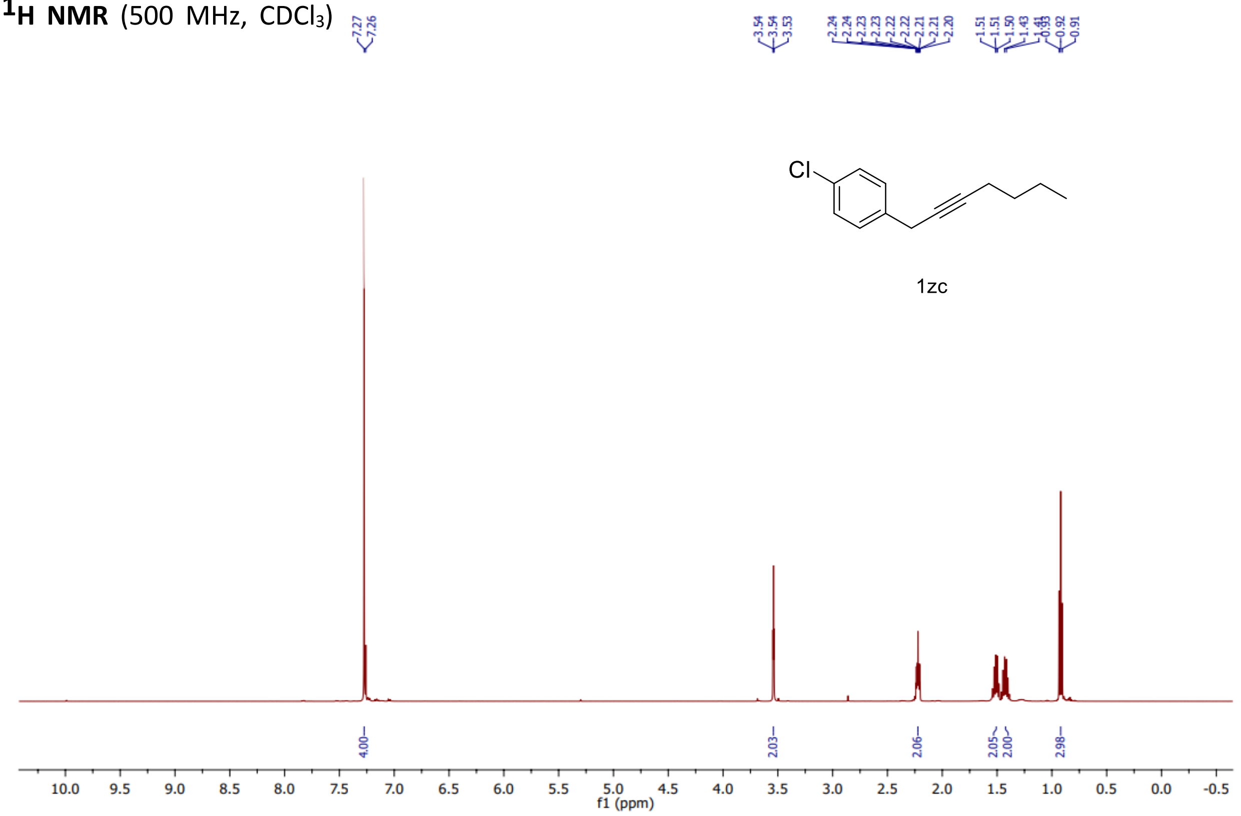

^{13}C NMR (125 MHz, CDCl$_3$)

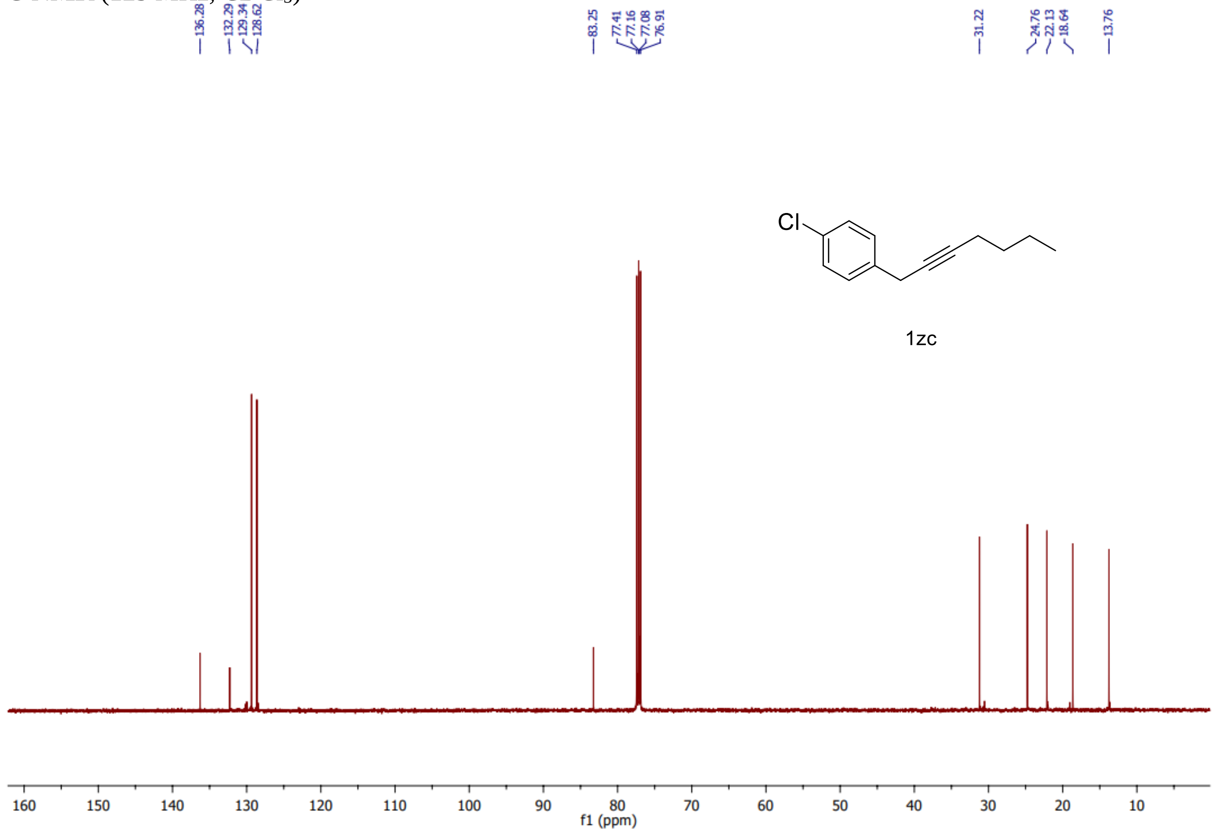

^{1}H NMR (500 MHz, $CDCl_3$)

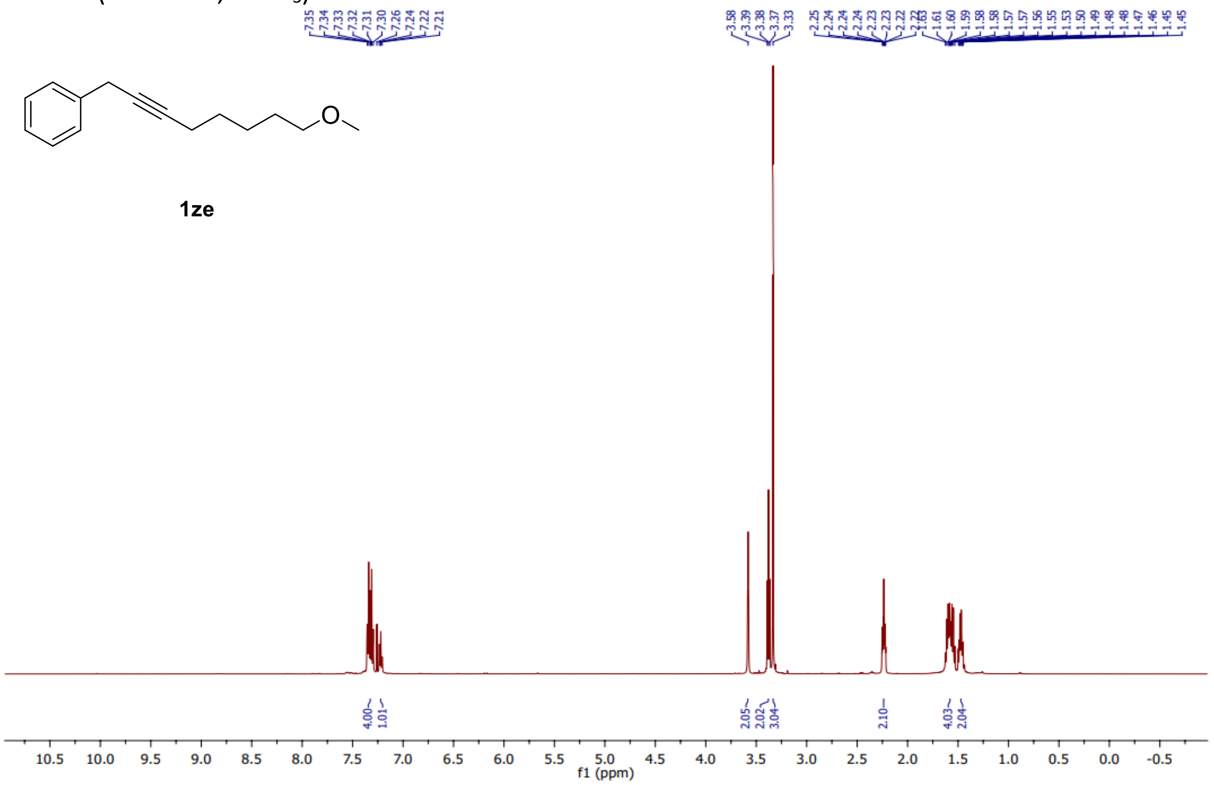

^{13}C NMR (125 MHz, $CDCl_3$)

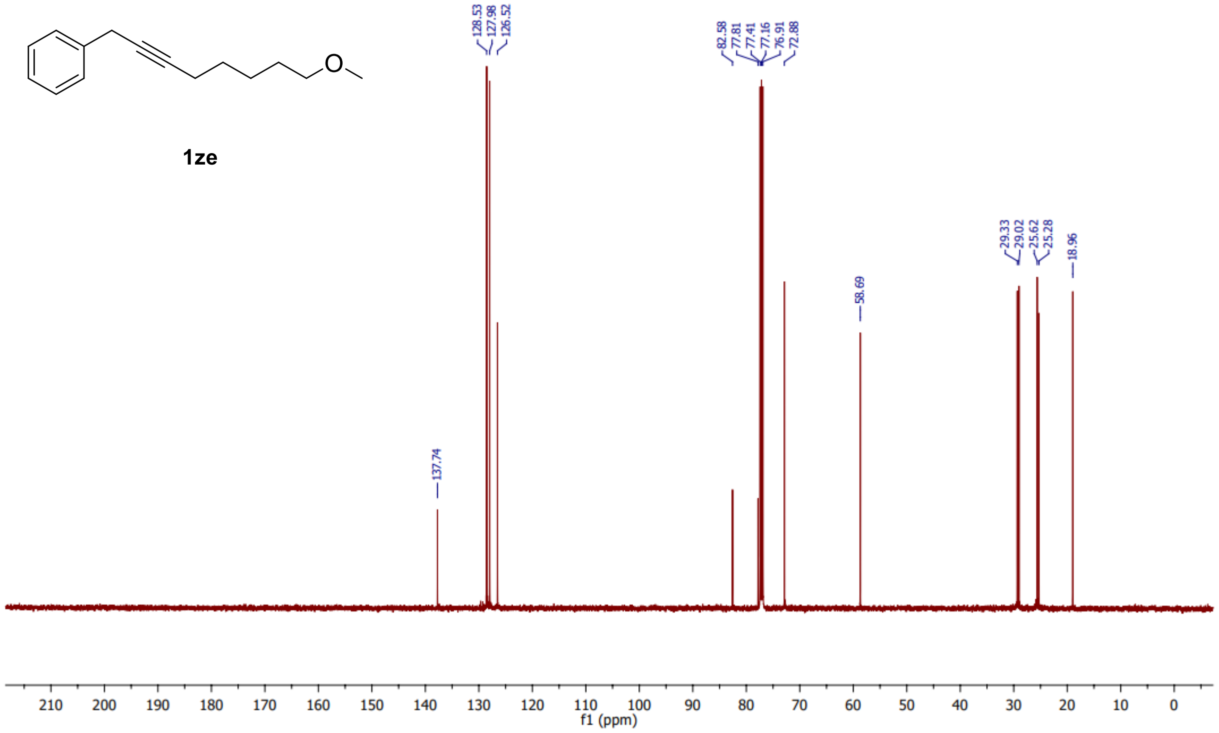

^{1}H NMR (500 MHz, $CDCl_3$)

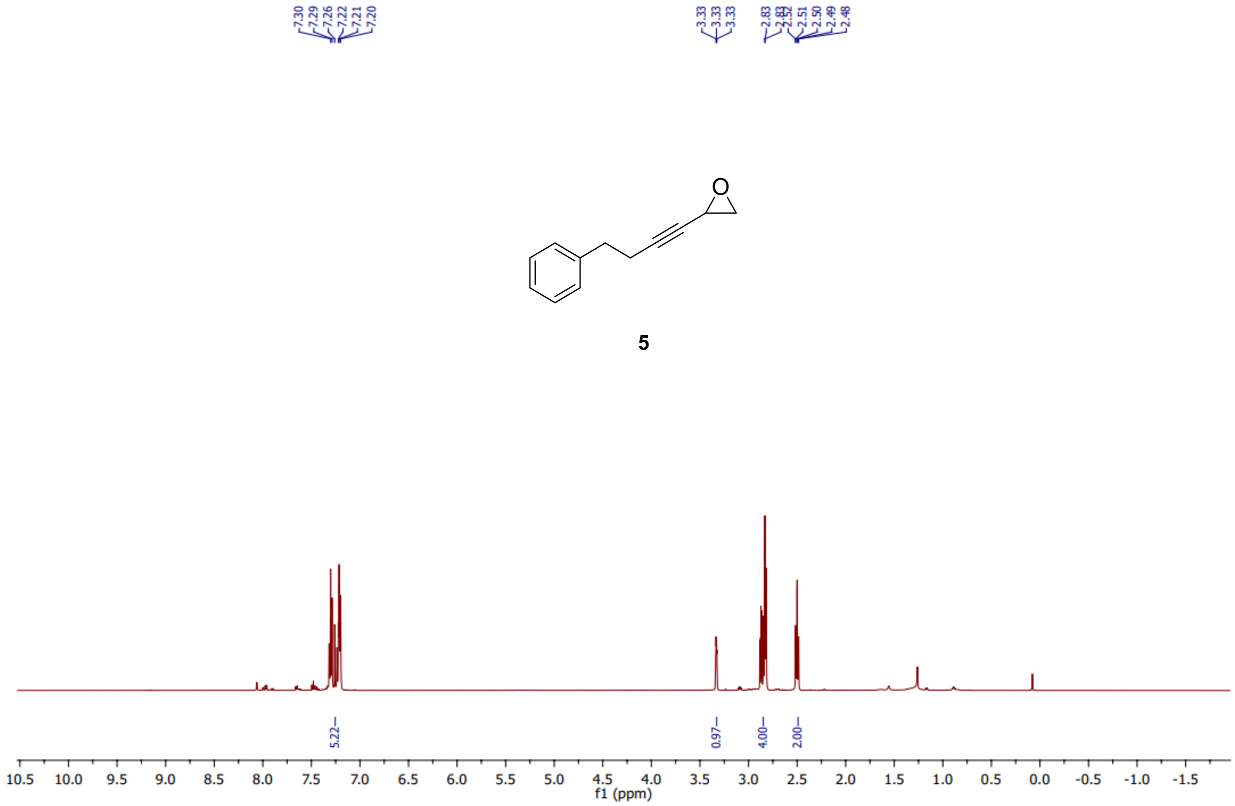

5

^{13}C NMR (125 MHz, $CDCl_3$)

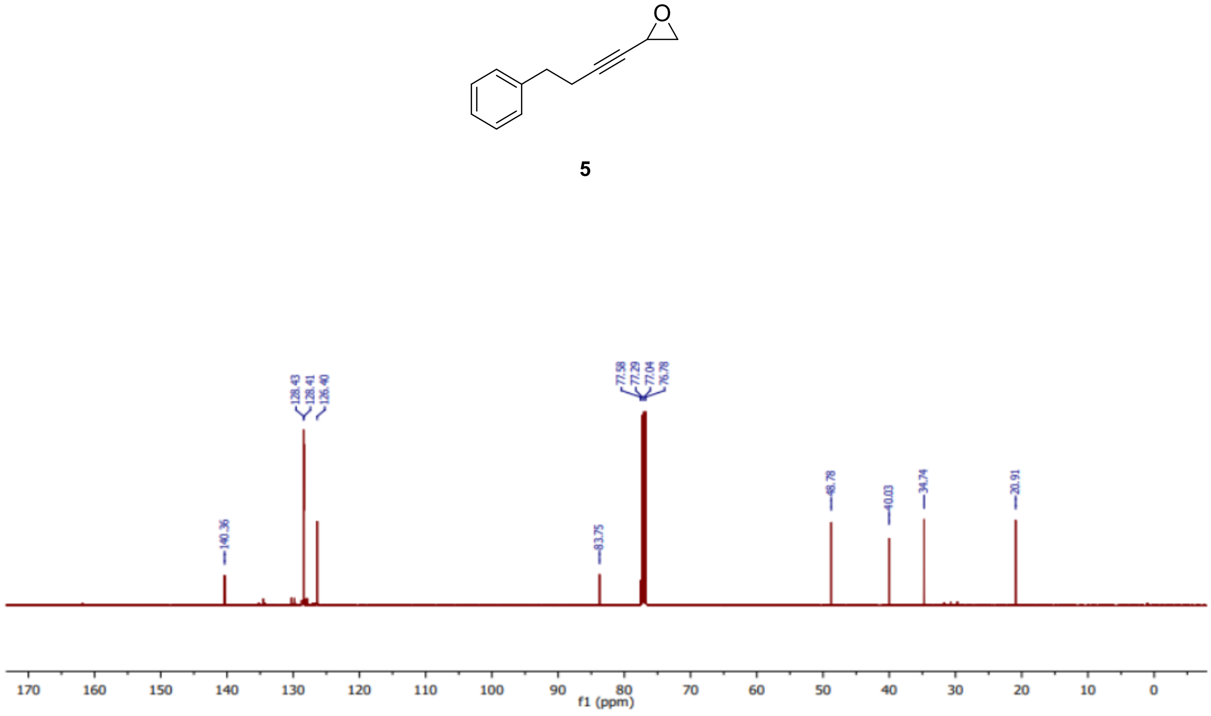

5

^{1}H NMR (500 MHz, $CDCl_3$)

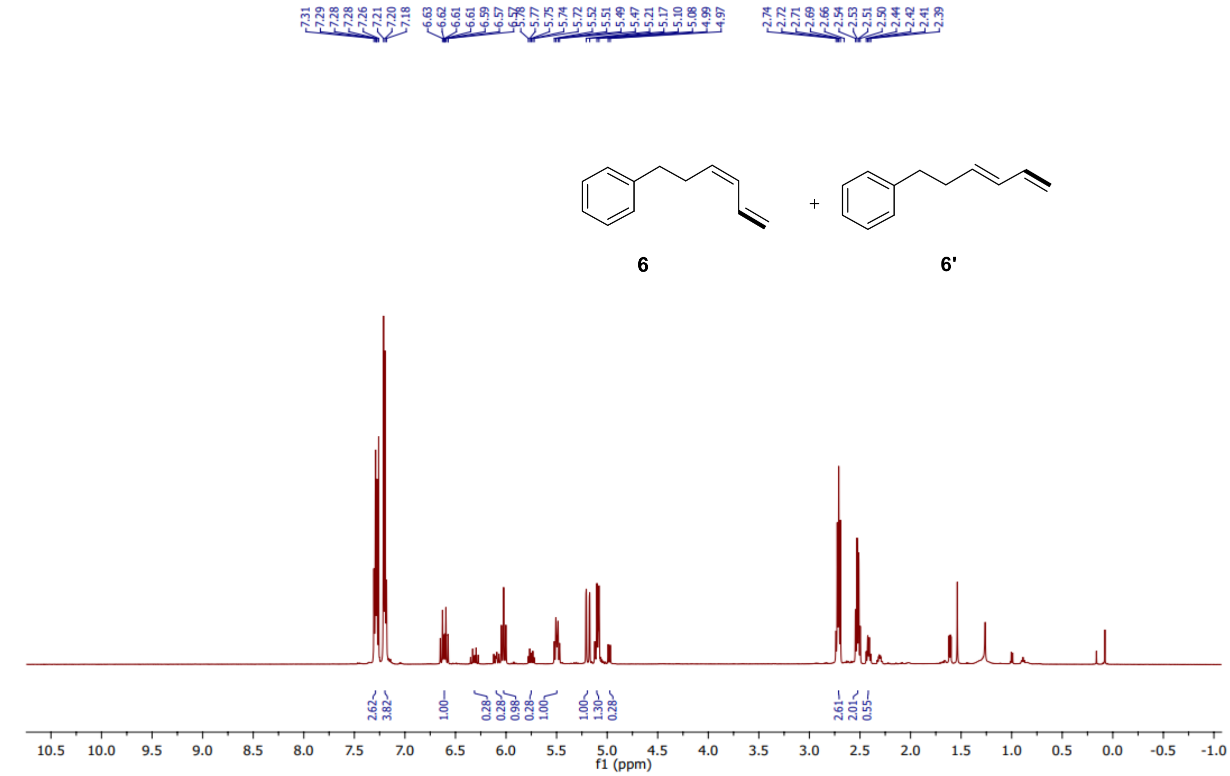

^{13}C NMR (125 MHz, $CDCl_3$)

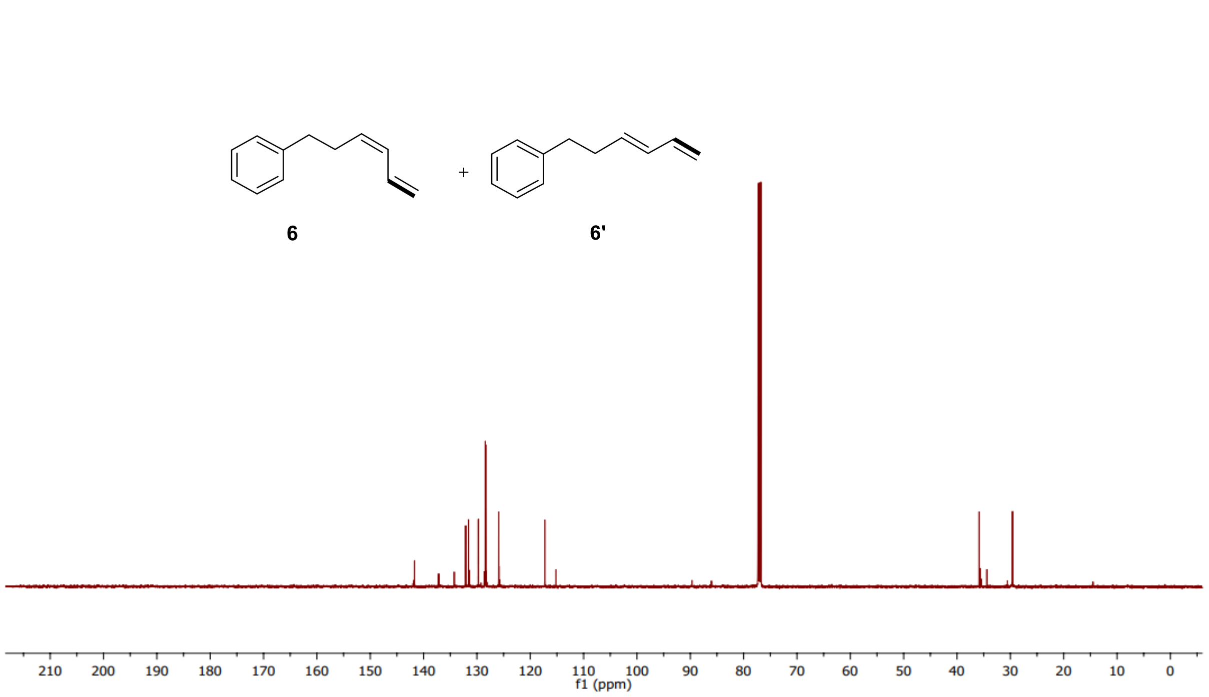

^{1}H NMR (500 MHz, $CDCl_3$)

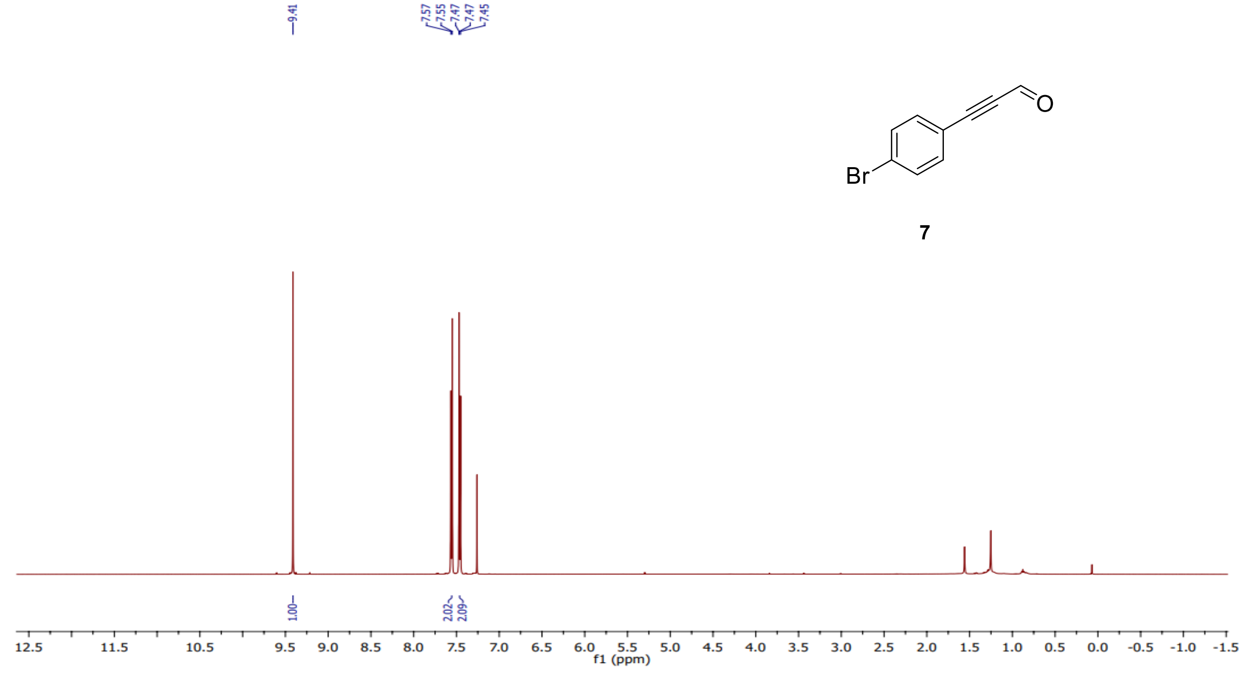

^{13}C NMR (125 MHz, $CDCl_3$)

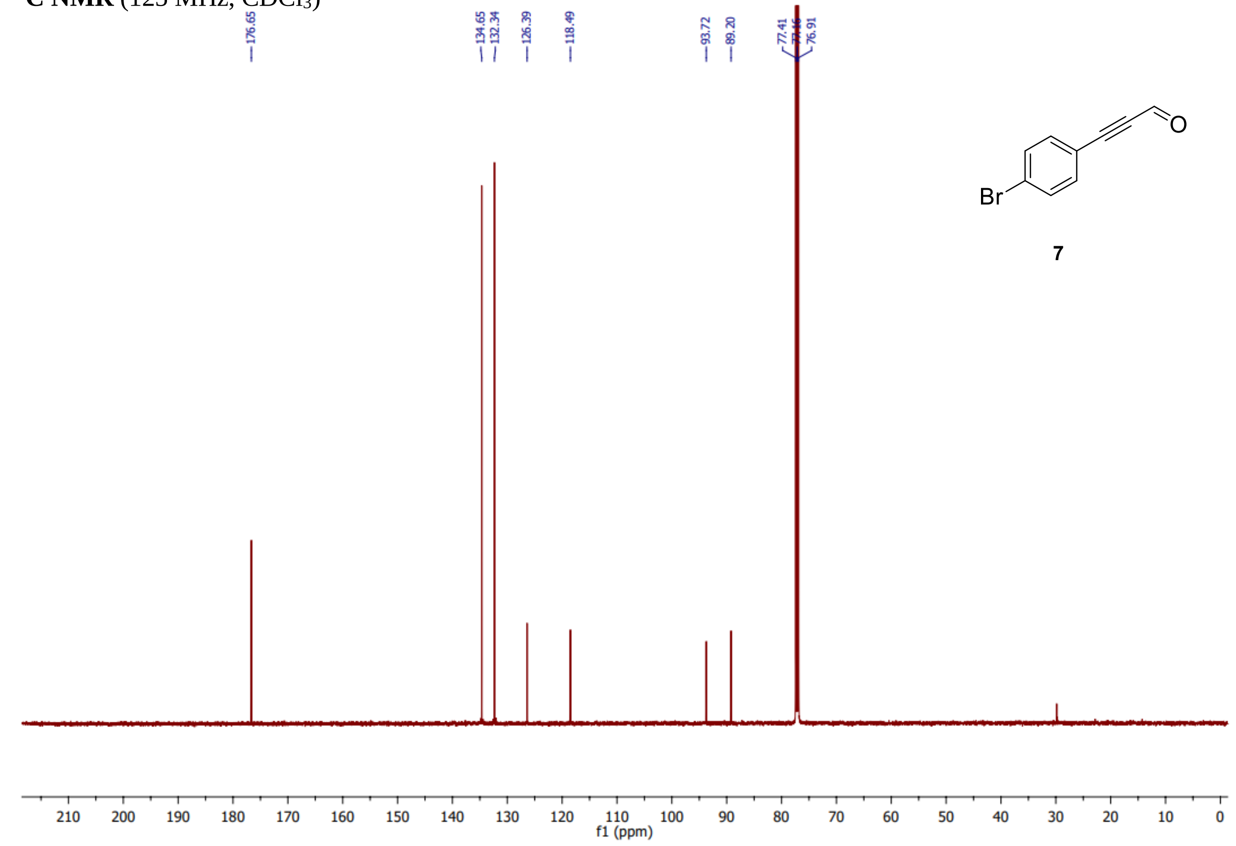

^{1}H **NMR** (500 MHz, $CDCl_3$)

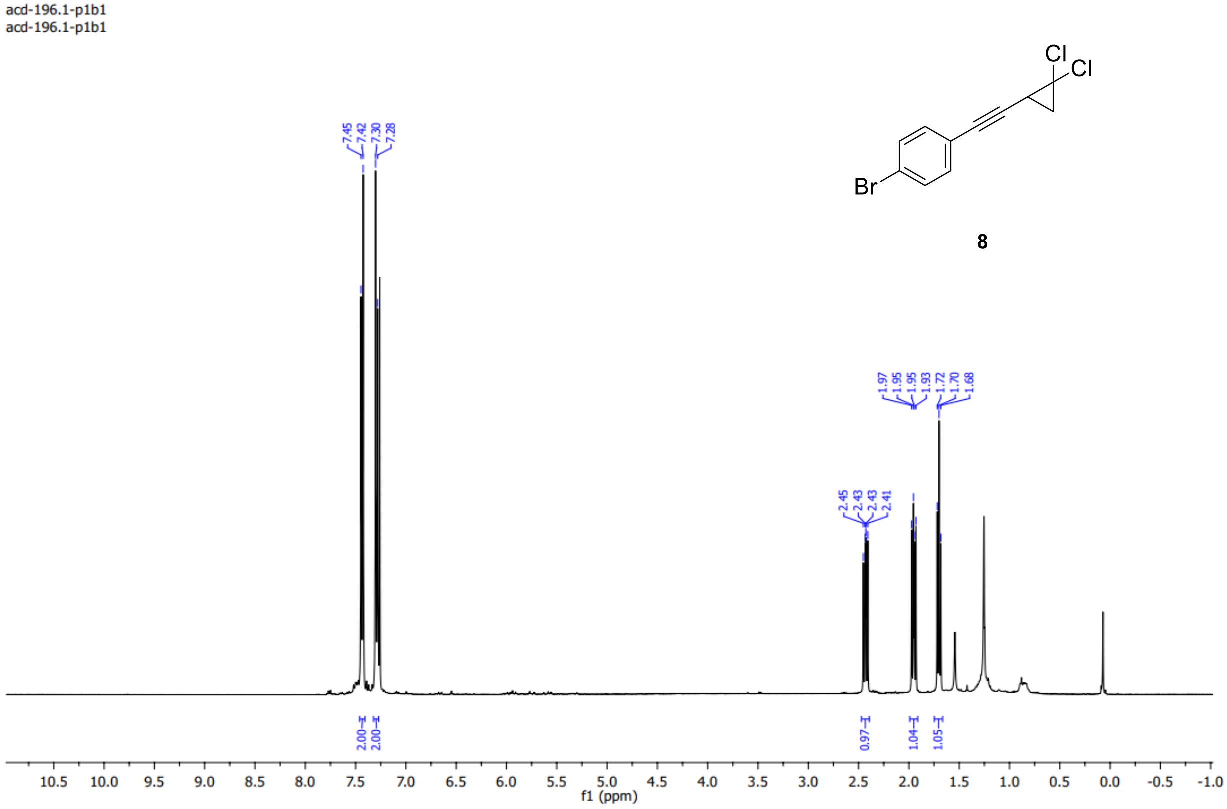

^{13}C **NMR** (125 MHz, $CDCl_3$)

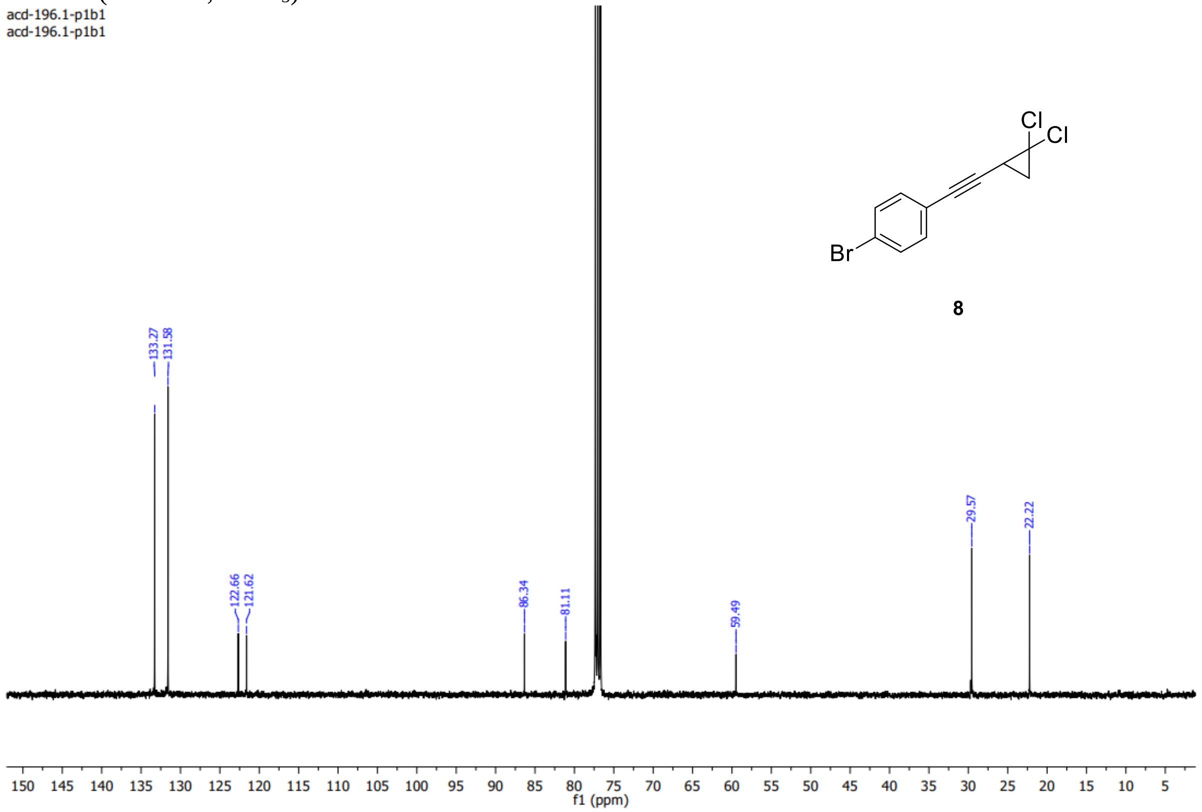

^{1}H NMR (300 MHz, $CDCl_3$)

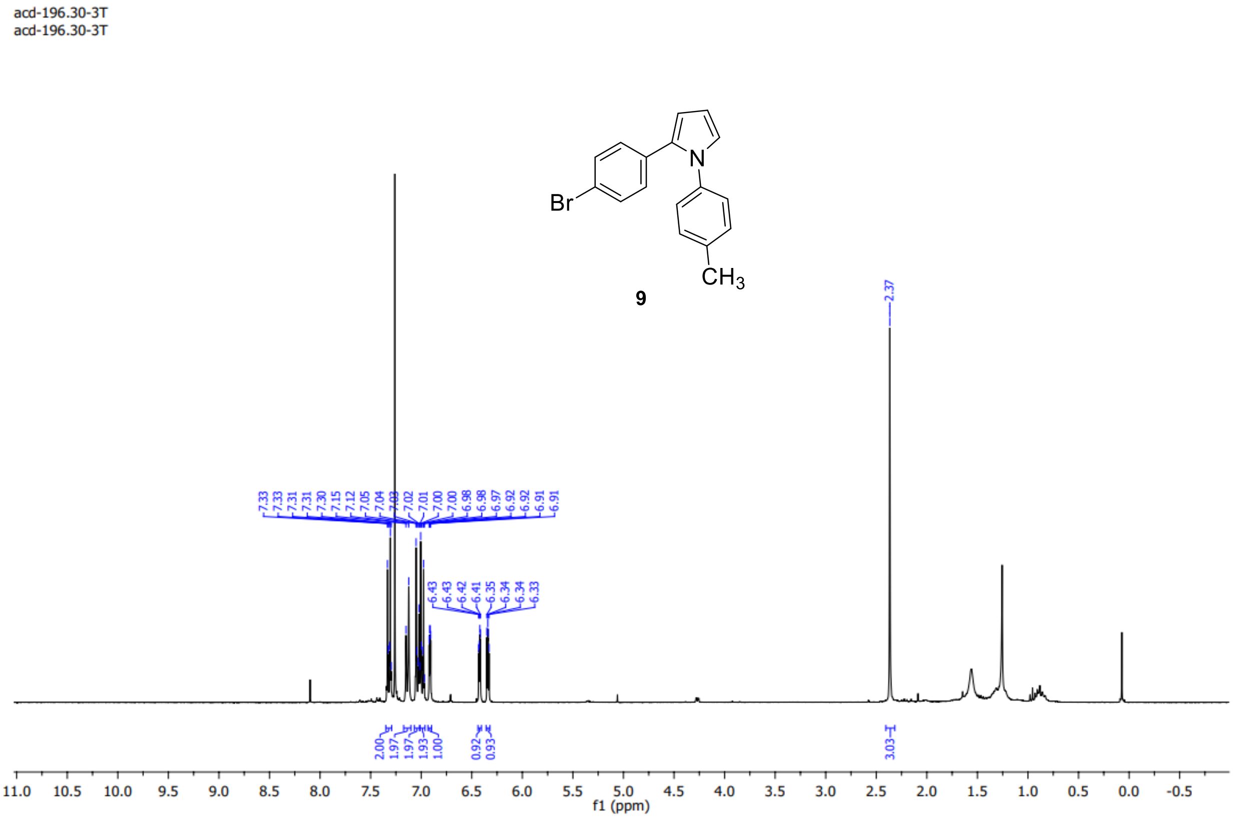

^{1}H NMR (400 MHz, $CDCl_3$)

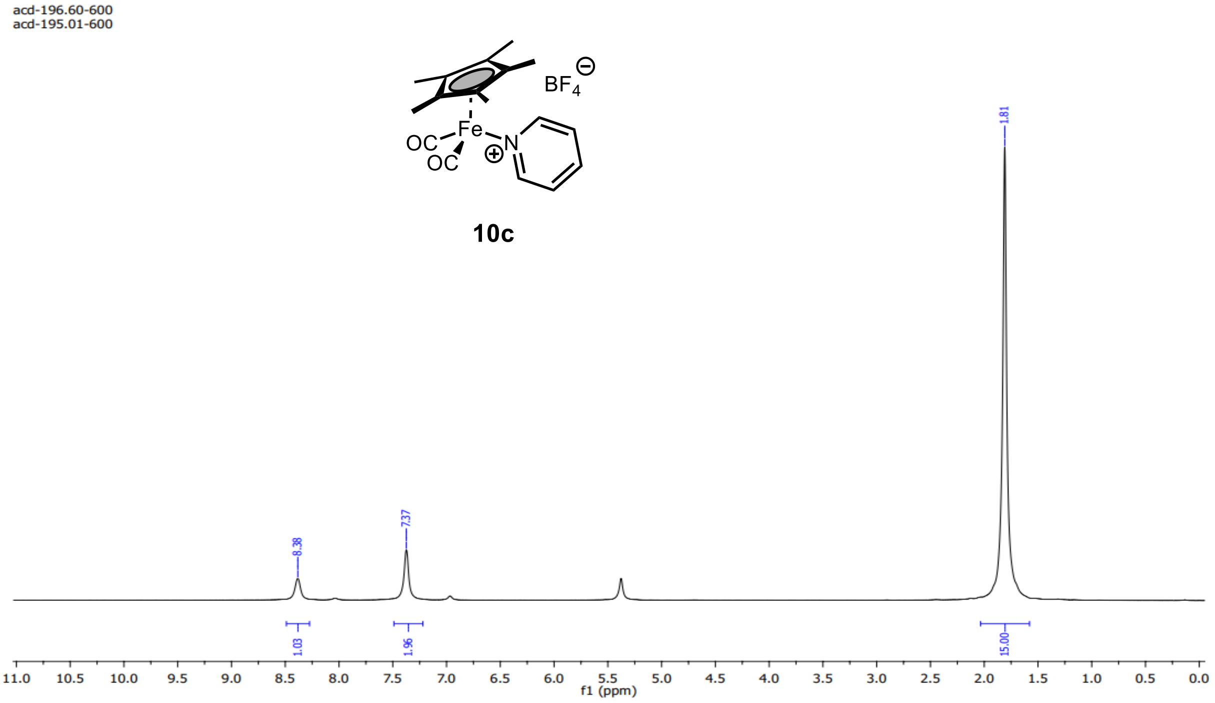

^{19}F NMR (376 MHz, $CDCl_3$)

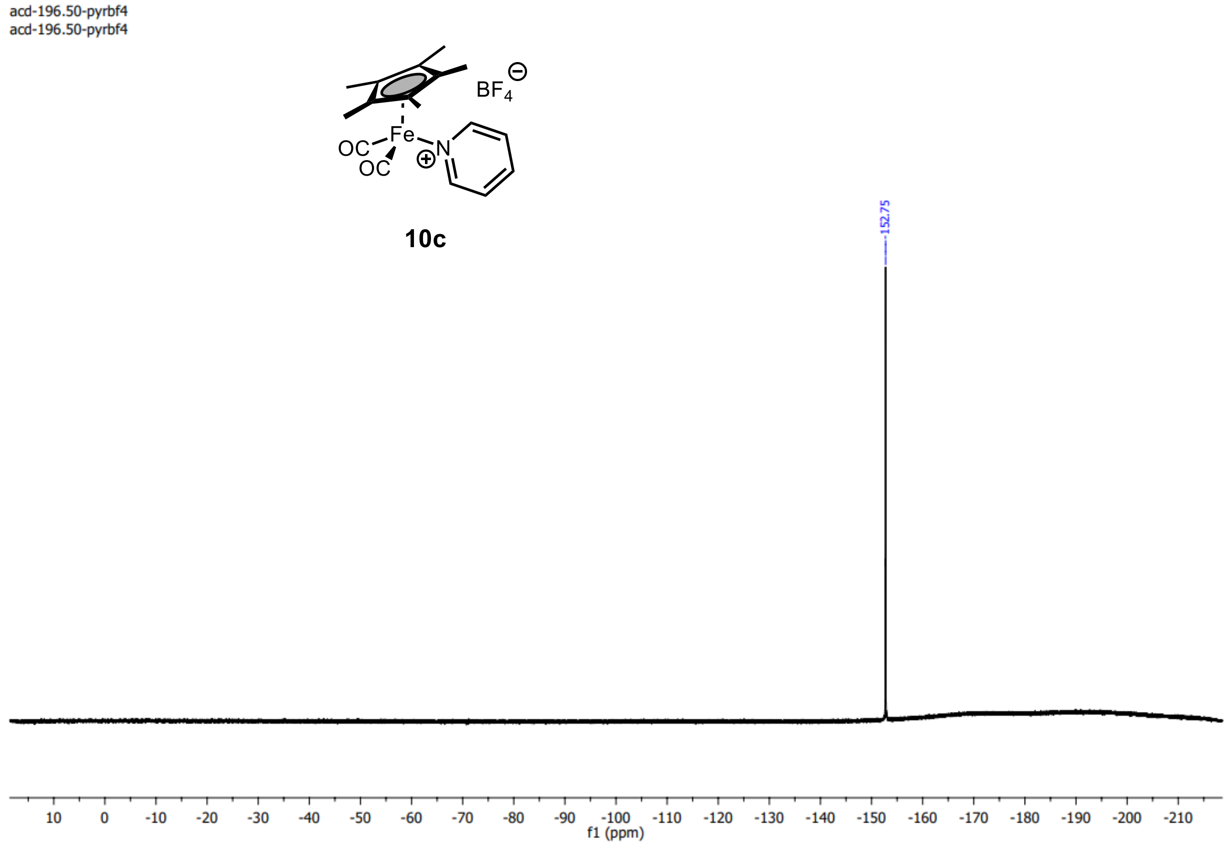

^{13}C NMR (101 MHz, $CDCl_3$)

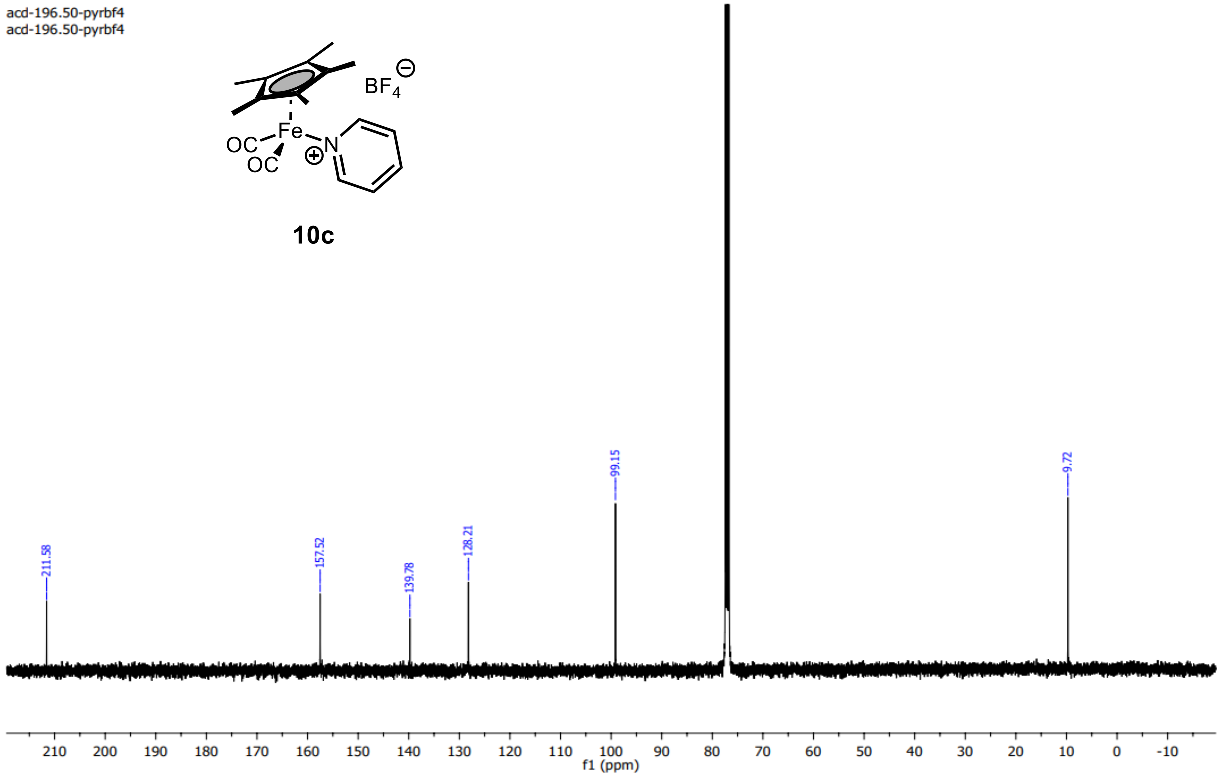

^{1}H NMR (500 MHz, $CDCl_3$)

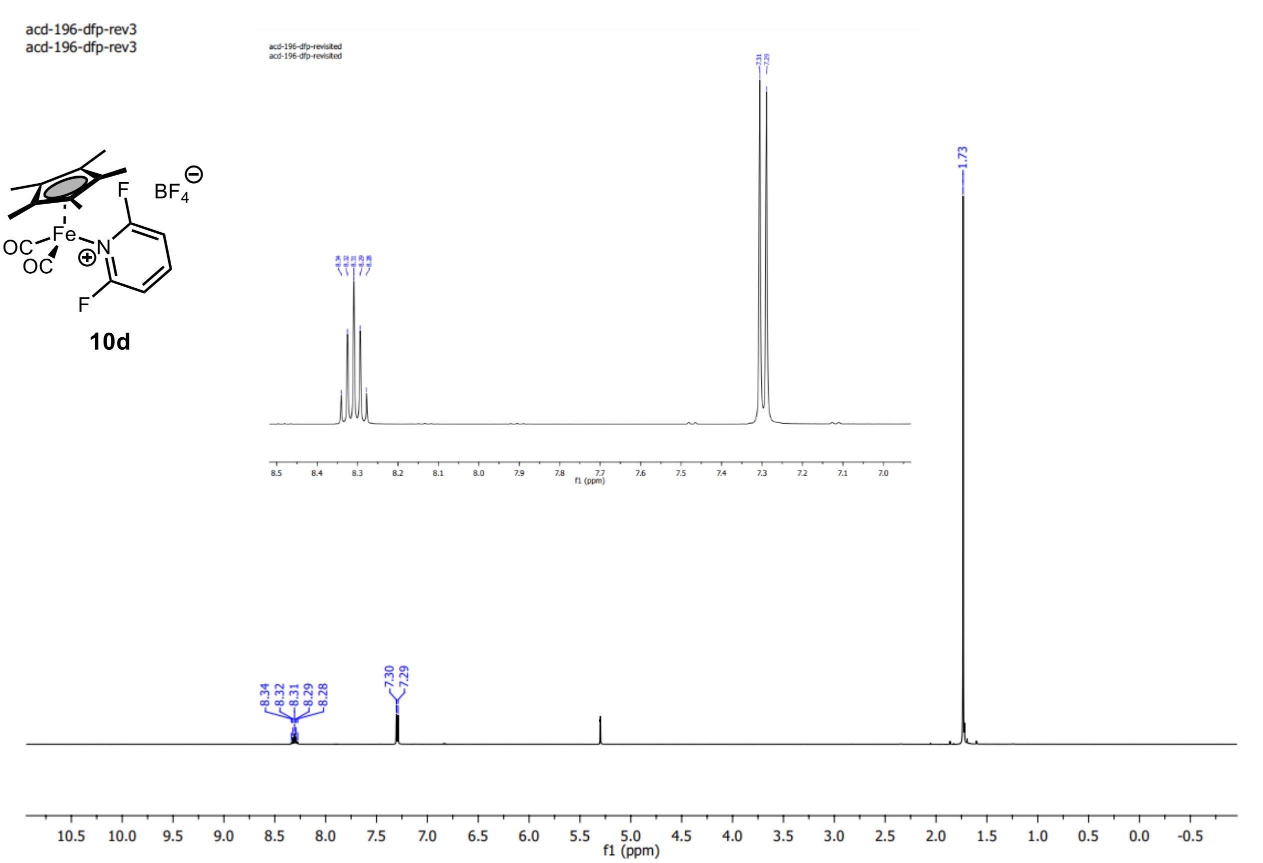

^{13}C NMR (125 MHz, $CDCl_3$)

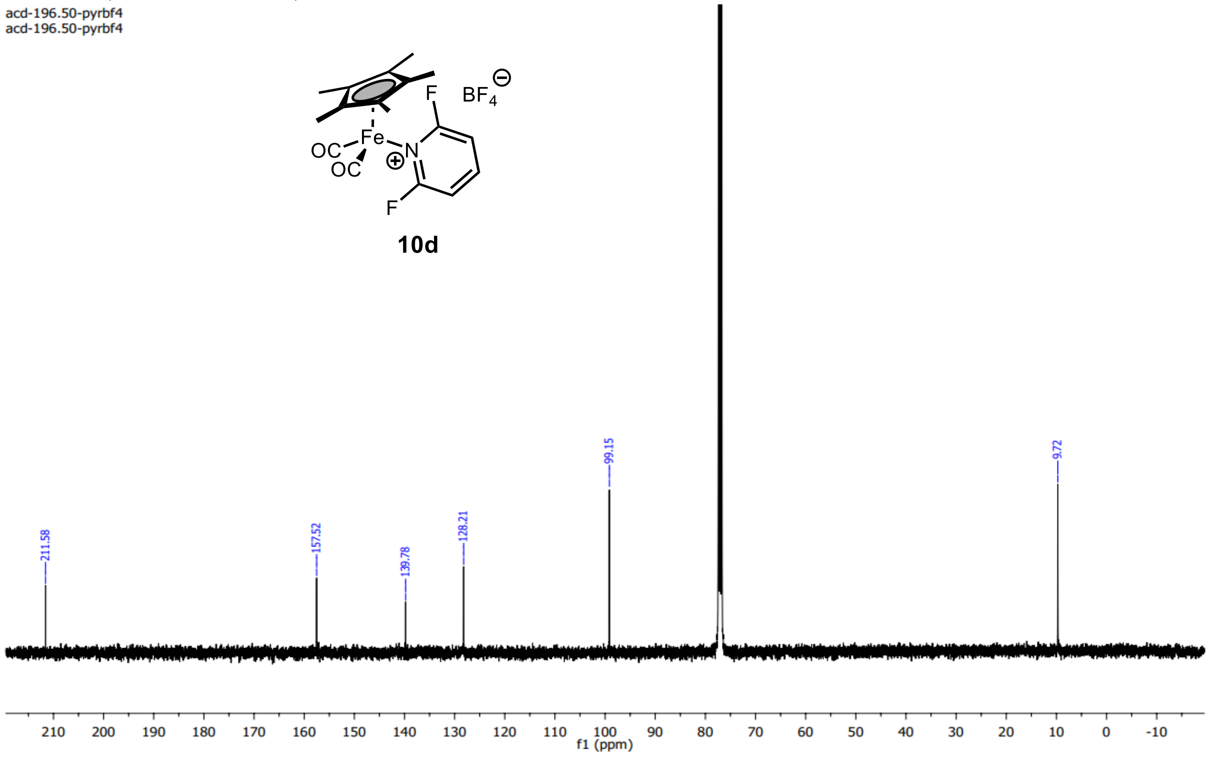

^{19}**F NMR** (470 MHz, $CDCl_3$)

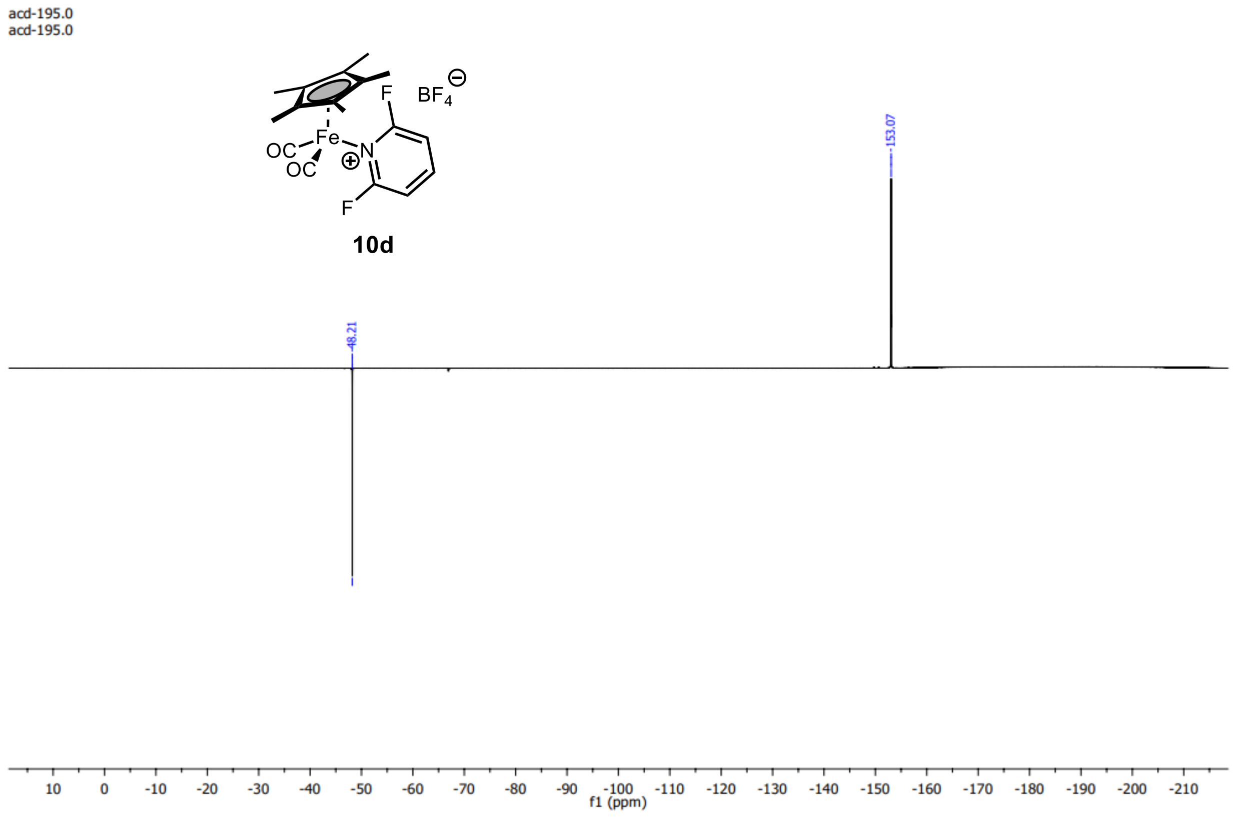

IR Data:

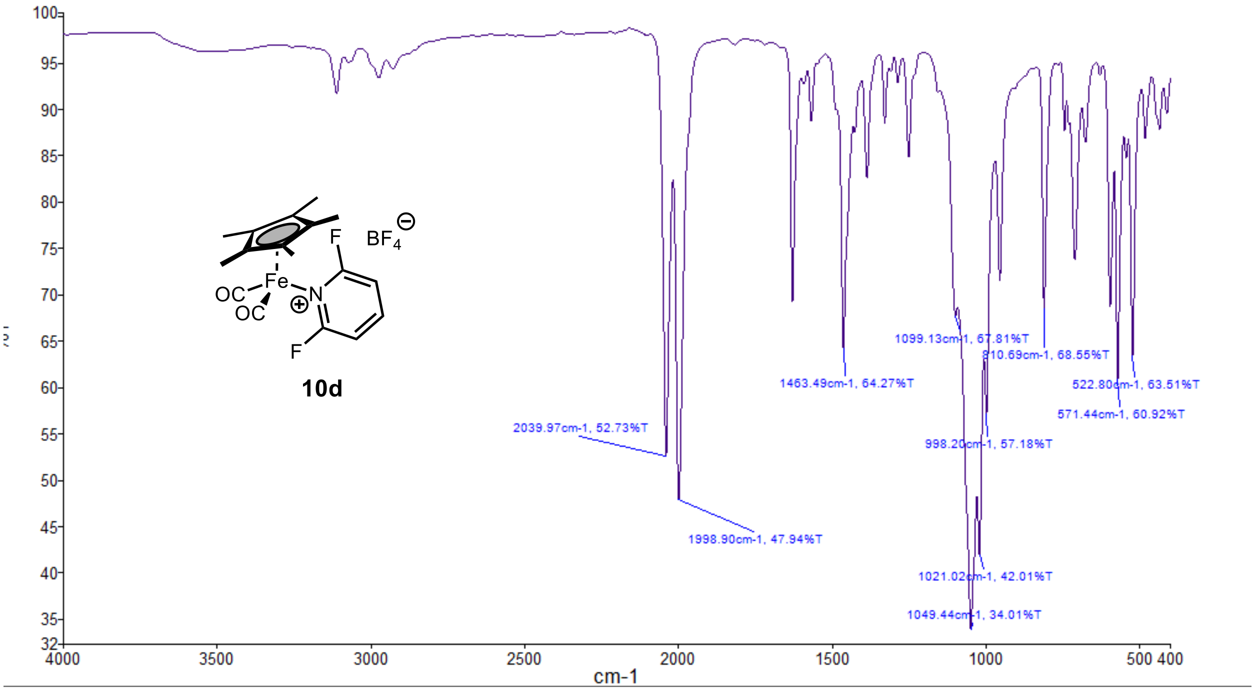

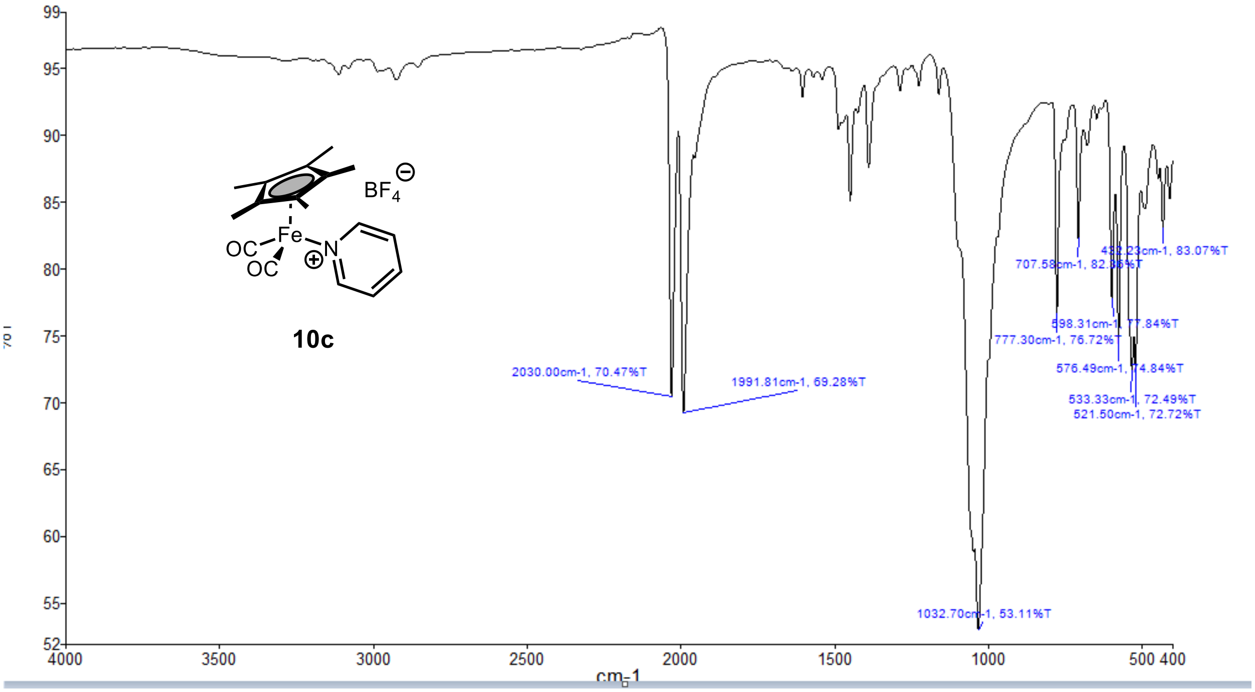

Supplement: Supplementary file 1 — ol4c00696_si_001.pdf [file ol4c00696_si_001.pdf]
